# An Umbrella Review of Meta-Analyses Evaluating Associations between Human Health and Exposure to Major Classes of Plastic-Associated Chemicals

**DOI:** 10.5334/aogh.4459

**Published:** 2024-08-19

**Authors:** Christos Symeonides, Edoardo Aromataris, Yannick Mulders, Janine Dizon, Cindy Stern, Timothy Hugh Barker, Ashley Whitehorn, Danielle Pollock, Tania Marin, Sarah Dunlop

**Affiliations:** 1 Minderoo Foundation, Perth, Western Australia, Australia; 2 Centre for Community Child Health, Royal Children’s Hospital, Parkville, Victoria, Australia; 3 JBI, Faculty of Health and Medical Sciences, The University of Adelaide, Adelaide, South Australia, Australia; 4 College of Nursing and Health Sciences, Flinders University, Adelaide, South Australia, Australia; 5 School of Biological Sciences, The University of Western Australia, Perth, Australia

**Keywords:** Umbrella review, Plastics, Plastic-associated chemicals, Human health, Evidence synthesis

## Abstract

*Background:* Epidemiological research investigating the impact of exposure to plastics, and plastic-associated chemicals, on human health is critical, especially given exponentially increasing plastic production. In parallel with increasing production, academic research has also increased exponentially both in terms of the primary literature and ensuing systematic reviews with meta-analysis. However, there are few overviews that capture a broad range of chemical classes to present a state of play regarding impacts on human health.

*Methods:* We undertook an umbrella review to review the systematic reviews with meta-analyses. Given the complex composition of plastic and the large number of identified plastic-associated chemicals, it was not possible to capture all chemicals that may be present in, and migrate from, plastic materials. We therefore focussed on a defined set of key exposures related to plastics. These were microplastics, due to their ubiquity and potential for human exposure, and the polymers that form the matrix of consumer plastics. We also included plasticisers and flame retardants as the two classes of functional additive with the highest concentration ranges in plastic. In addition, we included bisphenols and per- and polyfluoroalkyl substances (PFAS) as two other major plastic-associated chemicals with significant known exposure through food contact materials. Epistemonikos and PubMed were searched for systematic reviews with meta-analyses, meta-analyses, and pooled analyses evaluating the association of plastic polymers, particles (microplastics) or any of the selected groups of high-volume plastic-associated chemicals above, measured directly in human biospecimens, with human health outcomes.

*Results:* Fifty-two systematic reviews were included, with data contributing 759 meta-analyses. Most meta-analyses (78%) were from reviews of moderate methodological quality. Across all the publications retrieved, only a limited number of plastic-associated chemicals within each of the groups searched had been evaluated in relevant meta-analyses, and there were no meta-analyses evaluating polymers, nor microplastics. Synthesised estimates of the effects of plastic-associated chemical exposure were identified for the following health outcome categories in humans: birth, child and adult reproductive, endocrine, child neurodevelopment, nutritional, circulatory, respiratory, skin-related and cancers.

Bisphenol A (BPA) is associated with decreased anoclitoral distance in infants, type 2 diabetes (T2D) in adults, insulin resistance in children and adults, polycystic ovary syndrome, obesity and hypertension in children and adults and cardiovascular disease (CVD); other bisphenols have not been evaluated. Phthalates, the only plasticisers identified, are associated with spontaneous pregnancy loss, decreased anogenital distance in boys, insulin resistance in children and adults, with additional associations between certain phthalates and decreased birth weight, T2D in adults, precocious puberty in girls, reduced sperm quality, endometriosis, adverse cognitive development and intelligence quotient (IQ) loss, adverse fine motor and psychomotor development and elevated blood pressure in children and asthma in children and adults. Polychlorinated biphenyls (PCBs), polybrominated diphenyl ethers (PBDEs) but not other flame retardants, and some PFAS were identified and are all associated with decreased birth weight. In general populations, PCBs are associated with T2D in adults and endometriosis, bronchitis in infants, CVD, non-Hodgkin’s lymphoma (NHL) and breast cancer. In PCB-poisoned populations, exposure is associated with overall mortality, mortality from hepatic disease (men), CVD (men and women) and several cancers. PBDEs are adversely associated with children’s cognitive development and IQ loss. PBDEs and certain PFAS are associated with changes in thyroid function. PFAS exposure is associated with increased body mass index (BMI) and overweight in children, attention deficit hyperactive disorder (ADHD) in girls and allergic rhinitis. Potential protective associations were found, namely abnormal pubertal timing in boys being less common with higher phthalate exposure, increased high-density lipoprotein (HDL) with exposure to mono(2-ethyl-5-oxohexyl) phthalate (MEOHP) and reduced incidence of chronic lymphocytic lymphoma (a subtype of NHL) with PCB exposure.

*Conclusions:* Exposure to plastic-associated chemicals is associated with adverse outcomes across a wide range of human health domains, and every plastic-associated chemical group is associated with at least one adverse health outcome. Large gaps remain for many plastic-associated chemicals.

*Recommendations:* For research, we recommend that efforts are harmonised globally to pool resources and extend beyond the chemicals included in this umbrella review. Priorities for primary research, with ensuing systematic reviews, could include micro- and nanoplastics as well as emerging plastic-associated chemicals of concern such as bisphenol analogues and replacement plasticisers and flame retardants. With respect to chemical regulation, we propose that safety for plastic-associated chemicals in humans cannot be assumed at market entry. We therefore recommend that improved independent, systematic hazard testing for all plastic-associated chemicals is undertaken before market release of products. In addition because of the limitations of laboratory-based testing for predicting harm from plastic in humans, independent and systematic post-market bio-monitoring and epidemiological studies are essential to detect potential unforeseen harms.

## Introduction

Plastic is ubiquitous in our daily lives, being used in transport, agriculture, construction, and medical and pharmaceutical products, as well as food packaging [1]. Plastics are complex compounded materials comprising a polymer backbone combined with chemical additives such as plasticisers, flame retardants, ultra violet (UV), light and heat stabilisers, biocides and colourants. Other chemicals include processing aids and non-intentionally added substances (NIAS) such as impurities in feedstock materials, by-products of polymer production, degradation and transformation products, and contaminants from processing machinery [2–5]. Over 8,300 million metric tonnes (MMT) of virgin plastic has been produced [1] with an annual production of over 400 MMT predicted to triple by 2060 [6].

In parallel with increasing plastic production, there is increasing recognition of the health implications of ‘plastic-associated chemicals’ [3,7]. Additives are, for the most part, not covalently bound to the polymer [8]. Monomers may also leach from products over time as residual unreacted monomers or break down products, as may residual processing aids and NIAS as above [9]. These can then enter the human body via ingestion [10, 11], inhalation [12–15] or transdermally [15–18]. Consequently, commonly studied plastic-associated chemicals have been detected and are reliably measured in human biosamples across the human lifespan, from prenatally (amniotic fluid) through childhood to adulthood [19] and in the elderly [20].

Of the over 16,000 estimated monomers, additives and processing agents identified in regulatory databases as being used in plastics, only a minority are subject to global regulation while the majority lack hazard information [5, 21, 22]. However, where completed, pre-market in vitro or in vivo toxicological assessments have limitations regarding long-term low-dose exposure, availability of appropriate models for complex human health endpoints, suboptimal experimental animal study design and reporting with high risk of bias [23].

Nevertheless, after the introduction of plastic products to market, health effects can be directly evaluated in humans through observational research. Observational study designs, including cohort, case control and cross-sectional studies, are generally the most appropriate to assess risk of, and association with, adverse health outcomes, where controlled experimental exposure in humans would not be ethical [23]. Observational studies require reliable, sensitive methodologies to quantify individual exposure to the chemical or its metabolites in biosamples. These include availability of samples to quantify exposure at biologically relevant times, data in individuals on health outcome and potential confounding factors as well as sufficient numbers of individuals to reliably detect associations. However, there is no routine regulatory health surveillance of industrial chemicals such as those present in plastics, and the chemicals investigated by academic research studies typically cover only a small fraction of high-volume chemicals in production [24]. Indeed, a recent systematic evidence map has compiled the primary research on plastic and commonly studied plastic-associated chemicals, and revealed that only 25% of the searched chemicals have been studied in humans [25].

However, individual observational primary research studies are often limited by sample size, distribution of exposure, timing of exposure measurements (e.g., one-time urine measurements), outcome and other characteristics of the population sampled and/or difficulties in interpreting findings across multiple studies. Synthesis of findings is beneficial in evaluating the overall evidence base (e.g., for regulatory decisions). Systematic reviews with meta-analyses, meta-analyses, and pooled analyses draw on multiple primary research studies to combine statistical estimates of association for a single estimate.

A large number of systematic reviews with meta-analyses, meta-analyses, and pooled analyses have evaluated evidence of association between exposure to plastic-associated chemicals and human health outcomes such as cancer, pregnancy, and disorders of metabolic, cardiovascular and neurological systems [7]. Only a few attempts have been made to subsequently review these existing systematic reviews, and these are limited to specific plastic-associated chemicals or chemical classes, namely phthalates (‘overview of reviews’) and bisphenols (‘umbrella review’) [26, 27] and, to a very limited extent, in a broad umbrella review of all environmental risk factors for health [28].

Umbrella reviews are a recognised approach to conduct a systematic and standardised evaluation of a broad research topic for which there are multiple published systematic reviews and meta-analyses available [29]. They are regarded as one of the highest levels of evidence synthesis [30].

In setting the scope of the plastics and plastic-associated chemicals to be considered in this umbrella review, it was not possible to capture all chemicals that may be present in, and migrate from, plastic materials. Although increasingly large numbers of plastic-associated chemicals are being identified [5, 21, 22], the full extent of additional plastic-associated chemicals is unknown, especially for NIAS [9, 11, 31, 32]. In this umbrella review, we therefore focussed on a defined set of key exposures related to plastics. We included microplastics, due to their ubiquity and potential for human exposure [33] and the polymers that form the matrix of consumer plastics. We also included plasticisers and flame retardants as the two classes of functional additive with the highest concentration ranges in plastic [34]. In addition, we included bisphenols and a number of PFAS as two other major plastic-associated chemicals with significant known exposure through food contact materials [9].

Our umbrella review synthesises and presents findings from the meta-analytic literature examining associations between plastic-associated chemical exposure and human health outcomes across the lifespan.

## Methods

We followed established umbrella review methods [29] including an a priori protocol, key details of which were prospectively registered on the International Prospective Register of Systematic Reviews (PROSPERO) (CRD42020204893) and reported according to the Preferred Reporting Items for Systematic Reviews and Meta-Analyses (PRISMA) 2020 guidelines [35]. We used vote counting and harvest plots to assimilate the large and diverse data on plastic and plastic-associated chemical exposure and human health outcomes across the lifespan. A glossary of chemical abbreviations used is available in the supplementary materials (Suppl File 1.1).

### Search strategy

Epistemonikos, a comprehensive database of systematic reviews for health decision-making (https://www.epistemonikos.org) [36], and PubMed were searched on 26 August and 30 September 2020, respectively (JD; Suppl File 1.2). Search filters employed a combination of terms (and indexing terms in PubMed). We included broad terms such as ‘plastic’ alongside terms relating to functional terminology such as ‘plasticiser/plasticizer’ and ‘flame retardant.’ We also included common-use terminology and abbreviations such as ‘phthalates’ and ‘PVC’, and technical chemical terminology such as 4,4’-isopropylidenediphenol (bisphenol A) and di(2-ethylhexyl) phthalate (DEHP). Search terms encompassed microplastic particles; nanoplastics were not separately searched because reliable analytical techniques to quantify individual human exposure to these smaller particles, and therefore the opportunities for direct observational research, were not yet available. For plastic polymers, all major commodity polymers were considered: polyethylene, polypropylene, polyethylene terephthalate, polyvinyl chloride, polycarbonates, polystyrene, nylon(s) and fluoropolymers, including polytetrafluoroethylene. For plasticisers and flame retardants, our search terms were selected to capture all major chemical classes [25], including (ortho- and tere-) phthalates, cyclohexanoates, adipates, sebacates, trimellitates, dibenzoates, citrate esters, organophosphate esters (OPEs), PCBs, PBDEs and polybrominated biphenyls (PBBs). We also included a range of specific and general terms to capture other plasticisers or flame retardants not included in these major classes (decabromodiphenyl ethane, hexabromocyclododecane, any other polybrominated or polychlorinated chemicals and melamine polyphosphate). Bisphenols and PFAS were separately searched using a range of terms capturing common-use and technical terminology for these classes, and major chemicals within these classes. We also used specific search terms for flame-retardant bisphenols such as the halogenated bisphenol tetrabromobisphenol A and the organophosphate bisphenol A diphenyl phosphate. No date limits were applied; however, filters were applied to both databases to limit to systematic reviews. Grey literature was not included.

### Eligibility criteria

Eligibility criteria were aligned to the population, exposure, comparator and outcome (PECO) framework [37] (Table 1). We thus captured meta-analyses (i.e., systematic reviews with meta-analyses, meta-analyses, and pooled analyses) of studies that evaluated the association between exposure to plastic particles and plastic-associated chemicals and human health outcomes. This included environmental as well as occupational exposure and poisoning. We also captured any human health outcome irrespective of age. Participants could be healthy or have pre-existing illness.

**Table 1:** Details of the population, exposure, comparator, outcome (PECO) framework.

COMPONENT	DESCRIPTION
Population	General population exposed through environment or poisoning. Occupational exposure to plastic-associated chemicals is included, except if the occupational exposure occurs through plastic manufacturing or fossil fuel extraction. Exposure through medical, surgical, or dental devices such as prostheses or implants was also excluded. Subgroup analyses focusing on population differences (e.g. age, gender) were included.
Exposure	Plastic-associated chemical exposure, considering comparisons of high vs. low exposure, any vs. none, and any linear or non-linear dose responses. Composite exposure to groups of chemicals (e.g. total phthalates, total polychlorinated biphenyls [PCBs]) and subgroup analyses of individual chemicals (e.g. specific phthalate diesters, specific PCB congeners) were included. Exposure measurements are required to be from human bio-samples. Indirect exposure measures (e.g. questionnaires, dust) were excluded.
Comparator	Comparisons within the general population, such as high vs. low exposure and any vs. none, without occupational, medical device-related, or indirect exposure measures.
Outcome	Health outcomes reported using statistical measures (e.g. relative risks [RR], odds ratios [OR], or regression coefficients). Meta-analyses needed to present separate analyses for different health outcomes and meet the primary or secondary analysis criteria of the reviewed articles.

Eligible exposures are shown in Suppl File 1.2. Meta-analyses examining exposure to other additives (e.g., antimicrobials, antioxidants, antistatic agents, fillers, processing agents, and UV, light and heat stabilisers) or combined exposures were not included. Meta-analyses of studies investigating endocrine-disrupting chemicals that included plastic polymers or additives were eligible for inclusion, but only if evaluated separately from chemicals that were not plastic related.

We included any analysis with comparisons of plastic-associated chemical exposure, including high versus low, any versus none, and any linear or non-linear dose responses. Meta-analyses of studies were ineligible if they included studies where measures of exposure were indirect (e.g., questionnaire-based surveys, dust), where exposure was attributable to an occupation in plastic manufacturing or fossil fuel extraction, or in the presence of a medical, surgical or dental device such as a prosthesis or implant. If an article presented separate meta-analyses for more than one health outcome (and any combination of exposures), we included each of these separately, recording whether extracted estimates related to the primary analysis (or analyses) of the paper, or related to a secondary analysis. Articles that did not present a meta-analysis or statistical combination of multiple studies for a health outcome, with a measure such as relative risks (RR), odds ratios (OR) or regression coefficients, were ineligible. Analyses of composite exposure to a group of plastic-associated chemicals were included, as well as subgroup analyses investigating individual chemicals (such as total phthalates and individual phthalate diesters or total PCBs and specific PCB congeners). Other subgroups that further investigated population differences (age, gender) and differences in measurement of exposure (e.g., serum, urine) aligned to the main analyses of the included reviews were also included. Only reviews and analyses published in English were included.

### Selection and assessment of methodological quality

Citations from database searching were uploaded into EndNote v9 (Clarivate Analytics) and duplicates removed. Titles and abstracts of remaining records were subsequently screened independently by two reviewers (JD, DP) considering the eligibility criteria. Full text of potentially relevant reviews and syntheses were retrieved and reviewed (JD, DP); where necessary, inclusion was determined by discussion between reviewers.

Methodological quality of eligible systematic reviews with meta-analyses, meta-analyses, and pooled analyses was independently assessed by two reviewers (JD, TB, DP, TM, AW). Umbrella review methodology appraises the quality of reporting of the systematic review, and not directly the quality of the primary research included therein. We used the ‘A MeaSurement Tool to Assess systematic Reviews’ (AMSTAR) tool [38], an 11-item checklist designed to assess methodological quality of systematic reviews of interventions. AMSTAR has been shown to be a reliable and valid tool for quality assessment of systematic reviews and meta-analyses of observational research [39]. AMSTAR was selected due to more rapid completion and greater inter-rater reliability to mitigate multiple appraisers involved (JD, TB, DP, TM, AW) rather than other tools [40]. A pilot appraisal was undertaken on a subset of eligible reviews (10%) to maximise the reliability of the process between members of the review team (JD, TB, DP, TM, AW). A third reviewer (EA) resolved any disagreements. We established an arbitrary categorisation system to convey the appraisal findings: AMSTAR scores of 9–11 were rated as high quality (low risk of bias), 5–8 as moderate quality and less than 5 as low quality (high risk of bias). Rules used for consistency for each question are available in Suppl File 1.3. For expedience, the AMSTAR tool was also used to assess the quality of included pooled analyses. Because pooled analyses lack many design features inherent in a systematic review [41], we therefore scored them universally as ‘low’ in the quality appraisal.

### Data extraction

Data were extracted from the included reviews using a structured form in MS Excel (Microsoft) tailored to prompt retrieval of relevant information. Data extraction was performed independently by a member of the review team (JD, TB, AW, TM, DP) and all data extractions subsequently verified independently by the remaining team members (CSy, EA, CSt, YM). Extracted descriptive details were citation details, conflict of interest declaration, date of last search, included study designs, number of studies included (in the review and in the meta-analyses), critical appraisal tool used and results of appraisal, participants (characteristics and total number), plastic exposure (type, route, measure and time), health outcome(s) and measures reported and authors’ conclusions. Effect estimates (EE) from included meta-analyses (main findings or subgroup analyses) were extracted as OR, RR or standardised mortality ratios (SMR) for dichotomous data. Standardised, unstandardised, or z-transformed (z), beta (β) coefficients, correlation coefficients (r) or standardised (SMD) or unstandardised mean differences (MD) were extracted for continuous data. All data were extracted exactly as reported in the source publications, making no adjustments for number of decimal points or suspected extraction errors from the primary literature.

### Data summary and presentation

Health outcomes assessed with meta-analyses were aligned to corresponding chapters in the International Classification of Diseases, ICD-11 (https://icd.who.int/en). Considering the wide range of exposures, outcomes and outcome measures identified, it was not possible to estimate overall EE and therefore no further statistical meta-analysis of findings was considered [29, 42].

To synthesise data and establish evidence of effect across a large heterogeneous data set, we used vote counting with harvest plots [42]. In rare instances where the same exposure/outcome has been reported, the range of EE has been presented. The bars in the harvest plots represent individual EE (main or subgroup), placed on a matrix to indicate whether exposure to the plastic-associated chemical had a negative (decreased, left-hand column) or positive (increased, right-hand column) influence on the outcome based on the EE (point) reported. Where there was no influence, the direction of any non-significant effect is indicated as an increase (>), no change (–), or a decrease (<) in the measure or risk estimate (centre column) (Suppl File 1.4) [42]. Effect size is not portrayed within the harvest plots but is presented in the narrative and Suppl File 2.

The outcome or outcome measure reported is indicated in the first column of the harvest plot matrix, including whether outcomes were continuous (‡) or dichotomous (†). Given the heterogeneity of outcomes as well as methods of measurement and reporting, harvest plots were constructed as follows. Bars representing dichotomous outcome measures (relative estimates of risk) or continuous outcomes (regression coefficients, mean differences in measure between exposed versus low/non-exposed groups) were assigned as an increase or decrease in the measure where the change is statistically significant (p < 0.05). Where articles presented sensitivity analyses based on a meta-analytical model, considering the heterogeneity in study designs, populations, exposures and level of exposure, random effects were selected preferentially over fixed effects. Dark filled bars indicate the main analysis for each review, and light filled bars indicate subgroup analyses of the same participants within reviews. Reviews are indicated by the citation number (see Table 2 for included reviews). Within each column, bars are organised left to right by chemical class (bisphenols, phthalates, PCBs, PBDEs, PFAS) and then within each chemical class from low to high molecular weight (for phthalates and PFAS), or congener (for PCBs and PBDEs; Suppl File 1.4).

**Table 2:** Characteristics of included reviews.

REVIEW DETAILS (AUTHOR AND YEAR AND NUMBER OF META-ANALYSES/EE)	OUTCOMES REPORTED	POPULATION (DESCRIPTION)	PLASTIC-ASSOCIATED CHEMICAL(S) INVESTIGATED	SUBGROUPS BY STUDY CHARACTERISTICS	BIOSPECIMEN AND EXPOSURE TIMING	AMSTAR SCORE (/11)
**Birth outcomes (** Fig 2 **)**						
Hu et al., 2018a [46] EE = 4	Birth weight	Infants	BPA	Pregnancy stages	Urine, blood, or amniotic fluid; prenatal	8
Golestanzadeh et al., 2019 [54] EE = 10	Birth weight	Infants	MMP, MEP, MnBP, MiBP, MBzP, ΣDEHP, MEHP, MEHHP, MEOHP, MECPP		Urine; prenatal	5
*Govarts et al., 2012 [50] EE = 1	Birth weight	Infants	PCB 153		Cord plasma or serum; maternal serum or breast milk; prenatal	3
Zou et al., 2019 [53] EE = 6	Birth weight	Infants	Total PCBs	Pregnancy stages, samples analysed	Cord blood; maternal serum; prenatal	4
Negri et al., 2017 [47] EE = 26	Birth weight	Infants	PFOA, PFOS	Transformed data, pregnancy stages, samples analysed	Cord serum; maternal serum or plasma or breast milk; prenatal	8
Steenland et al., 2018 [51] EE = 5	Birth weight	Infants	PFOA	Pregnancy stages, samples analysed	Maternal or cord blood; prenatal	4
Zhong et al., 2020 [52] EE = 4	Birth length, birth weight, head circumference, gestational age	Infants	BPA		Urine; prenatal	5
Zhao et al., 2017 [45] EE = 10	Birth length, birth weight, head circumference	Infants; with subgroup of girls and boys	Total PBDEs, BDE 47, BDE 99, BDE 100, BDE 153		Serum; prenatal	9
Johnson et al., 2014 [44] EE = 4	Birth length, birth weight, head circumference, ponderal index	Infants	PFOA		Cord blood; maternal serum; prenatal	10
Nieminen et al., 2013 [49] EE = 1	Sex ratio	Infants	Total PCBs		Maternal blood or breast milk; paternal blood; cord blood; prenatal	3
Zhang et al., 2020 [48] EE = 10	Spontaneous pregnancy loss	Adult reproductive women	MMP, MEP, MnBP, MiBP, MBzP, ΣDEHP, MEHP, MEHHP, MEOHP, MECPP		Urine	7
**Child Reproductive outcomes (** Fig 3 **)**						
Bigambo et al., 2020 [55] EE = 1	Onset of puberty/early puberty	Girls	BPA		Urine; prenatal and postnatal	5
Wen et al., 2015 [57] EE = 2	Precocious puberty	Girls from 0.5 to 11.3 years of age	DnBP, DEHP	Samples analysed	Urine or serum; postnatal	7
Golestanzadeh et al., 2020 [56] EE = 27	Abnormal timing of breast development (thelarche), abnormal timing of pubic hair development (pubarche) in girls and boys, abnormal age of menarche, testicular volume in boys	Adolescent boys and girls from 7 to 19 years of age	MMP, MEP, MnBP, MEHP, MEHHP, MEOHP		Urine; prenatal and postnatal	6
Dorman et al., 2019 [58] EE = 1	Anogenital distance	Male infants	ΣDEHP		Urine; prenatal	8
Nelson et al., 2020 [59] EE = 2	Anoclitoral and anofourchette distance	Female infants	BPA		Urine, cord serum or plasma; prenatal	7
**Adult reproductive outcomes (** Fig 4 **)**						
Wen et al., 2019 [64] EE = 1	Endometriosis	Women	BPA		Urine	7
Cai et al., 2019 [60] EE = 5	Endometriosis	Women	MEP, MBzP, MEHP, MEHHP, MEOHP		Urine or plasma	7
Cano-Sancho et al., 2019 [61] EE = 5	Endometriosis	Women	Total PCBs	Samples analysed, type of endometriosis	Serum or adipose tissue	8
Roy et al., 2015 [62] EE = 1	Endometriosis	Women	Total PCBs		Serum	3
Cai et al., 2015 [63] EE = 93	Low sperm concentration, Low sperm morphology	Subfertile men	MMP, MEP, MnBP, MBzP, ΣDEHP, MEHP, MEOHP, MEHP + MEOHP (combined); with different concentration levels		Urine	6
	Low sperm motility	Subfertile men	MMP, MEP, MnBP, MBzP, DnBP, ΣDEHP, DEHP, MEHP, MEOHP; with different concentration levels		DnBP and DEHP in seminal fluid; phthalate metabolites in urine	6
	Sperm motion (straight-line velocity, curvilinear velocity, linearity), sperm DNA (comet extent, %DNA in tail, tail distributed moment)	Subfertile men	MMP, MEP, MnBP, MBzP, MEHP; with different concentration levels		Urine	6
	Low semen volume	Subfertile men; with subgroup of men in their reproductive age	MnBP		Urine	6
**Endocrine outcomes (** Fig 5 **)**						
Kim et al., 2019a [70] EE = 36	Thyroid function (free thyroxine [ft4], total thyroxine [TT4], thyrotropin [TSH])	Adults and children; with subgroups of children, adults, pregnant women	MEHP, MEHHP, MEOHP		Urine	5
Zhao et al., 2015 [72] EE = 2	Thyroid function (total thyroxine [TT4], thyrotropin [TSH])	Adults and children	Total PBDEs		Serum (ng/g lipid)	9
Kim et al., 2018 [71] EE = 66	Thyroid function (free thyroxine [ft4], total thyroxine [TT4], thyrotropin [TSH], triiodothyronine [T3])	Adults; with subgroups of pregnant and non-pregnant adults	PFOA, PFOS, PFHxS; with different concentration levels		Blood	7
Hwang et al., 2018 [65] EE = 3	Type 2 diabetes	Adults	BPA	Samples analysed	Serum or urine	6
Rancière et al., 2015[66] EE = 1	Type 2 diabetes	Adults	BPA		Urine	7
*Wu et al., 2013 [68] EE = 5	Type 2 diabetes	Adults; majority women; one included study with PCB poisoning	Total PCBs, PCB 118, PCB 138, PCB 153, PCB 180		Serum	4
Song et al., 2016 [67] EE = 18	Type 2 diabetes, insulin resistance, fasting insulin, fasting glucose, 2-hour glucose, 2-hour insulin	Adults; with subgroups of men and women	BPA, total phthalates, MEP, MiBP, total PCBs		Serum (total PCBs) or urine	6
Shoshtari-Yeganeh et al., 2019 [69] EE = 10	Insulin resistance	Adults and children	MMP, MEP, MiBP, MBzP, ΣDEHP, MEHP, MEHHP, MEOHP, MECPP, MCPP		Serum or urine	4
**Children’s neurodevelopmental outcomes (** Fig 6 **)**						
Lam et al., 2017 [74] EE = 1	Intelligence Quotient (IQ) using the Full Scale Intelligence Quotient (FSIQ) or McCarthy Scale	Children from 4 to 7 years of age	BDE-47		Cord blood or maternal serum (ng/g lipid); prenatal	11
Lee et al., 2018 [75] EE = 4	Cognitive development or Intelligence Quotient (IQ) using Wechsler Intelligence Scale for Children (WISC), Bayley Scales of Infant Development (BSID) and subscale of BSID, Mental Development Index (MDI) and Full-scale intelligence quotient (FSIQ); psychomotor development using Psychomotor Development Index (PDI)	Children from 6 months to 12 years of age	DEHP metabolites (mDEHP)		Urine or plasma; prenatal and postnatal	7
Radke et al., 2020 [76] EE = 30	Cognitive development or Intelligence Quotient (IQ) using Bayley Scales of Infant Development, Mental Development Index (MDI), Bayley III Cognitive Development Scale and fine motor using Bayley III Fine Motor Scale	Children ≤ 4 years of age	MEP, MnBP, MiBP, MBzP, ΣDEHP	Girls and boys	Urine or plasma; prenatal and postnatal	8
*Forns et al., 2020 [77] EE = 30	Attention Deficit Hyperactivity Disorder (ADHD) using Attention Syndrome Scale of the Child Behavior Checklist (CBCL-ADHD), Hyperactivity/Inattention Problems subscale of the Strengths and Difficulties Questionnaire (SDQ-Hyperactivity/Inattention) and ADHD Criteria of Diagnostic and Statistical Manual of Mental Disorders, 4th ed.	Children 4 to 11 years of age	PFOA, PFOS	Girls and boys; estimated PFAS levels from birth to 24 months	Maternal serum/plasma or breast milk; prenatal except for breast milk	3
**Nutritional outcomes (** Fig 7 **)**						
Ribeiro et al., 2019 [81] EE = 17	BMI, BMI z-score, obesity, waist circumference	Adults and children	MEP, MnBP, MiBP, MBzP, MEHP, MEHHP, MEOHP, MECPP, MCPP		Urine; postnatal	6
Rancière et al., 2015 [66] EE = 7	Obesity, overweight (or generalised overweight), elevated waist circumference	Adults and children; with subgroup of adults and children	BPA		Urine; postnatal	7
Ribeiro et al., 2020 [80] EE = 7	Obesity, overweight (or generalised overweight), elevated waist circumference	Adults and children; with subgroup of adults and children	BPA		Urine; postnatal	7
Kim et al., 2019b [78] EE = 4	Obesity	Children; with subgroups of obese vs. normal-weight children	BPA; with subgroup of high exposure		Urine; postnatal	6
Wu et al., 2020a [79] EE = 3	Abdominal obesity, generalised obesity, overweight (or generalised overweight)	Adults and children	BPA		Urine; postnatal	5
Liu et al., 2018 [82] EE = 6	Obesity or overweight, BMI	Children	PFOA	Exposure timing, girls and boys	Maternal serum or plasma; cord blood; prenatal and postnatal	7
Golestanzadeh et al., 2019 [54] EE = 22	BMI, BMI z-score, waist circumference	Children	MMP, MEP, MnBP, MiBP, MBzP, MEHP, MEHHP, MEOHP, MECPP, MnOP, MCPP		Urine; postnatal	5
**Circulatory outcomes (** Fig 8 **)**						
*Dunder et al., 2019 [84] EE = 30	Serum lipids (low-density cholesterol [LDL-C], high-density cholesterol [HDL-C], total cholesterol [TC], triglycerides [TG] and apolipoprotein B [ApoB])	Adults and children	BPA	Adults (men and women) and children (girls and boys)	Urine; postnatal	4
Golestanzadeh et al., 2019 [54] EE = 24	Systolic blood pressure, diastolic blood pressure, high-density cholesterol (HDL), triglycerides (TG)	Children	MMP, MBzP, ΣDEHP, MEHP, MEHHP, MEOHP, MCPP		Urine; postnatal	5
Park et al., 2016 [85] EE = 4	Hypertension	Adults	PCB 118, 153, dioxin-like PCBs, non-dioxin-like PCB		Serum (lipid) or adipose tissue	7
Rancière et al., 2015 [66] EE = 1	Hypertension	Adults	BPA		Urine	7
Fu et al., 2020 [87] EE = 13	Cardiovascular disease	Adults and children	BPA, MEP, MnBP, MiBP, MBzP, MEHP, MEHHP, MEOHP, MECPP, Total PCBs, PCB 138, 153, 180		Urine, serum, plasma or adipose tissue; children, postnatal	6
*Li et al., 2015 [83] EE = 7	Cardiovascular disease, cerebrovascular disease and hypertension deaths	Adults; with subgroups of men and women with cerebrovascular disease and hypertension deaths	Special PCB exposure (poisoning)		Blood	4
**Respiratory outcomes (** Fig 9 **)**						
Wu et al., 2020b [90] EE = 80	Asthma	Adults and children	MEP, MnBP, MiBP, MBzP, DEHP or ΣDEHP, MEHP, MEHHP, MEOHP, MECPP, MCOP, MCNP, MCPP	Exposure timing prenatal and postnatal, adult men and women	Urine	5
Luo et al., 2020 [89] EE = 28	Asthma, allergic rhinitis, wheeze	Children	PFOA, PFOS, PFHxS, PFNA	Exposure timing, prenatal and postnatal	Infant’s/children’s cord blood or plasma or serum; maternal serum or plasma	7
Li et al., 2017 [88] EE = 8	Asthma	Children	MnBP, MiBP, MBzP, DEHP or ΣDEHP, MCOP	Exposure timing, prenatal and postnatal	Urine	9
*Gascon et al., 2014 [91] EE = 14	Bronchitis, wheeze and bronchitis and/or wheeze	Infants/children	PCB 153	Infants < 18 months and 18 to 49 months of age, prenatal and postnatal	Maternal blood or serum or breast milk; infant’s/children’s cord, plasma or serum; prenatal	3
**Skin disorder outcomes (** Fig 10 **)**						
Luo et al., 2020 [89] EE = 8	Atopic dermatitis and eczema, with subgroups of skin disorder	Children	PFOA, PFOS, PFHxS, PFNA		Infant’s/children’s cord blood or plasma or serum; maternal serum or plasma;	7
**Cancer and cancer related mortality (** Fig 11 **)**						
Roy et al., 2015 [62] EE = 1	Breast cancer	Women	Total PCBs		Serum, plasma or adipose tissue	3
Zhang et al., 2015 [93] EE = 1	Breast cancer	Women	Total PCBs	Serum and adipose sample only	Serum, plasma or adipose tissue	8
Leng et al., 2016 [94] EE = 17	Breast cancer	Women	PCB 187, 118, 138, 156, 170, 99, 153, 180, 183. Including analyses of two studies for only PCB 28, 52, 74, 77, 101, 105, 126, 167		Serum, plasma or adipose tissue	8
Zani et al., 2013 [92] EE = 6	Breast cancer	Women	Total PCBs		Serum, plasma or adipose tissue	2
	Non-Hodgkin’s lymphoma	Adults and children	Total PCBs, PCB 118, PCB 138, PCB 153, PCB 180		Blood, serum or adipose tissue	2
Catalani et al., 2019 [96] EE = 8	Non-Hodgkin’s lymphoma (NHL), subtypes of NHL (chronic lymphocytic leukaemia, diffuse large B-cell lymphoma, follicular lymphoma	Adults and children	Total PCBs, PCB 118, PCB 138, PCB 153, PCB 180, PCB 170,		Blood, serum or adipose tissue	7
Zani et al., 2017 [95] EE =3	Non-Hodgkin’s lymphoma	Adults and children	Total PCBs		Blood, serum or adipose tissue	5
	Non-Hodgkin’s lymphoma mortality, melanoma mortality	Adults	Special PCB exposure (occupational)		Blood, serum or adipose tissue	5
*Li et al., 2015 [15] EE = 12	All-cancer mortality and cancer-specific mortality (breast cancer, leukaemia, liver cancer, lung cancer, pancreatic cancer, rectal cancer, stomach cancer, uterine cancer)	Adults; with subgroups of men and women in some cancer types	Special PCB exposure (poisoning)		Blood	4
**Other outcomes (** Fig 12 **)**						
*Li et al., 2015 [83] EE = 6	Hepatic disease mortality, all-cause mortality	Adults; with subgroups of men and women	Special PCB exposure (poisoning)		Blood	4
**Case control studies (**Supplementary Fig S1**)**						
Wen et al., 2015 [57] EE = 7	Precocious puberty	Girls from 0.5 to 11.3 years of age	MEP, DnBP, MnBP, MBzP, DEHP, MEHP	Samples analysed	Urine or serum; postnatal	7
Hu et al., 2018b [73] EE = 3	Polycystic ovarian syndrome (PCOS)	Women; with subgroups with different age, method of measurement	BPA	Serum samples, age	Serum	9

*Legend:*

*pooled analysis.

*EE: number of effect estimates (from main and subgroup analyses) included from the systematic review or pooled analysis.*

*Superscript number indicates the reference number in the harvest plot figures.*

*Total phthalates: composite measure of phthalate metabolite exposure which is the total concentration of all phthalate metabolites measured.*

*ΣDEHP: sum of the DEHP metabolites (MEHP, MEHHP, MEOHP, MECPP, MCMHP).*

*Total PCBs: composite measure of PCB exposure which is the total concentration of all PCB congeners measured Total PBDEs: composite measure of PBDE exposure which is the total concentration of all PBDE congeners measured.*

*Bisphenol A (BPA), Di-n-butyl phthalate (DnBP), Di(2-ethylhexyl) phthalate (DEHP), Monomethyl phthalate (MMP), Monoethyl phthalate (MEP), Mono-n-butyl phthalate (MnBP), Monoisobutyl phthalate (MiBP), Monobenzyl phthalate (MBzP), Mono(2-ethylhexyl) phthalate (MEHP), Mono(2-ethyl-5-hydroxyhexyl) phthalate (MEHHP), Mono(2-ethyl-5-oxohexyl) phthalate (MEOHP), Mono(2-ethyl-5-carboxypentyl) phthalate (MECPP), Mono(2-carboxymethyl-5-hexyl) phthalate (MCMHP), Mono-n-octyl phthalate (MnOP), Mono(carboxyoctyl) phthalate (MCOP), Mono(carboxynonyl) phthalate (MCNP), Mono(3-carboxypropyl) phthalate (MCPP), Polychlorinated biphenyls (PCBs), Polybrominated diphenyl ethers (PBDEs) Perfluorooctanoic acid (PFOA), Perfluorooctanesulfonic acid (PFOS), Perfluorohexane sulfonate (PFHxS).*

## Results

### Review identification, selection and inclusion

**Figure 1:**
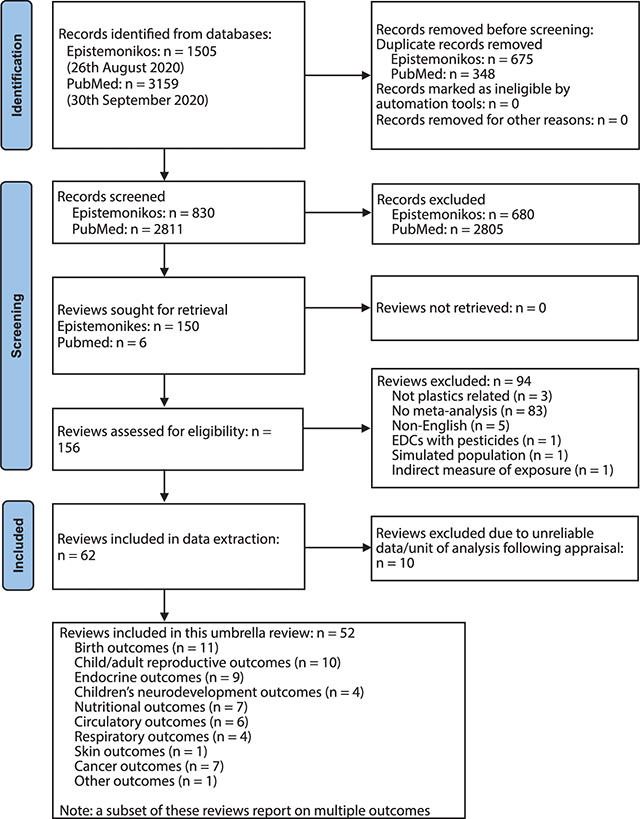
PRISMA flow diagram [35] presenting process of study identification, selection and final inclusion in the review project and the outcomes reported in this manuscript.

Database searching returned 3,641 unique records which were screened for eligibility, after electronic deduplication (Figure 1). Searching of PubMed offered only those reviews most recently published, not yet indexed in Epistemonikos. Following screening, 156 potentially eligible reviews were retrieved and the full text assessed. Sixty-two systematic reviews with meta-analyses, meta-analyses, and pooled analyses, were deemed eligible for inclusion. The predominant reason for exclusion of the remaining 94 reviews was lack of statistical meta-analysis and presentation of narrative synthesis only (Figure 1, Suppl File 1.5.1). During the conduct of this umbrella review, a further ten reviews were excluded where reporting of the EE was identified to have used data from the same studies (participants) repeatedly. This was most common for different plastic-associated chemical exposures (e.g., phthalate metabolites and PCB congeners) measured in the same participants, or where there were repeated measures over time from the same cohort, thereby introducing a unit of analysis error [43] (Figure 1, Suppl File 1.5.2). Ultimately, 52 systematic reviews with meta-analyses, meta-analyses, and pooled analyses were included (Figure 1).

There were no systematic reviews with meta-analyses addressing the health effects of plastic polymers, nor microplastics. We found meta-analysed data for only a very small number of plastic-associated chemicals: BPA, but no other bisphenols; certain ortho-phthalate diesters but no other plasticisers such as terephthalates, cyclohexanoates, adipates, trimellitates or benzoates; PCBs and PBDEs but no other flame retardants such as organophosphate esters; and only a small number of PFAS. Fifty-two eligible reviews and pooled analyses (46 reviews, 6 pooled analyses) reported on the following outcome categories: birth, child and adult reproductive, endocrine, child neurodevelopment, nutritional, circulatory, respiratory, skin-related, cancer and cancer-related mortality, hepatic disease mortality and all-cause mortality.

### Review characteristics

Characteristics of included reviews are presented in Table 2 and further details including all outcome data extracted are available in Suppl File 2. A total of 759 meta-analyses, including main analyses and subgroup analyses, were identified. Participants included infants, children and adults, including pregnant mothers, and were mostly general population samples, but also including highly exposed populations in some cases of PCB exposure. Plastic-associated chemicals included bisphenol A (BPA) for bisphenols, diester phthalates and monoester metabolites for plasticisers (e.g., DEHP, di-n-butyl phthalate [DnBP], and metabolites: monomethyl phthalate [MMP], monoethyl phthalate [MEP], mono(2-ethylhexyl) phthalate [MEHP], monobenzyl phthalate [MBzP]), PCBs and PBDEs for flame retardants, and PFAS (perfluorooctanoic acid [PFOA], perfluorooctane sulfonate [PFOS], perfluorohexane sulfonate [PFHxS], perfluorononanoic acid [PFNA]; Table 2).

### Summary of the evidence

#### Birth outcomes

**Figure 2:**
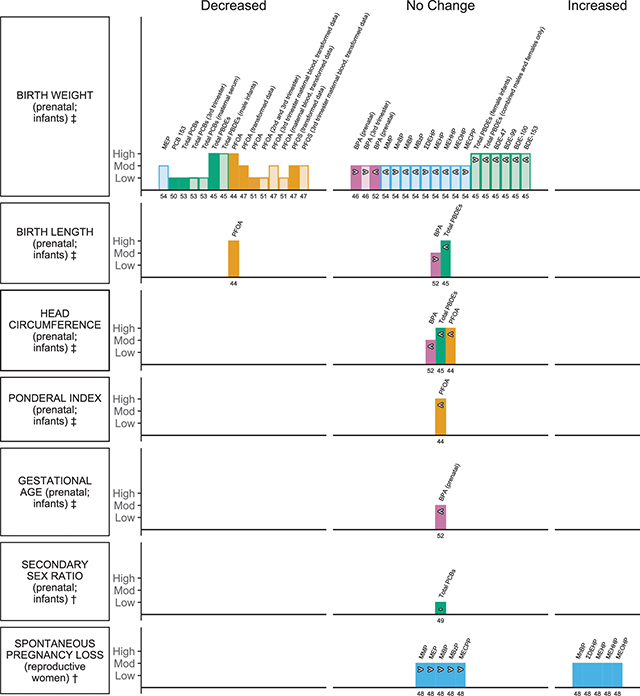
Harvest plot of exposure to plastic-associated chemicals and birth outcomes. Plastic-associated chemicals included are bisphenol A (BPA) (pink); phthalate monoester metabolites (blue), encompassing monomethyl phthalate (MMP), monoethyl phthalate (MEP), mono-n-butyl phthalate (MnBP), monoisobutyl phthalate (MiBP), monobenzyl phthalate (MBzP), mono(2-ethylhexyl) phthalate (MEHP), mono(2-ethyl-5-hydroxyhexyl) phthalate (MEHHP), mono(2-ethyl-5-oxohexyl) phthalate (MEOHP), mono(2-ethyl-5-carboxypentyl) phthalate (MECPP), and molar sum of the di(2-ethylhexyl) phthalate metabolites (∑DEHP); flame retardants (green) encompassing polychlorinated biphenyl (PCB), polybrominated diphenyl ethers (PBDEs), 2,2’,4,4’-tetrabromodiphenyl ether (BDE-47), 2,2’,4,4’,5-pentabromodiphenyl ether (BDE-99), 2,2’,4,4’,6-pentabromodiphenyl ether (BDE-100), 2,2’,4,4’,5,5’-hexabromodiphenyl ether (BDE-153); and per- and polyfluoroalkyl substances (PFAS) (orange), encompassing perfluorooctanoic acid (PFOA) and perfluorooctane sulfonate (PFOS). Outcomes are either dichotomous (†) or measured on a continuous scale (‡). Outcome measures include ‡birth weight, ‡birth length, ‡head circumference, ‡ponderal index, ‡gestational age, †secondary sex ratio and †spontaneous pregnancy loss. Each bar represents an individual effect estimate from the corresponding review, which is indicated by the number below each bar. The height of the bar represents the quality score of the review assessed using the AMSTAR tool. Low quality reflects a score of 1–4, moderate quality a score of 5–8 and high quality a score of 9–11. Dark filled bars represent the main analyses of each review; light filled bars represent sub-group analyses. Bars have been assigned as an increase or decrease (columns) in the measure where the change is statistically significant. Remaining bars appearing under ‘no change’ indicate direction of effect as an increase (>), no clear trend (–) (the estimate of relative risk was 1 or regression coefficient or mean difference was 0), or decrease (<) in the measure or risk estimate.

There were seven birth outcomes reported across ten systematic reviews with meta-analyses and one pooled analysis. Of these, evidence from available analyses suggests an association with a decrease in infant birth weight, and an increase in spontaneous pregnancy loss (SPL; i.e., miscarriage) by mothers across the plastic-associated chemical exposures that have been evaluated (Figure 2). Birth outcomes were addressed for BPA, phthalates, flame retardants and PFAS. Anthropometric measures including birth weight were the most commonly reported in eight reviews and one pooled analysis, followed by birth length and head circumference in three reviews. Other child outcomes, including ponderal index, gestational age, sex ratio and SPL, were each reported in one review. Where outcomes were measured in infants, exposure to plastic-associated chemicals was prenatal and details of type of samples measured are provided in Table 2.

The reviews that informed this outcome category ranged from low to high quality, scoring between 3 and 10 on the AMSTAR tool (Table 2; Figure 2; Suppl File 1.6). Only two reviews were informed by an a priori protocol and included searching for grey literature [44, 45]; duplicate selection and extraction could be confirmed for only five reviews [44–48]. Transparent reporting of included and excluded studies was provided by only two reviews [46,49], whereas all reviews provided detailed study characteristics and assessment of publication bias. Half of the included reviews provided no assessment of the quality of the included studies [49–53] and even fewer reviews considered quality further in their analyses [45, 46, 54]. One review investigating phthalates had problematic main analyses, as findings from the same sample of the population were used repeatedly within sub-analyses for each metabolite [54]. Overall, reviews of highest methodological quality informed flame retardant (PDBE) and PFAS (PFOA) exposure (Table 2; Figure 2; Suppl File 1.6).

##### Birth weight

All of the plastic-associated chemical classes included in this umbrella review were considered for this outcome. Fifty-two meta-analyses, including both main analyses and subgroup analyses, informed the association between plastic-associated chemical exposure and change in birth weight. The majority of effect estimates informing PFAS (10/13 EE) and flame retardants (PCBs and PDBEs; 9/15 EE) suggested a decrease in birth weight with exposure. One phthalate plasticiser (1/10 EE) was associated with a decrease in birth weight, and BPA exposure was not significantly associated with any change (5/5 EE) (Figure 2).

Two main analyses showed no significant association with a change in birth weight with exposure to BPA, ES 4.42g, 95%CI –8.83 to 17.67 (highest vs lowest exposure) [46] and β –0.049g, 95%CI –0.199 to 0.101 (untransformed) [52] respectively (Figure 2). Similarly, no association with a change in birth weight was observed irrespective of which trimester exposure was analysed (3/3 EE; Figure 2; first and second trimester not plotted; Suppl File 2.1) [46].

Ten meta-analyses from one review assessed the association of birthweight with prenatal phthalate metabolites (Figure 2) [54]. Results for the main analysis for this review were excluded due to unit of analysis error (see Section 3.3.1). A significant decrease in birth weight was observed for higher MEP, z –10.1g, 95%CI –18.57 to –1.6, with no significant change in estimates of association for all the remaining metabolites investigated, including ∑DEHP, though the majority tended towards a decrease (6/9 EE; Figure 2; Suppl File 2.1) [54].

One meta-analysis reported a significant association between higher exposure to PCBs (total) and reduced birth weight of β –0.59g, 95%CI –0.852 to –0.343 (untransformed). This association was consistent with measurement of exposure also in maternal serum, cord serum and across all trimesters of pregnancy (5/5 EE; Figure 2; cord serum, first and second trimester not plotted; Suppl File 2.1) [53]. Similarly, a significant association of β –0.15, 95%CI –0.24 to –0.05, was reported in a pooled analysis investigating the single congener, PCB 153 (Figure 2) [50]. Considering PDBEs, the association with reduced birth weight was statistically significant for the composite measure of exposure, β –50.56g, 95%CI –95.91 to –5.28, and for the subgroup analysis that included just male infants. Where studies included male and female infants, the reduction in birth weight was no longer significant and likely tempered by the observation that birth weight trended towards an increase when only female infants were analysed (Figure 2; Suppl File 2.1) [45]. Analyses of the individual congeners BDE-47, −99, −100 and −153 were not significantly associated with a change in birth weight, although there was a trend towards decreased birth weight for each congener (4/4 EE; Figure 2; Suppl File 2.1) [45].

Of the main analyses that investigated PFOA exposure in infants, all reported a statistically significant decrease in birth weight, with a range of β from –10.5 to –18.9g (Figure 2; Suppl File 2.1) [44, 47, 51]. The significant association was also observed in subgroup analyses where measure of exposure was determined from cord serum (1/3 EE; data not plotted; Suppl File 2.1) [47, 51] and maternal blood during the second (3/4 EE) and third trimester (2/2 EE) of pregnancy (Figure 2; Suppl File 2.1) [47, 51]. No changes were observed with exposure measured in the first trimester (2/2 EE; data not plotted; Suppl File 2.1) [47, 51]. Similarly, whilst exposure to PFOS was significantly associated with a decrease in birth weight of β –46.09g, 95%CI –80.33 to –11.85 in infants and when exposure was measured in mothers (also in cord serum; 1/2 EE; transformed; data not plotted; Suppl File 2.1) during the third trimester of pregnancy (1/2 EE) [47], no significant changes were observed with measures of exposure during the first two trimesters (2/2 EE; data not plotted; Suppl File 2.1) [47].

##### Birth length, head circumference and ponderal index

Seven meta-analyses addressed the remaining anthropometric measures pertinent to birth outcomes; three informed the association of plastic-associated chemical exposure with birth length and three meta-analyses from the same reviews informed the association with head circumference, while one analysis assessed ponderal index. Higher prenatal exposure to PFOA was associated with a significant decrease in birth length of β –0.06 cm, 95%CI –0.09 to –0.02, and non-significant decreases were observed for the majority of remaining outcome estimates (5/6 EE, Table 2; Figure 2) [44]. The remaining analyses reported no significant association of birth length with prenatal BPA exposure, β 0.058cm, 95%CI –0.072 to 0.188, nor head circumference, β –0.004cm, 95%CI –0.119 to 0.111 (Figure 2) [52]. Similarly, prenatal exposure to composite measures of PBDEs resulted in no significant decrease in birth length, β –0.33 cm, 95%CI –0.74 to 0.07 nor head circumference, β –0.175 cm, 95%CI –0.42 to 0.07, respectively (Figure 2) [45] and no significant change in head circumference, β –0.03cm, 95%CI –0.08 to 0.01 with PFOA exposure (Figure 2) [44]. No change was reported in ponderal index of infants with higher exposure to PFOA β –0.01 95%CI –0.08 to 0.01 (Figure 2) [44].

##### Gestational age and sex ratio

No changes were observed in two meta-analyses investigating the association with gestational age and BPA exposure, β –0.032 weeks, 95%CI –0.163 to 0.10 [52], nor secondary sex ratio 0.5, 95%CI 0.45 to 0.551 with higher exposure to PCBs (2/2 EE, Figure 2) [49].

##### Spontaneous pregnancy loss (SPL)

Ten meta-analyses for individual phthalate metabolites from one review reported the association of exposure to phthalate plasticisers in pregnant women and SPL (Figure 2) [48]. A significant increase in risk of SPL was observed for higher concentrations of mono-n-butyl phthalate (MnBP) and DEHP metabolites MEHP, mono(2-ethyl-5-hydroxyhexyl) phthalate (MEHHP) and mono(2-ethyl-5-oxohexyl) phthalate (MEOHP), as well as ∑DEHP, with a range in risk estimates from OR 1.34 to 1.79 (5/10 EE; Figure 2; Suppl File 2.1) [48]. The phthalate metabolites MMP, MEP, monoisobutyl phthalate (MiBP), MBzP and mono(2-ethyl-5-carboxypentyl) phthalate (MECPP) were not significantly associated with any change in risk of SPL, though all tended towards an increase (5/10 EE; Figure 2; Suppl File 2.1) [48].

### Child reproductive health outcomes

**Figure 3:**
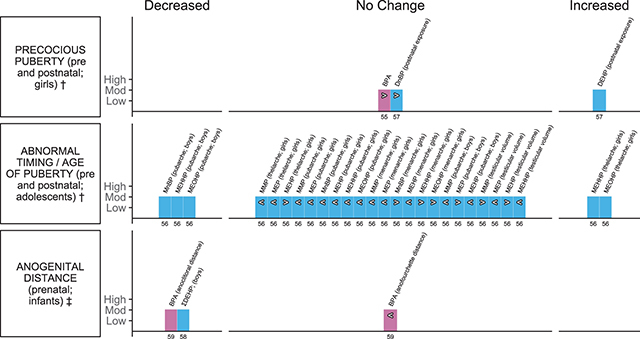
Harvest plot of exposure to plastic-associated chemicals and child reproductive outcome measures. Plastic-associated chemicals included are bisphenol A (BPA) (pink); and phthalate diesters diethylhexyl phthalate (DEHP) and di-n-butyl phthalate (DnBP) and monoester metabolites (blue), including monomethyl phthalate (MMP), monoethyl phthalate (MEP), mono-n-butyl phthalate (MnBP), monobenzyl phthalate (MBzP), mono(2-ethylhexyl) phthalate (MEHP), mono(2-ethyl-5-hydroxyhexyl) phthalate (MEHHP), mono(2-ethyl-5-oxohexyl) phthalate (MEOHP), mono(2-ethyl-5-carboxypentyl) phthalate (MECPP), and mono (3-carboxypropyl) phthalate (MCPP). Outcomes are either dichotomous (†) or measured on a continuous scale (‡). Outcomes measured include †precocious puberty, ‡anogenital distance measured by anoclitoral and anofourchette distance in girls and anoscrotal and anopenile distance in boys, †abnormal timing/age of puberty/early puberty measured by pubarche, menarche, thelarche and testicular volume. Each bar represents an individual effect estimate from the corresponding review, which is indicated by the number below each bar. The height of the bar represents the quality score of the review assessed using the AMSTAR tool. Moderate quality reflects a score of 5–8. Dark filled bars represent the main analyses of each review. Bars have been assigned as an increase or decrease (columns) in the measure where the change is statistically significant. Remaining bars appearing under ‘no change’ indicate direction of effect as an increase (>), or decrease (<) in the measure or risk estimate.

There were eight child reproductive health outcome measures evaluated across five systematic reviews with meta-analyses. Of these, the evidence suggests an association with changes in markers of the timing of puberty and adolescent development, and decreases in anogenital distance (AGD), in children with exposure to BPA and some phthalate plasticisers (Figure 3). Outcomes indicative of timing of puberty and adolescent development following prenatal and postnatal plastic-associated chemical exposure, including measures of abnormal timing of puberty- thelarche (breast development), menarche (first menstrual cycle) and pubarche (development of pubic hair; girls and boys) and precocious puberty (appearance of secondary sex characteristics before eight years of age) -were reported in three reviews (Table 2) [55–57]. Markers of AGD, including anoclitoral and anofourchette distance in girls and anoscrotal and anopenile distance in boys, were reported in two reviews following prenatal exposure (Table 2) [58, 59].

The reviews that informed this outcome category were all rated as moderate quality, scoring 5–8 on the AMSTAR tool (Table 2; Figure 3; Suppl File 1.6). Only one review was informed a priori [58] or included searching for grey literature [57]; duplicate selection and extraction could be confirmed for only two reviews [58, 59]. No reviews provided transparent records of included and excluded studies, whereas all reviews provided detailed study characteristics and details of assessment of quality of included studies (Table 2; Figure 3; Suppl File 1.6).

#### Onset of puberty

Thirty meta-analyses from three reviews informed the association between both pre- and postnatal plastic-associated chemical exposure and measures indicative of pubertal timing in girls and boys [55–57]. Measures included abnormal (early or delayed) timing of thelarche, abnormal age of pubarche and abnormal age of menarche, and a selection of these same measures was also used to report precocious puberty in girls. Measures in boys included abnormal timing of pubarche and testicular volume.

Two reviews investigated pre- and postnatal exposure and puberty outcomes in girls [55, 57] and one in adolescents [56] (Table 2). Onset of puberty before 8 or after 13 years of age was considered as abnormal timing across the measures considered. BPA exposure was not associated with the risk of precocious puberty in girls, ES 1.09, 95%CI 0.88 to 1.35 (Figure 3) [55]. Higher serum DEHP was significantly associated with an increased risk in precocious puberty in girls, OR 4.09, 95%CI 2.3 to 7.3; however, the increase was not statistically significant with exposure to DnBP, OR 3.26, 95%CI 0.69 to 15.42 (Figure 3) [57]. Seventeen meta-analyses addressed various measures indicative of onset of puberty with six phthalate metabolites in girls. An increased risk of abnormal timing of thelarche was observed with higher concentrations of the DEHP metabolites MEHHP, OR 1.48, 95%CI 1.11 to 1.85, and MEOHP, OR 1.52, 95%CI 1.15 to 1.88 (Figure 3) [56]. The majority of the remaining analyses suggested decreases with phthalate metabolites for age of thelarche (2/3 EE), menarche (3/6 EE) and pubarche (6/6 EE) though no changes were statistically significant (Figure 3; Suppl File 2.2) [56]. In boys, a decreased risk of abnormal age of pubarche (premature or delayed) with higher phthalate metabolites was observed for MnBP, OR 0.66, 95%CI 0.39 to 0.93, MEHHP, OR 0.61, 95%CI 0.32 to 0.91, and MEOHP, OR 0.61, 95%CI 0.26 to 0.97, while for the remaining metabolites meta-analysed (MMP, MEP, MEHP), no association was observed (2/3 EE decreased; Figure 3; Suppl File 2.2) [56]. Similarly, for testicular volume, no association was reported with any of the phthalate metabolites analysed (2/4 EE decreased; Figure 2; Suppl File 2.2) [56].

One review of case control studies also reported seven meta-analyses of the differences in phthalate metabolites detected in serum or urine between girls with precocious puberty and those without (Table 2; Suppl File 2.2) [57]. The serum concentration of DEHP, SMD 1.73, 95%CI 0.54 to 2.91, and DnBP, SMD 4.31, 95%CI 2.67 to 5.95, was greater in girls with precocious puberty than those without (Suppl Figure S1) [57]. No association was observed for the remaining metabolites (5/5 EE), three of which indicated an increased (non-significant) phthalate concentration in girls with precocious puberty (3/5 EE) assessed (Suppl File 2.2) [57].

#### Anogenital distance (AGD)

Three meta-analyses from two reviews informed the association between plastic-associated chemical exposure and measures of AGD in both female and male infants [58, 59]. Of the two analyses that investigated BPA exposure and AGD in female infants (Table 2), one reported a statistically significant decrease in anoclitoral distance, β −1.37, 95%CI −2.48 to −0.27, whereas the decrease in anofourchette distance was non-significant, β −1.07, 95%CI −3.65 to 1.51 (standardised % change per log_10_ change in BPA; Figure 3) [59]. One meta-analysis reported a statistically significant decrease in AGD (predominantly anoscrotal distance) in male infants with phthalate plasticiser exposure in utero, β −4.07, 95%CI −6.49 to −1.66 (standardised % change per log_10_ change in ∑DEHP or MEHP; Figure 3) [58].

### Adult reproductive health outcomes

**Figure 4:**
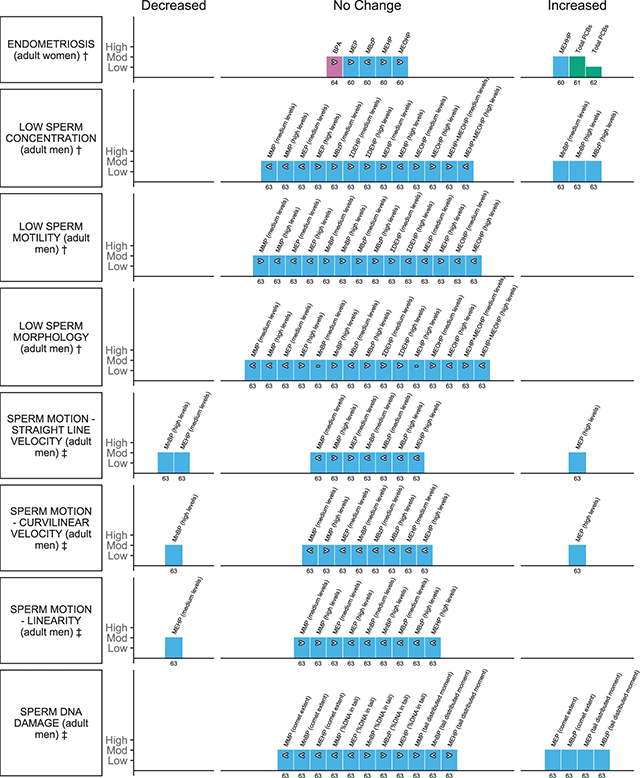
Harvest plot of exposure to plastic-associated chemicals and adult reproductive outcome measures. Plastic-associated chemicals included are bisphenol A (BPA) (pink); phthalate monoester metabolites (blue), including monomethyl phthalate (MMP), monoethyl phthalate (MEP), mono-n-butyl phthalate (MnBP), monobenzyl phthalate (MBzP), mono(2-ethylhexyl) phthalate (MEHP), mono(2-ethyl-5-hydroxyhexyl) phthalate (MEHHP), mono(2-ethyl-5-oxohexyl) phthalate (MEOHP), mono(2-ethyl-5-carboxypentyl) phthalate (MECPP), molar sum of the di(2-ethylhexyl) phthalate metabolites (∑DEHP), and mono (3-carboxypropyl) phthalate (MCPP); and flame retardants (green) encompassing polychlorinated biphenyl (PCB). Outcomes are either dichotomous (†) or measured on a continuous scale (‡). Outcomes measured include †endometriosis, †sperm concentration, ‡†sperm motility, †sperm morphology, †sperm volume, ‡sperm motion measured via straight line velocity, curvilinear velocity, linearity, and ‡sperm DNA damage measured via comet assay (comet extent), comet assay (percentage [%] DNA in tail) and comet assay (tail distributed moment). Each bar represents an individual effect estimate from the corresponding review, which is indicated by the number below each bar. The height of the bar represents the quality score of the review assessed using the AMSTAR tool. Low quality reflects a score of 1–4 and moderate quality a score of 5–8. Dark filled bars represent the main analyses of each review; light filled bars represent sub-group analyses. Bars have been assigned as an increase or decrease (columns) in the measure where the change is statistically significant. Remaining bars appearing under ‘no change’ indicate direction of effect as an increase (>), no clear trend (–) (the estimate of relative risk was 1 or regression coefficient or mean difference was 0), or decrease (<) in the measure or risk estimate.

Ten adult reproductive health outcome measures were reported in five systematic reviews with meta-analyses. Of these, the evidence available suggests an association with an increased risk of endometriosis in women, and reduction in sperm concentration and changes to motility, motion and increased sperm DNA damage in men with exposure to plastic-associated chemicals (Figure 4). Risk of endometriosis was the most commonly reported outcome addressed for BPA, phthalates and flame retardants in three reviews (Table 2) [60–62], while multiple measures of semen quality, semen motion and sperm DNA damage with phthalate metabolites were addressed in one review (Table 2) [63].

The majority of reviews that informed this outcome category were of moderate quality, scoring between 6 and 8 on the AMSTAR tool; one review was rated as low quality, scoring 3 (Table 2; Figure 4; Suppl File 1.6) [62]. Only one review was informed a priori [61], whereas the review by Wen et al. [64] had the most complete conduct and reporting of searching to identify studies. No reviews provided transparent recordings of included and excluded studies, whereas all reviews provided detailed study characteristics and details of assessment of quality of included studies as well as appropriate statistical analyses (Table 2; Figure 4; Suppl File 1.6). All reported outcomes for this outcome domain, except risk of endometriosis, were derived from one moderate quality review (Figure 4) [63].

#### Endometriosis

Twelve meta-analyses, including both main and subgroup analyses, from four reviews informed the association between plastic-associated chemical exposure and risk of endometriosis. Exposure to BPA was not significantly associated with an increase in endometriosis, OR 1.4, 95%CI 0.94 to 2.08 (Figure 4) [64]. A statistically significant increase in risk of endometriosis with higher exposure to PCBs was reported in two main analyses with a range of risk estimates between OR 1.70 and 1.91 (Figure 4; highest versus lowest exposure categories; Suppl File 2.3) [61, 62]. Subgroup analyses revealed significant increased association with deep endometriosis, endometriosis without peritoneal form (total), and serum samples; however, not those from adipose tissue (3/4 EE; data not plotted—Suppl File 2.3) [61]. Five meta-analyses from one review [60] assessed the association with phthalates and endometriosis in women (Figure 4). A significant association for endometriosis was observed for higher concentrations of MEHHP, OR 1.25, 95%CI 1.003 to 1.549, but no significant change in estimates of association for all of the remaining metabolites investigated (3/4 EE), with all (MEP, MEHP, MEOHP) except MBzP, tending towards an increase in risk (Figure 4; Suppl File 2.3) [60].

#### Semen quality

One review reported 93 meta-analyses pertinent to sperm production, sperm quality and sperm DNA damage with urinary phthalate metabolites (Suppl File 2.3; medium and high phthalate exposure categories) [63]. Measures included sperm concentration, motility (additionally reported for seminal DEHP and DnBP) and morphology, as well as semen motion parameters (straight-line velocity [VSL], curvilinear velocity [VCL] and linearity [LIN]) and indicators of sperm DNA damage (comet assay parameters—comet extent [CE], percent of DNA in tail [Tail%] and tail distributed moment [TDM]). Risk of low sperm concentration, motility and morphology was determined compared to predefined reference values in men (Suppl File 2.3) [63].

Sixteen meta-analyses assessed the association between phthalate metabolite levels in urine and low sperm concentration. Two metabolites, MnBP (medium and high levels, OR 2.39, 95%CI 1.26 to 4.53) and MBzP (high levels only, OR 2.23, 95%CI 1.16 to 4.3), were associated with an increased risk of reduced sperm concentration (3/16 EE; Figure 4), while eight of the remaining analyses tended towards an increase in risk (7/16 EE; Figure 4). There was inconsistency in the direction of effect for many of the metabolites, dependent on the level of exposure (medium vs. high; Figure 4; Suppl File 2.3). Considering the other classical semen parameters that were assessed for urinary phthalates, no significant association with low sperm motility or decreased morphology was observed for any of the metabolites investigated across 29 meta-analyses assessing varying levels of exposure (29/29 EE; Figure 4; Suppl File 2.3). Seven analyses (7/14 EE; Figure 4) tended towards an increased risk of low sperm motility and seven towards an increasing risk of low sperm morphology (7/15 EE; Figure 4). MnBP concentrations in the highest category were not associated with low semen volume (trend decrease; Suppl File 2.3; data not plotted). Conversely, both seminal DEHP, β –0.21, 95%CI –0.3 to –0.12 and DnBP, β –0.19, 95%CI –0.28 to –0.1 levels were significantly associated with low sperm motility (2/2 EE; data not plotted; Suppl File 2.3).

Thirty meta-analyses assessed the association between five urinary phthalate metabolites (MBP, MBzP, MMP, MEP and MEHP; medium and high levels) and the sperm motion parameters VSL, VCL and LIN (Figure 4; Suppl File 2.3) [63]. MnBP (high levels) was associated with decreased VSL, β –2.51 95%CI –4.44 to –0.59, and VCL, β –3.81 95%CI –6.74 to –0.87, while MEHP (medium levels) was similarly associated with decreased VSL, β –1.06 95%CI –1.99 to –0.12 (Figure 4). All remaining analyses suggested a tendency for VSL and VCL to decrease (12/20 EE) with phthalate metabolites, except for VSL and VCL with MMP (high levels) and VSL for MEP (medium levels; Figure 4, Suppl 2.3) [63]. Conversely, urinary MEP (high levels) was significantly associated with an increased VSL, β 2.36, 95%CI 0.28 to 4.45, and VCL, β 5.23, 95%CI 1.67 to 8.80, and a non-significant decrease in LIN (Figure 4). Of the remaining analyses, the majority (6/10 EE) tended towards a decrease in LIN (Figure 4).

Comet assay parameters indicative of sperm DNA damage, including, CE, Tail%, and TDM were each analysed for the five urinary phthalate metabolites (MBP, MBzP, MMP, MEP and MEHP; medium and high levels; 15 EE; Figure 4). An interquartile range increase in MEP (449.4 ug/L), β 4.22, 95%CI 1.66 to 6.77, and MBzP (11.35 ug/L), β 3.57, 95%CI 0.89 to 6.25, was associated with an increase in CE and also TDM, MEP β 1.64, 95%CI 0.24 to 3.03, MBzP β 1.72, 95%CI 0.33 to 3.12 (Figure 4; Suppl File 2.3) [63]. No significant associations were observed for the remaining metabolites, which tended to decrease for CE (3/3 EE); however, the majority tended to increase for Tail% (3/5 EE) and TDM (2/3 EE; Figure 4) [63].

### Endocrine outcomes

**Figure 5:**
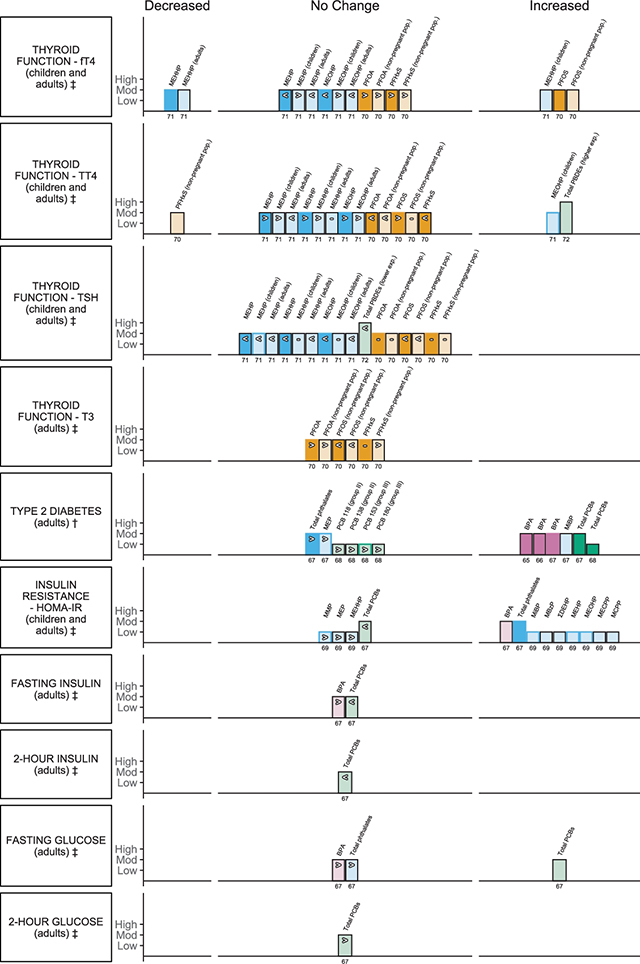
Harvest plot of exposure to plastic-associated chemicals and endocrine outcome measures. Plastic-associated chemicals included are bisphenol A (BPA) (pink); phthalate monoester metabolites (blue), encompassing monomethyl phthalate (MMP), monoethyl phthalate (MEP), monoisobutyl phthalate (MiBP), monobenzyl phthalate (MBzP), mono(2-ethylhexyl) phthalate (MEHP), mono(2-ethyl-5-hydroxyhexyl) phthalate (MEHHP), mono(2-ethyl-5-oxohexyl) phthalate (MEOHP), mono(2-ethyl-5-carboxypentyl) phthalate (MECPP), mono(3-carboxypropyl) phthalate (MCPP) and molar sum of the di(2-ethylhexyl) phthalate metabolites (∑DEHP); flame retardants (green) encompassing polychlorinated biphenyl (PCB), 2,3’,4,4’,5-pentachlorobiphenyl (PCB 118) group (gp) II, 2,2’,3,4,4’,5’-hexachlorobiphenyl (PCB 138) (gp II), 2,2’,4,4’,5,5’-hexachlorobiphenyl (PCB 153) (gp III), 2,2’,3,4,4’,5,5’-heptachlorobiphenyl PCB 180) (gp III), polybrominated diphenyl ethers (PBDEs); and per- and polyfluoroalkyl substances (PFAS) (orange), encompassing perfluorohexane sulfonate (PFHxS), perfluorooctanoic acid (PFOA), and perfluorooctane sulfonate (PFOS). Outcomes are either dichotomous (†) or measured on a continuous scale (‡). Outcomes measured include thyroid function measured by levels of ‡free thyroxine (fT4), ‡thyroxine (TT4), ‡thyroid-stimulating hormone (TSH), and ‡triiodothyronine (T3), †type 2 diabetes (T2D), ‡insulin resistance (HOMA-IR), ‡fasting insulin, ‡2-hour (hr) insulin, ‡fasting glucose and ‡2-hour glucose. Each bar represents an individual effect estimate from the corresponding review, which is indicated by the number below each bar. The height of the bar represents the quality score of the review assessed using the AMSTAR tool. Low quality reflects a score of 1–4, moderate quality a score of 5–8 and high quality a score of 9–11. Dark filled bars represent the main analyses of each review; light filled bars represent sub-group analyses. Bars have been assigned as an increase or decrease (columns) in the measure where the change is statistically significant. Remaining bars appearing under ‘no change’ indicate direction of effect as an increase (>), no clear trend (–) (the estimate of relative risk was 1 or regression coefficient or mean difference was 0), or decrease (<) in the measure or risk estimate.

Ten endocrine outcome measures were reported in eight systematic reviews with meta-analyses and one pooled analysis. Evidence suggests an association with changes in measures of thyroid function, an increasing risk of type 2 diabetes (T2D) and other measures of blood glucose regulation, including insulin resistance using the Homeostatic Model Assessment for Insulin Resistance (HOMA-IR) and fasting glucose, as well as polycystic ovary syndrome (PCOS) in women across the plastic-associated chemical exposures that have been evaluated (Figure 5; Suppl Figure S1). Endocrine outcomes were addressed for BPA, phthalates, flame retardants and PFAS. Risk of T2D was the most commonly reported endocrine outcome in three reviews and one pooled analysis [65–68], followed by HOMA-IR in two reviews [67, 69], while the remaining measures indicative of insulin regulation in the body, including fasting insulin and glucose, as well as 2-hr insulin and 2-hr glucose were reported in one review (Table 2) [67]. Measures of thyroid function were reported in three reviews, with thyroid stimulating hormone (TSH) and total thyroxine (TT4) reported in three reviews [70–72], free thyroxine (fT4) in two reviews [70, 71] and triiodothyronine (T3) in one review (Table 2) [71]. Additionally, one review reported on PCOS (Table 2) [73].

The reviews that informed this outcome category ranged from low to high methodological quality, scoring between 4 and 9 on the AMSTAR tool (Table 2; Figure 5; Suppl File 1.6). Overall, thyroid function was informed by higher-quality reviews than those informing diabetes and glucose homeostasis (Table 2; Figure 5; Suppl File 1.6). Only two reviews were informed by an a priori protocol [67, 72] and few included considerations of grey literature [69, 72, 73]. Duplicate selection and extraction could be confirmed for all but two reviews [65, 71] Transparent reporting of included and excluded studies was provided by only two reviews [66, 72], whereas all reviews provided detailed study characteristics. Almost half of the included reviews provided no assessment of the quality of the included studies [67–70] nor considered quality further in their analyses [65]. Two reviews had problematic main analyses, as findings from the same sample of the population were used repeatedly within sub-analyses for each metabolite [69] or congener [72]. These analyses were excluded.

#### Thyroid function

Phthalates, flame retardants and PFAS were considered in 104 analyses of thyroid hormone levels to inform the impact of plastic-associated chemical exposure on thyroid function. Decreases in estimates of association were observed for DEHP phthalate metabolites (MEHP, MEHHP, MEOHP) across the majority of population groups investigated for TSH (9/12 EE), fT4 (8/12 EE) and TT4 (4/12 EE), including children, adults and pregnant women (Figure 5; Suppl File 2.4) [70].

MEHHP was significantly associated with a decreased fT4 in the general population, r –0.03, 95%CI –0.05 to –0.01, and adults alone r –0.08, 95%C –0.14 to –0.01, though this association was reversed in children, r 0.06, 95%CI: 0.01 to 0.10. MEOHP was associated with TT4 in children, r 0.05, 95%CI 0.01 to 0.10 (Figure 5; Suppl File 2.4) [70]. DEHP exposure was not significantly associated with any change in TSH (Figure 5; Suppl File 2.4) [70]. In the sub-population of pregnant women, no associations were observed for DEHP exposure or any of the thyroid hormones measured (9/9 EE; data not plotted; Suppl File 2.4) [70].

One review reported 66 main and subgroup analyses investigating exposure to PFAS, including PFOA, PFOS and PFHxS and thyroid function [71]. Of the main analyses, one presented a weak significant positive association for exposure to PFOS and fT4 concentration in adult blood, z 0.05, 95%CI 0.03 to 0.08; this weak association between PFOS and fT4 was maintained when pregnant women were excluded from the analysis, z 0.06, 95% CI 0.02 to 0.09 (Figure 5; Suppl File 2.4) [71]. A significant negative association was also observed with exposure to PFHxS and TT4 when pregnant women were excluded from the analysis, –0.04, 95%CI –0.07 to –0.01 (Figure 5; Suppl File 2.4) [71]. Of the remaining 11 main analyses, increasing PFAS exposure showed a decrease in thyroid function in four (4/11 EE), an increase in five (5/11 EE) and no change in three (3/11 EE; Figure 5; Suppl File 2.3) [71]. Associations appeared independent of the level (low, intermediate, high; random effects) of mean concentration of PFAS in the blood (30 EE; data not plotted) [71]. Of the remaining analyses of the sub-populations, when pregnant women were excluded, seven were showing a decreasing trend or no change (7/12 EE), whilst five showed an increasing trend (5/12 EE; Figure 5; Suppl File 2.4) [71]. Considering pregnant women only, six analyses of thyroid outcome measures (6/12 EE) showed some increase in measure, whilst three showed no change (3/12 EE; data not plotted; Suppl File 2.4) [71].

Two meta-analyses from one review informed the association between flame-retardant exposure and thyroid function (Figure 5) [72]. Results for the main analysis for this review were excluded due to unit of analysis errors (see endocrine outcomes main section above). Comparing serum PBDE levels, exposure to total PBDE levels between 35 and 100 ng/g lipid was associated with TT4, z 0.15, 95%CI 0.06 to 0.24 (Figure 5; Suppl File 2.4) [72]. No association was observed with total PBDE exposure <30 ng/g lipid and TSH, z –0.07, 95%CI –0.14 to 0.00 (Figure 5; Suppl File 2.4) [72].

#### Type 2 diabetes

BPA, phthalate plasticisers and flame retardants were considered in 16 meta-analyses of plastic-associated chemical exposure and risk of T2D. An increase in risk estimate was observed for all analyses informing PCB (8/8 EE), phthalates (3/3 EE) and BPA (5/5 EE) exposure; for the majority of analyses the association was statistically significant (Figure 5, Suppl File 2.4).

Three main analyses reported a statistically significant increased risk of T2D with exposure to BPA (3/3 EE; Figure 5). Two analyses reported a range from OR 1.28 to 1.47 [65, 66] and a third a RR 1.45, 95%CI 1.13 to 1.87 (highest versus lowest exposure, Figure 5; Suppl File 2.4) [67]. The significant association was also observed with subgroup analyses irrespective of whether the measure of exposure was determined from either urine or serum (2/2 EE; data not plotted; Suppl File 2.4) [65]. Considering phthalates, MiBP was significantly associated with higher risk of T2D, RR 1.90, 95%CI 1.17 to 3.09, while one main analysis of total phthalates and additional subgroup analysis of MEP suggested similar though non-significant increase of T2D in adults (Figure 5; Suppl File 2.4) [67].

Of the main analyses that investigated total PCB exposure in adults (Table 2), both reviews reported a statistically significant increase in risk of T2D (2/2 EE; Figure 5) with OR 1.7 [68] and RR 2.39 (highest versus lowest exposure) [67]. The review by Song et al. [67] included all of the studies that were included in the review by Wu et al. [68], as well as other retrospective studies (Suppl File 2.4). The significant association was also observed in subgroup analyses of females, but not males (2/2 EE; data not plotted; Suppl File 2.4) [67]. All analyses of total PCBs included some cohorts with either poisoning due to ingestion or instances of exposure to contaminated areas. Estimates of individual group II (PCB 118, 138) and group III (PCB 153, 180) congeners all increased with higher relative exposure, though non-significantly (4/4 EE; Figure 5; Suppl File 2.4) [68].

#### Diabetes-related metabolic traits

The same plastic-associated chemicals (BPA, phthalates and flame retardants) were considered in 20 meta-analyses investigating other diabetes-related metabolic traits; 13 informed the association with HOMA-IR from two reviews [67, 69], while the remaining analyses of other diabetes related measures were all derived from the same review (7/7 EE; highest to lowest exposure; Table 2; Figure 5) [67].

Higher BPA concentrations were significantly associated with higher HOMA-IR, MD 0.80 mg/dL, 95%CI 0.36 to 1.25 (Figure 5) [67]. Similarly, higher total phthalates concentrations were significantly associated with HOMA-IR, MD 0.71 mg/dL, 95% CI 0.30 to 1.12 (Figure 5) [67]. This relationship was maintained consistently when individual metabolites were examined (10/10 EE), with multiple metabolites showing significant associations (β range of 0.02 to 0.26), including MiBP, MBzP, MCPP as well as ∑DEHP and the individual DEHP metabolites, MEHP, MEOHP, MECPP (7/10 EE), while MMP, MEP and one DEHP metabolite, MEHHP, showed non-significant increases (3/10 EE; Figure 5; Suppl File 2.4) [69]. Results for the main analysis for this review were excluded due to unit of analysis error (see endocrine outcomes main section above) [69]. Conversely, total PCB exposure tended to decrease HOMA-IR, MD −2.05 mg/dL, 95%CI −4.65 to 0.56 (highest versus lowest exposure; Figure 5) [7]. Neither higher BPA nor higher total PCB exposure were significantly associated with fasting insulin (2/2 EE; Figure 5; Suppl File 2.4), nor was higher total PCB exposure significantly associated with lower 2hr insulin (Figure 5; Suppl File 2.4) [67].

Four meta-analyses from one review analysed blood glucose measures [67]. Exposure to higher total PCBs was significantly associated with an increase in fasting glucose, MD 3.27 mg/dL, 95%CI 1.87 to 4.67, and although neither higher BPA nor higher total phthalate concentrations were associated, both tended to increase non-significantly (Figure 5; Suppl File 2.4) [67]. Two-hour glucose increased with higher total PCB concentration (Figure 5; Suppl File 2.4) [67].

#### Polycystic ovary syndrome

One review of case control studies also reported meta-analyses of the differences in BPA levels detected in serum and follicular fluid (Table 2; Suppl File 2.4) [73]. Women with PCOS were found to have significantly higher BPA levels than women without PCOS, SMD 2.44, 95%CI 1.27 to 3.61 (Suppl Figure S1) [73]. This association was maintained when assessing serum samples only and when limited to women over 19 years of age (Suppl Figure S1; Suppl File 2.4) [73].

### Child neurodevelopment outcomes

**Figure 6:**
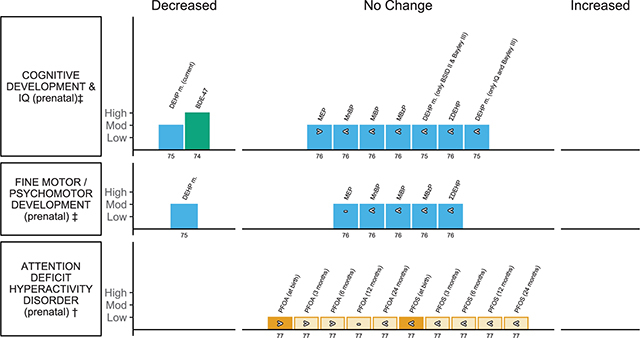
Harvest plot of exposure to plastic-associated chemicals and children’s neurodevelopmental outcome measures. Plastic-associated chemicals included are phthalates (blue) where exposure was determined based on monoester metabolites monoethyl phthalate (MEP), mono-n-butyl phthalate (MnBP), monoisobutyl phthalate (MiBP), monobenzyl phthalate (MBzP), molar sum of all di(2-ethylhexyl) phthalate metabolites measured (∑DEHP), and best single measure of metabolite(s) of di(2-ethylhexyl) phthalate (DEHP m.); flame retardants (green) including polybrominated diphenyl ethers (PBDEs) where exposure was determined based on a prevalent congener 2,2’,4,4’-tetrabromodiphenyl ether (BDE-47); and per- and polyfluoroalkyl *substances (*PFAS) (orange) including perfluorooctanoic acid (PFOA) and perfluorooctane sulfonate (PFOS). Outcome measures are either dichotomous (†) or measured on a continuous scale (‡). Outcomes include ‡Cognitive Development and Intelligence Quotient (IQ) (measured on the Mental Development Index (MDI) of the Bayley Scales of Infant Development, 2nd ed. (BSID-II), Cognitive Development subscale of the Bayley Scales of Infant and Toddler Development, 3rd ed. (Bayley-III), General Cognitive Scale (GCS) of the McCarthy Scales of Children’s Abilities (MSCA), and Full Scale IQ (FSIQ) of the Wechsler Preschool & Primary Scale of Intelligence (WPPSI) or Wechsler Intelligence Scale for Children (WISC)); ‡Fine Motor/Psychomotor Development (measured on the Psychomotor Development Index (PDI) of BSID-II, and Fine Motor subscale of Bayley-III) and †Attention Deficit Hyperactive Disorder (ADHD) (measured with the Attention Problems Syndrome Scale of the Child Behaviour Checklist (CBCL), the Hyperactivity/Inattention subscale of the Strengths and Difficulties Questionnaire (SDQ) and the ADHD Diagnostic and Statistical Manual of Mental Disorder 4th ed (DSM-IV)). Each bar represents an individual effect estimate from the corresponding review, which is indicated by the number below each bar. The height of the bar represents the quality score of the review assessed using the AMSTAR tool. Low quality reflects a score of 1–4, moderate (mod) quality a score of 5–8 and high quality a score of 9–11. Dark filled bars represent the primary analyses of each review; unfilled bars represent sub-group analyses. Bars have been assigned as an increase or decrease (columns) in the measure where the change is statistically significant. Remaining bars appearing under ‘no change’ indicate direction of effect as an increase (>), no clear trend (–) (the estimate of relative risk was 1 or regression coefficient or mean difference was 0), or decrease (<) in the measure or risk estimate.

There were three domains of neurodevelopmental outcome reported in children up to 12 years of age across three systematic reviews with meta-analyses and one pooled analysis. Of these, the evidence suggests an association with a decrease in children’s cognitive development and intelligence quotient (IQ), a decrease in fine motor development, and no change in attention deficit hyperactive disorder (ADHD) with exposure to plastic-associated chemicals evaluated (Figure 6). Child neurodevelopment outcomes were addressed for phthalates, flame retardants and PFAS (Table 2; Figure 6). Meta-analyses included separate consideration of prenatal and postnatal exposure to plastic-associated chemicals (Table 2).

The reviews that informed this outcome category ranged from moderate to high quality and scored between 7 and 11 on the AMSTAR tool, whilst the pooled analysis scored 3 (Table 2; Figure 6; Suppl File 1.6). The review by Lam et al. [74] informing the impact of flame-retardant exposure (BDE-47) on children’s IQ, fulfilled all of the AMSTAR criteria (11/11). Neither of the other reviews considered grey literature sources, nor transparent reporting of included and excluded studies [75, 76]. The reviews by Lam et al. [74] and Radke et al. [76] were informed by an a priori protocol. All of the reviews and pooled analysis provided detailed characteristics of included studies [74–77].

#### Cognitive development or intelligence quotient (IQ)

Eighteen meta-analyses, including both main and subgroup analyses, from two reviews informed the association between prenatal phthalate exposure and measures of cognitive development or IQ in children [75, 76]. The phthalate metabolites MEP, MnBP, MiBP, MBzP and DEHP metabolites, measured in urine or plasma, were investigated (18 EE). Of the main analyses, the majority reported a non-significant decrease in measures of cognitive development or IQ with increasing phthalates (6/7 EE), including ∑DEHP metabolites, β −0.1, 95%CI −0.8 to 0.5; DEHP metabolites, β −0.36, 95%CI −1.05 to 0.32; MnBP, β –0.2, 95%CI –0.7 to 0.4; MiBP, β –0.1, 95%CI –0.6 to 0.4, MBzP; β –0.1, 95%CI –0.8 to 0.5; except the phthalate metabolite MEP, β 0.3, 95%CI –0.3 to 0.9 (Figure 6) [75, 76]. Considering subgroups of girls and boys, in girls the majority of analyses similarly reported a non-significant, inverse association (4/5 EE; data not plotted; Suppl File 2.5) [76]. Whilst in boys, small, non-significant improvements in cognitive development or IQ were observed (4/5 EE, one EE no change; data not plotted; Suppl File 2.5) [76]. One meta-analysis evaluated the association between postnatal (current) phthalate exposure and measures of children’s cognitive development or IQ [75], with measures including the General Cognitive Scale (GCS) of the McCarthy Scales of Children’s Abilities (MSCA) and Full Scale IQ (FSIQ) of the Wechsler Preschool & Primary Scale of Intelligence (WPPSI) or Wechsler Intelligence Scale for Children (WISC) (Table 2). A significant reduction in cognitive performance or IQ, β –1.03, 95%CI –1.88 to 0.18, was found with postnatal exposure to DEHP metabolites (Figure 6; Suppl File 2.5) [75].

One meta-analysis informed the association between prenatal flame-retardant exposure and children’s IQ, assessed on the WPPSI or MSCA [74]. A significant inverse association was found with prenatal BDE-47 exposure and cognitive development or IQ, β –3.7 points, 95% CI: –6.56 to –0.83 (Figure 6; Suppl File 2.5) [74].

#### Fine motor or psychomotor development

Sixteen meta-analyses, including both main and subgroup analyses, from two reviews informed the association between prenatal phthalate exposure and measures of fine motor or psychomotor development in children, measured using Bayley Scales of Infant Development, 2nd ed. (BSID-II) or Bayley Scales of Infant and Toddler Development, 3rd ed. (Bayley-III) [75, 76]. There were four phthalate metabolites (MEP, MnBP, MiBP and MBzP) as well as DEHP metabolites investigated, measured in urine or plasma. Of the main analyses, prenatal DEHP metabolite exposure was associated with a decrease in psychomotor development in children, β –0.80, 95%CI –1.48 to –0.12 (1/6 EE; Figure 6). However, there were no significant changes with the other metabolites investigated, nor with ∑DEHP (5/6 EE; Suppl File 2.5; Figure 6) [75]. Considering girls and boys separately, higher prenatal MBzP exposure was also associated with a significant decrease in fine or psychomotor development (1/5 EE; data not plotted; Suppl File 2.5) [76], and non-significant inverse associations were observed for MnBP and MiBP in girls (2/5 EE; data not plotted; Suppl File 2.5) [76]. In boys, a small, non-significant, positive association was observed in the majority of analyses, as with cognitive development and IQ (4/5 EE; data not plotted; Suppl File 2.5) [76].

#### Attention deficit hyperactive disorder (ADHD)

Thirty meta-analyses from one pooled analysis [77] reported the association of prenatal exposure to PFOA and PFOS and ADHD in children 4–11 years of age. A pharmacokinetic model was used to generate estimates of PFOS and PFOA levels from birth until 24 months of age. No significant risk was observed with exposure to either PFOA (inter-quartile range –IQR increase 3–7ng/ml) or PFOS (IQR increase 1–5 ng/ml) at birth, 3, 6, 12 and 24 months and ADHD (10/10 EE; Figure 6; Suppl file 2.5) [77], with double the number of estimates indicating a decreased risk (6/10 EE; Figure 6) compared to an increased (3/10 EE; Figure 6) risk. Considering subgroups of girls and boys, in girls, risk of ADHD tended to increase with PFOA and PFOS exposure at all time points assessed (10/10 EE; data not plotted; Suppl File 2.5) [77]; the association was significant for PFOA exposure at birth and also at three months for ADHD (2/10 EE; data not plotted; Suppl file 2.5) [77]. Conversely, in boys, findings include both slight decreases (7/10 EE) and increases (3/10 EE) in risk estimates (data not plotted; Suppl File 2.5) [77].

### Nutritional outcomes

**Figure 7:**
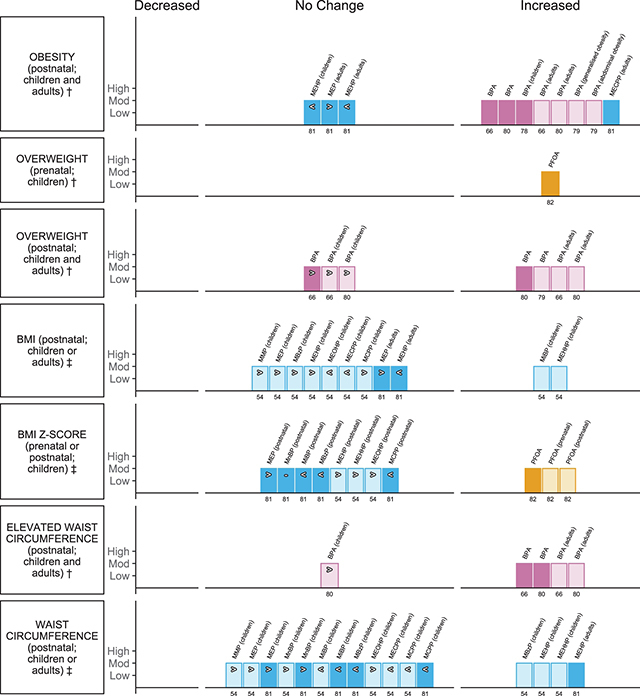
Harvest plot of exposure to plastic-associated chemicals and nutritional outcome measures. Plastic-associated chemicals included are bisphenol A (BPA) (pink) and phthalate monoester metabolites (blue), including monomethyl phthalate (MMP), monoethyl phthalate (MEP), mono-n-butyl phthalate (MnBP), monoisobutyl phthalate (MiBP), monobenzyl phthalate (MBzP), mono(2-ethylhexyl) phthalate (MEHP), mono(2-ethyl-5-hydroxyhexyl) phthalate (MEHHP), mono(2-ethyl-5-oxohexyl) phthalate (MEOHP), mono(2-ethyl-5-carboxypentyl) phthalate (MECPP), mono-n-octyl phthalate (MnOP) and mono (3-carboxypropyl) phthalate (MCPP). Outcome measures are either dichotomous (†) or measured on a continuous scale (‡). Outcomes measured include †obesity including abdominal obesity and generalized obesity, †overweight including generalized overweight, ‡Body Mass Index (BMI) and ‡BMI z score, †elevated waist circumference and ‡waist circumference. Each bar represents an individual effect estimate from the corresponding review, which is indicated by the number below each bar. The height of the bar represents the quality score of the review assessed using the AMSTAR tool. Moderate quality reflects a score of 5–8. Dark filled bars represent the primary analyses of each review; light filled bars represent sub-group analyses. Bars have been assigned as an increase or decrease (columns) in the measure where the change is statistically significant. Remaining bars appearing under ‘no change’ indicate direction of effect as an increase (>), no change (–) (the estimate of relative risk was 1 or regression coefficient or mean difference was 0), or decrease (<) in the measure or risk estimate.

There were multiple nutritional outcomes reported in seven systematic reviews with meta-analyses. The available evidence suggests an increased risk of obesity and related anthropometric measures—overweight, BMI and elevated waist circumference—with exposure to plastic-associated chemicals (Figure 7). Nutritional outcomes were addressed for BPA, phthalates and PFAS. Exposure to plastic-associated chemicals was postnatal in the majority of included meta-analyses for both children and adults, with prenatal exposure also assessed for PFAS (Table 2).

The reviews that informed this outcome category were all of moderate quality, scoring between 5 and 7 on the AMSTAR tool (Table 2; Suppl File 1.6). None of the included reviews were informed by an a priori protocol, and it was unclear in three reviews if duplicate extraction of data was performed [66, 78, 79]. Only one of the reviews that informed this outcome category provided a complete list of excluded as well as included studies [66], whereas two reviews out of the five included considered the results of critical appraisal in the analysis. It was unclear in two of the included reviews if statistical analysis was appropriate [79, 80].

#### Obesity

Fifteen meta-analyses, including both main and subgroup analyses, from five systematic reviews informed the association between BPA and phthalates and risk of obesity [66, 78–81]. None of the included reviews used a reference standard for categorisation of obesity.

Two meta-analyses reported a significantly increased risk of obesity with higher BPA exposure in the general population with an OR range of 1.57 to 1.67 (2/2 EE; Figure 7; highest versus lowest category; Suppl File 2.6) [66, 80]. This finding was maintained in subgroup analyses considering different patterns of obesity, with significant associations reported for both generalised obesity, OR 1.83, 95% CI 1.59 to 2.12, and abdominal obesity, OR 1.43, 95%CI 1.27 to 1.62 (2/2 EE; Figure 7; highest versus lowest category) [79], as well as in a dose response analyses for these two outcomes (2/2 EE; per 1ng/mL increase in BPA; data not plotted; Suppl File 2.6) [79]. A significant association was also maintained in an analysis of postnatal exposure in children alone, OR 1.57, 95%CI 1.09 to 2.23 (Figure 7; Suppl File 2.6) [78], and in adults alone, an OR range of 1.50 to 1.60 (2/2 EE; Figure 7; Suppl File 2.6) [66, 80], although an alternative meta-analytical approach applied to studies of children did not find a statistically significant difference in urinary BPA concentration in obese and non-obese children (Suppl File 2.6; 2 EE, data not plotted) [78].

One review assessed the association of three phthalate metabolites, MEP, MEHP and MECPP, and risk of obesity (Figure 7) [81]. A significant increase in risk of obesity in adults was observed with the DEHP metabolite MECPP, OR 1.67, 95%CI 1.3 to 2.16, and was also observed for MEP, though non-significant (Figure 7; high versus low exposure; Suppl File 2.6) [80]. A non-significant reduction in the risk estimate was observed with the DEHP metabolite MEHP (Figure 7; Suppl File 2.6) [80]. The only meta-analysis of childhood obesity was for MEHP, with a similar non-significant inverse association (Figure 7; Suppl File 2.6) [80].

#### Overweight

Eight meta-analyses including both main and subgroup analyses from four systematic reviews informed the association between risk of overweight and exposure to BPA and PFOA [66, 79, 80, 82]. No reference standard for overweight was provided by any of the included reviews.

Of three analyses including both children and adults, two reported a significant increase in risk of overweight with higher exposure to BPA, OR range of 1.24 to 1.32 (Figure 7) [79, 80], while a similar increase was reported, though non-significant, in the other meta-analysis, OR 1.21, 95%CI 0.98 to 1.50 (Figure 7) [66]. This relationship with higher BPA exposure was maintained in a dose response analysis (per 1ng/mL increase in BPA; data not plotted; Suppl File 2.6. [79] Similarly, this significant risk of overweight was also observed in meta-analyses from two reviews including only adults (same studies included), OR 1.25, 95%CI 1.01 to 1.56 (Figure 7; 2/2 EE; Suppl File 2.6) [66, 80], while only the positive trend in the association was maintained in children (2/2 EE; Figure 7; Suppl File 2.6) [66, 80].

Similar to the effects reported with exposure to BPA, in a main analysis investigating prenatal PFOA exposure, a significant association with risk of overweight was observed in children, ES 1.25, 95%CI 1.04 to 1.50 (Figure 7; Suppl File 2.6) [82].

#### Body mass index

Twenty-four meta-analyses, including both main and subgroup analyses, from three systematic reviews informed the association between exposure to phthalates or PFAS, and BMI or BMI z-score [54, 81, 82]. The majority of phthalates assessed showed a positive association with increased BMI in children with increasing concentrations of phthalate metabolites (10/12 EE) [54]. Of these, two metabolites, MiBP and MEHHP, showed a statistically significant increase in BMI, whereas a small, non-significant reduction in BMI was reported with increasing MEOHP and MECPP (Figure 7; Suppl File 2.6) [54]. Similar trends were observed when considering BMI z-score in children, with all metabolites assessed by one review (3/3 EE; MEHP, MEHHP and MEOHP) showing a small, non-significant increase in BMI z-score with increasing urinary phthalate concentration (Figure 7; Suppl File 2.6) [54]. In another review, however, no change was reported with MnBP exposure in children, while a small, non-significant, positive association was reported for MEP and small, non-significant reductions in BMI z-score with increasing concentration of MiBP, MBzP and MCPP (Figure 7; 3/5 EE; Suppl File 2.6) [81]. Only two metabolites were assessed in adults for BMI small positive association with MEP, and a small negative association with MEHP (Figure 7; Suppl File 2.6) [81].

One systematic review presented one main analysis and four subgroup analyses investigating BMI z-score and the association with PFOA exposure in children. A significant increase in BMI z-score was observed with increasing PFOA exposure in children, β 0.10, 95%CI 0.03 to 17.00 (Figure 7) [82], a relationship that was maintained irrespective of whether exposure was prenatal, β 0.09, 95%CI 0.02 to 0.17, or postnatal, β 0.16, 95%CI 0.01 to 0.30 (Figure 7; 3/3 EE) [82]. This small, positive association with PFOA exposure was maintained in girls; however, not in boys (Figure 7; data not plotted; Suppl File 2.6) [82].

#### Waist circumference

Twenty-one meta-analyses, including both main and subgroup analyses, from four reviews informed the association between BPA, phthalates and waist circumference [54, 66, 80, 81].

Two meta-analyses from two reviews found a consistent association between elevated waist circumference and BPA exposure with an OR range of 1.48 to 1.49 (Figure 7; Suppl File 2.6) [66, 80]. No reference for elevated waist circumference was provided in either review. This significant association with BPA exposure, as observed with other anthropometric measures related to obesity, was maintained in adults, OR range of 1.50 to 1.52 (Figure 7; Suppl File 2.6) [66, 80]. In children, a similar positive association was also observed; however, this was not statistically significant, OR 1.62, 95%CI 0.97 to 2.72 (Figure 7) [80].

Two reviews reported 15 meta-analyses assessing the association of increasing phthalate levels with waist circumference in children [54, 81]. A positive association was reported for MEP and MnBP (4/4 EE) from both reviews, whilst a negative association was reported for MiBP and MCPP from both reviews (4/4 EE). For MBzP, the results were inconsistent, with a negative association found in one (Figure 7; Suppl File 2.6) [81], but a statistically significant positive association in the other (Figure 7; Suppl File 2.6) [56]. For the remaining phthalate metabolites assessed, including MMP, MEP, MEHP, MEHHP and MEOHP, positive associations were observed with waist circumference, which were statistically significant for MEHP and MEHHP (Figure 7; Suppl File 2.6) [56]. Only one metabolite, MEHP, was assessed for adults, with a finding of a significant positive association with increased waist circumference, β 0.58, 95%CI 0.55 to 0.62 (Figure 7) [81], consistent with that found for children.

### Circulatory outcomes

**Figure 8:**
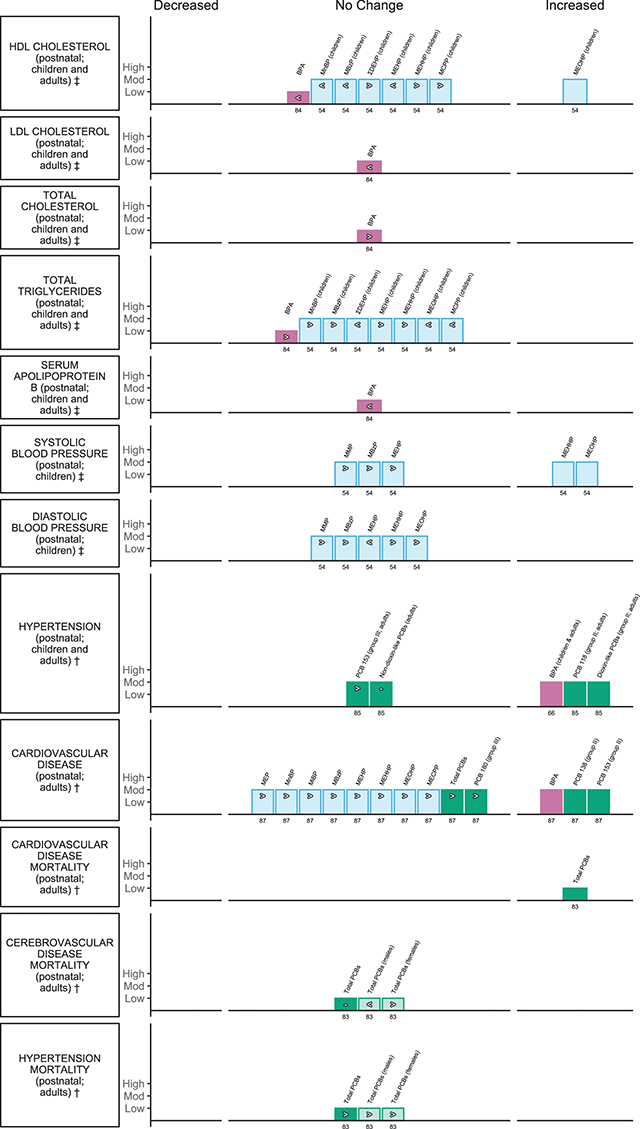
Harvest plot of exposure to plastic-associated chemicals and circulatory outcome measures. The plastic-associated chemicals included are bisphenol A (BPA) (pink); phthalate monoester metabolites (blue), including monomethyl phthalate (MMP), monoethyl phthalate (MEP), mono-n-butyl phthalate (MnBP), monoisobutyl phthalate (MiBP), monobenzyl phthalate (MBzP), mono(2-ethylhexyl) phthalate (MEHP), mono(2-ethyl-5-hydroxyhexyl) phthalate (MEHHP), mono(2-ethyl-5-oxohexyl) phthalate (MEOHP), mono(3-carboxypropyl) phthalate (MCPP); mono(2-ethyl-5-carboxypentyl) phthalate (MECPP), and the molar sum of the di(2-ethylhexyl) phthalate metabolites (∑DEHP); and flame retardants (green) including polychlorinated biphenyl (PCB). Outcome measures are either dichotomous (†) or measured on a continuous scale (‡). Outcomes measured include serum lipids encompassing concentrations in low-density lipoprotein (LDL), high-density lipoprotein (HDL), total cholesterol (TC), triglycerides (TG) and apolipoprotein B (ApoB); child systolic blood pressure (SBP); child diastolic blood pressure (DBP); cardiovascular disease (CVD; for BPA and phthalates children also included with sampling frame [17]); CVD mortality; cerebrovascular disease mortality; hypertension and hypertension mortality. Each bar represents an individual effect estimate from the corresponding review, which is indicated by the number below each bar. The height of the bar represents the quality score of the review assessed using the AMSTAR tool. Low quality reflects a score of 1–4 and moderate (mod) quality a score of 5–8. Dark filled bars represent the primary analyses of each review; light filled bars represent sub-group analyses. Bars have been assigned as an increase or decrease (columns) in the measure where the change is statistically significant. Remaining bars appearing under ‘no change’ indicate direction of effect as an increase (>), no change (–) (the estimate of relative risk was 1 or regression coefficient or mean difference was 0), or decrease (<) in the measure or risk estimate.

There were seven circulatory outcomes reported in four systematic reviews with meta-analyses and two pooled analyses. Of these, the evidence suggests an association with increased systolic blood pressure (SBP) and increased high-density lipoprotein (HDL) levels in children, increased risk of hypertension in adults and increased risk of CVD and CVD mortality with exposure to the plastic-associated chemicals evaluated (Figure 8). Circulatory outcomes were addressed for BPA, phthalates and flame retardants. Exposure to plastic-associated chemicals was measured in children and adults, and outcome measures included serum lipids (HDL, low-density lipoprotein [LDL], total cholesterol [TC], triglycerides [TG] and apolipoprotein B [ApoB]), blood pressure (SBP and diastolic [DBP]), risk of CVD and hypertension and mortality attributable to CVD, cerebrovascular disease and hypertension (Table 2).

The reviews that informed this outcome category were of moderate quality, scoring between 5 and 7 on the AMSTAR tool, whereas the pooled analysis ranged from low to moderate quality, scoring 4 [83] and 6 [84] respectively (Table 2; Suppl File 1.6). Consistent with many of the other outcomes reported here, reviews that informed this category failed to provide any evidence of an a priori protocol. In one review [85] and one pooled analysis [83] it was clear that data extraction was performed in duplicate. None of the reviews considered grey literature, and only one review provided clarity regarding study inclusion and exclusion and adequate details to completely assess the methods of synthesis used [66].

#### Serum lipid levels

Forty-nine meta-analyses, including both main and subgroup analyses from one systematic review and one pooled analysis, informed the association between BPA or phthalate metabolites exposure and measures of serum lipids in children and adults [54, 84]. Of the five main meta-analyses of children and adults, there was no significant association with BPA exposure and changes in HDL, LDL, TC, TG and ApoB; however, the majority of estimates tended to decrease, an undesirable effect in the case of HDL cholesterol, with increased exposure (3/5 EE; Figure 8; Suppl File 2.7) [84]. Similarly, in the 30 subgroup analyses of children and adults separately, including analyses for males and females for each outcome measure, the majority of serum lipid measures also tended to decrease, though not significantly (25/30 EE; data not plotted; Suppl File 2.7) [84].

One review presented 14 subgroup meta-analyses investigating the association between phthalate metabolites and HDL and TG in children [54]. Results for the main analysis for this review were excluded due to unit of analysis error. A beneficial increase in serum HDL levels was observed with increasing concentration of one DEHP metabolite, MEOHP, z 0.31, 95%CI 0.25 to 0.37, but with non-significant findings in each direction for two other DEHP metabolites, MEHHP and MEHP, and a much-attenuated overall finding for ∑DEHP, z 0.09, 95%CI –0.26 to 0.44. Of the other phthalate metabolites evaluated, there were non-significant decreases in serum HDL (undesirable) for MnBP and MBzP (2/3 EE), but an increase for the nonspecific phthalate metabolite MCPP (1/3 EE; Figure 8; Suppl File 2.7) [54]. The observed profile was largely inversed for serum TG, with non-significant beneficial decreases observed for increasing concentration of ∑DEHP, MEOHP and MCPP, and a non-significant undesirable increase in circulating TG with the remaining metabolites investigated (4/4 EE; Figure 8; Suppl File 2.7) [54].

#### Blood pressure and hypertension

One systematic review with ten subgroup meta-analyses informed the association between phthalates and SBP and DBP in children [54]. Results for the main analysis for this review were excluded due to unit of analysis error. All meta-analyses reported a positive association with SBP (5/5 EE) with increasing postnatal phthalate metabolites. For two metabolites, the association was significant: MEHHP, β 0.16, 95%CI 0.09 to 0.23, and MEOHP, β 0.12, 95%CI 0.12 to 0.24 (Figure 8; Suppl File 2.7) [54]. Similarly, positive associations were observed for DBP for the majority of metabolites investigated, (4/5 EE) except MEHP, where DBP decreased slightly with increasing concentration (Figure 8; Suppl File 2.7) [54].

Two reviews, including five meta-analyses informed the association between BPA and flame retardant exposure and hypertension [66, 85]. A significant increase in hypertension (SBP >140mmHg and/or DBP >90mmHg) was reported with exposure to BPA, OR 1.41, 95%CI 1.12 to 1.79 in adults (Figure 7; highest vs lowest exposure) [66]. Similarly, in the analyses investigating flame retardant exposure and hypertension (SBP >140mmHg and/or DBP >90mmHg; receiving medication or doctor diagnosed), a significant positive association with hypertension was observed with the sum of group II dioxin like PCBs (following the Wolff et al. classification [86]), OR 1.45 95%CI 1.00 to 2.12, and the individual group II PCB 118, OR 1.26, 95%CI 1.00 to 1.58 (highest to lowest exposure; Figure 8; Suppl File 2.7) [85]. A non-significant positive association was also reported with exposure to the non-dioxin-like group III PCB 153, but not with combined exposure to non-dioxin-like PCBs (Figure 8; Suppl File 2.7) [85].

#### Cardiovascular disease (CVD)

One systematic review comprising 13 main and subgroup meta-analyses informed the association between BPA, phthalate and flame retardant exposure and risk of CVD in children and adults [87]. Results for the two overall analyses for phthalates and PCBs were excluded due to unit of analysis errors. Of the main analyses evaluating BPA and three individual PCBs (138, 153, 180), 3/4 reported an increased risk of CVD with exposure to BPA OR 1.19, 95%CI 1.03 to 1.37, and the flame retardants PCB 138, OR 1.35, 95%CI 1.10 to 1.66, and PCB 153, OR 1.35, 95%CI 1.13 to 1.62 (Figure 8; highest vs. lowest or per unit increase) [87]. Non-significant increased risk was observed for total PCBs and PCB 180 (Figure 8; Suppl File 2.7) [87]. Similarly, risk of CVD tended to increase, though non-significantly, with all eight phthalate metabolites investigated in subgroup meta-analyses (8/8 EE; Figure 8; Suppl File 2.7) [87].

#### Mortality—cardiovascular disease, cerebrovascular disease and hypertension

One pooled analysis of two highly exposed cohorts presented seven meta-analyses investigating mortality attributable to CVD, cerebrovascular disease and hypertension respectively, following incidents of PCB poisoning [83]. An increased risk of CVD deaths was observed with PCB poisoning with a reported a SMR of 1.3, 95%CI 1.0 to 1.7, though no significant change was observed for cerebrovascular disease deaths SMR 1.0, 95%CI 0.8 to 1.29, which was consistent in sub-group meta-analysis for males and females (Figure 8; 2/2 EE increase; Suppl File 2.7) [83]. A non-significant increase in deaths attributable to hypertension was similarly reported in exposed adults, SMR 1.6, 95%CI 0.9 to 2.9 (Figure 8) [83], a trend maintained in the sub-analyses for both males and females (Figure 8; 2/2 EE; Suppl File 2.7) [83].

### Respiratory outcomes

**Figure 9:**
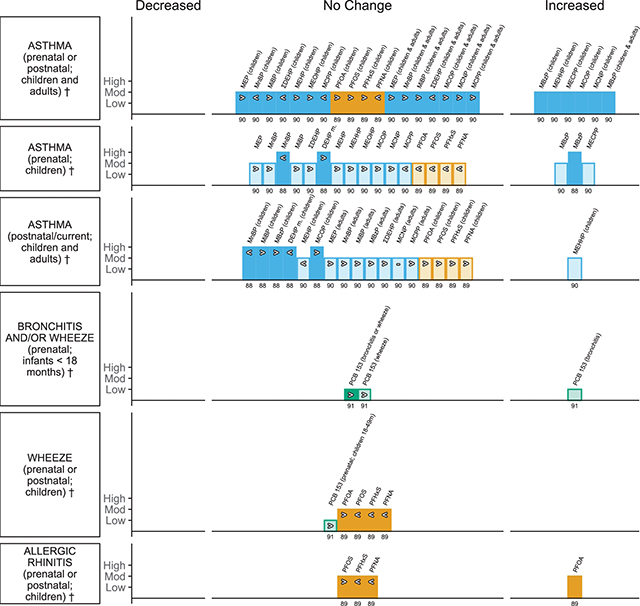
Harvest plot of exposure to plastic-associated chemicals and respiratory outcomes. Plastic-associated chemicals included are phthalate monoester metabolites (blue), encompassing monoethyl phthalate (MEP), mono-n-butyl phthalate (MnBP), monoisobutyl phthalate (MiBP), monobenzyl phthalate (MBzP), mono(2-ethylhexyl) phthalate (MEHP), mono(2-ethyl-5-hydroxyhexyl) phthalate (MEHHP), mono(2-ethyl-5-oxohexyl) phthalate (MEOHP), mono(2-ethyl-5-carboxypentyl) phthalate (MECPP), molar sum of the di(2-ethylhexyl) phthalate metabolites (∑DEHP), mono(carboxyisooctyl) phthalate (MCOP), monocarboxyisononyl phthalate (MCNP), and mono (3-carboxypropyl) phthalate (MCPP); flame retardants (green) including polychlorinated biphenyl (PCB); and per- and polyfluoroalkyl *substances (*PFAS) (orange) including perfluorohexane sulfonate (PFHxS), perfluorooctanoic acid (PFOA), perfluorooctane sulfonate (PFOS) and perfluorononanoic acid (PFNA). Outcomes are dichotomous (†) and include risk of asthma, bronchitis, wheeze and allergic rhinitis. Each bar represents an individual effect estimate from the corresponding review, which is indicated by the number below each bar. The height of the bar represents the quality score of the review assessed using the AMSTAR tool. Low quality reflects a score of 1–4, moderate (mod) quality a score of 5–8 and high quality a score of 9–11. Dark filled bars represent the primary analyses of each review; light filled bars represent sub-group analyses. Bars have been assigned as an increase or decrease (columns) in the measure where the change is statistically significant. Remaining bars appearing under ‘no change’ indicate direction of effect as an increase (>), no change (–) (the relative estimate was 1), or decrease (<) in the estimate.

There were four respiratory outcomes reported in three systematic reviews with meta-analyses and one pooled analysis. Of these, the evidence suggests an association with increased risk of asthma with some phthalate metabolites, MBzP in particular, bronchitis in children with exposure to PCBs and allergic rhinitis with exposure to PFOA (Figure 9). Respiratory outcomes were addressed for phthalates, flame retardants and PFAS. Outcomes included asthma in three reviews [88–90], wheeze in two reviews [89, 91], and bronchitis [91] and allergic rhinitis [89] in one review each (Table 2). Exposure to plastic-associated chemicals included both prenatal and postnatal for children (Table 2). The majority of the included reviews assessed categorical, high versus low, exposure.

The reviews that informed this outcome category scored between 5 and 9 on the AMSTAR tool, whereas the pooled analysis [91] scored 3 (Table 2; Suppl File 1.6). The evidence informing the impact of phthalates and PFAS was all high to moderate quality. None of the included studies searched grey literature, nor provided complete indication of study inclusion and exclusion, nor considered the results of appraisal (which was performed by all except in the pooled analysis) in the analysis. This was with the exception of one review by Li et al. [88], which was also the only review to be informed by an a priori protocol. Where it could be adequately determined, the statistical analysis appeared appropriate in all of the studies that informed this outcome.

#### Asthma

Two systematic reviews and one published meta-analysis presenting 88 main and subgroup meta-analyses informed the association between plastic-associated chemical exposure and asthma (highest versus lowest exposure) [88–90]. Main analyses presented by Wu et al. [90] considered 11 urinary phthalates as well as ∑DEHP. In both main and subgroup analyses investigating phthalate metabolites in children (Figure 9; Table 2), a statistically significant increase in risk of asthma with MBzP was reported OR 1.17, 95%CI 1.05 to 1.29 [90]. In further main analyses, significant associations with asthma in children were also observed with DEHP metabolites MEHHP, OR 1.13, 95%CI 1.03 to 1.24, and MECPP, OR 1.20, 95%CI 1.00 to 1.42, as well as with metabolites of related higher molecular weight phthalates, including mono (carboxy-isooctyl) phthalate (MCOP), OR 1.19, 95%CI 1.02 to 1.37, and mono (carboxynonyl) phthalate (MCNP) OR 1.15, 95%CI 1.00 to 1.31 (Figure 9; Suppl File 2.8) [90]. Similarly, risk of asthma in children tended to also increase though not significantly, with some remaining metabolites investigated, except for MnBP, MCPP and ∑DEHP (Figure 5; 4/7 EE; Suppl File 2.8) [90]. This relationship remained consistent when timing of exposure was explored in children, with significant associations observed with prenatal MBzP, showing an OR range of 1.15 to 1.38 and MECPP, OR 1.23, 95%CI 1.03 to 1.47 (Figure 9; Suppl File 2.8) [90]. The positive trend with phthalates was maintained across the majority of remaining analyses (11/13 EE; Figure 9; Suppl File 2.8) [90]. Results were less equivocal with postnatal phthalates in children. One metabolite exposure, MEHHP, resulted in a significant increase in risk of asthma, OR 1.30, 95%CI 1.09 to 1.65 (Figure 9) [90], and three of the remaining six analyses showed non-significant increases (3/6 EE; Figure 9; Suppl File 2.8) [90]. The majority of further sub-analyses in the general population showed a trend to towards an increase in risk of asthma with phthalate metabolites when restricted to postnatal assessment and also in adults only (13/15 EE; Figure 9; postnatal only, data not plotted; Suppl File 2.8) [90]; a significant association was observed for postnatal exposure to MBzP (Figure 9; Suppl File 2.8) [90]. No significant associations were reported with subgroups of males or females with over half of analyses tending towards positive association (7/12 EE) and the remainder negative (5/12 EE; data not plotted; Suppl File 2.8) [90].

Four meta-analyses from one review assessed the association between exposure to PFAS and risk of asthma in children up to 19 years old (Table 2) [89]. No statistically significant risk of asthma was reported; however, small increases were observed with exposure to PFOA, PFOS, PFHxS (3/4 EE) though not PFNA (Figure 9; Suppl File 2.8) [89]. Similar non-significant increases were observed when only postnatal exposure was included for each analysis (4/4 EE; Figure 9; Suppl File 2.8) [89]. However, this trend was reversed with prenatal exposure (4/4 EE; Figure 9; Suppl File 2.8) [89].

#### Bronchitis and/or wheeze

One pooled analysis with six meta-analyses informed the association between flame retardants and bronchitis in children less than 18 months [91]. Increasing PCB 153 exposure was associated with an increased risk of bronchitis in these children, RR per doubling exposure 1.06, 95%CI 1.01 to 1.12 (Figure 9; Suppl File 2.8) [91]. This positive association was no longer significant when exposure was analysed categorically (2/2 EE; highest, medium versus lowest; data not plotted; Suppl file 2.8) [91]. Similarly, three main analyses assessing risk of bronchitis and/or wheeze in infants reported a small increase in RR per doubling of exposure 1.02, 95%CI 0.96 to 1.12 (Figure 9; Suppl File 2.8) [91], whereas the direction of this association was reversed with categorical analysis (highest, medium vs. lowest), though neither risk estimates were statistically significant (2/2 EE; Figure 9; Suppl File 2.8; data not plotted) [91]. Similar results were reported in the cohorts analysed when considering wheeze alone, with small positive associations observed per doubling of exposure in children under 18 months old and also in the cohort with an average age over 18 months (2/2 EE; Figure 9; Suppl File 2.8) [91]. Similar, non-significant positive associations were observed for these outcomes with categorical analyses, comparing high and medium versus low PCB exposure (3/4 EE; data not plotted; Suppl File 2.8) [91]. Exposure to PFAS and risk of wheeze in children was also assessed in four meta-analyses from one review (Table 2) [89]. No significant risk of wheeze was reported. However, small decreases in risk were observed with exposure to PFOS, PFHxS, PFNA (3/4 EE) though not PFOA (Figure 9; Suppl File 2.8) [89]. An identical trend for each PFAS was observed when prenatal exposure was considered alone (4/4 EE; data not plotted; Suppl File 2.8) [89].

#### Allergic rhinitis

Eight meta-analyses from one review assessed the association between exposure to PFAS and risk of allergic rhinitis in children up to 19 years old (Table 2) [89]. A significant association with increased risk of allergic rhinitis was observed with exposure to PFOA, OR 1.32, 95%CI 1.13 to 1.55, while exposure to PFOS only increased risk minimally. Conversely, PFHxS and PFNA exposure led to small decreases in the observed risk estimates (Figure 9; Suppl File 2.8) [89]. A similar trend for each PFAS, including significant risk with PFOA was maintained with prenatal exposure only (4/4 EE; data not plotted; Suppl File 2.8) [89].

### Skin-related outcomes

**Figure 10:**
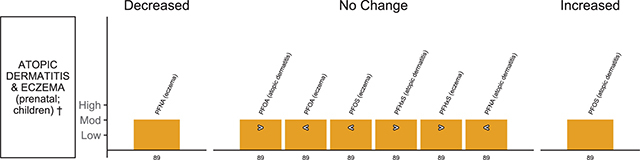
Harvest plot of prenatal exposure to plastic-associated chemicals and skin-related outcomes in children. Plastic-associated chemicals included are per- and polyfluoroalkyl *substances (*PFAS) (orange), encompassing perfluorohexane sulfonate (PFHxS), perfluorooctanoic acid (PFOA), perfluorooctane sulfonate (PFOS) and perfluorononanoic acid (PFNA). Outcomes are dichotomous(†) and include atopic dermatitis and eczema. Each bar represents an individual effect estimate from the corresponding review, which is indicated by the number below each bar. The height of the bar represents the quality score of the review assessed using the AMSTAR tool. Moderate (mod) quality reflects a score of 5–8. Dark filled bars represent the primary analyses of each review; light filled bars represent sub-group analyses. Bars have been assigned as an increase or decrease (columns) in the measure where the change is statistically significant. Remaining bars appearing under ‘no change’ indicate direction of effect as an increase (>) or decrease (<) in the measure or risk estimate.

One skin related outcome was reported in one systematic review with meta-analyses [89]. In this review, a distinction was made between studies of atopic dermatitis and eczema, and these were meta-analysed separately. However, no justification was provided for the distinction between these two closely related terms and a combined analysis is not provided. The data as analysed suggest prenatal exposure to PFNA may have a protective effect against risk of eczema in children (Figure 10); however, this is unclear as this significant effect was not replicated in the analysis of atopic dermatitis studies. Exposure to plastic-associated chemicals was prenatal and details of type of samples measured are provided in Table 2.

The review that informed the evidence regarding the impact of PFAS on this outcome category was rated as moderate quality, scoring 7/11 on the AMSTAR tool (Table 2; Suppl File 1.6). As with other reviews in this field, there was no evidence of an a priori protocol, grey literature was not considered, nor was a complete list of included and excluded studies provided. While appraisal of the included studies was performed, nowhere was it apparent that these results were then considered further in the analysis presented.

#### Atopic dermatitis and eczema

Eight meta-analyses from one systematic review informed the association between prenatal PFAS exposure and atopic dermatitis and eczema (Table 2; high versus low exposure) [89]. Exposure to PFNA appeared to result in a statistically significant decrease in risk of eczema, OR 0.89, 95%CI 0.80 to 0.99, and a similar non-significant decrease in risk of atopic dermatitis (2/2 EE; Figure 10). A reduction in the risk of eczema was also observed with PFOS and PFOA (2/2 EE; Figure 10; Suppl File 2.9) [89]. Risk of eczema tended to increase with exposure to PFHxS (1/4 EE) and atopic dermatitis with PFOA, PFOS and PFHxS exposure (3/4 EE) Figure 10; Suppl File 2.9) [89]. However, only PFOS was significantly associated with atopic dermatitis (Figure 10; Suppl File 2.9) [89].

### Cancer outcomes

**Figure 11:**
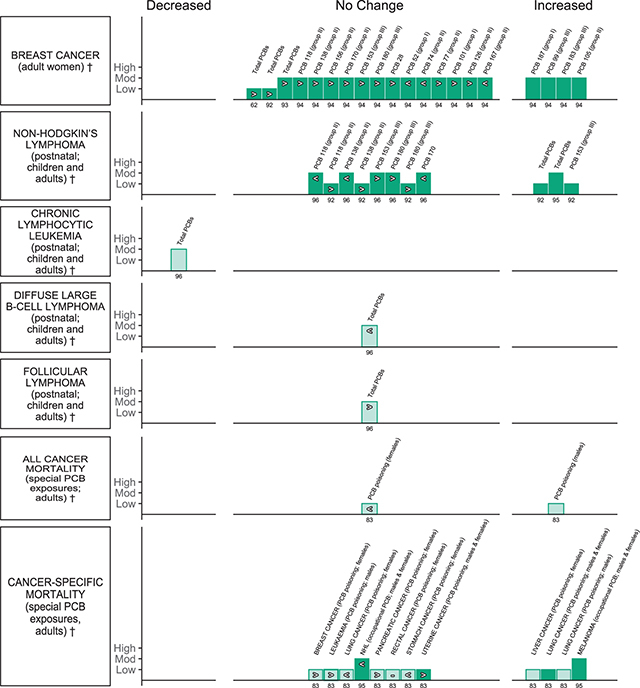
Harvest plot of exposure to plastic-associated chemicals and cancer outcomes. Plastic-associated chemicals included are flame retardants (green), including polychlorinated biphenyl (PCB) further organised by group – gp I (44, 52, 101, 107, 187, 201), gp II (105, 118, 138, 156, 167, 170) and gp III (99, 153, 180, 183, 203) as well as PCB 28. Outcomes are dichotomous (†) and include breast cancer, non-Hodgkin’s lymphoma (NHL), NHL subtypes—chronic lymphocytic leukemia (CLL), diffuse large B-cell lymphoma (DLBCL), follicular lymphoma (FL)—and cancer-specific mortality: all cancer, breast cancer, leukaemia, liver cancer, lung cancer, melanoma, NHL, pancreatic cancer, rectal cancer, stomach cancer and uterine cancer. PCB poisoning refers to populations exposed to PCB-contaminated food and PCB occupational refers to populations occupationally exposed to PCBs. Each bar represents an individual effect estimate from the corresponding review, which is indicated by the number below each bar. The height of the bar represents the quality score of the review assessed using the AMSTAR tool. Low quality reflects a score of 1–4 and moderate (mod) quality a score of 5–8. Dark filled bars represent the primary analyses of each review; light filled bars represent sub-group analyses. Bars have been assigned as an increase or decrease (columns) in the measure where the change is statistically significant. Remaining bars appearing under ‘no change’ indicate direction of effect as an increase (>) or decrease (<) in the measure or risk estimate.

The association between plastic-associated chemical exposure and occurrence of three different types of cancer was reported in six systematic reviews with meta-analyses and one pooled analysis. Of these, the evidence suggests an association with an increased risk of non-Hodgkin’s lymphoma (NHL) with occupational PCB exposure, as well as increased risk of breast cancer with exposure to four individual PCB congeners (Figure 11). There was, however, also evidence of a protective effect for chronic lymphocytic leukemia—a subtype of NHL. A further 11 cancer-related mortality outcomes were evaluated in one systematic review with meta-analyses and one pooled analysis. Evidence was found of an increased risk for all cancer-related mortality in males, liver cancer mortality in females and mortality attributable to lung cancer and malignant melanoma. Flame retardants, specifically PCBs, were the only plastic-associated chemicals evaluated for cancer outcomes. Breast cancer was the most commonly investigated type of cancer reported in four reviews [62, 92–94], followed by NHL and its subtypes in three reviews [92, 95, 96]. Cancer specific mortality was reported in one review [95] and one pooled analysis [83] and all cancer mortality in one pooled analysis [83]. Cancer-related mortality was predominantly assessed in highly exposed cohorts arising from occupational exposure or incidents of PCB poisoning (Table 2).

The reviews that informed the impact of PCBs on this outcome category ranged from moderate to low quality scored between 2 and 8 on the AMSTAR tool (Table 2; Suppl File 1.6). Only one review was informed by an a priori protocol [96], whereas only two of the included studies provided clear indication of duplicate data extraction [83, 93]. Consistent with most of the reviews informing this project, grey literature searching was not performed by any review and clear reporting of excluded studies in particular was also poor, with only one review [94] and the pooled analysis [83] informing this outcome providing the expected details. Half of the reviews critically appraised the included studies and of those that did [93, 94, 96], only the review by Zhang et al. [93] considered the results of the appraisal further in the analysis, which was appropriate in most studies (Suppl File 1.6).

#### Breast cancer

Twenty-two meta-analyses informed the association between flame retardant exposure and risk of breast cancer. Three reviews presented main analyses indicating non-significant associations between total PCB exposure and breast cancer, range of OR 1.09 to 1.33 (highest versus lowest exposure) [62, 92, 93]. This statistically non-significant positive association was maintained in subgroup analyses restricted to the samples taken from serum and adipose tissue only (2/2 EE; Table 2; data not plotted; Suppl File 2.10) [93]. The remaining main and subgroup analyses assessed exposure to 17 individual PCB congeners (Table 2; Figure 11) [94]. A significant increased risk of breast cancer was reported with exposure to PCB 187 (Group I), OR 1.18, 95%CI 1.01 to 1.39, PCB 105 (Group II), OR 2.22, 95%CI 1.18 to 4.17, PCB 99 (Group III), OR 1.36, 95%CI 1.02 to 1.80, and PCB 183 (Group III), OR 1.56, 95%CI 1.25 to 1.95 (Figure 11; Suppl File 2.10) [94]. Small, statistically non-significant increases were observed for most of the remaining congeners investigated (10/13 EE; Group I 1/2 EE; Group II 6/8 EE; Group III 2/2 EE; Figure 10; Suppl File 2.11) [94].

#### Non-Hodgkin’s lymphoma (NHL)

Fourteen meta-analyses, including main and subgroup analyses, informed the association between flame-retardant exposure and risk of NHL in the general population. A significant increased risk of NHL with exposure to total PCBs was reported in the two available main analyses, OR range of 1.4 to 1.5 (Figure 11) [92, 95]. Five individual PCB congeners were assessed in two reviews; both reviews reported increased risk estimates for NHL with exposure to Group III PCBs 153, RR/OR range of 1.1 to 1.5 (2/2 EE) and PCB 180, RR/OR range of 1.07 to 1.4 (2/2 EE; Figure 11; Suppl File 2.10) [92, 96], which was found to be statistically significant in one (Figure 11; Suppl File 2.10) [92]. Results were equivocal for the remaining congeners, with one review reporting statistically non-significant increases for PCB 118 and 138 (2/2 EE; Group II; OR range of 1.08 to 1.32; Figure 11; Suppl File 2.10) [92] and the other, non-significant decreased risk estimates for these same congeners and also PCB 170 (3/3 EE; Group II; Figure 11; Suppl File 2.10) [96]. Of three subgroup analyses investigating subtypes of NHL, one estimate corresponded to a significant protective effect for chronic lymphocytic leukemia (CLL) with exposure to total PCBs, RR 0.63, 95%CI 0.39 to 0.87 (Figure 11) [96]. A reduction in risk of diffuse large B-cell lymphoma (DLBCL), though non-significant, was also reported, whereas a non-significant positive association was observed for follicular lymphoma (FL) with exposure to PCBs (Figure 11; Suppl File 2.10) [96].

#### Cancer mortality

Fourteen meta-analyses informed the association between flame-retardant exposure and risk of cancer mortality in adults, with the majority reported according to gender. The majority of analyses were provided by a pooled analysis assessing two cohorts with high incident exposure from poisoning [83]. A significant association with mortality attributable to cancer and exposure to PCBs was reported for all cancer mortality in males, SMR 1.3, 95%CI 1.1 to 1.6, liver cancer mortality in females, SMR 2.0, 95%CI 1.1 to 3.6, lung cancer mortality in both males and females, SMR 1.5, 95%CI 1.1 to 2.1 and also lung cancer mortality among males only, SMR 1.2, 95%CI 1.2 to 2.3 (Figure 11) [83]. Increased risk of malignant melanoma mortality in males and females was also significant, SMR 1.32, 95%CI 1.05 to 1.64 (Figure 11) [95]. No significant risk in cancer mortality was observed with PCB exposure by poisoning in eight other meta-analyses; however, a trend to increased risk of mortality from breast cancer and uterine cancer in women, leukaemia and pancreatic cancer was reported (4/8 EE). Conversely, mortality in women attributable to all cancers, lung cancer and stomach cancer, decreased with PCB poisoning, though not significantly. No change was observed in rectal cancer mortality in females (Figure 11; Suppl File 2.10) [83]. A non-significant decreased risk in NHL mortality was observed in workers occupationally exposed to PCBs, SMR 0.94, 95%CI 0.73 to 1.23 (Figure 11) [95].

### Other outcomes

**Figure 12:**
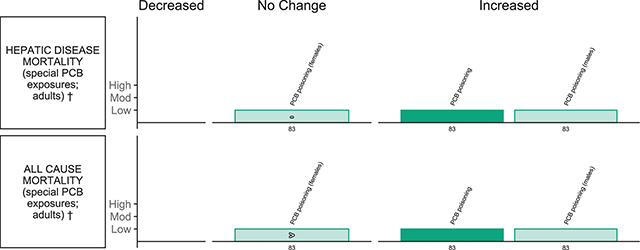
Harvest plot of exposure to plastic-associated chemicals and other outcomes. Plastic-associated chemicals included are flame retardants (green), including polychlorinated biphenyls (PCB) in populations exposed to contaminated food. All outcomes are dichotomous (†). Outcomes measured include mortality attributable to hepatic disease and all-cause mortality. Each bar represents an individual effect estimate from the corresponding review, which is indicated by the number below each bar. The height of the bar represents the quality score of the review assessed using the AMSTAR tool. Low quality reflects a score of 1–4 and moderate quality a score of 5–8. Dark filled bars represent the primary analyses of each review; light filled bars represent sub-group analyses. Bars have been assigned as an increase or decrease (columns) in the measure where the change is statistically significant. Remaining bars appearing under ‘no change’ indicate direction of effect as an increase (>), no change (–) (the relative estimate was 1), or decrease (<) in the measure or risk estimate.

Two additional mortality outcomes, hepatic disease mortality and all-cause mortality, were reported in one pooled analysis, each in relation to flame retardants following poisoning incidents in two cohorts (Table 2) [83]. Evidence suggests an association with increased risk of death attributable to hepatic disease and increased death from all causes in adults exposed to flame retardants (Figure 12).

The pooled analysis investigating hepatic disease mortality and all-cause mortality with PCB exposure scored 4/11, low quality, with the AMSTAR tool (Table 2; Suppl file 1.6). The pooled analysis provided clear indication of duplicate data management, in which cohorts were included and their details. Appropriate statistical analysis was performed.

#### Hepatic disease mortality

A statistically significant increase in mortality attributable to hepatic disease with PCB exposure was reported in a main analysis of males and females with SMR 1.5, 95%CI 1.0-2.4, and in the subgroup of males only, SMR 1.9, 95%CI 1.3 to 2.8; however, not in females, SMR 1.0, 95%CI 0.5–1.9 (Figure 8; Suppl File 2.7) [83].

#### All-cause mortality

A statistically significant increase in mortality with PCB exposure was reported in a main analysis of with SMR 1.1, 95%CI 1.1 to 1.2, and in the subgroup of males only, SMR 1.2, 95%CI 1.1 to 1.3, but not in females, SMR 1.1, 95%CI 0.9 to 1.2 (Figure 12; Suppl File 2.11) [83].

## Discussion

## Associations between plastic-associated chemical exposure and health outcomes

For each of the chemical groups for which meta-analytical data were retrieved (i.e., BPA, phthalate plasticisers, PCBs and PBDEs flame retardants, as well as some PFAS), significant association was established for at least one adverse human health outcome. Despite a multitude of chemicals that are used to make plastic [5, 21, 22], and an exponential increase in plastic production [1], there are limited epidemiological data meta-analysed to evaluate the safety of these chemicals in humans. For our search period, we found only a total of 62 systematic reviews with meta-analyses meeting our initial eligibility criteria, with 10 of these excluded due to unit of analysis errors [43].We report on 759 meta-analyses related to a range of health outcomes.

Direction of association is not anticipated to be consistent across different chemical classes captured within this umbrella review, but consistency of findings within a chemical class provides additional evidence of association reflecting an underling biological causal pathway. Considering the regulatory implications of this work, action would be at a chemical level, not a health-outcome specific level. Here, we therefore reframe and summarise the key findings aggregated by chemical class.

BPA exposure was found to be significantly associated with adverse child reproductive, endocrine, nutritional and circulatory outcomes. This is seen in the anoclitoral distance in girls with prenatal exposure, T2D in adults, insulin resistance measured as HOMA-IR in both adults and children, PCOS in women, increased risk of obesity (separately established in both children and adults), elevated waist circumference and overweight status in adults (with a consistent trend in children for each), hypertension (on evaluation of children and adults combined) and CVD.

Exposure to phthalates is significantly associated with adverse birth, child reproductive, endocrine, child neurodevelopment and circulatory outcomes. This presents as SPL in pregnant women (with strongest evidence for DnBP and DEHP, but with consistent trends for all others meta-analysed), decreased AGD in newborn boys with prenatal exposure (specifically evaluated for DEHP exposure) and insulin resistance measured as HOMA-IR in both adults and children (with strongest evidence for diisobutyl phthalate [DiBP], butyl benzyl phthalate [BBP] and DEHP, but with consistent trends for all others meta-analysed and association established for total phthalate exposure). Furthermore, certain phthalates are associated with decreased birth weight of newborns with prenatal exposure (diethyl phthalate [DEP]), T2D in adults (DiBP), precocious puberty in adolescent girls (DEHP), a number of measures of reduced sperm quality in men (DnBP and BBP with decreased sperm concentration, DnBP and DEHP with decreased sperm velocity, DEP and BBP with DNA damage as measured by increased CE and TDM) and endometriosis in women (DEHP when measured as its metabolite MEHHP and with similar trends for all other DEHP metabolites meta-analysed). However, for these, association for individual phthalates was not established across all other phthalates evaluated and does not therefore establish adverse associations for phthalates as a group. In addition, associations were seen for decreased fine motor and psychomotor development after prenatal exposure, and increased SBP in children following postnatal exposure. There were additional concerning findings for child neurodevelopment, nutritional and respiratory outcomes. These presented as decreased cognitive development and IQ loss in children, with strong evidence for postnatal exposure to DEHP, but inconsistent findings for prenatal exposure to MEP specifically. Additionally, certain phthalates (BBP) are associated with asthma, but lacking consistent trends for other individual phthalates, preventing any conclusions on phthalates as a group. A recent narrative review on phthalates and allergic diseases such as asthma and rhinoconjunctivitis, also reports a reason for concern [97]. Furthermore, a consistent trend of association with CVD is also found for all phthalates evaluated, although no individual finding was statistically significant.

PCBs, PBDEs and PFAS are each significantly associated with adverse birth outcomes, which is seen in the decreased birth weight of newborns with prenatal exposure, and additionally with decreased birth length for PFAS. PCBs also show significant associations with adverse adult reproductive and endocrine outcomes. This is reflected in T2D in adults and endometriosis in women. Of concern within endocrine outcomes, exposure to PBDEs and certain PFAS are also associated with changes in measures of thyroid function (increased TT4 for high exposure to PBDEs, increased fT4 for PFOS and decreased TT4 for PFHxS). However, similar association was not established for lower exposure to PBDEs, or for other PFAS, and we cannot draw conclusions of adverse associations for PDBEs and PFAS as a group. For PCB exposure, significant associations were also found for adverse circulatory, respiratory, cancer and other outcomes. This is due to increased CVD and hypertension after PCB exposure, mortality from CVD after PCB poisoning as well as bronchitis in infants following prenatal exposure. Additionally, there were significant associations for multiple types of cancer in the general population and cancer mortality in special risk populations (i.e., occupationally exposed or poisoning). Lastly, PCB poisoning was significantly associated with mortality from hepatic disease in males, and from all-cause mortality for men and women combined. While not significant, there is also a trend for increased hypertension mortality after PCB poisoning. It is important to note that many of these studies are based on PCB poisoning which occurred through the ingestion of contaminated rice bran that had been contaminated during processing [83, 92]. While exposure in these circumstances was at high levels and not directly through use in plastics, studies of special exposure populations give highly relevant information on potential health impacts of chemicals at higher exposure and complement separate findings of studies in the general population. PBDE exposure is significantly associated with adverse child neurodevelopment outcomes, seen as reduced children’s cognitive development and IQ loss after prenatal exposure to BDE-47. Furthermore, exposure to PFAS is significantly associated with adverse nutritional outcomes. This is reflected in the increased risk for overweight status after prenatal exposure and BMI after pre- or postnatal exposure in children. While there was a significant increase of allergic rhinitis after PFOA exposure, this association was not seen for other PFAS analysed. Furthermore, while prenatal exposure to PFOA was associated with ADHD in girls, inconsistent findings were reported for boys, as well as for exposure to PFOS.

There was evidence for only three protective effects. One was seen for associations between phthalate exposure and timing of puberty. However, while the abnormal timing of puberty was less common in boys with higher phthalate exposure, adverse associations were found in girls for this class of chemicals. Higher DEHP exposure when measured as its metabolite MEOHP is associated with increased (beneficial) HDL levels. However, there was an inconsistent trend in the opposite direction when measured as either of two other DEHP metabolites (MEHHP or MEHP). Additionally, increased PCB exposure is associated with reduced incidence of chronic lymphocytic leukemia, a subtype of NHL, but there was and increased incidence of NHL as a whole. Therefore, none of these specific exposure outcome associations provide reassurance regarding safety.

## Chemicals identified

Due to the hierarchal relationship between of primary publications and systematic reviews, considering both volume and timing, it is not surprising that this umbrella review captured a narrow range of chemicals that are, or were, common high production volume plastic-associated chemicals and have been suspected to be harmful to human health for some time, namely BPA, phthalates, PCBs, PBDEs, and PFAS. BPA is primarily (95%) used in the production of polycarbonate plastics and polymer resins [98]. Similarly, ortho-phthalate diesters comprise 85% of the total plasticiser market; as a specific example, ~97% of DEHP is used as a plasticizer, with the remainder being predominantly used as solvents [99]. PCB flame retardants had an application in plastics alongside their major application in electrical capacitors and heat exchangers [100–102]. They are still present in modern recycled plastics as legacy chemicals [103] and are included in key comprehensive lists of plastic-associated chemicals [5, 21, 22]. PBDEs were used in substantial quantities in the manufacture of plastic components of electronic devices and in polyurethane furniture [104]. PFAS are a large family of chemicals with applications including protective coatings for food packaging, textiles and furniture, and the production of fluoropolymers used in non-stick cookware and waterproof fabrics [77, 105]. PFAS may also form during surface fluorination of plastic packaging containers [106, 107].

In addition, there is a paucity of systematic and meta-analysed data for the plastic-associated chemicals that are replacing those that have been shown to be harmful to human health but are increasing in production volumes. For example, due to health concerns [108] and concomitant regulation [109, 110], BPA is being replaced by bisphenol analogues such as Bisphenol F and Bisphenol S despite emerging concerns about their safety [111]. Such replacement, or “regrettable substitution” [112] is similarly occurring for PDBEs with replacement by OPEs [113], and for phthalates with phthalate substitutes [99]. Furthermore, there is a gap in the primary literature on micro- and nanoplastic exposure and human health outcomes [25, 114], which explains the absence of systematic reviews and meta-analyses.

## Comparison with other studies

To our knowledge, this study is first to investigate the complete, high-level, evidence for human health effects of plastics and plastic-associated chemicals across a broad range of plastic chemical groups. However, there have been other overviews with narrower focus or alternative systematic methodologies.

Eales et al. [26] recently presented a structured overview of human health effects of phthalate plasticisers. That review was narrower in scope than our umbrella review in terms of the breadth of plastic-associated chemicals considered, but broader in including narrative reviews. Allowing for this, findings are highly consistent. As with our umbrella review, the authors find a consistent pattern of association between prenatal phthalate exposure and decreased AGD in boys, and moderate evidence for an association between phthalate exposure and low birth weight, endometriosis and T2D. Somewhat stronger findings for abnormal sperm-quality measures (evaluated to meet their criteria for robust evidence) are likely to reflect the conclusions of several reviews without meta-analysis and therefore omitted in our umbrella review, including in particular a high-quality review by Radke et al. [113] applying the United States Environmental Protection Agency’s Integrated Risk Information System (IRIS) systematic review methods. Findings of some evidence of associations with decreased gestational age at birth (prematurity), changes in sex hormones, decreased anofourchette distance in girls, and lower antral follicle count in women are similarly based on reviews without meta-analysis excluded in our umbrella review. Whilst omitted from our umbrella review by design, these narrative findings must not be dismissed; in the absence of meta-analysis, they may reflect the best available evidence for these exposure-outcome combinations. Furthermore, Eales et al. included a meta-analysis of association with anofourchette distance [114] that we excluded here due to unit of analysis errors [43]. Conversely, we additionally report association between phthalate exposure and SPL evaluated in a 2020 review [48], which may have been published after the search undertaken by Eales et al. [26]. Our findings for an association between phthalate exposure and precocious puberty similarly reflect two additional systematic reviews that were published in 2020 [55, 56].

Consistent with our umbrella review, Eales et al. [26] also find robust association between postnatal phthalate exposure and adverse child cognitive development / lower IQ, robust association between exposure to the phthalate BBP and childhood asthma and mixed evidence of association with obesity, BMI and waist circumference, which was strongest for DEHP and adult obesity. Stronger conclusions from Eales et al. [26] in relation to prenatal exposure are likely to reflect the findings of an additional review without meta-analysis that was omitted in our umbrella review [115]. A finding of moderate evidence of association of phthalate exposure with ADHD, and some evidence of association with autism spectrum disorder (ASD), are similarly based on reviews without meta-analysis excluded here due to unit analysis errors [116, 117]. Eales et al. [26] similarly finds some evidence of association with atopic dermatitis [118] and, in addition, hearing disorders [119] and markers of oxidative stress [120]. A finding of moderate evidence of association with breast cancer is based on a study excluded here due to the statistical approach in the meta-analyses [121]. Whilst omitted from our umbrella review by design, findings without meta-analysis must not be dismissed since they may reflect the best available evidence for these exposure-outcome combinations. Conversely, we additionally report association between phthalate exposure, specifically DEHP, and increased SBP based on the 2019 meta-analysis by Golestanzadeh et al. [54], the details of which were in supplementary material that may not have been reviewed by Eales et al. [26], although Golestanzadeh et al. [54] do also confirm their findings of association between various phthalate and increased blood pressure in the main body of their publication.

Similarly, Lin et al. [27] recently published an umbrella review of human health outcomes of BPA exposure. That umbrella review is confined to a single plastic-associated chemical, BPA, selects only the most recently published meta-analysis for each exposure-outcome association, in contrast to the vote-counting approach here, and is based on a search strategy which extended to mid 2023. Allowing for these differences, the authors findings are highly consistent with those presented here. With respect to birth outcomes, a finding of significant association with preterm delivery and reduced gestational age at birth is based on a meta-analysis published outside our search dates [122]. On the one hand, a review the year prior, presented here, had found a trend in that direction which was not statistically significant [52]. On the other hand, with respect to child reproductive outcomes, AGD in girls is omitted as an outcome by Lin et al. [27], where a statistically significant association was found by Nelson et al. [59]. The reason for this omission by the authors is unclear as Nelson et al. [59] is referenced elsewhere in their review. Finally, two additional endocrine outcomes are evaluated within very recently published meta-analyses captured by Lin et al. [27]. Although beyond the range of our study, we note that there were no new statistically significant findings for either gestational diabetes or neonatal thyroid hormones. Other differences similarly do not impact our key findings. While Lin et al. [27] do not include findings for insulin resistance, fasting insulin and glucose, they do report on the clinical outcomes of T2D with the same findings as ours with statistically significant association on meta-analysis. A meta-analysis of BPA exposure and endometriosis [64], reported here, is also omitted by Lin et al. [27], but again with no significant association with BPA exposure. Study quality is assessed against AMSTAR 2 by Lin et al. [27], which is similar to our preferred measure of the original AMSTAR tool, but details of scoring are not presented to allow for any comparison.

In addition, statistically significant adverse associations that we find in this umbrella review are replicated in the findings of Lin et al., including evidence of association between exposure to BPA and each of obesity, overweight status, increased wait circumference, CVD and hypertension. An additional finding by Lin et al. [27] of association between BPA and decreased HDL is reported based on a meta-analysis by Fu et al. [87]. No statistically significant findings are reported for any other lipid parameters, consistent with our own findings. While Fu et al. [87] do report finding correlation between BPA and lower HDL, details of that meta-analysis are not available in either the paper or associated supplementary material. Our findings for lipid parameters are instead based on an analysis by Dunder et al. [84] the previous year, who do not find statistically significant association with HDL. There are no other differences in findings with respect to other papers within the scope of both umbrella reviews, although there are a number of additional findings by Lin et al. [27] based on very recently published meta-analyses beyond the scope of this study, notably including statistically significant adverse associations with allergic respiratory and skin disease, immunological and renal parameters [123, 124].

## Strengths of the umbrella review

Strengths of our review’s eligibility criteria and search strategy include searches across two databases, including a major database indexing systematic reviews. In addition, given the large number of plastics and plastic-associated chemicals, we evaluated a broad scope of common polymers and high-volume plasticisers, flame retardants, bisphenols and PFAS, against all outcomes reported—an approach not undertaken to date. Moreover, we present our findings in qualitative harvest plots, complemented by the quantitative effect size estimates in the narrative of the results section, and supplementary material. This provides the audience with the full picture of the evidence base covered. Additional strengths include the combination of experimental and epidemiological evidence, the assessment of methodological quality against objective criteria (AMSTAR), and concomitant evaluation of statistical methodology for possible unit of analysis errors, which we found to be prevalent and were excluded.

## Limitations of the umbrella review

### Source systematic reviews

The overall scope of our findings is limited by the availability of meta-analyses, reflecting, but not accounted for, by gaps in availability of primary research [25]. In addition, there are a number of limitations in the source systematic reviews.

First, methodological and statistical issues led to the exclusion of 10 systematic reviews otherwise within the scope of this paper (Suppl File 1.5.2), as well as individual meta-analyses from 12 systematic reviews in which other meta-analyses were not impacted and are included (Suppl File 2). Reasons for exclusion are listed for each excluded review paper in Supplementary Information (Suppl File 1.5.2). Underlying statistical issues are explored in a separate publication [43].

Second, a number of limitations in methodology of meta-analysis or reporting of the methodology was identified against AMSTAR criteria for included meta-analyses and pooled analyses. This is reported in detail within our results. Of particular note, limitations relate to risk of bias in the primary literature itself. Although meta-analysis is a beneficial tool to combine estimates of relationships across different studies, the reliability of estimates from the included primary studies can also impact the reliability of meta-analysis. These risks can be evaluated with risk-of-bias assessments. It was notable that across the 52 studies in this umbrella review, AMSTAR scores for the 36 studies that did report use of a risk-of-bias tool (ranging from 5 to 11) tended to be higher than the score for the 16 that did not (range 2-6). It is recommended that future reviews report on risk-of-bias assessment of the primary studies included.

Overall, 28 of the meta-analyses and pooled analyses in this umbrella review were assessed to be derived from reviews of high quality (AMSTAR score 9–11), 595 of moderate quality (AMSTAR 5–8), and 136 from reviews of low quality (AMSTAR <5) and, from the available information presented, should be interpreted with some caution. That said, all exposure-outcome associations evaluated within a review assessed to be of low quality by their AMSTAR rating were separately evaluated in at least one other review of moderate quality or above, with the exception of associations between in utero PCB exposure and birth weight and sex ratio. Reassuringly, in all cases where the exposure-outcome combination had been separately evaluated in a review assessed to be of moderate or high quality, the findings were consistent with that in the review assessed to be of low quality. Specifically, meta-analyses in other reviews confirm association between total PCB exposure and both breast cancer and NHL. However, additional meta-analyses were not found for a number of other exposure-outcome combinations: association between PCB poisoning and mortality statistics; risk of ADHD with PFAS exposure [77]; lipid levels with BPA exposure [84] and bronchitis and wheeze in children following PCB exposure [91]. Without further detail on methodology, these associations should be treated with caution but not dismissed; lower AMSTAR scores indicate only risk of bias, not that bias is present, and in the absence of additional meta-analyses these lower-scoring reviews may reflect the best available evidence synthesis for these exposure-outcome combinations.

Conflict of interest (COI) is a known source for risk of bias. Using AMSTAR, we were only able to evaluate COI of the included studies, of which the majority declared no COI (n = 48) and only a few did not report on COI (n = 4). In addition, evaluation at the systematic review level as we did here, precluded assessing whether there was any COI in the primary literature underpinning the findings of our included studies. As such, COI is another aspect where there is a potential risk of bias that we could not explore, but which does not indicate that bias was present.

### Process

Beyond limitations in the source systematic reviews, there are a number of limitations in the process employed in this umbrella review.

First, we selected outcomes for which data had been meta-analysed. Meta-analysis has some distinct advantages when considering an evidence base: it can increase the statistical power of the analysis, increase the generalisability of the results overall and increase the confidence in the results where marked heterogeneity is absent (be it methodological, clinical or statistical). However, meta-analysis is not always the most suitable approach for synthesising evidence. Our approach omitted a large number of reviews, including narrative, and their included studies that were either too heterogenous to combine statistically, or where only one study was identified to inform the outcome. Second, our method of synthesis used vote counting, harvest plots and narrative. With this method, the direction of effect and its significance is readily accommodated whereas the effect size, number of included studies, sample size and heterogeneity are not. However, we have provided this information in the supplementary materials (Suppl File 2). Additionally, the source review literature generally does not cover detailed findings such as dose-response modeling, disease burden, or effects of all covariates. As these detailed findings need a study designed specifically to address them, primary research is better suited than systematic or umbrella reviews.

Second, we used the original AMSTAR tool to assess the quality of all included reviews, including the pooled analyses of large cohorts of participants [50, 68]. Despite AMSTAR 2 being designed to better accommodate reviews with inclusion of non-randomised studies, as mentioned (see Methods section), AMSTAR has performed adequately for review of observational research, is faster to complete and has high inter-rater reliability and hence was selected [39, 40]. Furthermore, pooled analysis methodology does not necessarily include specific criteria for undertaking systematic reviews, such as a comprehensive search and screening of the literature. Therefore, the small subset of pooled analyses that met the inclusion criteria for this umbrella review expectedly scored low in the quality appraisal with AMSTAR. In this umbrella review, a low-scoring pooled analysis is different from a low-scoring systematic review with meta-analysis.

## Frameworks for assessing epidemiological studies

Formal frameworks have been developed for the systematic evaluation of epidemiological toxicology data. These include the United States National Toxicology Program’s Office of Health Assessment and Translation (OHAT) [125, 126], IRIS [113, 127] and the Navigation Guide Systematic Review Methodology [23]. These frameworks provide guidance on all steps in the conduct of systematic reviews of observational studies of aetiology: problem formulation and shaping the research question, defining exposure and outcomes, literature search strategy, evaluating risk of bias, planning the statistical analyses and translation of findings. Frameworks such as these were rarely used across the reviews identified in this umbrella review, with just five reviews following one of the three frameworks above [44, 58, 61, 74, 76].

There was more widespread use of reporting guidelines such as PRISMA and Meta-analysis of Observational Studies in Epidemiology (MOOSE) [128] (the former recently revised [35]) which were used in an additional 20 included reviews. Notably, all 5 reviews which had followed a formal framework were evaluated to score 8 or higher on AMSTAR (range 8–11) and all had assessed risk of bias in the primary literature. The 20 reviews which had followed a reporting guideline inconsistently included a risk-of-bias assessment and were typically assessed to be of lower quality on AMSTAR (with score ranging from 4 to 9) and the 27 reviews that followed neither a formal framework nor reporting guideline were even more variable in quality (with score ranging from 2 to 9). The uptake of frameworks such as OHAT, IRIS and the Navigation Guide is recommended for reviewers interested in meta-analysis of plastic-associated chemical exposure on human health.

## Future evidence synthesis

This umbrella review reports only on eligible systematic reviews published up until August 2020. A subsequent search until August 2023 identified a further 76 potentially eligible systematic reviews, reflecting an exponential increase in the primary literature and its subsequent quantitative synthesis by systematic review with meta-analysis (Suppl File 1.7). Although the current umbrella review represents findings only up to August 2020, those findings nevertheless demonstrate associations between exposure and a wide range of adverse health outcomes. Of note, 74 of the subsequent systematic reviews covered the same breadth of human health outcomes as in our umbrella review, with only 2 examining a different domain, namely ‘immunology’. Inevitably, further synthesis will be required to evaluate this burgeoning literature on the same chemical classes quantitatively, on these and other health outcomes.

In addition, to address the gaps in terms of the chemicals evaluated, we recommend that the focus is shifted to include emerging plastic-associated chemicals of concerns such as substitutes for bisphenols, phthalates and flames retardants [98, 110–112], as well as other classes of plastic-associated chemicals with likely high human exposure risk such as UV-stabilisers (bensophenones, benzotroazoles), antioxidants (e.g., hindered phenol antioxidants, nonylphenols) and heat stabilisers (e.g., organotins) [3].

## Conclusion

We are exposed to plastic during everyday life via food packaging, construction materials, household goods, and transport as well as via environmental pollution of air, water, land and soil [3]. Our umbrella review summarises available evidence of adverse human health effects of plastic-associated chemicals in consumer products. We find that each chemical group with available meta-analysis or pooled-analysis data is associated with at least one adverse human health impact within the broad categories of birth, child and adult reproductive and endocrine, child neurodevelopment, nutritional, circulatory, respiratory, skin-related disorders and cancer outcomes. We also find significant gaps in the literature, considering there are over 16,000 chemicals used in plastics [5, 21, 22] Our findings have implications for the unknown safety of multiple plastic-associated chemicals which lack evaluation in humans.

Key priority areas without available data include the health effects of micro- and nanoplastics, bisphenol analogues, non-phthalate plasticisers and the alternative halogenated and organophosphate flame retardants that have replaced PBDEs. The critical importance of such post-market surveillance to regulation of chemicals is underscored by the high-volume plastic-associated chemicals evaluated in this umbrella review. Indeed, the findings of this umbrella review exemplify the principle that safety cannot be assumed at the point of entry of a chemical to market, without process to systematically monitor for and identify post-market toxicity.

## Additional Files

The additional files for this article can be found as follows:

10.5334/aogh.4459.s1 Supplementary File 1. Glossary and abbreviations.

10.5334/aogh.4459.s1 Supplementary File 2. Characteristics of included reviews.

## Data Availability

All extracted data from included reviews are available in Suppl File 2. Excel format data are also available upon request.
