# Supplementary material for: An Umbrella Review of Meta-Analyses Evaluating Associations between Human Health and Exposure to Major Classes of Plastic-Associated Chemicals
Source: Ann Glob Health. 2024 Aug 19;90(1):52. doi: 10.5334/aogh.4459 (PMC11342836; doi:10.5334/aogh.4459)
Supplement: Supplementary File 1. — Glossary and abbreviations. [file agh-90-1-4459-s1.pdf]

Supplementary file 1

1.1 Glossary and abbreviations

1.1.1 Glossary

For details, see Cochrane Handbook for Systematic Reviews of Interventions (Higgins et al. 2022)

**Effect size**	A quantitative measure of the magnitude of the effect. In this instance, the larger the effect size, the stronger the relationship between the exposure to a plastic-associated chemical/s and a health outcome.
**β-coefficient**	The degree of change in the outcome variable for every 1-unit of change in the predictor variable, in this case, the plastic-associated chemical.
**z-transformed β-coefficient**	Transformation of the sampling distribution of the correlation coefficient so that it becomes normally distributed.
**Fixed effects model**	Fixed effects model assumes that the true effect observed is the same in every study and any differences are solely due to random sampling error (chance) within each study. The results can be considered a typical effect.
**Random effects model**	Random effects model assumes that observed effects follow a distribution (usually normal) and differences are due to both random sampling error (chance) within studies and also variation between studies. The result can be considered an average effect.
**Fixed variables**	Variables that can be measured without error. It is assumed that a fixed variable in one study will have the same value as a fixed variable in another study (e.g. spontaneous pregnancy loss).
**General population**	A study sample or population that is representative of general population exposure to the chemical of interest (c.f. a special exposure group or a special risk group for exposure). Including both adults and children unless otherwise specified.
**Meta-analysis**	A statistical technique used to combine the numerical results of 2 or more separate studies of the same research question, in order to derive a single overall estimate.
**Odds ratio**	A quantitative measure of association between an exposure and an outcome, in this instance a health outcome. The odds ratio represents the odds that an outcome will occur given a particular exposure, compared to the odds of the outcome occurring in the absence of that exposure. Odds ratios are most commonly used in case-control studies; however, they can also be used in cross-sectional and cohort study designs. Odds ratios > 1 indicate that an exposure is harmful; < 1 indicates a protective effect.
**Pooled analysis**	A statistical technique for combining individual data (c.f. results) from 2 or more studies and re-analysing as a whole.
**Risk ratio**	A quantitative measure of association comparing the risk of a health outcome among one group with the risk among another group in this instance the groups being differentiated by exposure to plastic-associated chemicals. Calculated by dividing the risk in one group by the

	risk in the comparison group. A risk ratio of 1 indicated identical risk in both groups. A risk ratio > 1 indicates and increased risk, usually in the more highly exposed group; a risk ratio <1 indicates a decreased risk.
†	Symbol used to indicate a dichotomous health outcome.
‡	Symbol used to indicate a continuous health outcome.

1.1.2 Abbreviations

Chemicals

Bisphenols

**BPA**	Bisphenol A

Phthalates and metabolites

**ΣDEHP**	Sum of all measured DEHP metabolites
**BBP**	Butyl benzyl phthalate
**DEP**	Diethyl phthalate
**DEHP**	Di (2-ethylhexyl) phthalate
**DEHP met.**	Di (2-ethylhexyl) phthalate metabolites
**DiBP**	Diisobutyl phthalate
**DnBP**	Di-n-butyl phthalate
**MBzP**	Monobenzyl phthalate
**MECPP**	Mono (2-ethyl-5-carboxypentyl) phthalate
**MEHP**	Mono (2-ethylhexyl) phthalate
**MEHHP**	Mono (2-ethyl-5-hydroxyhexyl) phthalate
**MEOHP**	Mono (2-ethyl-5-oxohexyl) phthalate
**MEP**	Monoethyl phthalate
**MiBP**	Monoisobutyl phthalate
**MMP**	Monomethyl phthalate
**MnBP**	Mono-n-butyl phthalate

Flame retardants

**OPE**	Organophosphate ester
**PBB**	Polybrominated biphenyl
**PBDEs**	Polybrominated diphenyl ethers
**PCBs**	Polychlorinated biphenyls (and individual PCB congeners, e.g. PCB 99, PCB 105, PCB 183, PCB 187)
**PCB classes I, II, III**	Functional classes of PCBs based on structural and biological considerations (Wolff et al. 1997)

Per- and Polyfluoroalkyl Substances

**PFAS**	Per- and Polyfluoroalkyl Substances
**PFHxS**	Perfluorohexane sulfonate
**PFNA**	Perfluorononanoic acid
**PFOA**	Perfluorooctanoic acid
**PFOS**	Perfluorooctane sulfonate

Polymers

**PVC**	Polyvinylchloride

Health outcomes

**AGD**	Anogenital distance
**CE**	Comet extent
**ADHD**	Attention deficit hyperactive disorder
**ApoB**	Apolipoprotein B
**ASD**	Autism spectrum disorder
**Bayley-III**	Bayley Scales of Infant and Toddler Development, 3rd ed
**BMI**	Body mass index
**BSID-II**	Bayley Scales of Infant Development, 2nd ed
**CBCL**	Child Behaviour Checklist
**CVD**	Cardiovascular disease
**DBP**	Diastolic blood pressure
**DLBCL**	Diffuse large B-cell lymphoma
**DSM-IV**	Diagnostic and Statistical Manual of Mental Disorder, 4th ed
**FL**	Follicular lymphoma
**FSIQ**	Full Scale IQ
**fT4**	Free thyroxine
**GCS**	General Cognitive Scale
**HDL**	High-density lipoprotein
**HOMA-IR**	Homeostatic Model Assessment for Insulin Resistance
**IQ**	Intelligence quotient
**LDL**	Low-density lipoprotein
**LIN**	Linearity
**MDI**	Mental Development Index
**MSCA**	McCarthy Scales of Children's Abilities
**NHL**	Non-Hodgkin's lymphoma
**PCOS**	Polycystic ovary syndrome
**PDI**	Psychomotor Development Index
**SBP**	Systolic blood pressure
**SDQ**	Strengths and Difficulties Questionnaire
**SPL**	Spontaneous pregnancy loss
**T2D**	Type 2 diabetes mellitus
**T3**	Triiodothyronine
**Tail%**	Percent of DNA in tail
**TC**	Total cholesterol
**TDM**	Tail distributed moment
**TG**	Triglycerides
**TSH**	Thyroid stimulating hormone
**TT4**	Total thyroxine
**VCL**	Curvilinear velocity
**VSL**	Straight line velocity
**WISC**	Wechsler Intelligence Scale for Children
**WPPSI**	Wechsler Preschool & Primary Scale of Intelligence

Other

**AMSTAR**	Assessment MeaSurement Tool for the Analysis of Systematic Reviews
**EE**	Effect estimates
**ICD-11**	International Classification of Diseases, 11th revision

**IRIS**	Integrated Risk Information System
**MD**	Mean difference
**MMT**	Million metric tonnes
**MOOSE**	Meta-analysis of Observational Studies in Epidemiology
**NIAS**	Non-intentionally added substances
**OHAT**	Office of Health Assessment and Translation
**OR**	Odds ratio
**PECO**	Population Exposure Comparator Outcome
**PRISMA**	Preferred Reporting Items for Systematic Reviews and Meta-Analyses
**RR**	Relative risk
**SMD**	Standardised mean difference
**SMR**	Standardised mortality ratios

References

Higgins, J., Thomas, J., Chandler, J., Cumpston, M., Li, T., Page, M., Welch, V. (Eds.), 2022. Cochrane Handbook for Systematic Reviews of Interventions, Version 6.3 (updated February 2022). ed. Cochrane.

Wolff M, Camann D, Gammon M, Stellman S. Proposed PCB Congener Groupings for Epidemiological Studies. *Environmental Health Perspectives*. 1997. 105(1): 13-14.

1.2 Search strategies

Epistemonikos search (26 August 2020)

**Plastics and plastic polymers**	Plastic*" OR "microplastic*" OR "polyethylene*" OR "polypropylene*" OR "polyethylene terephthalate*" OR "polystyrene*" OR "polyvinyl chloride*" OR "polycarbonate*" OR "polylactide*" OR "teflon"
**Plastic-associated chemicals**	
Bisphenols	bisphen* OR diphen* OR BPA OR 4,4'-isopropylidenediphenol OR 2,2-Bis(4-hydroxyphenyl)propane OR Diphenylolpropane OR 4,4'-(propane-2,2-diyl)diphenol
Plasticisers	"plasticiser*" OR "plasticizer*" OR "phthalate*" OR "phthalic acid" OR "orthophthal*" OR "ortho-phthal*" OR "benzene-1,2-dicarboxy" OR "benzenedicarboxy*" OR "DEHP" OR "terephthalate*" OR "adipate*" OR "sebacate*" OR "trimellitate*" OR "tricresyl*" OR "cresyldiphenyl*" OR "cyclohexanoate*" OR "dibenzoate*" OR "Acetyl tributyl citrate"
Flame retardants & PFAS	"flame retardant*" OR "fire retardant*" OR "fire proof*" OR "fireproof*" OR "polychlorinated biphenyl*" OR "PCB" OR "PCBs" OR "polychlorinated*" OR "tetradecachloro*" OR "polybrominated biphenyl*" OR "Polybrominated Diphenyl Ether*" OR "PBDE" OR "PBDEs" OR "polybrom*" OR "decabromo*" OR "hexabromocyclododecan*" OR "HBCD" OR "tetrabromobisphenol*" OR "TBBP-A" OR "TBBPA" OR "TBBP" OR "tetrabrom*" OR "organophosphate ester*" OR "triphenyl phosphate*" OR "Triphenylphosphate" OR "triphenyl ester" OR "tricresyl*" OR "trixylyl*" OR "trixylenyl*" OR "tris(2-chloroethyl)phosphate" OR "TCEP" OR "tris(chloropropyl)phosphate" OR "TCPP" OR "tris(1,3-dichloro-2-propyl)phosphate" OR "TDCPP" OR "resorcinol bis*" OR "RDP" OR "bisphenol A diphenyl phosphate" OR "BADP" OR "melamine polyphosphate*" OR "diphenylcresylphosphate*" OR "Tetrachlorophthal*" OR "fluoropolymer*" OR "Polyfluor*" OR "Perfluor*" OR "PFOA" OR "PFOS" OR "PFAS" OR "hexafluoropropylene" OR "GENX" OR "polytetrafluoroethylene" OR "PTFE"
Endocrine disrupting chemicals	"endocrine-disrupting" OR "endocrine disrupting" OR "environmental endocrine disrupting" OR "endocrine disruptor" OR "endocrine-disruptor"
Limits	NOT surgery<br><br>Systematic reviews only

PubMed search (30 September 2020)

**Plastics and plastic polymers**	"plastics"[mh:noexp] OR plastic*[tiab] OR "microplastics"[mh] OR microplastic*[tiab] OR "polyethylenes"[mh] OR polyethylene*[tiab] OR "polypropylenes"[mh] OR polypropylene*[tiab] OR "polystyrenes"[mh] OR polystyrene*[tiab] OR "polyvinyl chloride"[mh] OR polyvinyl chloride*[tiab] OR polycarbonate*[tiab] OR teflon[tiab] OR nylon[tiab] OR "plasticizers"[mh] OR "flame retardants"[mh] OR "endocrine disruptors"[mh] OR endocrine disrupt*[tiab]
**Plastic-associated chemicals**	
Bisphenols	bisphen*[tiab] OR diphen*[tiab] OR BPA[tiab] OR 4,4'-isopropylidenediphenol[tiab] OR "2,2-Bis(4-hydroxyphenyl)propane"[tiab] OR Diphenylolpropane[tiab] OR "4,4'-(propane-2,2-diyl)diphenol"[tiab]
Plasticisers	plasticiser*[tiab] OR plasticizer*[tiab] OR phthalate*[tiab] OR phthalic acid[tiab] OR orthophthal*[tiab] OR ortho-phthal*[tiab] OR "benzene-1,2-dicarboxy"[tiab] OR benzenedicarboxy*[tiab] OR DEHP[tiab] OR terephthalate*[tiab] OR adipate*[tiab] OR sebacate*[tiab] OR trimellitate*[tiab] OR tricresyl*[tiab] OR cresyldiphenyl*[tiab] OR cyclohexanoate*[tiab] OR dibenzoate*[tiab] OR Acetyl tributyl citrate[tiab]
Flame retardants & PFAS	flame retardant*[tiab] OR fire retardant*[tiab] OR fireproof[tiab] OR polychlorinated biphenyl*[tiab] OR PCBs[tiab] OR polychlorinated biphenyl*[tiab] OR tetradecachloro*[tiab] OR "polybrominated biphenyl"[tiab] OR polybrominated biphenyl*[tiab] OR Polybrominated Diphenyl Ether*[tiab] OR PBDEs[tiab] OR polybrom*[tiab] OR decabromo*[tiab] OR hexabromocyclododecan*[tiab] OR "HBCD"[tiab] OR tetrabromobisphenol*[tiab] OR TBBPA[tiab] OR TBBP[tiab] OR tetrabrom*[tiab] OR organophosphate ester*[tiab] OR triphenyl phosphate*[tiab] OR Triphenylphosphate*[tiab] OR triphenyl ester*[tiab] OR "tricresyl"[tiab] OR trixylyl*[tiab] OR trixylenyl*[tiab] OR tris(2-chloroethyl)phosphate[tiab] OR TCEP[tiab] OR tris(chloropropyl)phosphate[tiab] OR TCPP[tiab] OR tris(1,3-dichloro-2-propyl)phosphate*[tiab] OR TDCPP[tiab] OR resorcinol bis*[tiab] OR RDP[tiab] OR bisphenol A diphenyl phosphate[tiab] OR BADP[tiab] OR melamine polyphosphate*[tiab] OR diphenylcresylphosphate*[tiab] OR Tetrachlorophthal*[tiab] OR fluoropolymer*[tiab] OR Polyfluor*[tiab] OR Perfluor*[tiab] OR PFOA[tiab] OR PFOS[tiab] OR PFS[tiab] OR hexafluoropropylene*[tiab] OR GENX[tiab] OR polytetrafluoroethylene*[tiab] OR PTFE[tiab]
Systematic review/ meta-analysis	AND (Systematic review [sb] OR meta-analysis)
Limits	NOT surgery

1.3 AMSTAR – A measurement tool to assess the methodological quality of systematic reviews and decision rules

1. Was an 'a priori' design provided?
The research question and inclusion criteria should be established before the conduct of the review.

Note: Need to refer to a protocol, ethics approval, or pre-determined/a priori published research objectives to score a "yes."

□ Yes
□ No
□ Can't answer
□ Not applicable

---

2. Was there duplicate study selection and data extraction?
There should be at least two independent data extractors and a consensus procedure for disagreements should be in place.

Note: 2 people do study selection, 2 people do data extraction, consensus process or one person checks the other's work.

Reviewers' decision notes: This question was assigned a YES if both study selection and data extraction were conducted. However, if only one was fulfilled, then Q2 was assigned a CAN'T ANSWER

□ Yes
□ No
□ Can't answer
□ Not applicable

---

3. Was a comprehensive literature search performed?
At least two electronic sources should be searched. The report must include years and databases used (e.g., Central, EMBASE, and MEDLINE). Key words and/or MESH terms must be stated and where feasible the search strategy should be provided. All searches should be supplemented by consulting current contents, reviews, textbooks, specialized registers, or experts in the particular field of study, and by reviewing the references in the studies found.

Note: If at least 2 sources + one supplementary strategy used, select "yes" (Cochrane register/Central counts as 2 sources; a grey literature search counts as supplementary).

Reviewers' decision notes: This question was assigned a YES, if there were at least two databases searched and one supplementary search strategy used (i.e., reference checking, contacting experts in the field of study, grey literature search).

□ Yes
□ No
□ Can't answer
□ Not applicable

---

4. Was the status of publication (i.e. grey literature) used as an inclusion criterion?
The authors should state that they searched for reports regardless of their publication type. The authors should state whether or not they excluded any reports (from the systematic review), based on their publication status, language etc.

□ Yes
□ No
□ Can't answer
□ Not applicable

Note: If review indicates that there was a search for "grey literature" or "unpublished literature," indicate "yes." SIGLE database, dissertations, conference proceedings, and trial registries are all considered grey for this purpose. If searching a source that contains both grey and non-grey, must specify that they were searching for grey/unpublished lit.

---

5. Was a list of studies (included and excluded) provided?
A list of included and excluded studies should be provided.

Note: Acceptable if the excluded studies are referenced. If there is an electronic link to the list but the link is dead, select "no."

□ Yes
□ No
□ Can't answer
□ Not applicable

---

6. Were the characteristics of the included studies provided?
In an aggregated form such as a table, data from the original studies should be provided on the participants, interventions and outcomes. The ranges of characteristics in all the studies analyzed e.g., age, race, sex, relevant socioeconomic data, disease status, duration, severity, or other diseases should be reported.

Note: Acceptable if not in table format as long as they are described as above.

□ Yes
□ No
□ Can't answer
□ Not applicable

---

7. Was the scientific quality of the included studies assessed and documented?
'A priori' methods of assessment should be provided (e.g., for effectiveness studies if the author(s) chose to include only randomized, double-blind, placebo controlled studies, or allocation concealment as inclusion criteria); for other types of studies alternative items will be relevant.

Note: Can include use of a quality scoring tool or checklist, e.g., Jadad scale, risk of bias, sensitivity analysis, etc., or a description of quality items, with some kind of result for EACH study ("low" or "high" is fine, as long as it is clear which studies scored "low" and which scored "high"; a summary score/range for all studies is not acceptable).

□ Yes
□ No
□ Can't answer
□ Not applicable

---

8. Was the scientific quality of the included studies used appropriately in formulating conclusions?
The results of the methodological rigor and scientific quality should be considered in the analysis and the conclusions of the review, and explicitly stated in formulating recommendations.

□ Yes
□ No
□ Can't answer
□ Not applicable

Note: Might say something such as "the results should be interpreted with caution due to poor quality of included studies." Cannot score "yes" for this question if scored "no" for question 7.

---

9. Were the methods used to combine the findings of studies appropriate?
For the pooled results, a test should be done to ensure the studies were combinable, to assess their homogeneity (i.e., Chi-squared test for homogeneity, I^2). If heterogeneity exists a random effects model should be used and/or the clinical appropriateness of combining should be taken into consideration (i.e., is it sensible to combine?).

Note: Indicate "yes" if they mention or describe heterogeneity, i.e., if they explain that they cannot pool because of heterogeneity/variability between interventions.

Reviewers' decision notes: This question was assigned a YES if methods to combine overall findings or composite measures and/or subgroup findings were valid. Two categories of meta-analyses were possible- (1) main or overall meta-analysis for each group of plastic chemicals (i.e., BPA, phthalates, flame retardants, PFAS) and (2) meta-analysis of specific chemicals within a group of plastic chemical (i.e., specific phthalate metabolites MMP, MEP, MBzP etc for phthalates, or PCB congeners like PCB 118, 170). If only one of these analyses is valid, methods were considered partially correct and therefore Q9 was assigned a CAN'T ANSWER.

□ Yes
□ No
□ Can't answer
□ Not applicable

---

10. Was the likelihood of publication bias assessed?
An assessment of publication bias should include a combination of graphical aids (e.g., funnel plot, other available tests) and/or statistical tests (e.g., Egger regression test, Hedges-Olken).

Note: If no test values or funnel plot included, score "no". Score "yes" if mentions that publication bias could not be assessed because there were fewer than 10 included studies.

□ Yes
□ No
□ Can't answer
□ Not applicable

---

11. Was the conflict of interest included?
Potential sources of support should be clearly acknowledged in both the systematic review and the included studies.

Note: To get a "yes," must indicate source of funding or support for the systematic review AND for each of the included studies.

Reviewers' decision notes: This question was assigned a YES, if authors declared no conflict of interest in their review but not for individual studies included. The declaration of conflict of interest from the primary studies is not required in the PRISMA reporting guide. However, as AMSTAR Q11 noted, the team included the following statement in the appraisal: authors declared no conflict of interest in their

□ Yes
□ No
□ Can't answer
□ Not applicable

review, but no declarations from the authors of the primary studies included were also noted.

Additional reviewers' decision notes: For pooled analyses, questions and decisions were applied as in the systematic reviews. This is because some pooled analyses had the same considerations with those of the systematic reviews particularly with questions 2, 3 and 11. Some pooled analyses conducted duplicate data extraction but not selection of data sources (Q2), conducted a search of the literature though not comprehensive (Q3), and authors declared no conflict of interest in their review but not for the individual studies or data sources.

Shea *et al. BMC Medical Research Methodology* 2007 **7**:10 doi:10.1186/1471-2288-7-10

1.4 Decision rules for harvest plots

- Plots display dichotomous outcomes or those measured on a continuous scale. An outcome may by measured by either or by both (e.g. abnormal sperm motility is binary, sperm motility continuous). If both used, they are represented on individual rows.
- If results are not significant, they are placed in the 'no change' section of the plot and an arrow (<,>) indicating direction of effect is included in each bar. No arrow (-) should be presented when the relative risk (RR)/odds ratio (OR) etc is 1 (dichotomous data) or the regression coefficient or mean difference is 0 (continuous).
- Bars representing main analyses are indicated by shading in the plot; subgroup data is presented with no shading in the plot i.e. outline only. Where it is indeterminable i.e. multiple analyses are not in fact subgroups of a main analysis – all should be shaded.
- Considering ordering within each column (i.e. in the decrease, no change, increase sections) BPA (pink), phthalates (blue), flame retardants (green), PFAS (orange) and are presented left to right. All data relating to individual plastic chemicals as well as their total (e.g. individual phthalates as well as total phthalates) should be plotted (pending further rules described below). Within each plastic chemical group, the order of these is based on their molecular weight (lowest to highest from left to right for phthalates and PFAS; flame retardants by their group (I, II or III) and then numerical order of congeners).
- Height of bars dependent on methodological quality as determined by each reviews AMSTAR score. Low quality reflects a score of 1-4, moderate quality (5-8) and high quality (9-11). Each bar represents the results from an individual meta-analysis; the citation number needs to be included (indicated below each bar).
- Where outcome data was provided separately for different population groups (e.g. adults, children, special exposure in Population column) and timing of exposure (prenatal and/or postnatal), where possible this should be indicated in the first/title column, any further differentiation should be indicated above the bar. Similar for age (adult/child) and gender (boys/girls).
- For reviews providing multiple estimates of the same outcome (e.g. PFOA childhood obesity presents effect size; relative risk and odds ratio), effect size was plotted. Random effects model should be plotted preferentially where fixed is also provided. Adjusted/transformed estimates should be plotted preferentially rather than unadjusted/untransformed estimates.
- Consider all outcomes provided in data tables to be plotted (even if multiple methods to measure same outcome e.g. all assay methods measuring sperm motion) however the following decisions regarding additional subgroup inclusions were made. Ideally the review will have presented a main analysis where the outcome has been measured with a particular technique, or sample - that is the analysis preferentially plotted, other subgroups should be plotted where there is some indication of statistical significance only or where they summarise the similarity across the dataset.
- Subgroup data **plotted** relating to:
    - population (i.e. gender (even if mixed e.g. male and female infants); age (e.g. children; adults); pregnancy (and non-pregnant population);
    - timing/timepoints (e.g. birth; 6 months etc); ideally representative one should be selected;
    - highest versus lowest exposure categories preferentially;

- If subgroups from multiple sampling of same exposure available, preferentially plot exposure in "urine" for BPA and phthalates and "blood, serum, milk etc" for PCBs, PFASs. These are most common due to hydro/lipophilicity.
- Where subgroups by differentiation of outcome measure (e.g. deep endometriosis, total endometriosis), plot main outcome preferentially (e.g. endometriosis)
- Where subgroups relate to pregnancy trimesters only plot 3rd trimester data or those that combine 3rd trimester data e.g. 2nd & 3rd trimester data

Subgroup data **not plotted** – ineligible for review

- Geographical region/country;
- Caucasian/white race/non-white race;
- sensitivity analyses (e.g. statistical model; adjusted/not adjusted (e.g. for maternal parity; birthweight (BMI z-score)
- Differentiation of study quality/appraisal score/risk of bias (e.g. high, medium, low);
- Differentiation of study design/type (e.g. case control studies, cohort studies);
- Differentiation of measurement technique, method/technique (e.g. ELISA method; HPLC method); hormone levels (e.g. LH/FSH), HOMA-IR levels (PCOS); hospital sample; laparoscopy among controls (Endometriosis)

1.5 List of excluded studies

1.5.1 Studies excluded after full text assessment against eligibility criteria

Citation	Reason for exclusion
AMSTER, E. 2019. Public health impact of coal-fired power plants: a critical systematic review of the epidemiological literature. International journal of environmental health research, 1-23.	Not plastic-associated chemicals
AZARPAZHOOH, A. & MAIN, P. A. 2008. Is there a risk of harm or toxicity in the placement of pit and fissure sealant materials? A systematic review. Journal (Canadian Dental Association), 74, 179-83.	No meta-analysis
BACH, C. C., BECH, B. H., BRIX, N., NOHR, E. A., BONDE, J. P. & HENRIKSEN, T. B. 2015. Perfluoroalkyl and polyfluoroalkyl substances and human fetal growth: A systematic review. Critical reviews in toxicology, 45, 1-15.	No meta-analysis
BACH, C. C., VESTED, A., JØRGENSEN, K. T., BONDE, J. P., HENRIKSEN, T. B. & TOFT, G. 2016. Perfluoroalkyl and polyfluoroalkyl substances and measures of human fertility: a systematic review. Critical reviews in toxicology, 46, 1-21.	No meta-analysis
BALLESTEROS, V., COSTA, O., IÑIGUEZ, C., FLETCHER, T., BALLESTER, F. & LOPEZ-ESPINOSA, M. J. 2017. Exposure to perfluoroalkyl substances and thyroid function in pregnant women and children: A systematic review of epidemiologic studies. Environment international, 99, 15-28.	No meta-analysis
BARROS, N., TULVE, N. S., HEGGEM, D. T. & BAILEY, K. 2018. Review of built and natural environment stressors impacting American-Indian/Alaska-Native children. Reviews on environmental health, 33, 349-381.	No meta-analysis
BÉRANGER, R., LE CORNET, C., SCHÜZ, J. & FERVERS, B. 2013. Occupational and environmental exposures associated with testicular germ cell tumours: systematic review of prenatal and life-long exposures. PloS one, 8, e77130.	No meta-analysis
BONDE, J. P., FLACHS, E. M., RIMBORG, S., GLAZER, C. H., GIWERCMAN, A., RAMLAU-HANSEN, C. H., HOUGAARD, K. S., HØYER, B. B., HÆRVIG, K. K., PETERSEN, S. B., RYLANDER, L., SPECHT, I. O., TOFT, G. & BRÄUNER, E. V. 2016. The epidemiologic evidence linking prenatal and postnatal exposure to endocrine disrupting chemicals with male reproductive disorders: a systematic review and meta-analysis. Human reproduction update, 23, 104-125.	EDCs with pesticides
BORRELLI, I. 2007. [Endocrine disruptors: literature review on toxicology and application field in occupational medicine]. Giornale italiano di medicina del lavoro ed ergonomia, 29, 526-8	Italian
BOWMAN, J. D. & CHOUDHURY, M. 2016. Phthalates in neonatal health: friend or foe? Journal of developmental origins of health and disease, 7, 652-664.	No meta-analysis
CAPOROSSI, L. & PAPALEO, B. 2015. Exposure to Bisphenol a and Gender Differences: From Rodents to Humans Evidences and Hypothesis about the Health Effects. Journal of xenobiotics, 5, 5264.	No meta-analysis
CARRÉ, J., GATIMEL, N., MOREAU, J., PARINAUD, J. & LÉANDRI, R. 2017. Does air pollution play a role in infertility?: a systematic review. Environmental health : a global access science source, 16, 82.	No meta-analysis
CESARIO, S. K. & HUGHES, L. A. 2007. Precocious puberty: a comprehensive review of literature. Journal of obstetric, gynecologic, and neonatal nursing : JOGNN / NAACOG, 36, 263-74.	No meta-analysis

Citation	Reason for exclusion
CHEN ZEE, E., CORNET, P., LAZIMI, G., RONDET, C., LOCHARD, M., MAGNIER, A. M. & IBANEZ, G. 2013. Impact of endocrine disrupting chemicals on birth outcomes. Gynécologie, obstétrique & fertilité, 41, 601-10.	French
COCUZZA, M. & ESTEVES, S. C. 2014. Shedding light on the controversy surrounding the temporal decline in human sperm counts: a systematic review. The Scientific World Journal, 2014, 365691.	No meta-analysis
DAI, Y., HUO, X., CHENG, Z., FAAS, M. M. & XU, X. 2020. Early-life exposure to widespread environmental toxicants and maternal-fetal health risk: A focus on metabolomic biomarkers. The Science of the total environment, 739, 139626	No meta-analysis
DE COCK, M., MAAS, Y. G. & VAN DE BOR, M. 2012. Does perinatal exposure to endocrine disruptors induce autism spectrum and attention deficit hyperactivity disorders? Review. Acta paediatrica (Oslo, Norway : 1992), 101, 811-8.	No meta-analysis
DE COCK, M. & VAN DE BOR, M. 2014. Obesogenic effects of endocrine disruptors, what do we know from animal and human studies? Environment international, 70, 15-24.	No meta-analysis
DZIEWIRSKA, E., HANKE, W. & JUREWICZ, J. 2018. Environmental non-persistent endocrine-disrupting chemicals exposure and reproductive hormones levels in adult men. International journal of occupational medicine and environmental health, 31, 551-573.	No meta-analysis
EJAREDAR, M., LEE, Y., ROBERTS, D. J., SAUVE, R. & DEWEY, D. 2017. Bisphenol A exposure and children's behavior: A systematic review. Journal of exposure science & environmental epidemiology, 27, 175-183.	No meta-analysis
EJAREDAR, M., NYANZA, E. C., TEN EYCKE, K. & DEWEY, D. 2015. Phthalate exposure and childrens neurodevelopment: A systematic review. Environmental research, 142, 51-60.	No meta-analysis
FERNÁNDEZ, M. F., OLMOS, B. & OLEA, N. 2007. [Exposure to endocrine disruptors and male urogenital tract malformations [cryptorchidism and hypospadias]]. Gaceta sanitaria / S.E.S.P.A.S, 21, 500-14.	Spanish
GASCON, M., MORALES, E., SUNYER, J. & VRIJHEID, M. 2013. Effects of persistent organic pollutants on the developing respiratory and immune systems: a systematic review. Environment international, 52, 51-65.	No meta-analysis
GÓMEZ-MERCADO, C. A., MEJÍA-SANDOVAL, G., SEGURA-CARDONA, Á. M., ARANGO-ÁLZATE, C. M., HERNÁNDEZ-GONZÁLEZ, S. I., PATIÑO-GARCÍA, D. F. & BARRAZA-VILLARREAL, A. 2018. Pregnant Women's Exposure to Bisphenol A (BPA) and its Relation to their Children's Obesity: A systematic Review. *Rev. Fac. Nac. Salud Pública,* 36, 66-74.	No meta-analysis
GOODMAN, M., LAKIND, J. S. & MATTISON, D. R. 2014. Do phthalates act as obesogens in humans? A systematic review of the epidemiological literature. Critical reviews in toxicology, 44, 151-75.	No meta-analysis
GUTIÉRREZ-TORRES, D. S., BARRAZA-VILLARREAL, A., HERNANDEZ-CADENA, L., ESCAMILLA-NUÑEZ, C. & ROMIEU, I. 2018. Prenatal Exposure to Endocrine Disruptors and Cardiometabolic Risk in Preschoolers: A Systematic Review Based on Cohort Studies. Annals of global health, 84, 239-249.	No meta-analysis

Citation	Reason for exclusion
HAREL, Z., HAREL, S., SHAH, P. S., WALD, R., PERL, J. & BELL, C. M. 2013. Gastrointestinal adverse events with sodium polystyrene sulfonate (Kayexalate) use: a systematic review. The American journal of medicine, 126**,** 264.e9-264.e24	No meta-analysis
HIPWELL, A. E., KAHN, L. G., FACTOR-LITVAK, P., PORUCZNIK, C. A., SIEGEL, E. L., FICHOROVA, R. N., HAMMAN, R. F., KLEIN-FEDYSHIN, M., HARLEY, K. G. & PROGRAM COLLABORATORS FOR ENVIRONMENTAL INFLUENCES ON CHILD HEALTH, O. 2019. Exposure to non-persistent chemicals in consumer products and fecundability: a systematic review. Human reproduction update, 25**,** 51-71.	No meta-analysis
HØYER, B. B., LENTERS, V., GIWERCMAN, A., JÖNSSON, B. A. G., TOFT, G., HOUGAARD, K. S., BONDE, J. P. E. & SPECHT, I. O. 2018. Impact of Di-2-Ethylhexyl Phthalate Metabolites on Male Reproductive Function: a Systematic Review of Human Evidence. Current environmental health reports, 5**,** 20-33.	No meta-analysis
JAACKS, L. M. & STAIMEZ, L. R. 2015. Association of persistent organic pollutants and non-persistent pesticides with diabetes and diabetes-related health outcomes in Asia: A systematic review. Environment international, 76C**,** 57-70.	No meta-analysis
JEDDI, M. Z., JANANI, L., MEMARI, A. H., AKHONDZADEH, S. & YUNESIAN, M. 2016. The role of phthalate esters in autism development: A systematic review. Environmental research, 151**,** 493-504.	No meta-analysis
KALKBRENNER, A. E., SCHMIDT, R. J. & PENLESKY, A. C. 2014. Environmental chemical exposures and autism spectrum disorders: a review of the epidemiological evidence. Current problems in pediatric and adolescent health care, 44**,** 277-318.	No meta-analysis
KALLIORA, C., MAMOULAKIS, C., VASILOPOULOS, E., STAMATIADES, G. A., KALAFATI, L., BAROUNI, R., KARAKOUSI, T., ABDOLLAHI, M. & TSATSAKIS, A. 2018. Association of pesticide exposure with human congenital abnormalities. Toxicology and applied pharmacology, 346**,** 58-75.	No meta-analysis
KAY, V. R., BLOOM, M. S. & FOSTER, W. G. 2014. Reproductive and developmental effects of phthalate diesters in males. Critical reviews in toxicology, 44**,** 1-32.	No meta-analysis
KAY, V. R., CHAMBERS, C. & FOSTER, W. G. 2013. Reproductive and developmental effects of phthalate diesters in females. Critical reviews in toxicology, 43**,** 200-19.	No meta-analysis
KERN, J. K., GEIER, D. A., HOMME, K. G., KING, P. G., BJØRKLUND, G., CHIRUMBOLO, S. & GEIER, M. R. 2017. Developmental neurotoxicants and the vulnerable male brain: a systematic review of suspected neurotoxicants that disproportionally affect males. Acta neurobiologiae experimentalis, 77**,** 269-296.	No meta-analysis
KILCOYNE, K. R. & MITCHELL, R. T. 2019. Effect of environmental and pharmaceutical exposures on fetal testis development and function: a systematic review of human experimental data. Human reproduction update, 25**,** 397-421.	No meta-analysis
KIM, Y. R., PACELLA, R. E., HARDEN, F. A., WHITE, N. & TOMS, L. L. 2019. A systematic review: Impact of endocrine disrupting chemicals exposure on fecundity as measured by time to pregnancy. Environmental research, 171**,** 119-133.	No meta-analysis

Citation	Reason for exclusion
KRAMER, S., HIKEL, S. M., ADAMS, K., HINDS, D. & MOON, K. 2012. Current status of the epidemiologic evidence linking polychlorinated biphenyls and non-hodgkin lymphoma, and the role of immune dysregulation. Environmental health perspectives, 120**,** 1067-75.	No meta-analysis
KUO, C. C., MOON, K., THAYER, K. A. & NAVAS-ACIEN, A. 2013. Environmental chemicals and type 2 diabetes: an updated systematic review of the epidemiologic evidence. Current diabetes reports, 13**,** 831-49.	No meta-analysis
JAAKKOLA, J. J. K., KNIGHT, T. L., INSTITUTE OF, O. & ENVIRONMENTAL MEDICINE, U. O. B. B. B. T. T. U. K. J. J. B. A. U. 2008. The role of exposure to phthalates from polyvinyl chloride products in the development of asthma and allergies: a systematic review and meta-analysis. *Environmental Health Perspectives,* 116**,** 845-853.	Indirect measure of exposure, not human biospecimen
LAKIND, J. S., GOODMAN, M. & MATTISON, D. R. 2014. Bisphenol A and indicators of obesity, glucose metabolism/type 2 diabetes and cardiovascular disease: a systematic review of epidemiologic research. Critical reviews in toxicology, 44**,** 121-50.	No meta-analysis
LAM, J., KOUSTAS, E., SUTTON, P., JOHNSON, P. I., ATCHLEY, D. S., SEN, S., ROBINSON, K. A., AXELRAD, D. A. & WOODRUFF, T. J. 2014. The Navigation Guide—Evidence-Based Medicine Meets Environmental Health: Integration of Animal and Human Evidence for PFOA Effects on Fetal Growth. Environmental health perspectives, 122**,** 1040-51.	No meta-analysis
LINDBOHM, M. L. 1993. Effects of styrene on the reproductive health of women: a review. IARC scientific publications**,** 163-9.	No meta-analysis
MALLOZZI, M., LEONE, C., MANURITA, F., BELLATI, F. & CASERTA, D. 2017. Endocrine Disrupting Chemicals and Endometrial Cancer: An Overview of Recent Laboratory Evidence and Epidemiological Studies. International journal of environmental research and public health, 14.	No meta-analysis
MARÍ-BAUSET, S., DONAT-VARGAS, C., LLÓPIS-GONZÁLEZ, A., MARÍ-SANCHIS, A., PERAITA-COSTA, I., LLOPIS-MORALES, J. & MORALES-SUÁREZ-VARELA, M. 2018. Endocrine Disruptors and Autism Spectrum Disorder in Pregnancy: A Review and Evaluation of the Quality of the Epidemiological Evidence. Children (Basel, Switzerland), 5.	No meta-analysis
MÍNGUEZ-ALARCÓN, L., HAUSER, R. & GASKINS, A. J. 2016. Effects of bisphenol A on male and couple reproductive health: a review. Fertility and sterility, 106**,** 864-70.	No meta-analysis
NEGRI, E., BOSETTI, C., FATTORE, E. & LA VECCHIA, C. 2003. Environmental exposure to polychlorinated biphenyls (PCBs) and breast cancer: a systematic review of the epidemiological evidence. European journal of cancer prevention: the official journal of the European Cancer Prevention Organisation (ECP), 12**,** 509-16.	No meta-analysis
NORTH, M. L., TAKARO, T. K., DIAMOND, M. L. & ELLIS, A. K. 2014. Effects of phthalates on the development and expression of allergic disease and asthma. Annals of allergy, asthma & immunology: official publication of the American College of Allergy, Asthma, & Immunology, 112**,** 496-502.	No meta-analysis
OPPENEER, S. J. & ROBIEN, K. 2015. Bisphenol A exposure and associations with obesity among adults: a critical review. Public health nutrition, 18**,** 1-17.	No meta-analysis

**Citation**	**Reason for exclusion**
OVERGAARD, L. E., BONEFELD, C. M., FREDERIKSEN, H., MAIN, K. M. & THYSSEN, J. P. 2016. The association between phthalate exposure and atopic dermatitis with a discussion of phthalate induced secretion of interleukin-1β and thymic stromal lymphopoietin. Expert review of clinical immunology, 12**,** 609-16.	No meta-analysis
PEINADO, F. M., ARTACHO-CORDÓN, F., BARRIOS-RODRÍGUEZ, R. & ARREBOLA, J. P. 2020. Influence of polychlorinated biphenyls and organochlorine pesticides on the inflammatory milieu. A systematic review of in vitro, in vivo and epidemiological studies. Environmental research, 186**,** 109561.	No meta-analysis
PERETZ, J., VROOMAN, L., RICKE, W. A., HUNT, P. A., EHRLICH, S., HAUSER, R., PADMANABHAN, V., TAYLOR, H. S., SWAN, S. H., VANDEVOORT, C. A. & FLAWS, J. A. 2014. Bisphenol a and reproductive health: update of experimental and human evidence, 2007-2013. Environmental health perspectives, 122**,** 775-86.	No meta-analysis
PERGIALIOTIS, V., KOTROGIANNI, P., CHRISTOPOULOS-TIMOGIANNAKIS, E., KOUTAKI, D., DASKALAKIS, G. & PAPANTONIOU, N. 2018. Bisphenol A and adverse pregnancy outcomes: a systematic review of the literature. The journal of maternal-fetal & neonatal medicine: the official journal of the European Association of Perinatal Medicine, the Federation of Asia and Oceania Perinatal Societies, the International Society of Perinatal Obstetricians, 31**,** 1-15.	No meta-analysis
PETERSEN, K. U., LARSEN, J. R., DEEN, L., FLACHS, E. M., HÆRVIG, K. K., HULL, S. D., BONDE, J. P. E. & TØTTENBORG, S. S. 2020. Per- and polyfluoroalkyl substances and male reproductive health: a systematic review of the epidemiological evidence. Journal of toxicology and environmental health. Part B, Critical reviews, 23**,** 276-291.	No meta-analysis
POLAŃSKA, K., JUREWICZ, J. & HANKE, W. 2013. Review of current evidence on the impact of pesticides, polychlorinated biphenyls and selected metals on attention deficit / hyperactivity disorder in children. International journal of occupational medicine and environmental health, 26**,** 16-38.	No meta-analysis
RADKE, E. G., BRAUN, J. M., MEEKER, J. D. & COOPER, G. S. 2018. Phthalate exposure and male reproductive outcomes: A systematic review of the human epidemiological evidence. Environment international, 121**,** 764-793.	No meta-analysis
RADKE, E. G., GALIZIA, A., THAYER, K. A. & COOPER, G. S. 2019. Phthalate exposure and metabolic effects: a systematic review of the human epidemiological evidence. Environment international, 132**,** 104768.	No meta-analysis
RADKE, E. G., GLENN, B. S., BRAUN, J. M. & COOPER, G. S. 2019. Phthalate exposure and female reproductive and developmental outcomes: a systematic review of the human epidemiological evidence. Environment international, 130**,** 104580.	No meta-analysis
RANJIT, N., SIEFERT, K. & PADMANABHAN, V. 2010. Bisphenol-A and disparities in birth outcomes: a review and directions for future research. Journal of Perinatology: official journal of the California Perinatal Association, 30**,** 2-9.	No meta-analysis
RAPPAZZO, K. M., COFFMAN, E. & HINES, E. P. 2017. Exposure to Perfluorinated Alkyl Substances and Health Outcomes in Children: A Systematic Review of the Epidemiologic Literature. International journal of environmental research and public health, 14.	No meta-analysis

Citation	Reason for exclusion
RIVOLLIER, F., KREBS, M. O. & KEBIR, O. 2019. Perinatal Exposure to Environmental Endocrine Disruptors in the Emergence of Neurodevelopmental Psychiatric Diseases: A Systematic Review. International journal of environmental research and public health, 16.	No meta-analysis
ROCHESTER, J. R., BOLDEN, A. L. & KWIATKOWSKI, C. F. 2018. Prenatal exposure to bisphenol A and hyperactivity in children: a systematic review and meta-analysis. Environment international, 114, 343-356.	No meta-analysis
ROSSIGNOL, D. A., GENUIS, S. J. & FRYE, R. E. 2014. Environmental toxicants and autism spectrum disorders: a systematic review. Translational psychiatry, 4, e360.	No meta-analysis
ROTH, N. & WILKS, M. F. 2014. Neurodevelopmental and neurobehavioural effects of polybrominated and perfluorinated chemicals: a systematic review of the epidemiological literature using a quality assessment scheme. Toxicology letters, 230, 271-81.	No meta-analysis
RUIZ-HERNANDEZ, A., KUO, C. C., RENTERO-GARRIDO, P., TANG, W. Y., REDON, J., ORDOVAS, J. M., NAVAS-ACIEN, A. & TELLEZ-PLAZA, M. 2015. Environmental chemicals and DNA methylation in adults: a systematic review of the epidemiologic evidence. Clinical epigenetics, 7, 55.	No meta-analysis
SAIKAT, S., KREIS, I., DAVIES, B., BRIDGMAN, S. & KAMANYIRE, R. 2013. The impact of PFOS on health in the general population: a review. Environmental science. Processes & impacts, 15, 329-35.	No meta-analysis
SALAY, E. & GARABRANT, D. 2009. Polychlorinated biphenyls and thyroid hormones in adults: a systematic review appraisal of epidemiological studies. Chemosphere, 74, 1413-9.	No meta-analysis
SHARP, D. 2009. Environmental toxins, a potential risk factor for diabetes among Canadian Aboriginals. International journal of circumpolar health, 68, 316-26.	Not plastic-associated chemicals
SOAVE, I., OCCHIALI, T., ASSORGI, C., MARCI, R. & CASERTA, D. 2020. Environmental toxin exposure in polycystic ovary syndrome women and possible ovarian neoplastic repercussion. Current Medical Research & Opinion, 36, 693-703.	No meta-analysis
SOAVE, I., OCCHIALI, T., ASSORGI, C., MARCI, R. & CASERTA, D. 2020. Environmental toxins exposure in PCOS women and possible ovarian neoplastic repercussion. Current medical research and opinion, 1.	No meta-analysis
SOWLAT, M. H., LOTFI, S., YUNESIAN, M., AHMADKHANIHA, R. & RASTKARI, N. 2016. The association between bisphenol A exposure and type-2 diabetes: a world systematic review. Environmental science and pollution research international, 23, 21125-21140.	No meta-analysis
STANIFER, J. W., STAPLETON, H. M., SOUMA, T., WITTMER, A., ZHAO, X. & BOULWARE, L. E. 2018. Perfluorinated Chemicals as Emerging Environmental Threats to Kidney Health: A Scoping Review. Clinical journal of the American Society of Nephrology: CJASN, 13, 1479-1492.	No meta-analysis
STRAKOVSKY, R. S. & SCHANTZ, S. L. 2018. Impacts of bisphenol A (BPA) and phthalate exposures on epigenetic outcomes in the human placenta. Environmental epigenetics, 4, dvy022.	No meta-analysis
SUGENG, E. J., DE COCK, M., SCHOONMADE, L. J. & VAN DE BOR, M. 2017. Toddler exposure to flame retardant chemicals: Magnitude, health concern and potential risk- or protective factors of exposure: Observational studies summarized in a systematic review. Chemosphere, 184, 820-831.	No meta-analysis

Citation	Reason for exclusion
SUN, D., WANG, Q., CHEN, H., XU, F., ZHANG, Y., ZHANG, T., LIU, H. & YE, L. 2017. Changes of serum thyroid hormone levels in DEHP exposure population: A meta-analysis. Journal of Jilin University Medicine Edition, 43, 66-72.	Chinese
SWEENEY, M. R., O'LEARY, K. G., JENEY, Z., BRAUNLIN, M. C. & GIBB, H. J. 2019. Systematic review and quality ranking of studies of two phthalate metabolites and anogenital distance, bone health, inflammation, and oxidative stress. Critical reviews in toxicology, 1-21.	No meta-analysis
TERRELL, M. L., HARTNETT, K. P. & MARCUS, M. 2011. Can environmental or occupational hazards alter the sex ratio at birth? A systematic review. Emerging health threats journal, 4, 7109.	No meta-analysis
TOMZA-MARCINIAK, A., STEPKOWSKA, P., KUBA, J. & PILARCZYK, B. 2018. Effect of bisphenol A on reproductive processes: A review of in vitro, in vivo and epidemiological studies. J Appl Toxicol, 38, 51-80.	No meta-analysis
TSAI, M. S., CHEN, M. H., LIN, C. C., NG, S., HSIEH, C. J., LIU, C. Y., HSIEH, W. S. & CHEN, P. C. 2017. Children's environmental health based on birth cohort studies of Asia. The Science of the total environment, 609, 396-409.	No meta-analysis
VABRE, P., GATIMEL, N., MOREAU, J., GAYRARD, V., PICARD-HAGEN, N., PARINAUD, J. & LEANDRI, R. D. 2017. Environmental pollutants, a possible etiology for premature ovarian insufficiency: a narrative review of animal and human data. Environmental health : a global access science source, 16, 37.	No meta-analysis
VAN CAUWENBERGH, O., DI SERAFINO, A., TYTGAT, J. & SOUBRY, A. 2020. Transgenerational epigenetic effects from male exposure to endocrine-disrupting compounds: a systematic review on research in mammals. Clinical epigenetics, 12, 65.	No meta-analysis
VERNER, M. A., LOCCISANO, A. E., MORKEN, N. H., YOON, M., WU, H., MCDOUGALL, R., MAISONET, M., MARCUS, M., KISHI, R., MIYASHITA, C., CHEN, M. H., HSIEH, W. S., ANDERSEN, M. E., CLEWELL, H. J. & LONGNECKER, M. P. 2015. Associations of Perfluoroalkyl Substances (PFAS) with Lower Birth Weight: An Evaluation of Potential Confounding by Glomerular Filtration Rate Using a Physiologically Based Pharmacokinetic Model (PBPK). Environmental health perspectives, 123, 1317-24.	Simulated population
XIE, M. Y., NI, H., ZHAO, D. S., WEN, L. Y., LI, K. S., YANG, H. H., WANG, S. S., ZHANG, H. & SU, H. 2016. Exposure to bisphenol A and the development of asthma: a systematic review of cohort studies. Reproductive toxicology (Elmsford, N.Y.), 65, 224-229.	No meta-analysis
XU, J., CHEN, H., XU, F., WANG, Q., ZHANG, Y., LIU, H., ZHANG, T. & YE, L. 2017. Association between phthalate ester exposure and population obesity: A Meta-analysis. Journal of Jilin University Medicine Edition, 43, 306-310	Chinese
YAGHJYAN, L., GHITA, G. L., DUMONT-DRISCOLL, M., YOST, R. A. & CHANG, S. H. 2016. Maternal exposure to di-2-ethylhexylphthalate and adverse delivery outcomes: a systematic review. Reproductive toxicology (Elmsford, N.Y.), 65, 76-86.	No meta-analysis
YANG, L., SHANG, L., WANG, S., YANG, W., HUANG, L., QI, C., GURCAN, A., YANG, Z. & CHUNG, M. C. 2020. The association between prenatal exposure to polycyclic aromatic hydrocarbons and birth weight: A meta-analysis. PloS one, 15, e0236708.	Not plastic-associated chemicals

Citation	Reason for exclusion
ZAMKOWSKA, D., KARWACKA, A., JUREWICZ, J. & RADWAN, M. 2018. Environmental exposure to non-persistent endocrine disrupting chemicals and semen quality: An overview of the current epidemiological evidence. International journal of occupational medicine and environmental health, 31**,** 377-414.	No meta-analysis
ZAREAN, M., KEIKHA, M., POURSAFA, P., KHALIGHINEJAD, P., AMIN, M. & KELISHADI, R. 2016. A systematic review on the adverse health effects of di-2-ethylhexyl phthalate. Environmental science and pollution research international, 23**,** 24642-24693.	No meta-analysis
ZAREAN, M., POURSAFA, P., AMIN, M. M. & KELISHADI, R. 2018. Association of Endocrine Disrupting Chemicals, Bisphenol A and Phthalates, with Childhood Obesity: A Systematic Review. Journal of Pediatrics Review, 6**,** 1-16.	No meta-analysis
ZEINOMAR, N., OSKAR, S., KEHM, R. D., SAHEBZEDA, S. & TERRY, M. B. 2020. Environmental exposures and breast cancer risk in the context of underlying susceptibility: A systematic review of the epidemiological literature. Environmental research, 187**,** 109346.	No meta-analysis
ZHANG, Q., CHEN, X. Z., HUANG, X., WANG, M. & WU, J. 2019. The association between prenatal exposure to phthalates and cognition and neurobehavior of children-evidence from birth cohorts. Neurotoxicology, 73**,** 199-212.	No meta-analysis
ZHENG, L. Y., SANDERS, A. P., SALAND, J. M., WRIGHT, R. O. & ARORA, M. 2017. Environmental exposures and pediatric kidney function and disease: A systematic review. Environmental research, 158**,** 625-648.	No meta-analysis
ZUCCARELLO, P., CONTI, G. O., CAVALLARO, F., COPAT, C., CRISTALDI, A., FIORE, M. & FERRANTE, M. 2018. Implication of dietary phthalates in breast cancer. A systematic review. Food and chemical toxicology: an international journal published for the British Industrial Biological Research Association, 118**,** 667-674.	No meta-analysis

1.5.2 Studies excluded due to unit of analysis error identified during critical appraisal

Citation	Reason for exclusion
BOFFETTA, P., CATALANI, S., TOMASI, C., PIRA, E. & APOSTOLI, P. 2018. Occupational exposure to polychlorinated biphenyls and risk of cutaneous melanoma: A meta-analysis. *European Journal of Cancer Prevention*, 27(1), 62-9.	multiple use of the same data in the analyses
BLIATKA, D., NIGDELIS, M. P., CHATZIMELETIOU, K., MASTORAKOS, G., LYMPERI, S. & GOULIS, D. G. 2020. The effects of postnatal exposure of endocrine disruptors on testicular function: a systematic review and a meta-analysis. *Hormones (Athens, Greece),* 19**,** 157-169.	multiple use of the same data in the analyses
FREEMAN, M. D. & KOHLES, S. S. 2012. Plasma levels of polychlorinated biphenyls, non-Hodgkin lymphoma, and causation. *Journal of environmental and public health,* 2012**,** 258981.	multiple use of the same data in the analyses
FU, Z., ZHAO, F., CHEN, K., XU, J., LI, P., XIA, D. & WU, Y. 2017. Association between urinary phthalate metabolites and risk of breast cancer and uterine leiomyoma. *Reproductive Toxicology.* 74, 132-142.	multiple use of the same data in the analyses

**Citation**	**Reason for exclusion**
LIM, J. E., PARK, S. H., JEE, S. H. & PARK, H. 2015. Body concentrations of persistent organic pollutants and prostate cancer: a meta-analysis. *Environmental science and pollution research international,* 22**,** 11275-84.	multiple use of the same data in the analyses
NILSEN, F. M. & TULVE, N. S. 2020. A systematic review and meta-analysis examining the interrelationships between chemical and non-chemical stressors and inherent characteristics in children with ADHD. *Environmental research,* 180**,** 108884.	multiple use of the same data in the analyses
TANG, M., CHEN, K., YANG, F. & LIU, W. 2014. Exposure to organochlorine pollutants and type 2 diabetes: a systematic review and meta-analysis. *PloS one,* 9**,** e85556.	multiple use of the same data in the analyses
WANG, C., YANG, L., WANG, S., ZHANG, Z., YU, Y., WANG, M., CROMIE, M., GAO, W. & WANG, S. L. 2016. The classic EDCs, phthalate esters and organochlorines, in relation to abnormal sperm quality: a systematic review with meta-analysis. *Scientific reports,* 6**,** 19982.	multiple use of the same data in the analyses
ZAREAN, M., KEIKHA, M., FEIZI, A., KAZEMITABAEE, M. & KELISHADI, R. 2019. The role of exposure to phthalates in variations of anogenital distance: A systematic review and meta-analysis. *Environmental Pollution (Barking, Essex: 1987)*, 247, 172-9.	multiple use of the same data in the analyses (both exposure and outcomes)
ZHOU, Z., LEI, Y., WEI, W., ZHAO, Y., JIANG, Y., WANG, N., LI, X. & CHEN, X. 2019. Association between prenatal exposure to bisphenol a and birth outcomes: A systematic review with meta-analysis. *Medicine,* 98**,** e17672.	multiple use of the same data in the analyses

1.6 Critical appraisal of included reviews and pooled analyses

Author & Year	Q1	Q2	Q3	Q4	Q5	Q6	Q7	Q8	Q9	Q10	Q11	Score
*Dunder et al. 2019 [84]	N	CA	Y	N	N	Y	Y	Y	CA	Y	Y	6
*Gascon et al. 2014 [91]	N	N	N	N	N	Y	N	N	Y	N	Y	3
*Govarts et al. 2012 [50]	N	N	N	N	N	Y	N	N	Y	N	Y	3
*Li et al. 2015 [83]	N	Y	N	N	Y	Y	N	N	Y	N	N	4
*Wu et al. 2013 [68]	N	Y	CA	N	N	Y	N	N	Y	N	Y	4
Bigambo et al. 2020 [55]	N	CA	Y	N	N	Y	Y	CA	CA	Y	Y	5
Hu et al. 2018a [46]	N	Y	Y	N	Y	Y	Y	N	Y	Y	Y	8
Cai et al. 2015 [63]	N	CA	N	N	N	Y	Y	Y	Y	Y	Y	6
Cai et al. 2019 [60]	N	Y	N	N	N	Y	Y	Y	Y	Y	Y	7
Cano-Sancho et al. 2019 [61]	Y	Y	CA	N	N	Y	Y	Y	Y	Y	Y	8
Catalani et al. 2019 [96]	Y	CA	Y	N	N	Y	Y	N	CA	Y	Y	6
Dorman et al. 2019 [58]	Y	Y	Y	N	N	Y	Y	Y	Y	N	Y	8
*Forns et al. 2020 [77]	N	N	N	N	N	Y	N	N	Y	N	Y	3
Fu et al. 2020 [87]	N	CA	Y	N	N	Y	Y	Y	CA	Y	Y	6
Golestanzadeh et al. 2019 [54]	N	CA	Y	N	N	Y	Y	N	CA	Y	Y	5
Golestanzadeh et al. 2020 [56]	N	N	Y	N	N	Y	Y	Y	N	Y	Y	6
Hwang et al. 2018 [65]	N	CA	Y	N	N	Y	Y	N	Y	Y	Y	6
Johnson et al. 2014 [44]	Y	Y	Y	Y	N	Y	Y	Y	Y	Y	Y	10
Kim et al. 2018 [70]	N	CA	Y	N	N	Y	Y	Y	Y	Y	Y	7
Kim et al. 2019a [71]	N	Y	N	N	N	Y	N	N	Y	Y	Y	5
Kim et al. 2019b [78]	N	CA	Y	N	N	Y	Y	N	Y	Y	Y	6
Lam et al. 2017 [74]	Y	Y	Y	Y	Y	Y	Y	Y	Y	Y	Y	11
Lee et al. 2018 [75]	N	CA	Y	N	N	Y	Y	Y	Y	Y	Y	7
Leng et al. 2016 [94]	N	CA	Y	Y	Y	Y	Y	N	Y	Y	Y	8
Li et al. 2017 [88]	Y	Y	Y	N	N	Y	Y	Y	Y	Y	Y	9
Liu et al. 2018 [82]	N	Y	Y	N	N	Y	Y	N	Y	Y	Y	7
Luo et al. 2020 [89]	N	Y	Y	N	N	Y	Y	N	Y	Y	Y	7
Negri et al. 2017 [47]	N	Y	Y	N	N	Y	Y	Y	Y	Y	Y	8
Nelson et al. 2020 [59]	N	Y	Y	N	N	Y	Y	Y	Y	N	Y	7
Nieminen et al. 2013 [49]	N	CA	Y	N	Y	Y	N	N	CA	N	N	3
Park et al. 2016 [85]	N	Y	Y	N	N	Y	Y	Y	CA	Y	Y	7
Radke et al. 2020 [76]	Y	Y	Y	N	N	Y	Y	Y	Y	N	Y	8
Rancière et al. 2015 [66]	N	CA	Y	N	Y	Y	Y	Y	Y	N	Y	7
Ribeiro et al. 2019 [81]	N	Y	N	N	N	Y	Y	N	Y	Y	Y	6
Ribeiro et al. 2020 [80]	N	Y	Y	N	N	Y	Y	Y	CA	Y	Y	7
Roy et al. 2015 [62]	N	N	N	N	N	Y	N	N	Y	N	Y	3
Shoshtari-Yeganeh et al. 2019 [69]	N	Y	N	Y	N	Y	CA	N	N	Y	N	4
Song et al. 2016 [67]	Y	Y	CA	N	N	Y	N	N	Y	Y	Y	6
Steenland et al. 2018 [51]	N	CA	N	N	N	Y	N	N	Y	Y	Y	4
Wu et al. 2020b [90]	N	Y	Y	N	N	N	Y	N	CA	Y	Y	5
Wen et al. 2015 [57]	N	CA	Y	Y	N	Y	Y	Y	Y	N	Y	7

Author & Year	Q1	Q2	Q3	Q4	Q5	Q6	Q7	Q8	Q9	Q10	Q11	Score
Wen et al. 2019 [64]	N	N	Y	Y	N	Y	Y	Y	CA	Y	Y	7
Wu et al. 2020a [79]	N	CA	Y	N	N	Y	Y	N	CA	Y	Y	5
Hu et al. 2018b [73]	N	Y	Y	Y	N	Y	Y	Y	Y	Y	Y	9
Zani et al. 2013 [92]	N	N	N	N	N	Y	N	N	Y	N	N	2
Zani et al. 2017 [95]	N	CA	Y	N	N	Y	N	N	Y	Y	Y	5
Zhang et al. 2015 [93]	N	Y	Y	Y	N	Y	Y	Y	CA	Y	Y	8
Zhang et al. 2020 [48]	N	Y	Y	N	N	Y	Y	Y	N	Y	Y	7
Zhao et al. 2015 [72]	Y	Y	CA	Y	Y	Y	Y	Y	N	Y	Y	9
Zhao et al. 2017 [45]	Y	Y	Y	Y	N	Y	Y	N	Y	Y	Y	9
Zhong et al. 2020 [52]	N	CA	Y	N	N	Y	N	N	Y	Y	Y	5
Zou et al. 2019 [53]	N	Y	Y	N	N	N	N	N	CA	Y	Y	4

**pooled analysis*
Y, yes; N, no; CA, can't answer: AMSTAR 1 -measurement tool to assess the methodological quality of systematic reviews

1. *Was an 'a priori' design provided?*
2. *Was there duplicate study selection and data extraction?*
3. *Was a comprehensive literature search performed?*
4. *Was the status of publication (i.e. grey literature) used as an inclusion criterion?*
5. *Was a list of studies (included and excluded) provided?*
6. *Were the characteristics of the included studies provided?*
7. *Was the scientific quality of the included studies assessed and documented?*
8. *Was the scientific quality of the included studies used appropriately in formulating conclusions?*
9. *Were the methods used to combine the findings of studies appropriate?*
10. *Was the likelihood of publication bias assessed?*
11. *Was the conflict of interest included?*

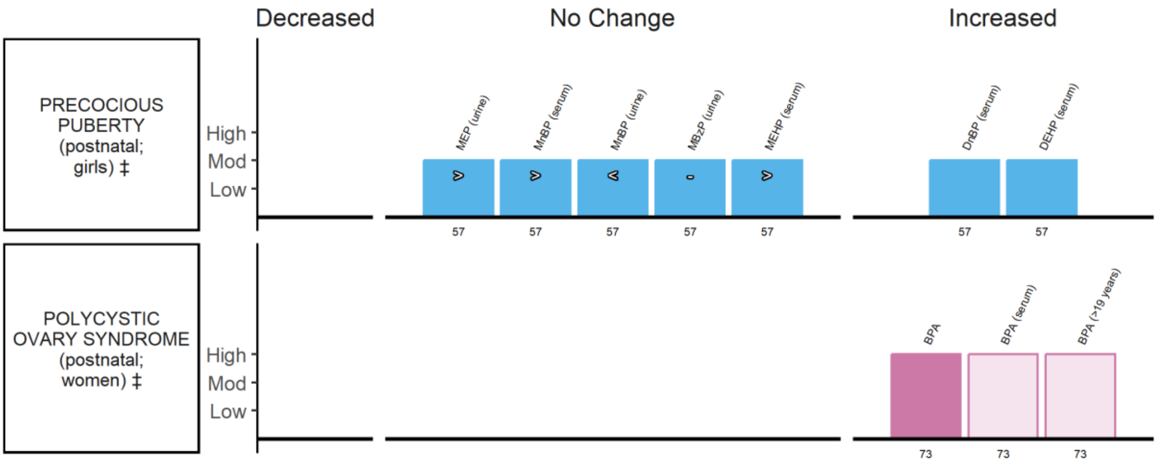

Suppl Fig S1: Harvest plot of exposure to plastic chemicals and precocious puberty in girls and polycystic ovarian syndrome in women. Plastic-associated chemicals included are bisphenol A (BPA) (pink); phthalate diesters diethylhexyl phthalate (DEHP) and dibutyl phthalate (DnBP) and phthalate mono ester metabolites (blue) encompassing monomethyl phthalate (MMP), monoethyl phthalate (MEP), mono-n-butyl phthalate (MnBP), monobenzyl phthalate (MBzP) and mono(2-ethylhexyl) phthalate (MEHP).

Exposure is measured on a continuous scale (‡) and standardised mean difference in plastic chemical concentration between females with the outcome and without represented.

Each bar represents the results from an individual meta-analysis. The citation is indicated by the number below each bar (see main manuscript references). The height of the bar represents the quality score of the review assessed using the AMSTAR tool. Moderate quality reflects a score of 5-8 and high quality a score of 9-11. Filled bars represent the primary analyses of each review. Bars have been assigned as an increase or decrease (columns) in the measure where the change is statistically significant. Remaining bars appearing under 'no change' indicate direction of effect as an increase (>), no clear trend (-) (the estimate of relative risk was 1 or regression coefficient or mean difference was 0), or decrease (<) in the measure.

1.7 Updated search results

Studies identified with the updated search strategy, identifying publications between August 2020 and August 2023:

1. Zhang H, Ben Y, Han Y, Zhang Y, Li Y, Chen X. Phthalate exposure and risk of diabetes mellitus: Implications from a systematic review and meta-analysis. *Environmental Research*. 2022;204:112109. doi:10.1016/j.envres.2021.112109
2. Zare Jeddi M, Soltanmohammadi R, Barbieri G, et al. To which extent are per-and poly-fluorinated substances associated to metabolic syndrome? *Reviews on Environmental Health*. 2022;37(2):211-228. doi:10.1515/reveh-2020-0144
3. Wang W, Hong X, Zhao F, Wu J, Wang B. The effects of perfluoroalkyl and polyfluoroalkyl substances on female fertility: A systematic review and meta-analysis. *Environmental Research*. 2023;216:114718. doi:10.1016/j.envres.2022.114718
4. Wang H, Wei K, Wu Z, et al. Association between per- and polyfluoroalkyl substances and semen quality. *Environ Sci Pollut Res*. 2022;30(10):27884-27894. doi:10.1007/s11356-022-24182-3
5. Steenland K, Hofmann JN, Silverman DT, Bartell SM. Risk assessment for PFOA and kidney cancer based on a pooled analysis of two studies. *Environment International*. 2022;167:107425. doi:10.1016/j.envint.2022.107425
6. Pourhassan B, Pourbabaki R, Omidi F, Kalantary S, Beigzadeh Z. Meta-Analysis of Body Concentration of Polychlorinated Biphenyls and Prostate Cancer. *Toxicol Ind Health*. 2022;38(11):757-772. doi:10.1177/07482337221129162
7. Hirke A, Varghese B, Varade S, Adela R. Exposure to endocrine-disrupting chemicals and risk of gestational hypertension and preeclampsia: A systematic review and meta-analysis. *Environmental Pollution*. 2023;317:120828. doi:10.1016/j.envpol.2022.120828
8. Zhong Q, Liu H li, Fu H, Niu Q shan, Wu H bing, Huang F. Prenatal exposure to phthalates with preterm birth and gestational age: A systematic review and meta-analysis. *Chemosphere*. 2021;282:130991. doi:10.1016/j.chemosphere.2021.130991
9. Yuan S, Du X, Liu H, et al. Association between bisphenol A exposure and thyroid dysfunction in adults: a systematic review and meta-analysis. *Toxicol Ind Health*. 2023;39(4):188-203. doi:10.1177/07482337231156284
10. Gui SY, Qiao JC, Xu KX, et al. Association between per- and polyfluoroalkyl substances exposure and risk of diabetes: a systematic review and meta-analysis. *J Expo Sci Environ Epidemiol*. 2023;33(1):40-55. doi:10.1038/s41370-022-00464-3
11. Zhang X, Xue L, Deji Z, et al. Effects of exposure to per- and polyfluoroalkyl substances on vaccine antibodies: A systematic review and meta-analysis based on epidemiological studies. *Environmental Pollution*. 2022;306:119442. doi:10.1016/j.envpol.2022.119442
12. Yao X, Geng S, Zhu L, Jiang H, Wen J. Environmental pollutants exposure and gestational diabetes mellitus: Evidence from epidemiological and experimental studies. *Chemosphere*. 2023;332:138866. doi:10.1016/j.chemosphere.2023.138866
13. Yao H, Fu Y, Weng X, et al. The Association between Prenatal Per- and Polyfluoroalkyl Substances Exposure and Neurobehavioral Problems in Offspring: A Meta-Analysis. *IJERPH*. 2023;20(3):1668. doi:10.3390/ijerph20031668
14. Wu Y, Wang J, Wei Y, et al. Maternal exposure to endocrine disrupting chemicals (EDCs) and preterm birth: A systematic review, meta-analysis, and meta-regression analysis. *Environmental Pollution*. 2022;292:118264. doi:10.1016/j.envpol.2021.118264
15. Yan D, Jiao Y, Yan H, Liu T, Yan H, Yuan J. Endocrine-disrupting chemicals and the risk of gestational diabetes mellitus: a systematic review and meta-analysis. *Environ Health*. 2022;21(1):53. doi:10.1186/s12940-022-00858-8
16. Goodrich JA, Walker DI, He J, et al. Metabolic Signatures of Youth Exposure to Mixtures of Per- and Polyfluoroalkyl Substances: A Multi-Cohort Study. *Environ Health Perspect*. 2023;131(2):027005. doi:10.1289/EHP11372
17. Castellini C, Muselli M, Parisi A, et al. Association between urinary bisphenol A concentrations and semen quality: A meta-analytic study. *Biochemical Pharmacology*. 2022;197:114896. doi:10.1016/j.bcp.2021.114896

18. Wu Y, Wang J, Wei Y, et al. Contribution of prenatal endocrine-disrupting chemical exposure to genital anomalies in males: The pooled results from current evidence. *Chemosphere*. 2022;286:131844. doi:10.1016/j.chemosphere.2021.131844
19. Wright JM, Lee AL, Rappazzo KM, Ru H, Radke EG, Bateson TF. Systematic review and meta-analysis of birth weight and PFNA exposures. *Environmental Research*. 2023;222:115357. doi:10.1016/j.envres.2023.115357
20. Wu Q, Li G, Zhao CY, Na XL, Zhang YB. Association between phthalate exposure and obesity risk: A meta-analysis of observational studies. *Environmental Toxicology and Pharmacology*. 2023;102:104240. doi:10.1016/j.etap.2023.104240
21. Wu M, Wang S, Weng Q, et al. Prenatal and postnatal exposure to Bisphenol A and Asthma: a systemic review and meta-analysis. *J Thorac Dis*. 2021;13(3):1684-1696. doi:10.21037/jtd-20-1550
22. Stratakis N, Rock S, La Merrill MA, et al. Prenatal exposure to persistent organic pollutants and childhood obesity: A systematic review and meta-analysis of human studies. *Obesity Reviews*. 2022;23(S1):e13383. doi:10.1111/obr.13383
23. Wang H, He H, Wei Y, Gao X, Zhang T, Zhai J. Do phthalates and their metabolites cause poor semen quality? A systematic review and meta-analysis of epidemiological studies on risk of decline in sperm quality. *Environ Sci Pollut Res*. 2022;30(12):34214-34228. doi:10.1007/s11356-022-24215-x
24. Moon S, Yu SH, Lee CB, Park YJ, Yoo HJ, Kim DS. Effects of bisphenol A on cardiovascular disease: An epidemiological study using National Health and Nutrition Examination Survey 2003–2016 and meta-analysis. *Science of The Total Environment*. 2021;763:142941. doi:10.1016/j.scitotenv.2020.142941
25. Martínez MÁ, Marquès M, Salas-Huetos A, Babio N, Domingo JL, Salas-Salvadó J. Lack of association between endocrine disrupting chemicals and male fertility: A systematic review and meta-analysis. *Environmental Research*. 2023;217:114942. doi:10.1016/j.envres.2022.114942
26. Yu Y, Quan X, Wang H, Zhang B, Hou Y, Su C. Assessing the health risk of hyperuricemia in participants with persistent organic pollutants exposure - a systematic review and meta-analysis. *Ecotoxicology and Environmental Safety*. 2023;251:114525. doi:10.1016/j.ecoenv.2023.114525
27. Uldbjerg CS, Koch T, Lim YH, et al. Prenatal and postnatal exposures to endocrine disrupting chemicals and timing of pubertal onset in girls and boys: a systematic review and meta-analysis. *Human Reproduction Update*. 2022;28(5):687-716. doi:10.1093/humupd/dmac013
28. Zhang M, Qiao J, Xie P, Li Z, Hu C, Li F. The Association between Maternal Urinary Phthalate Concentrations and Blood Pressure in Pregnancy: A Systematic Review and Meta-Analysis. *Metabolites*. 2023;13(7):812. doi:10.3390/metabo13070812
29. Zhang L, Liang J, Gao A. Contact to perfluoroalkyl substances and thyroid health effects: A meta-analysis directing on pregnancy. *Chemosphere*. 2023;315:137748. doi:10.1016/j.chemosphere.2023.137748
30. Zhang H, Chen S, Chen X, et al. Exposure to phthalate increases the risk of eczema in children: Findings from a systematic review and meta-analysis. *Chemosphere*. 2023;321:138139. doi:10.1016/j.chemosphere.2023.138139
31. Yu C, Lu J, Zhao J, et al. Maternal phthalate exposure during pregnancy and male reproductive disorders: a systematic review and metaanalysis. *Turk J Pediatr*. 2022;64(2):187-209. doi:10.24953/turkjped.2020.2060
32. Yang Z, Liu H yu, Yang Q yun, et al. Associations between exposure to perfluoroalkyl substances and birth outcomes: A meta-analysis. *Chemosphere*. 2022;291:132909. doi:10.1016/j.chemosphere.2021.132909
33. Luo Y, Deji Z, Huang Z. Exposure to perfluoroalkyl substances and allergic outcomes in children: A systematic review and meta-analysis. *Environmental Research*. 2020;191:110145. doi:10.1016/j.envres.2020.110145
34. Tang N, Wang D, Chen X, Zhang M, Lv W, Wang X. Maternal bisphenol A and triclosan exposure and allergic diseases in childhood: a meta-analysis of cohort studies. *Environ Sci Pollut Res*. 2022;29(55):83389-83403. doi:10.1007/s11356-022-21575-2
35. Mérida DM, Moreno-Franco B, Marquès M, León-Latre M, Laclaustra M, Guallar-Castillón P. Phthalate exposure and the metabolic syndrome: A systematic review and meta-analysis. *Environmental Pollution*. 2023;333:121957. doi:10.1016/j.envpol.2023.121957
36. Wang X, Wang L lu, Tian Y kuan, et al. Association between exposures to phthalate metabolites and preterm birth and spontaneous preterm birth: A systematic review and meta-analysis. *Reproductive Toxicology*. 2022;113:1-9. doi:10.1016/j.reprotox.2022.07.006
37. Wang J, Zhang J, Fan Y, et al. Association between per- and polyfluoroalkyl substances and risk of gestational diabetes mellitus. *International Journal of Hygiene and Environmental Health*. 2022;240:113904. doi:10.1016/j.ijheh.2021.113904

38. Sun M, Cao X, Wu Y, Shen L, Wei G. Prenatal exposure to endocrine-disrupting chemicals and thyroid function in neonates: A systematic review and meta-analysis. *Ecotoxicology and Environmental Safety*. 2022;231:113215. doi:10.1016/j.ecoenv.2022.113215
39. Taheri E, Riahi R, Rafiei N, Fatehizadeh A, Iqbal HMN, Hosseini SM. Bisphenol A exposure and abnormal glucose tolerance during pregnancy: systematic review and meta-analysis. *Environ Sci Pollut Res*. 2021;28(44):62105-62115. doi:10.1007/s11356-021-16691-4
40. Odutola MK, Benke G, Fritschi L, Giles GG, van Leeuwen MT, Vajdic CM. A systematic review and meta-analysis of occupational exposures and risk of follicular lymphoma. *Environmental Research*. 2021;197:110887. doi:10.1016/j.envres.2021.110887
41. Mehri F, Bashirian S, khazaei S, Jenabi E. Association between pesticide and polychlorinated biphenyl exposure during pregnancy and autism spectrum disorder among children: a meta-analysis. *Clin Exp Pediatr*. 2021;64(6):286-292. doi:10.3345/cep.2020.00864
42. Qu A, Cao T, Li Z, et al. The association between maternal perfluoroalkyl substances exposure and early attention deficit hyperactivity disorder in children: a systematic review and meta-analysis. *Environ Sci Pollut Res*. 2021;28(47):67066-67081. doi:10.1007/s11356-021-15136-2
43. Namat A, Xia W, Xiong C, et al. Association of BPA exposure during pregnancy with risk of preterm birth and changes in gestational age: A meta-analysis and systematic review. *Ecotoxicology and Environmental Safety*. 2021;220:112400. doi:10.1016/j.ecoenv.2021.112400
44. Lan L, Wei H, Chen D, et al. Associations between maternal exposure to perfluoroalkylated substances (PFASs) and infant birth weight: a meta-analysis. *Environ Sci Pollut Res*. 2023;30(38):89805-89822. doi:10.1007/s11356-023-28458-0
45. Gao X, Ni W, Zhu S, et al. Per- and polyfluoroalkyl substances exposure during pregnancy and adverse pregnancy and birth outcomes: A systematic review and meta-analysis. *Environmental Research*. 2021;201:111632. doi:10.1016/j.envres.2021.111632
46. Pan K, Xu J, Long X, Yang L, Huang Z, Yu J. The relationship between perfluoroalkyl substances and hypertension: A systematic review and meta-analysis. *Environmental Research*. 2023;232:116362. doi:10.1016/j.envres.2023.116362
47. Liu Z, Lu Y, Zhong K, Wang C, Xu X. The associations between endocrine disrupting chemicals and markers of inflammation and immune responses: A systematic review and meta-analysis. *Ecotoxicology and Environmental Safety*. 2022;234:113382. doi:10.1016/j.ecoenv.2022.113382
48. Moreno-Gómez-Toledano R, Arenas MI, Vélez-Vélez E, et al. Bisphenol a Exposure and Kidney Diseases: Systematic Review, Meta-Analysis, and NHANES 03–16 Study. *Biomolecules*. 2021;11(7):1046. doi:10.3390/biom11071046
49. Liu G, Cai W, Liu H, Jiang H, Bi Y, Wang H. The Association of Bisphenol A and Phthalates with Risk of Breast Cancer: A Meta-Analysis. *IJERPH*. 2021;18(5):2375. doi:10.3390/ijerph18052375
50. Jin S, Cui S, Xu J, Zhang X. Associations between prenatal exposure to phthalates and birth weight: A meta-analysis study. *Ecotoxicology and Environmental Safety*. 2023;262:115207. doi:10.1016/j.ecoenv.2023.115207
51. Meng H, Zhou Y, Jiang Y. Association of bisphenol A with puberty timing: a meta-analysis. *Reviews on Environmental Health*. 2021;36(4):459-466. doi:10.1515/reveh-2020-0091
52. Liu B, Zhu L, Wang M, Sun Q. Associations between Per- and Polyfluoroalkyl Substances Exposures and Blood Lipid Levels among Adults—A Meta-Analysis. *Environ Health Perspect*. 2023;131(5):056001. doi:10.1289/EHP11840
53. Liao YC, Xu YJ, Chen JK, et al. Sex differences in children's cognitive functions and phthalates exposure: a meta-analysis. *Pediatr Res*. 2023;94(5):1609-1618. doi:10.1038/s41390-023-02672-5
54. Lee DW, Lim HM, Lee JY, et al. Prenatal exposure to phthalate and decreased body mass index of children: a systematic review and meta-analysis. *Sci Rep*. 2022;12(1):8961. doi:10.1038/s41598-022-13154-9
55. Jiang H, Liu H, Liu G, et al. Associations between Polyfluoroalkyl Substances Exposure and Breast Cancer: A Meta-Analysis. *Toxics*. 2022;10(6):318. doi:10.3390/toxics10060318
56. Hu CY, Qiao JC, Gui SY, Xu KX, Dzhambov AM, Zhang XJ. Perfluoroalkyl and polyfluoroalkyl substances and hypertensive disorders of pregnancy: A systematic review and meta-analysis. *Environmental Research*. 2023;231:116064. doi:10.1016/j.envres.2023.116064
57. Gui SY, Chen YN, Wu KJ, et al. Association Between Exposure to Per- and Polyfluoroalkyl Substances and Birth Outcomes: A Systematic Review and Meta-Analysis. *Front Public Health*. 2022;10:855348. doi:10.3389/fpubh.2022.855348

58. Gao X, Yan D, Li G, Wei Y, He H, Zhai J. Polychlorinated biphenyls and risk of metabolic syndrome and comparison with the risk of diabetes: A systematic review and meta-analysis. *Science of The Total Environment*. 2023;900:165773. doi:10.1016/j.scitotenv.2023.165773
59. Gao H, Chen L wen, Gong C, et al. The associations between prenatal phthalate exposure and childhood glycolipid metabolism and blood pressure: An updated systematic review and a pilot meta-analysis of prospective cohort studies. *Ecotoxicology and Environmental Safety*. 2023;262:115157. doi:10.1016/j.ecoenv.2023.115157
60. Gang N, Van Allen K, Villeneuve PJ, MacDonald H, Bruin JE. Sex-specific Associations Between Type 2 Diabetes Incidence and Exposure to Dioxin and Dioxin-like Pollutants: A Meta-analysis. *Front Toxicol*. 2022;3:685840. doi:10.3389/ftox.2021.685840
61. Dev P, Chakravarty K, Pandey M, et al. Effect of persistent organic pollutants in patients with ischemic stroke and all stroke: A systematic review and meta-analysis. *Toxicology*. 2023;494:153567. doi:10.1016/j.tox.2023.153567
62. Gao H, Chen D, Zang M. Association between phthalate exposure and insulin resistance: a systematic review and meta-analysis update. *Environ Sci Pollut Res*. 2021;28(40):55967-55980. doi:10.1007/s11356-021-16252-9
63. Frigerio G, Ferrari CM, Fustinoni S. Prenatal and childhood exposure to per-/polyfluoroalkyl substances (PFASs) and its associations with childhood overweight and/or obesity: a systematic review with meta-analyses. *Environ Health*. 2023;22(1):56. doi:10.1186/s12940-023-01006-6
64. Fiolet T, Mahamat-Saleh Y, Frenoy P, Kvaskoff M, Romana Mancini F. Background exposure to polychlorinated biphenyls and all-cause, cancer-specific, and cardiovascular-specific mortality: A systematic review and meta-analysis. *Environment International*. 2021;154:106663. doi:10.1016/j.envint.2021.106663
65. Duque-Cartagena T, Mundstock E, Dala Bernardina Dalla M, Vontobel Padoin A, Cañon-Montañez W, Mattiello R. The role of environmental pollutants in body composition: Systematic review and meta-analysis. *Environmental Research*. 2023;228:115840. doi:10.1016/j.envres.2023.115840
66. Deji Z, Liu P, Wang X, Zhang X, Luo Y, Huang Z. Association between maternal exposure to perfluoroalkyl and polyfluoroalkyl substances and risks of adverse pregnancy outcomes: A systematic review and meta-analysis. *Science of The Total Environment*. 2021;783:146984. doi:10.1016/j.scitotenv.2021.146984
67. Costello E, Rock S, Stratakis N, et al. Exposure to per- and Polyfluoroalkyl Substances and Markers of Liver Injury: A Systematic Review and Meta-Analysis. *Environ Health Perspect*. 2022;130(4):046001. doi:10.1289/EHP10092
68. Conforti A, Carbone L, Simeon V, et al. Unravelling the link between phthalate exposure and endometriosis in humans: a systematic review and meta-analysis of the literature. *J Assist Reprod Genet*. 2021;38(10):2543-2557. doi:10.1007/s10815-021-02265-3
69. Bartell SM, Vieira VM. Critical review on PFOA, kidney cancer, and testicular cancer. *Journal of the Air & Waste Management Association*. 2021;71(6):663-679. doi:10.1080/10962247.2021.1909668
70. Abellan A, Mensink-Bout SM, Garcia-Esteban R, et al. In utero exposure to bisphenols and asthma, wheeze, and lung function in school-age children: a prospective meta-analysis of 8 European birth cohorts. *Environment International*. 2022;162:107178. doi:10.1016/j.envint.2022.107178
71. Crawford L, Halperin SA, Dzierlenga MW, et al. Systematic review and meta-analysis of epidemiologic data on vaccine response in relation to exposure to five principal perfluoroalkyl substances. *Environment International*. 2023;172:107734. doi:10.1016/j.envint.2023.107734
72. Cong X, Liu Q, Li W, et al. Systematic review and meta-analysis of breast cancer risks in relation to 2,3,7,8-tetrachlorodibenzo-p-dioxin and per- and polyfluoroalkyl substances. *Environ Sci Pollut Res*. 2023;30(37):86540-86555. doi:10.1007/s11356-023-28592-9
73. Choi J, Kim JY, Lee HJ. Human Evidence of Perfluorooctanoic Acid (PFOA) Exposure on Hepatic Disease: A Systematic Review and Meta-Analysis. *IJERPH*. 2022;19(18):11318. doi:10.3390/ijerph191811318
74. Cao T, Qu A, Li Z, et al. The relationship between maternal perfluoroalkylated substances exposure and low birth weight of offspring: a systematic review and meta-analysis. *Environ Sci Pollut Res*. 2021;28(47):67053-67065. doi:10.1007/s11356-021-15061-4
75. Bräuner EV, Lim YH, Koch T, et al. Endocrine Disrupting Chemicals and Risk of Testicular Cancer: A Systematic Review and Meta-analysis. *The Journal of Clinical Endocrinology & Metabolism*. Published online July 16, 2021:dgab523. doi:10.1210/clinem/dgab523
76. Abdullah Soheimi SS, Abdul Rahman A, Abd Latip N, Ibrahim E, Sheikh Abdul Kadir SH. Understanding the Impact of Perfluorinated Compounds on Cardiovascular Diseases and Their Risk Factors: A Meta-Analysis Study. *IJERPH*. 2021;18(16):8345. doi:10.3390/ijerph18168345
