# Supplementary material for: An Umbrella Review of Meta-Analyses Evaluating Associations between Human Health and Exposure to Major Classes of Plastic-Associated Chemicals
Source: Ann Glob Health. 2024 Aug 19;90(1):52. doi: 10.5334/aogh.4459 (PMC11342836; doi:10.5334/aogh.4459)
Supplement: Supplementary File 2. — Characteristics of included reviews. [file agh-90-1-4459-s2.pdf]

Supplementary file 2 – Characteristics of included reviews

2.1 Birth outcomes

Study details	Appraisal details	Participants	Plastic exposure	Health outcomes	Findings	Subgroup Findings	AMSTAR score
**Zhang et al., 2020 [48] - Associations between phthalate exposure and risk of spontaneous pregnancy loss: A systematic review and meta-analysis. No COI declared**							
**Last search**<br>Apr-20<br><br>**Study types**<br>n=4 was case control; n= 4 were prospective cohort studies<br><br>**Included studies in the review** = 8<br><br>**Included studies in the meta-analysis =** 8	Newcastle-Ottawa Scale. All studies scored between 7-9 and deemed to be high-quality by the authors.	Reproductive age women un-derwent spontaneous pregnancy loss). Reproductive loss occurred from conception to 27 weeks, however, n=3 studies were not limited by gestational week. Control group (defined as: reproductive age women without pregnancy loss)<br><br>N = 4713 (651 cases and 4063 controls)	**Type**<br>MMP<br><br>**Route**<br>In-utero<br><br>**Measure**<br>Urinary phthalates levels (Measurements noted: µg/g, ng/mL, µg/L)<br><br>**Exposure time**<br>During pregnancy; non-specific about mothers' exposure.	Spontaneous pregnancy loss<br>0R with 95% CI	Overall analyses omitted due to unit of analysis error	No relationship between MMP and spontaneous pregnancy loss with reproductive women OR: 1.54 (95% 0.91-2.60, 5 studies, participants unknown)	7
			**Type**<br>MBP<br><br>**Route**<br>In-utero<br><br>**Measure**<br>Urinary phthalates levels (Measurements noted: µg/g, ng/mL, µg/L)<br><br>**Exposure time**	Spontaneous pregnancy loss<br>0R with 95% CI		Exposure to MBP was positively significant associated with spontaneous pregnancy loss with reproductive women OR:1.34 (95%1.04-1.72 7 studies, participants unknown)	

Study details	Appraisal details	Participants	Plastic exposure	Health outcomes	Findings	Subgroup Findings	AMSTAR score
			During pregnancy; non-specific about mothers' exposure.				
			**Type**<br>MEP<br><br>**Route**<br>In-utero<br><br>**Measure**<br>Urinary phthalates levels (Measurements not noted)<br><br>**Exposure time**<br>During pregnancy; non-specific about mothers' exposure.	Spontaneous pregnancy loss<br>0R with 95% CI		No relationship between MEP and spontaneous pregnancy loss with reproductive women OR: 1.30(95% 0.84-2.03 7 studies, participants unknown)	
			**Type**<br>MiBP<br><br>**Route**<br>In-utero<br><br>**Measure**<br>Urinary phthalates levels (Measurements not noted)<br><br>**Exposure time**<br>During pregnancy; non-specific about mothers' exposure.	Spontaneous pregnancy loss<br>0R with 95% CI		No relationship between MiBP and spontaneous pregnancy loss with reproductive women OR: 1.31(95% 0.69-2.49 4 studies, participants unknown)	
			**Type**<br>MBzP	Spontaneous pregnancy loss<br>0R with 95% CI		No relationship between MBzP and spontaneous pregnancy loss with reproductive women OR:	

Study details	Appraisal details	Participants	Plastic exposure	Health outcomes	Findings	Subgroup Findings	AMSTAR score
			**Route**<br>In-utero<br><br>**Measure**<br>Urinary phthalates levels (Measurements not noted)<br><br>**Exposure time**<br>During pregnancy; non-specific about mothers' exposure.			1.10 (95% 0.74-1.64, 4 studies, participants unknown)	
			**Type**<br>MEHP<br><br>**Route**<br>In-utero<br><br>**Measure**<br>Urinary phthalates levels (Measurements not noted)<br><br>**Exposure time**<br>During pregnancy; non-specific about mothers' exposure.	Spontaneous pregnancy loss 0R with 95% CI		Exposure to MEHP was positively significant associated with spontaneous pregnancy loss with reproductive women OR:1.57 (95% 1.29-1.90 7 studies, participants unknown)	
			**Type**<br>MEHHP<br><br>**Route**<br>In-utero<br><br>**Measure**	Spontaneous pregnancy loss 0R with 95% CI		Exposure to MEHHP was positively significant associated with spontaneous pregnancy loss with reproductive women OR:1.59 (95% 1.23-2.07 6 studies, participants unknown)	

Study details	Appraisal details	Participants	Plastic exposure	Health outcomes	Findings	Subgroup Findings	AMSTAR score
			Urinary phthalates levels (Measurements not noted)<br><br>**Exposure time**<br>During pregnancy; non-specific about mothers' exposure.				
			**Type**<br>MEOHP<br><br>**Route**<br>In-utero<br><br>**Measure**<br>Urinary phthalates levels (Measurements not noted)<br><br>**Exposure time**<br>During pregnancy; non-specific about mothers' exposure.	Spontaneous pregnancy loss 0R with 95% CI		Exposure to MEOHP was positively significant associated with spontaneous pregnancy loss with reproductive women OR:1.47 (95% 1.15-1.89 6 studies, participants unknown)	
			**Type**<br>MECCP<br><br>**Route**<br>In-utero<br><br>**Measure**<br>Urinary phthalates levels (Measurements not noted)<br><br>**Exposure time**	Spontaneous pregnancy loss 0R with 95% CI		No relationship between MECCP and spontaneous pregnancy loss with reproductive women OR: 1.08 (95% 0.80-1.46 3 studies, participants unknown)	

Study details	Appraisal details	Participants	Plastic exposure	Health outcomes	Findings	Subgroup Findings	AMSTAR score
			During pregnancy; non-specific about mothers' exposure.				
			**Type**<br>ΣDEHP<br><br>**Route**<br>In-utero<br><br>**Measure**<br>Urinary phthalates levels (Measurements not noted)<br><br>**Exposure time**<br>During pregnancy; non-specific about mothers' exposure.	Spontaneous pregnancy loss<br>0R with 95% CI		Exposure to ΣDEHP (metabolite included: MEHP, MEHHP, MEOHP & MECPP) was positively significant associated with spontaneous pregnancy loss with reproductive women OR:1.79 (95% 1.27-2.53 3 studies, participants unknown)	
**Zhong et al., 2020 [52] - Association of prenatal exposure to phenols and parabens with birth size: A systematic review and meta-analysis. No COI declared.**							
**Last search**<br>Jul-19<br><br>**Study types**<br>case-control (n=1), cohort (n=20)<br><br>**Included studies in the review** = 21<br><br>**Included studies in**	No critical appraisal	Pregnant women and their infants<br><br>N = 11,497	**Type**<br>Bisphenol A (BPA)<br><br>**Route**<br>Maternal (prenatal)<br><br>**Measure**<br>Maternal urine (units NR)<br><br>**Exposure time**<br>Prenatal	Birth weight<br>Beta Coefficient and 95% CI	No association between prenatal BPA exposure and neonatal birthweight in infants (b= -0.049g, 95%CI: -0.199, 0.101; 9 studies, 4636 participants).	urine was collected in the third trimester of pregnancy (p-value for heterogeneity = 0.051; I2 = 54.6%).	5
				Birth length<br>Beta Coefficient and 95% CI	No association between prenatal BPA exposure and neonatal birth length in infants (b= 0.058cm, 95%CI: -0.072, 0.188; 9 studies, 4636 participants).		
				Head circumference<br>Beta Coefficient and 95% CI	No association between prenatal BPA exposure and neonatal birth length in infants (b=-0.004cm, 95%CI: -0.119, 0.111; 9 studies, 4636 participants).		

Study details	Appraisal details	Participants	Plastic exposure	Health outcomes	Findings	Subgroup Findings	AMSTAR score
**the meta-analysis** = 9				Gestational age<br>Beta Coefficient and 95% CI	No association between prenatal BPA exposure and gestational age (b=-0.032 weeks, 95%CI: -0.163, 0.10; 9 studies, 4636 participants).		
**Zhao et al., 2017 [45] - Correlation between Prenatal Exposure to Polybrominated Diphenyl Ethers (PBDEs) and Infant Birth Outcomes: A Meta-Analysis and an Experimental Study. no COI declared.**							
**Last search**<br>Jun-16<br><br>**Study types**<br>cross-sectional<br><br>**Included studies in the review** = 7<br><br>**Included studies in the meta-analysis** = 7	A cross-sectional assessment tool - not named (studies of high quality)	infants (at birth)<br><br>N = 1,332	**Type**<br>polybrominated diphenyl ethers (PBDEs)<br><br>**Route**<br>via mother<br><br>**Measure**<br>ng/g lipid<br><br>**Exposure time**<br>pre-natal	infant birth outcomes (IBO) - birth weight (g)<br>beta coefficient (b)	Exposure to pre-natal PBDE was negatively associated with birthweight in infants (b=-50.56, 95%CI: -95.91,-5.28; 7 studies, 1332 participants)	No association between pre-natal PBDE and low birthweight in infant females (b=-50.598, 95%CI: -95.914,-5.252; 2 studies, 265 participants)	**9**
						Exposure to pre-natal PBDE was negatively associated with birthweight in infant males (b=-121.456, 95%CI: -230.139-12.773; 2 studies, 296 participants)	
						No association between pre-natal PBDE and birthweight in the remaining studies that grouped infant females and males (b=-54.388, 95%CI: -115.982,7.206; 3 studies, 771 participants)	
			**Type**<br>2,20 ,4,40 - Tetrabromodiphenyl ether (BDE-47)	infant birth outcomes (IBO) - birth weight (g)<br>beta coefficient (b)	No association between pre-natal PBDE-47 and birthweight in infants (b=-41.54, 95%CI: -90.35,7.28; 4 studies, 768 participants)	No subgroup analysis	
			**Type**	infant birth outcomes (IBO) - birth weight (g)<br>beta coefficient (b)	No association between pre-natal PBDE-99 and birthweight in infants (b=-29.78, 95%CI: -	No subgroup analysis	

Study details	Appraisal details	Participants	Plastic exposure	Health outcomes	Findings	Subgroup Findings	AMSTAR score
			2,20 ,4,40 ,5-Pentabromodiphenyl ether (BDE-99)		95.09,35.53; 4 studies, 768 participants)		
			**Type** 2,20 ,4,40 ,6-Pentabromodiphenyl ether (BDE-100)	infant birth outcomes (IBO) - birth weight (g) beta coefficient (b)	No association between pre-natal PBDE-100 and birthweight in infants (b=-28.55, 95%CI: -91.19,34.10; 4 studies, 768 participants)	No subgroup analysis	
			**Type** 2,20 ,4,40, 5,50 -Hexabromodiphenyl ether (BDE-153)	infant birth outcomes (IBO) - birth weight (g) beta coefficient (b)	No association between pre-natal PBDE-153 and birthweight in infants (b=-41.22, 95%CI: -102.73,20.29; 4 studies, 768 participants)	No subgroup analysis	
			**Type** polybrominated diphenyl ethers (PBDEs)	infant birth outcomes (IBO) - birth length (cm) beta coefficient (b)	No association between pre-natal PBDE and birth length in infants (b=-0.33, 95%CI: -0.74,0.07; 3 studies, 632 participants)	No subgroup analysis	
**Johnson et al., 2014 [44] - The Navigation Guide - Evidence-Based Medicine Meets Environmental Health: Systematic Review of Human Evidence for PFOA Effects on Foetal Growth. No COI declared**							
**Last search** 2017 (actual date NR)<br><br>**Study types** Unspecified<br><br>**Included studies in the review** = 47<br>**Included studies in**	GRADE - individual studies not critically appraised.	Children (<18 years old)<br><br>N = 3,484	**Type** PFOA<br><br>**Route** Postnatal (aged 6-18 years)<br><br>**Measure** Urine (units unspecified)<br><br>**Exposure time** Unspecified	Birth Weight Beta coefficient effect size (natural units)	Prenatal PFOA exposure was associated with a reduced birth weight (b = -18.9 grams, 95% CI: -29.8, -7.9 grams, (p-value not specified); 9 studies; 4149 participants).		**10**
				Birth Length Beta coefficient effect size (natural units)	Prenatal PFOA exposure was associated with a reduced birth length (b = -0.06 cm, 95% CI: -0.09, -0.02 cm, (p-value not specified); 5 studies; 2853 participants).	No sub-group analysis	
				Ponderal Index	No association between prenatal PFOA exposure and ponderal	No sub-group analysis	

Study details	Appraisal details	Participants	Plastic exposure	Health outcomes	Findings	Subgroup Findings	AMSTAR score
**the meta-analysis** = 5				Beta coefficient effect size (natural units)	index (b = -0.01, 95% CI: - 0.03, 0.01, (p-value not specified); 4 studies, 1510 participants).		
				Head circumference<br>Beta coefficient effect size (natural units)	No association between prenatal PFOA exposure and head circumference (b=-0.03 cm, 95% CI: -0.08, 0.01, (p-value not specified); 4 studies, 2497 participants).	No sub-group analysis	
**Hu et al., 2018 [46] -The association between prenatal bisphenol A exposure and birth weight: a meta-analysis. No COI declared**							
**Last search**<br>Aug-17<br><br>**Study types**<br>Case-control (n=2) and cohort (n=12)<br><br>**Included studies in the review** = 14<br><br>**Included studies in the meta-analysis** = 8	Office of Health Assessment (OHAT) critical appraisal tool. In summary, there were 2 (25%) studies scored as high-quality, 6 (75%) studies scored as medium quality.	Pregnant women and their infants.<br><br>N = 6,208	**Type**<br>Bisphenol A (BPA)<br><br>**Route**<br>in utero/ maternal exposure<br><br>**Measure**<br>ng/ml or µg/g Maternal urine sample/ maternal blood sample/ amniotic fluid<br><br>**Exposure time**<br>NR	Birth Weight<br>Pooled effect size and 95% CI	No association between prenatal BPA exposure and birth weight in infants (ES=4.42g, 95%CI: -8.83, 17.67; 8 studies, 2876 participants).	No association between prenatal BPA exposure measured during the first trimester and birth weight in infants (ES=44.41g, 95%CI: -113.45, 202.67; 2 studies, 395 participants). No association between prenatal BPA exposure measured during the second trimester and birth weight in infants (ES =37.89g, 95%CI: -209.68, 285.46; 2 studies, 292 participants). No association between prenatal BPA exposure measured during the third trimester and birth weight in infants (ES= -34.38g, 95%CI: -16.69, 85.49; 3 studies, 1512 participants).	8
**Golestanzadeh et al., 2019 [54] - Association of exposure to phthalates with cardiometabolic risk factors in children and adolescents: a systematic review and meta-analysis. No COI declared.**							

Study details	Appraisal details	Participants	Plastic exposure	Health outcomes	Findings	Subgroup Findings	AMSTAR score
**Last search** Dec-18<br><br>**Study types** cohort (n=17) cross sectional (n=15) case-control (n=3)<br><br>**Included studies in the review** = 35<br><br>**Included studies in the meta-analysis =** 23	STROBE checklist. Note this is a reporting guideline not a critical appraisal tool. The quality of the included observational studies was good and most of them gained a high score.	Children (≤ 18 years)<br><br>N = 24,943	**Type** Phthalates<br><br>**Route** Unspecified<br><br>**Measure** Urine and serum (units unspecified)<br><br>**Exposure time** Prenatal	Birth weight<br>Pooled correlation coefficients and 95% CI	Overall analyses omitted due to unit of analysis error	For LMWP:<br>Exposure to MEP was negatively associated with birthweight (z= -10.1, 95%CI: − 18.57, − 1.6; 3 studies, 4775 participants) but not for MMP (z = -0.05, 95%CI: -20.99, 20.90; 2 studies,4476 participants), MBP (z = 0.05, 95%CI: -0.51, 0.62; 4 studies, 5296 participants) and MiBP (z = -0.11, 95%CI: -0.87, 0.65; 2 studies, 820 participants).<br>For HMWP: No significant associations with birth weight in the subgroup analysis of HMWPs; MECPP (z = 16.15, 95%CI:−18.3, 50.58; 3 studies, 1822 participants), MEHHP (z = −0.16, 95%CI:−1.27, 0.9; 5 studies, 5424 participants), MEOHP (z = − 0.39, 95%CI:−12.9, 12.13; 5 studies, 5424 participants), MEHP (z = − 0.79, 95%CI:− 3.84 2.62; 4 studies, 4461 participants), MBzP (z = −2.38, 95%CI:− 9.20 - 3.53; 3 studies, 4294 participants), DEHP (z = 3.85, 95%CI: −17.8, 25.6; 4604 participants).	5

Govarts et al., 2012 [50] - Birth Weight and Prenatal Exposure to Polychlorinated Biphenyls (PCBs) and Dichlorodiphenyldichloroethylene (DDE): A Meta-analysis within 12 European Birth Cohorts. No COI declared.

Study details	Appraisal details	Participants	Plastic exposure	Health outcomes	Findings	Subgroup Findings	AMSTAR score
**Last search**<br>Unspecified<br><br>**Study types**<br>Cohort<br><br>**Included studies in the meta-analysis =**<br>12	unspecified	Infant-mother pairs<br><br>N = 7,762	**Type**<br>Polychlorinated biphenyl (PCB)<br><br>**Route**<br>Maternal/prenatal<br><br>**Measure**<br>Cord plasma or serum/ maternal serum or blood/breast milk (ng/L or ng/g fat)<br><br>**Exposure time**<br>Prenatal	Birth weight<br>Beta Coefficient and 95% CI	Exposure to PCBs was associated with lower birth weight (b= -0.15, -0.24, -0.05; 12 studies, 7666 participants). 150g reduction per 1-µg/L increase in PCB-153 cord serum levels	No subgroup analysis	3
**Zou et al., 2019 [53] - Neonatal Weight and Prenatal Exposure to Polychlorinated Biphenyls: A Meta-Analysis. No COI declared.**							

**Last search**<br>Jun-18<br><br>**Study types**<br>Not reported<br><br>**Included studies in the review** =<br>7<br><br>**Included studies in the meta-analysis** = 7	not reported	infants (at birth)<br><br>N = 8,054	**Type**<br>Polychlorinated biphenyls (PCBs)<br><br>**Route**<br>Via mother<br><br>**Measure**<br>Maternal serum/cord blood<br><br>**Exposure time**<br>Unspecified	neonatal birth weight (g)<br>beta coefficient (b) and 95%CI	Exposure to PCB throughout pregnancy was negatively associated with infant birth weight (b= -0.59, 95%CI: -0.85,-0.34; 7 studies, 8054 participants)	Each trimester was negatively associated with infant birthweight<br><br>First trimester exposure (β -0.386, 95% CI: -0.559, -0.213; 3 studies, participants unspecified)<br>Second trimester exposure (β -0.494, 95% CI: -0.660, -0.328,2 studies, participants unspecified)<br>Third trimester exposure (β -0.657, 95%CI: -0.905, -0.410, 6 studies, participants unspecified)<br>Exposure to PCB in subgroup of cord serum measurement was negatively associated with infant birthweight β= -0.833, 95%CI: -1.695, -0.029; 2 studies, 1004 participants<br><br>Exposure to PCB in subgroup of maternal serum measurement was negatively associated with infant birthweight β= -0.504, 95%CI: -0.785 to -0.223; 5 studies, 7050 participants	4

Study details	Appraisal details	Participants	Plastic exposure	Health outcomes	Findings	Subgroup Findings	AMSTAR score
**Negri et al., 2017 [47] - Exposure to PFOA and PFOS and foetal growth: a critical merging of toxicological and epidemiological data. No COIs declared**							
**Last search**<br>Nov-15<br><br>**Study types**<br>Cross-sectional (n=4)<br>Prospective Cohort (n=9)<br>Case Control (n=3)<br><br>**Included studies in the review** = 16<br><br>**Included studies in the meta-analysis** = 9	Two researchers independently assessed the methodological aspects of each study using a modification of the "Newcastle-Ottawa Quality Assessment Scale".<br><br>For the four cross-sectional studies, the evaluation ranged between 4 and 5 out of a total of 6 points. Potential bias could emerge from incomplete control of confounding and lack of representativen	Children born to pregnant mothers studied during the reproductive / developmental time period (before and / or during pregnancy. Mothers and children exposed to PFFA as assessed using a biological sample<br><br>N = 8,335	**Type**<br>Perfluoralkyl acids (PFAA)<br>Which include:<br>Perfluorooctanoic acid (PFOA)<br>Perfluorooctane sulfonic acid (PFOS)<br><br>**Route**<br>Unspecified, but detection limited to maternal or umbilical cord serum, plasma or whole blood or maternal milk<br><br>**Measure**<br>Authors have presented both natural units as mean PFAA (ng/mL) and then the natural log<br><br>**Exposure time**<br>Unspecified<br>But exposure was prenatal	Birth Weight<br>ONLY CONSIDERING PFOA<br>Beta coefficient effect size (natural units; untransformed)	Prenatal exposure to PFOA was associated with a decrease in birthweight (b = -12.8 grams, 95% CI: -23.21, -2.38grams, (p-value not provided); 12 studies; 6501 participants); untransformed data; estimated linear regression coefficient range -213 to 154g for an increase of 1 loge ng/mL PFOA	No association between PFOA and birthweight via maternal blood samples collected in the first to second trimester using untransformed data (b -10.5, 95%CI: -23.6 to 2.6; 6 studies, participants unspecified) and third trimester (b -20; 95%CI: -52.1 to 12.1; 2 studies, participants unspecified), and in via cord samples using untransformed data (b -35.3, 95%CI: -101.0 to 30.7; 4 studies, participants unspecified)	8

Study details	Appraisal details	Participants	Plastic exposure	Health outcomes	Findings	Subgroup Findings	AMSTAR score
	ess of the study. Population. Among the 12 cohort studies (authors included case-control as a 'cohort' study), the evaluation ranged from 3/7 to 7/7. Again, potential risk of bias could mostly derive from lack of control of confounding and unclear adequacy of follow-up of women from recruitment to delivery.		**Type**<br>Perfluoralkyl acids (PFAA)<br>Which include:<br>Perfluorooctanoic acid (PFOA)<br>Perfluorooctane sulfonic acid (PFOS)<br><br>**Route**<br>Unspecified, but detection limited to maternal or umbilical cord serum, plasma or whole blood or maternal milk<br><br>**Measure**<br>Authors have presented both natural units as mean PFAA (ng/mL) and then the natural log<br><br>**Exposure time**<br>Unspecified<br>But exposure was prenatal	Birth Weight<br>ONLY CONSIDERING PFOA<br>Beta coefficient effect size (natural log; transformed)	Prenatal exposure to PFOA was associated with a decrease in birthweight per 1 log(ng/ml) PFOA (-27.12grams, 95% CI: -50.64, -3.60grams, (p-value not provided); 9 studies; 3844 participants); transformed data; estimated LRC ranged from 142 to 5 g for an increase of 1 loge ng/mL PFOA,	No association between prenatal PFOA exposure and birthweight via maternal blood samples collected in the first to second trimester using transformed data (b-10.6, 95%CI: -43.2 to22.0; 4 studies, participants unspecified). However, PFOA exposure was associated with decreased birthweight in maternal blood samples collected in the third trimester (b -51.0, 95%CI: -86.6 to -15.5; 3 studies, participants unspecified).<br>No association between prenatal PFOA exposure and birthweight via cord samples using transformed data (b -24.4, 95%CI: -66.3 to 18.2; 3 studies)	
			**Type**<br>Perfluoralkyl acids (PFAA)<br>Which include:<br>Perfluorooctanoic acid (PFOA)<br>Perfluorooctane sulfonic acid (PFOS)<br><br>**Route**	Birth Weight<br>ONLY CONSIDERING PFOS<br>Beta coefficient effect size (natural log; transformed)	Prenatal exposure to PFOS was associated with a decrease in birthweight per 1 log(ng/ml) PFOS (b = -46.09, 95% CI: -80.33, -11.85grams, (p-value not provided); 8 studies; 3677 participants); transformed; estimated linear regression coefficient range -140 to 66.1g	No association between prenatal PFOS exposure and birthweight via maternal blood samples collected in the first to second trimester using transformed data (b -4.0, 95%CI: -62.3 to 54.3; 4 studies, participants unspecified). However, PFOS exposure was associated with	

Study details	Appraisal details	Participants	Plastic exposure	Health outcomes	Findings	Subgroup Findings	AMSTAR score
			Unspecified, but detection limited to maternal or umbilical cord serum, plasma or whole blood or maternal milk<br><br>**Measure**<br>Authors have presented both natural units as mean PFAA (ng/mL) and then the natural log<br><br>**Exposure time**<br>Unspecified<br>But exposure was prenatal		for an increase of 1 loge ng/mL PFOS	decreased birthweight in maternal blood samples collected in the third trimester (b -65.1, 95%CI: -127.0 to -3.2; 2 studies, participants unspecified) and umbilical cord samples collected (b -93.2, 95%CI: -149.0 to -37.8; 3 studies, participants unspecified)	
**Steenland et al., 2018 [51] - Serum Perfluorooctanoic Acid and Birthweight: An Updated Meta-analysis With Bias Analysis. No COIs declared**							
**Last search**<br>Dec-17<br><br>**Study types**<br>Unspecified<br><br>**Included studies in the review** = 24<br><br>**Included studies in the meta-analysis =** 24	No critical appraisal appears to have been conducted	No further characteristics given<br><br>N = 19,173	**Type**<br>PFOA<br><br>**Route**<br>unspecified<br><br>**Measure**<br>ng/ml of maternal or cord blood log- untransformed PFOA. For studies that gave results only for log-transformed PFOA (nine studies; 11 results), we approximated the results for an untransformed analysis by iteratively minimizing the squared deviation of a new linear	birthweight<br>Assumed birthweight mean of about 3500grams. Summary coefficient (95% CI)	Exposure to PFOA there was an association between a change of birthweight of -10.5 g (-16.7, -4.4) for every ng/ml of maternal or cord blood (24studies, no participant data noted); approximately a drop of 0.3% in weight per unit of serum PFOA, assuming a mean birthweight of about 3,500 g	No association between PFOA and when blood sampling was early (First trimester a mixture of first and second,<br>or mostly/all preconception) was found s -3.3 [-9.6, 3.0, 7 studies, 5,393 births) compared with studies in which the blood sampling was late<br>(either second or third trimester, or a mixture of second/third trimester) and measurement of PFOA in blood sampling. -17.8 [-25.0, -10.6, 17 studies, 7,563 births).<br><br>sub-analysis of maternal blood studies to cord blood studies,	4

Study details	Appraisal details	Participants	Plastic exposure	Health outcomes	Findings	Subgroup Findings	AMSTAR score
			curve from the original logarithmic one, over a scale of 0 to 10 ng/ml PFOA,<br><br>**Exposure time**<br>In-utero but unspecified about how mothers were exposed.			found exposure to PFOA to be associated with blood studies, a change in birthweight -9.2 (-15.6, -2.8) g per ng/ml serum PFOA, 15 studies,<br>no participant data). Cord blood studies found an association between change in birthweight of -13.3 (-24.7, -1.8) g per ng/ml serum PFOA, 9 studies	
**Nieminen et al., 2013 [49] - Polychlorinated biphenyls (PCBs) in relation to secondary sex ratio –A systematic review of published studies. No COI declared**							
**Last search**<br>end of 2011 (no specific on date)<br><br>**Study types**<br>No discussion on study type or design<br><br>**Included studies in the review** = 15<br><br>**Included studies in the meta-**	Methodology quality was assessed by the authors. They did not use a formalised validated scale	No sample size was presented of the individual studies. The review included articles from pregnant women (Indigenous people of the Russian Artic; Lake Michigan mothers who had eaten Lake Michigan fish); Mothers who came to the hospital for delivery; female anglers; individuals who purchased from contaminated	**Type**<br>Polychlorinated biphenyls (PCBs)<br><br>**Route**<br>Direct or indirect exposure of PCBs through maternal or paternal<br><br>**Measure**<br>Blood serum: ug/L or ng/g; Cord serum: ng/mL; Breast milk: mg/kg High exposure group was taken as defined in the original papers: as the upper half (values above median), the highest quartile, quintile or 10th percentile group of the measured PCB distribution. A total sum of PCBs or a sum of more than six	secondary sex ratio (proportion of males) (Maternal exposure)<br>The main outcome measure was the proportion of boys among the newborns<br>Effect size with 95% CI	Direct high maternal exposure to PCBs and secondary sex ratio: 0.5(95%ci: 0.45,0.551, 8 studies)<br><br>Indirect high maternal exposure to PCBs did not reveal any extremely Low proportion of boys: 0.503 (95% CI: 0.487-0.519, 6 studies)<br><br>Internal comparisons between high and Low exposures groups: Please note that these are narrative figures alone- unable to identify these in a table which reports differently to these. Unable to also verify the studies included in the narrative analysis.<br><br>Difference in proportion of boys between direct high and Low	No subgroup analysis	3

Study details	Appraisal details	Participants	Plastic exposure	Health outcomes	Findings	Subgroup Findings	AMSTAR score
**analysis =** 15		farms, fish-eaters, residents of xop codes that contain PCB waste sites families in polluted JingHai country , individuals who ingested contaminated waste, women who had worked in three electrical capacitor plants.	congeners measured in the studies was used as an exposure measure. Indirect assessment of PCB exposure includes studies were PCB levels were not measured directly from each participant but other information existed showing that the parents were exposed to PCBs.<br><br>**Exposure time**<br>unspecified		maternal exposure was -0.048 (95%CI: 0.121,0.026)<br>Difference in proportion of boys between indirect high and Low maternal exposure was -0.033 (95%CI:-0.017,0.011)<br><br>Data extracted from table (4)<br><br>No association between the Difference of proportions of newborn males between groups with high and Low indirect maternal exposure of PCBs: -0.003 (95% CI: -0.017;0.011, 4 studies)<br>Exposure to Difference of proportions of newborn males between groups with high and Low direct maternal exposure is associated with PCBs: -0.069 (95% CI: 0.174;0.022, 9 studies)		

2.2 Child reproductive outcomes

Study details	Appraisal details	Participants	Plastic exposure	Health outcomes	Findings	Subgroup Findings	AMSTAR score
**Dorman et al., 2018 [58] - Systematic reviews and meta-analyses of human and animal evidence of prenatal diethylhexyl phthalate exposure and changes in male anogenital distance. No COI declared**							
**Last search** Oct-14	OHAT RoB tool (NTP 2015). Elements were rated as definitely	male infants: No further information about included	**Type**<br>diethylhexyl phthalate (DEHP)	anogenital distance (AGD). beta coefficient w 95% CI	Exposure to sum of DEHP (preferred over MEHP and DEHP metabolites) was associated with a change in AGD of −4.07 (95% CI:		8

Study details	Appraisal details	Participants	Plastic exposure	Health outcomes	Findings	Subgroup Findings	AMSTAR score
**Study types** observational prospective cohort studies<br><br>**Included studies in the review =** 6; however only 5 included in meta-analysis as only 1 study used AGD index<br><br>**Included studies in the meta-analysis =**5	low RoB, probably low RoB, probably high RoB, definitely high RoB, or not reported (NR). A rating of NR was considered equivalent to probably high-risk RoB. Most studies scores probably low risk to definitely low risk of bias in their evaluation. Bar Suzuki et al 2012 which was reported to have a high risk of bias as the reviewers could not be confident of their outcome assessments.	participants were reported<br><br>N = unspecified	**Route**<br>Unspecified<br><br>**Measure**<br>maternal urine ng/ml<br><br>**Exposure time**<br>In-utero between 1st and 3rd trimester		−6.49, −1.66; 5 studies, no participant data noted) % change per log10 change in urinary DEHP metabolite concentrations. Preferentially used AS measures to represent AGD over AP.		
**Nelson et al., 2020 [59] - In utero exposure to persistent and nonpersistent endocrine-disrupting chemicals and anogenital distance. A systematic review of epidemiological studies. No COI declared.**							
**Last search** Sep-19<br><br>**Study types** prospective cohort (n=13), cross	Newcastle–Ottawa Scale (NOS) with 15 items; good-quality studies (13–15 points); fair quality (10-12 points) and poor	Pregnant women and children (newborn up to 12 months)<br><br>N = NR	**Type**<br>BPA<br><br>**Route**<br>maternal (prenatal)<br><br>**Measure**	ano genital distance (AGD) specifically AGDAC (ano genital distance anal-clitoral distance) and AGDAF (ano genital distance anal-fourchette distance) in female children at birth	Significant summary estimate for the change in AGDAC (b=−1.374, 95% CI: −2.475 to −0.274; P = 0.014) % change per log10 change in maternal urinary first trimester BPA concentrations (3 studies, 1760 participants)	No subgroup analysis	7

Study details	Appraisal details	Participants	Plastic exposure	Health outcomes	Findings	Subgroup Findings	AMSTAR score
sectional (n=2), retrospective (n=1)<br><br>**Included studies in the review =** 16<br><br>**Included studies in the meta-analysis =** 3	quality (less than 9). Eleven articles of good quality, three of fair quality, and two poor quality.		Urinary BPA (µg/L) in first trimester; median concentration range 0.82-0.99µg/L in two studies and 1.26 µg/g Cr (adjusted for creatinine) in one study<br><br>**Exposure time**<br>NR	beta coefficients standardized to a percent change per log10 change; standardization done by dividing each reported beta coefficient by the mean value of the AGD	Nonsignificant estimate for the change in AGDAF (b=−1.069, 95% CI: −3.648 to −1.511; p = 0.417) % change per log10 change in maternal urinary BPA concentrations (3 studies, 1760 participants)	No subgroup analysis	
**Golestanzadeh et al., 2020 [56] - Association of phthalate exposure with precocious and delayed pubertal timing in girls and boys: a systematic review and meta-analysis. No COIs declared**							
**Last search**<br>Jul-19<br><br>**Study types**<br>Case control (n=13)<br>Cohort (n-17)<br>Cross sectional (n=9)<br><br>**Included studies in the review =** 39<br><br>**Included studies in**	The STROBE checklist was used to evaluate the methodology quality of the included papers. Discussion of appraisal was limited to "most of the included papers entered in this systematic review were high-quality papers based on the STROBE checklist".	Adolescent boys and girls, age ranging from 7 years (min) to 19 years (max).<br><br>N = 10,524	**Type**<br>Monoethyl phthalate (MEP)<br><br>**Route**<br>Unspecified. But exposure measured through blood or urine analysis<br><br>**Measure**<br>Unspecified<br><br>**Exposure time**<br>Unspecified	Abnormal breast development age<br>(Early breast development defined as before the age of 8; delayed breast development defined as after the age of 13)<br>OR and 95%CI<br>"If the regression coefficient remains in the interval +0.5 then correlation and regression coefficients are highly correlated (r=0.84) in a linear way. Therefore, extracted beta coefficients were transformed into correlation and then z Fisher transformation of correlation with the corresponding standard error was considered as desired effect size."	No association between MEP exposure and abnormal breast development age (OR=0.82, 95%CI: 0.6, 1.05; 3 studies, 609 participants).	No subgroup analysis	6

Study details	Appraisal details	Participants	Plastic exposure	Health outcomes	Findings	Subgroup Findings	AMSTAR score
**the meta-analysis**<br>= 4			**Type**<br>Monoethyl phthalate (MEP)<br><br>**Route**<br>Unspecified. But exposure measured through blood or urine analysis<br><br>**Measure**<br>Unspecified<br><br>**Exposure time**<br>Unspecified	Abnormal age of pubic-hair development (girls)<br>(Early development before the age of 8; delayed development defined as after the age of 13)<br>OR and 95%CI<br>"If the regression coefficient remains in the interval +0.5 then correlation and regression coefficients are highly correlated (r=0.84) in a linear way. Therefore, extracted beta coefficients were transformed into correlation and then z Fisher transformation of correlation with the corresponding standard error was considered as desired effect size	No association between MEP exposure and abnormal pubic-hair development age (OR =0.99, 95%CI: 0.81, 1.17; 3 studies, 609 participants).	No subgroup analysis	
			**Type**<br>Monoethyl phthalate (MEP)<br><br>**Route**<br>Unspecified. But exposure measured through blood or urine analysis<br><br>**Measure**<br>Unspecified<br><br>**Exposure time**<br>Unspecified	Abnormal age of menarche<br>(Early development before the age of 8; delayed development defined as after the age of 13)<br>OR and 95%CI<br>"If the regression coefficient remains in the interval +0.5 then correlation and regression coefficients are highly correlated (r=0.84) in a linear way. Therefore, extracted beta coefficients were transformed into correlation and then z Fisher transformation of correlation with the corresponding standard	No association between MEP exposure and an abnormal age of menarche (OR=0.89, 95%CI: 0.62, 1.16, 3 studies, 609 participants).	No subgroup analysis	

Study details	Appraisal details	Participants	Plastic exposure	Health outcomes	Findings	Subgroup Findings	AMSTAR score
				error was considered as desired effect size			
			**Type**<br>Monoethyl phthalate (MEP)<br><br>**Route**<br>Unspecified. But exposure measured through blood or urine analysis<br><br>**Measure**<br>Unspecified<br><br>**Exposure time**<br>Unspecified	Abnormal age of pubic-hair development (boys)<br>(Early development before the age of 8; delayed development defined as after the age of 13)<br>OR and 95%CI<br>"If the regression coefficient remains in the interval +0.5 then correlation and regression coefficients are highly correlated (r=0.84) in a linear way. Therefore, extracted beta coefficients were transformed into correlation and then z Fisher transformation of correlation with the corresponding standard error was considered as desired effect size	No association between MEP exposure and abnormal pubic-hair development age (OR=1.02, 95%CI: 0.85, 1.19; 3 studies, 609 participants).	N No subgroup analysis	
			**Type**<br>Monoethyl phthalate (MEP)<br><br>**Route**<br>Unspecified. But exposure measured through blood or urine analysis<br><br>**Measure**<br>Unspecified<br><br>**Exposure time**<br>Unspecified	Testicle volume (authors have not described how this variable has been categorised)<br>OR and 95%CI<br>"If the regression coefficient remains in the interval +0.5 then correlation and regression coefficients are highly correlated (r=0.84) in a linear way. Therefore, extracted beta coefficients were transformed into correlation and then z Fisher transformation of correlation with the corresponding standard	No association between MEP exposure and testicle volume (OR = 0.99, 95%CI: 0.77, 1.21; 2 studies, 387 participants).	No subgroup analysis	

Study details	Appraisal details	Participants	Plastic exposure	Health outcomes	Findings	Subgroup Findings	AMSTAR score
				error was considered as desired effect size			
			**Type**<br>Mono-methyl phthalate (MMP)<br><br>**Route**<br>Unspecified. But exposure measured through blood or urine analysis<br><br>**Measure**<br>Unspecified<br><br>**Exposure time**<br>Unspecified	Abnormal breast development age<br>(Early breast development defined as before the age of 8; delayed breast development defined as after the age of 13)<br>OR and 95%CI<br>"If the regression coefficient remains in the interval +0.5 then correlation and regression coefficients are highly correlated (r=0.84) in a linear way. Therefore, extracted beta coefficients were transformed into correlation and then z Fisher transformation of correlation with the corresponding standard error was considered as desired effect size	No association between MMP exposure and abnormal breast development age (OR = 0.84, 95%CI: 0.67, 1.01; 3 studies, 609 participants).	No subgroup analysis	
			**Type**<br>Mono-methyl phthalate (MMP)<br><br>**Route**<br>Unspecified. But exposure measured through blood or urine analysis<br><br>**Measure**<br>Unspecified<br><br>**Exposure time**	Abnormal age of pubic-hair development (girls)<br>(Early development before the age of 8; delayed development defined as after the age of 13)<br>OR and 95%CI<br>"If the regression coefficient remains in the interval +0.5 then correlation and regression coefficients are highly correlated (r=0.84) in a linear way. Therefore, extracted beta coefficients were transformed	No association between MMP exposure and abnormal pubic-hair development age (OR = 0.95, 95% CI: 0.77, 1.14; 3 studies, 609 participants).	No subgroup analysis	

Study details	Appraisal details	Participants	Plastic exposure	Health outcomes	Findings	Subgroup Findings	AMSTAR score
			Unspecified	into correlation and then z Fisher transformation of correlation with the corresponding standard error was considered as desired effect size			
			**Type**<br>Mono-methyl phthalate (MMP)<br><br>**Route**<br>Unspecified. But exposure measured through blood or urine analysis<br><br>**Measure**<br>Unspecified<br><br>**Exposure time**<br>Unspecified	Abnormal age of menarche<br>(Early development before the age of 8; delayed development defined as after the age of 13)<br>OR and 95%CI<br>"If the regression coefficient remains in the interval +0.5 then correlation and regression coefficients are highly correlated (r=0.84) in a linear way. Therefore, extracted beta coefficients were transformed into correlation and then z Fisher transformation of correlation with the corresponding standard error was considered as desired effect size	No association between MMP exposure and an abnormal age of menarche (OR= 0.89, 95%CI: 0.68, 1.10; 3 studies, 609 participants).	No subgroup analysis	
			**Type**<br>Mono-methyl phthalate (MMP)<br><br>**Route**<br>Unspecified. But exposure measured through blood or urine analysis<br><br>**Measure**<br>Unspecified	Abnormal age of pubic-hair development (boys)<br>(Early development before the age of 8; delayed development defined as after the age of 13)<br>OR and 95%CI<br>"If the regression coefficient remains in the interval +0.5 then correlation and regression coefficients are highly correlated (r=0.84) in a linear way. Therefore, extracted beta	No association between MMP exposure and abnormal pubic-hair development age (OR = 0.63, 95% CI: 0.23, 1.03; 4 studies, 727 participants).	No subgroup analysis	

Study details	Appraisal details	Participants	Plastic exposure	Health outcomes	Findings	Subgroup Findings	AMSTAR score
			**Exposure time**<br>Unspecified	coefficients were transformed into correlation and then z Fisher transformation of correlation with the corresponding standard error was considered as desired effect size			
			**Type**<br>Mono-methyl phthalate (MMP)<br><br>**Route**<br>Unspecified. But exposure measured through blood or urine analysis<br><br>**Measure**<br>Unspecified<br><br>**Exposure time**<br>Unspecified	Testicle volume (authors have not described how this variable has been categorised)<br>OR and 95%CI<br>"If the regression coefficient remains in the interval +0.5 then correlation and regression coefficients are highly correlated (r=0.84) in a linear way. Therefore, extracted beta coefficients were transformed into correlation and then z Fisher transformation of correlation with the corresponding standard error was considered as desired effect size	No association between MMP exposure and testicle volume (OR=1.01, 95%CI: 0.59, 1.44; 3 studies, 505 participants).	No subgroup analysis	
			**Type**<br>MnBP (Abbreviation not explained)<br><br>**Route**<br>Unspecified. But exposure measured through blood or urine analysis<br><br>**Measure**	Abnormal breast development age<br>(Early breast development defined as before the age of 8; delayed breast development after the age of 13)<br>OR and 95%CI<br>"If the regression coefficient remains in the interval +0.5 then correlation and regression coefficients are highly correlated (r=0.84) in a linear way.	No association between MnBP exposure and abnormal breast development age (OR = 1.03, 95%CI: 0.27, 1.79; 2 studies, 423 participants).	No subgroup analysis	

Study details	Appraisal details	Participants	Plastic exposure	Health outcomes	Findings	Subgroup Findings	AMSTAR score
			Unspecified<br><br>**Exposure time**<br>Unspecified	Therefore, extracted beta coefficients were transformed into correlation and then z Fisher transformation of correlation with the corresponding standard error was considered as desired effect size			
			**Type**<br>MnBP (Abbreviation not explained)<br><br>**Route**<br>Unspecified. But exposure measured through blood or urine analysis<br><br>**Measure**<br>Unspecified<br><br>**Exposure time**<br>Unspecified	Abnormal age of pubic-hair development (girls)<br>(Early development before the age of 8; delayed development after the age of 13)<br>OR and 95%CI<br>"If the regression coefficient remains in the interval +0.5 then correlation and regression coefficients are highly correlated (r=0.84) in a linear way. Therefore, extracted beta coefficients were transformed into correlation and then z Fisher transformation of correlation with the corresponding standard error was considered as desired effect size	No association between MnBP exposure and anormal age of pubic-hair development (OR =0.88, 95% CI: 0.59, 1.16, 2 studies, 423 participants).	No subgroup analysis	
			**Type**<br>MnBP (Abbreviation not explained)<br><br>**Route**<br>Unspecified. But exposure measured through blood or urine analysis	Abnormal age of menarche<br>(Early development before the age of 8; delayed development after the age of 13)<br>OR and 95%CI<br>"If the regression coefficient remains in the interval +0.5 then correlation and regression coefficients are highly correlated	No association between MnBP exposure and an abnormal age of menarche (OR=1.01, 95%CI: 0.06, 1.96; 2 studies, 423 participants).	No subgroup analysis	

Study details	Appraisal details	Participants	Plastic exposure	Health outcomes	Findings	Subgroup Findings	AMSTAR score
			**Measure**<br>Unspecified<br><br>**Exposure time**<br>Unspecified	(r=0.84) in a linear way. Therefore, extracted beta coefficients were transformed into correlation and then z Fisher transformation of correlation with the corresponding standard error was considered as desired effect size			
			**Type**<br>MnBP (Abbreviation not explained)<br><br>**Route**<br>Unspecified. But exposure measured through blood or urine analysis<br><br>**Measure**<br>Unspecified<br><br>**Exposure time**<br>Unspecified	Abnormal age of pubic-hair development (boys)<br>(Early development before the age of 8; delayed development defined as after the age of 13)<br>OR and 95%CI<br>"If the regression coefficient remains in the interval +0.5 then correlation and regression coefficients are highly correlated (r=0.84) in a linear way. Therefore, extracted beta coefficients were transformed into correlation and then z Fisher transformation of correlation with the corresponding standard error was considered as desired effect size	MnBP exposure was associated with a decreased odds of abnormal pubic-hair development age (OR=0.66, 95%CI: 0.39, 0.93; 2 studies, 423 participants).	No subgroup analysis	
			**Type**<br>Mono-ethylhexyl phthalate (MEHP)<br><br>**Route**<br>Unspecified. But exposure measured through blood or urine analysis	Abnormal breast development age<br>(Early breast development defined as before the age of 8; delayed breast development after the age of 13)<br>OR and 95%CI<br>"If the regression coefficient	No association between MEHP exposure and abnormal breast development (OR = 1.16, 95%CI: 0.73, 1.59; 3 studies, 609 participants).	No subgroup analysis	

Study details	Appraisal details	Participants	Plastic exposure	Health outcomes	Findings	Subgroup Findings	AMSTAR score
			**Measure**<br>Unspecified<br><br>**Exposure time**<br>Unspecified	remains in the interval +0.5 then correlation and regression coefficients are highly correlated (r=0.84) in a linear way. Therefore, extracted beta coefficients were transformed into correlation and then z Fisher transformation of correlation with the corresponding standard error was considered as desired effect size			
			**Type**<br>Mono-ethylhexyl phthalate (MEHP)<br><br>**Route**<br>Unspecified. But exposure measured through blood or urine analysis<br><br>**Measure**<br>Unspecified<br><br>**Exposure time**<br>Unspecified	Abnormal age of pubic-hair development (girls)<br>(Early development before the age of 8; delayed development defined as after the age of 13)<br>OR and 95%CI<br>"If the regression coefficient remains in the interval +0.5 then correlation and regression coefficients are highly correlated (r=0.84) in a linear way. Therefore, extracted beta coefficients were transformed into correlation and then z Fisher transformation of correlation with the corresponding standard error was considered as desired effect size	No association between MEHP exposure and abnormal pubic-hair development age (OR =0.91, 95CI: 0.74, 1.08, 3 studies, 609 participants).	No subgroup analysis	
			**Type**<br>Mono-ethylhexyl phthalate (MEHP)<br><br>**Route**	Abnormal age of menarche<br>(Early development before the age of 8; delayed development after the age of 13)	No association between MEHP exposure and an abnormal age of menarche (OR=0.89, 95%CI: 0.66, 1.11; 3 studies, 609 participants).	No subgroup analysis	

Study details	Appraisal details	Participants	Plastic exposure	Health outcomes	Findings	Subgroup Findings	AMSTAR score
			Unspecified. But exposure measured through blood or urine analysis<br><br>**Measure**<br>Unspecified<br><br>**Exposure time**<br>Unspecified	OR and 95%CI<br>"If the regression coefficient remains in the interval +0.5 then correlation and regression coefficients are highly correlated (r=0.84) in a linear way. Therefore, extracted beta coefficients were transformed into correlation and then z Fisher transformation of correlation with the corresponding standard error was considered as desired effect size			
			**Type**<br>Mono-ethylhexyl phthalate (MEHP)<br><br>**Route**<br>Unspecified. But exposure measured through blood or urine analysis<br><br>**Measure**<br>Unspecified<br><br>**Exposure time**<br>Unspecified	Abnormal age of pubic-hair development (boys)<br>(Early development before the age of 8; delayed development defined as after the age of 13)<br>OR and 95%CI<br>"If the regression coefficient remains in the interval +0.5 then correlation and regression coefficients are highly correlated (r=0.84) in a linear way. Therefore, extracted beta coefficients were transformed into correlation and then z Fisher transformation of correlation with the corresponding standard error was considered as desired effect size	No association between MEHP exposure and abnormal pubic-hair development age (OR=0.89, 95%CI: 0.62, 1.16; 3 studies, 609 participants).	No subgroup analysis	
			**Type**<br>Mono-ethylhexyl phthalate (MEHP)	Testicle volume (authors have not described how this variable has been categorised)	No association between MEHP exposure and testicle volume	No subgroup analysis	

Study details	Appraisal details	Participants	Plastic exposure	Health outcomes	Findings	Subgroup Findings	AMSTAR score
			**Route**<br>Unspecified. But exposure measured through blood or urine analysis<br><br>**Measure**<br>Unspecified<br><br>**Exposure time**<br>Unspecified	OR and 95%CI<br>"If the regression coefficient remains in the interval +0.5 then correlation and regression coefficients are highly correlated (r=0.84) in a linear way. Therefore, extracted beta coefficients were transformed into correlation and then z Fisher transformation of correlation with the corresponding standard error was considered as desired effect size	(OR=1.13, 95%CI: 0.88, 1.37; 2 studies, 387 participants).		
			**Type**<br>Mono(2-ethyl-5-hydroxyhexyl) phthalate (MEHHP)<br><br>**Route**<br>Unspecified. But exposure measured through blood or urine analysis<br><br>**Measure**<br>Unspecified<br><br>**Exposure time**<br>Unspecified	Abnormal breast development age<br>(Early breast development defined as before the age of 8; delayed breast development defined as after the age of 13)<br>OR and 95%CI<br>"If the regression coefficient remains in the interval +0.5 then correlation and regression coefficients are highly correlated (r=0.84) in a linear way. Therefore, extracted beta coefficients were transformed into correlation and then z Fisher transformation of correlation with the corresponding standard error was considered as desired effect size	Exposure to MEHHP was associated with an increased odds of abnormal breast development age (OR = 1.48, 95%CI: 1.11, 1.85; 2 studies, 387 participants).	No subgroup analysis	
			**Type**<br>Mono(2-ethyl-5-	Abnormal age of pubic-hair development (girls)	No association between MEHHP and abnormal pubic-hair	No subgroup analysis	

Study details	Appraisal details	Participants	Plastic exposure	Health outcomes	Findings	Subgroup Findings	AMSTAR score
			hydroxyhexyl) phthalate (MEHHP)<br><br>**Route**<br>Unspecified. But exposure measured through blood or urine analysis<br><br>**Measure**<br>Unspecified<br><br>**Exposure time**<br>Unspecified	(Early development before the age of 8; delayed development defined as after the age of 13)<br>OR and 95%CI<br>"If the regression coefficient remains in the interval +0.5 then correlation and regression coefficients are highly correlated (r=0.84) in a linear way. Therefore, extracted beta coefficients were transformed into correlation and then z Fisher transformation of correlation with the corresponding standard error was considered as desired effect size	development age (OR = 0.96, 95% CI: 0.59, 1.13; 2 studies, 387 participants).		
			**Type**<br>Mono(2-ethyl-5-hydroxyhexyl) phthalate (MEHHP)<br><br>**Route**<br>Unspecified. But exposure measured through blood or urine analysis<br><br>**Measure**<br>Unspecified<br><br>**Exposure time**<br>Unspecified	Abnormal age of menarche (Early development before the age of 8; delayed development after the age of 13)<br>OR and 95%CI<br>"If the regression coefficient remains in the interval +0.5 then correlation and regression coefficients are highly correlated (r=0.84) in a linear way. Therefore, extracted beta coefficients were transformed into correlation and then z Fisher transformation of correlation with the corresponding standard error was considered as desired effect size	No association between MEHHP exposure and an abnormal age of menarche (OR=1.07, 95%CI: 0.14, 2.01; 2 studies, 387 participants).	No subgroup analysis	

Study details	Appraisal details	Participants	Plastic exposure	Health outcomes	Findings	Subgroup Findings	AMSTAR score
			**Type**<br>Mono(2-ethyl-5-hydroxyhexyl) phthalate (MEHHP)<br><br>**Route**<br>Unspecified. But exposure measured through blood or urine analysis<br><br>**Measure**<br>Unspecified<br><br>**Exposure time**<br>Unspecified	Abnormal age of pubic-hair development (boys)<br>(Early development before the age of 8; delayed development defined as after the age of 13)<br>OR and 95%CI<br>"If the regression coefficient remains in the interval +0.5 then correlation and regression coefficients are highly correlated (r=0.84) in a linear way. Therefore, extracted beta coefficients were transformed into correlation and then z Fisher transformation of correlation with the corresponding standard error was considered as desired effect size	Exposure to MEHHP was associated with a decreased odd of an abnormal pubic-hair development age (OR = 0.61, 95%CI: 0.32, 0.91; 2 studies, 387 participants).	No subgroup analysis	
			**Type**<br>Mono(2-ethyl-5-hydroxyhexyl) phthalate (MEHHP)<br><br>**Route**<br>Unspecified. But exposure measured through blood or urine analysis<br><br>**Measure**<br>Unspecified<br><br>**Exposure time**<br>Unspecified	Testicle volume (authors have not described how this variable has been categorised)<br>OR and 95%CI<br>"If the regression coefficient remains in the interval +0.5 then correlation and regression coefficients are highly correlated (r=0.84) in a linear way. Therefore, extracted beta coefficients were transformed into correlation and then z Fisher transformation of correlation with the corresponding standard error was considered as desired effect size	No association between MEHHP exposure and testicle volume (OR=0.79, 95%CI: 0.44, 1.14; 2 studies, 387 participants).	No subgroup analysis.	

Study details	Appraisal details	Participants	Plastic exposure	Health outcomes	Findings	Subgroup Findings	AMSTAR score
			**Type**<br>Mono(2-ethyl-5-oxohexyl) phthalate<br>(MEOHP)<br><br>**Route**<br>Unspecified. But exposure measured through blood or urine analysis<br><br>**Measure**<br>Unspecified<br><br>**Exposure time**<br>Unspecified	Abnormal breast development age<br>(Early breast development defined as before the age of 8; delayed breast development defined as after the age of 13)<br>OR and 95%CI<br>"If the regression coefficient remains in the interval +0.5 then correlation and regression coefficients are highly correlated (r=0.84) in a linear way. Therefore, extracted beta coefficients were transformed into correlation and then z Fisher transformation of correlation with the corresponding standard error was considered as desired effect size	Exposure to MEOHP was associated with an increased odds of abnormal breast development age (OR = 1.52, 95%CI: 1.15, 1.88; 2 studies, 387 participants).	No subgroup analysis	
			**Type**<br>Mono(2-ethyl-5-oxohexyl) phthalate<br>(MEOHP)<br><br>**Route**<br>Unspecified. But exposure measured through blood or urine analysis<br><br>**Measure**<br>Unspecified<br><br>**Exposure time**<br>Unspecified	Abnormal age of pubic-hair development (girls)<br>(Early development before the age of 8; delayed development defined as after the age of 13)<br>OR and 95%CI<br>"If the regression coefficient remains in the interval +0.5 then correlation and regression coefficients are highly correlated (r=0.84) in a linear way. Therefore, extracted beta coefficients were transformed into correlation and then z Fisher transformation of correlation	No association between MEOHP and abnormal pubic-hair development age (OR = 0.95 95% CI: 0.66, 1.23; 2 studies, 387 participants).	No subgroup analysis	

Study details	Appraisal details	Participants	Plastic exposure	Health outcomes	Findings	Subgroup Findings	AMSTAR score
				with the corresponding standard error was considered as desired effect size			
			**Type**<br>Mono(2-ethyl-5-oxohexyl) phthalate<br>(MEOHP)<br><br>**Route**<br>Unspecified. But exposure measured through blood or urine analysis<br><br>**Measure**<br>Unspecified<br><br>**Exposure time**<br>Unspecified	Abnormal age of menarche<br>(Early development before the age of 8; delayed development after the age of 13)<br>OR and 95%CI<br>"If the regression coefficient remains in the interval +0.5 then correlation and regression coefficients are highly correlated (r=0.84) in a linear way. Therefore, extracted beta coefficients were transformed into correlation and then z Fisher transformation of correlation with the corresponding standard error was considered as desired effect size	No association between MEOHP exposure and an abnormal age of menarche (OR=1.08 95% CI:0.19, 1.98, 2 studies, 387 participants).	No subgroup analysis	
			**Type**<br>Mono(2-ethyl-5-oxohexyl) phthalate<br>(MEOHP)<br><br>**Route**<br>Unspecified. But exposure measured through blood or urine analysis<br><br>**Measure**<br>Unspecified<br><br>**Exposure time**	Abnormal age of pubic-hair development (boys)<br>(Early development before the age of 8; delayed development defined as after the age of 13)<br>OR and 95%CI<br>"If the regression coefficient remains in the interval +0.5 then correlation and regression coefficients are highly correlated (r=0.84) in a linear way. Therefore, extracted beta coefficients were transformed into correlation and then z Fisher	Exposure to MEOHP was associated with a decreased odd for an abnormal pubic-hair development age (OR = 0.61, 95%CI: 0.26, 0.97; 2 studies, 387 participants).	No subgroup analysis	

Study details	Appraisal details	Participants	Plastic exposure	Health outcomes	Findings	Subgroup Findings	AMSTAR score
			Unspecified	transformation of correlation with the corresponding standard error was considered as desired effect size			
			**Type**<br>Mono(2-ethyl-5-oxohexyl) phthalate (MEOHP)<br><br>**Route**<br>Unspecified. But exposure measured through blood or urine analysis<br><br>**Measure**<br>Unspecified<br><br>**Exposure time**<br>Unspecified	Testicle volume (authors have not described how this variable has been categorised)<br>OR and 95%CI<br>"If the regression coefficient remains in the interval +0.5 then correlation and regression coefficients are highly correlated (r=0.84) in a linear way. Therefore, extracted beta coefficients were transformed into correlation and then z Fisher transformation of correlation with the corresponding standard error was considered as desired effect size	No association between MEOHP exposure and testicle	No subgroup analysis	
**Bigambo et al., 2020 [55] - Association between phenols exposure and earlier puberty in children: A systematic review and meta-analysis. No COIs declared**							
**Last search**<br>Feb-20<br><br>**Study types**<br>Cohort (n=4); Case-control (n=3); Cross-sectional (n=2)<br><br>**Included studies in**	Newcastle Ottawa Scale (Supornsilchai et al., 2016) was used to assess the quality of the included cohort and case-control studies, and the adapted Newcastle Ottawa cohort Scale form was used to assess	Girls<br><br>N = 4,737	**Type**<br>Bisphenol A (BPA)<br><br>**Route**<br>Unspecified<br><br>**Measure**<br>Urine<br><br>**Exposure time**<br>Unspecified	risk of earlier puberty (precocious puberty, earlier puberty, idiopathic central precocious puberty, premature thelarche, earlier menarche, and earlier pubarche) odds ratio (OR) and Hazard ratio (HR) combined in meta-analysis to overall effect size (ES) - dichotomous variables	No association between BPA and risk of earlier puberty in girls (ES=1.09, 95%CI: 0.88, 1.35; 8 studies, 3498 participants)	No subgroup analysis	5

Study details	Appraisal details	Participants	Plastic exposure	Health outcomes	Findings	Subgroup Findings	AMSTAR score
**the review =** 9<br><br>**Included studies in the meta-analysis =** 9	the cross-sectional studies (Herzog et al., 2013): low quality with score 0–3, moderate quality with score 4–6, and high quality with score 7–9. Two studies moderate quality with a moderate risk of bias (cross-sectional) and seven of high quality with low risk of bias.						
**Wen et al., 2015 [57] - Association of PAEs with Precocious Puberty in Children: A Systematic Review and Meta-Analysis. No COIs declared**							
**Last search** Mar-15<br><br>**Study types** Case-control<br><br>**Included studies in the review =** 14<br><br>**Included studies in the meta-analysis =** 14	New-Castle Ottawa Scale. Among 14 included studies, except for seven studies were considered as moderate risk of bias scores of 5 and 6), the others were assessed as low risk of bias (scores of 7 and 8) (Table 2). All studies had adequate case and controls definition,	Female children aged 0.5-11.3 years acted as cases (precocious puberty) and compared against female children aged 2.2-12 years).<br><br>N = 2,012	**Type** di(2-ethylhexyl) phthalate (DEHP)<br><br>**Route** Unspecified<br><br>**Measure** Concentration in the serum and urine (combined for MA)<br><br>**Exposure time** Unspecified	Prevalence precocious puberty (PP) (the appearance of secondary sex characteristics before the age of eight years in girls).<br>OR and 95%CI<br>No logistic regression<br><br>Serum concentration (mean and standard deviation)<br>SMD and 95%CI	There was a positive association between serum DEHP and the odds of PP (OR=4.09, 95%CI:2.3, 7.3; 7 studies, 1390 participants).<br><br>Serum concentration of DEHP was significantly greater in the PP group compared to the control group (SMD=1.73, 95%: 0.54, 2.91; 7 studies, 1564 participants).	No subgroup analysis<br><br>No subgroup analysis	7
			**Type** dibutyl phthalate (DBP)	Prevalence precocious puberty (PP) (the appearance of secondary sex characteristics	There was no association between serum DBP concentration and the odds of PP	No subgroup analysis	

Study details	Appraisal details	Participants	Plastic exposure	Health outcomes	Findings	Subgroup Findings	AMSTAR score
	and exposure assessment. No standard method was adopted in sampling, which would affect the representativeness of the cases. The controls were recruited from the same community as cases in only five studies. Seven just matched case and control by age without consideration of other confounding factors.		**Route**<br>Unspecified<br><br>**Measure**<br>Concentration in the blood and urine (combined for MA)<br><br>**Exposure time**<br>Unspecified	before the age of eight years in girls).<br>OR and 95%CI<br>No logistic regression<br><br>Serum concentration (mean and standard deviation)<br>SMD and 95%CI	(OR=3.26, 95%CI:0.69, 15.42; 5 studies, 1149 participants).		
					Serum concentration of DBP was significantly greater in the PP group compared to the control group (SMD=4.31, 95%CI:2.67, 5.95; 5 studies, 1323 participants).	No subgroup analysis	
			**Type**<br>Mono-ethylhexyl phthalate (MEHP)<br><br>**Route**<br>Unspecified<br><br>**Measure**<br>Concentration in the blood and urine (combined for MA)<br><br>**Exposure time**<br>Unspecified	Prevalence precocious puberty (PP) (the appearance of secondary sex characteristics before the age of eight years in girls).<br><br>Serum concentration (mean and standard deviation)<br>SMD and 95%CI<br><br>Urinary concentration<br>SMD and 95%CI	There was no association in serum MEHP concentration between the PP and the control group (SMD=0.18, 95%CI: -0.99, 1.36; 4 studies, 895 participants).	No subgroup analysis	
					There was no association in urinary MEHP concentration between the PP and the control group (SMD= -0.44, 95%CI: -1.18, 0.29; 3 studies, participants unspecified); Chinese studies only	No subgroup analysis	
			**Type**<br>Mono-butyl phthalate (MBP)<br><br>**Route**<br>Unspecified<br><br>**Measure**<br>Concentration in the blood	Prevalence precocious puberty (PP) (the appearance of secondary sex characteristics before the age of eight years in girls).<br><br>Serum concentration (mean and standard deviation)	There was no association in serum MBP concentration the PP and the control group (SMD=0.01, 95%CI: -0.3, 0.27; 3 studies, 784 participants)	No subgroup analysis	
					There was no association in urinary MBP concentration between the PP and the control group (SMD= -0.11, 95%CI: -0.48,	No subgroup analysis	

Study details	Appraisal details	Participants	Plastic exposure	Health outcomes	Findings	Subgroup Findings	AMSTAR score
			and urine (combined for MA)<br><br>**Exposure time**<br>Unspecified	SMD and 95%CI<br>Urinary concentration<br>SMD and 95%CI	0.26; 3 studies, participants unspecified)		
			**Type**<br>MMP<br><br>**Route**<br>Unspecified<br><br>**Measure**<br>Concentration in the blood and urine (combined for MA)<br><br>**Exposure time**<br>Unspecified	Prevalence precocious puberty (PP) (the appearance of secondary sex characteristics before the age of eight years in girls).<br><br>Urinary concentration<br>SMD and 95%CI	There was no association in urinary MMP concentration between the PP and the control group (SMD= 0.27, 95%CI: -0.21, 0.76; 3 studies, participants unspecified); Chinese studies only	No subgroup analysis	
			**Type**<br>MBzP<br><br>**Route**<br>Unspecified<br><br>**Measure**<br>Concentration in the blood and urine (combined for MA)<br><br>**Exposure time**<br>Unspecified	Prevalence precocious puberty (PP) (the appearance of secondary sex characteristics before the age of eight years in girls).<br><br>Urinary concentration<br>SMD and 95%CI	There was no association in urinary MBzP concentration between the PP and the control group (SMD= 0.00, 95%CI: -0.43, 0.43; 4 studies, participants unspecified)	No subgroup analysis	
			**Type**<br>MEP	Prevalence precocious puberty (PP) (the appearance of secondary sex characteristics	There was no association in urinary MEP concentration between the PP and the control	No subgroup analysis	

Study details	Appraisal details	Participants	Plastic exposure	Health outcomes	Findings	Subgroup Findings	AMSTAR score
			**Route**<br>Unspecified<br>**Measure**<br>Concentration in the blood and urine (combined for MA)<br><br>**Exposure time**<br>Unspecified	before the age of eight years in girls).<br><br>Urinary concentration<br>SMD and 95%CI	group (SMD= 0.73, 95%CI: -0.40, 1.86]; 3 studies, participants unspecified)		

2.3 Adult reproductive outcomes

Study details	Appraisal details	Participants	Plastic exposure	Health outcomes	Findings	Subgroup Findings	AMSTAR score
**Wen et al., 2019 [64] - The risk of endometriosis after exposure to endocrine-disrupting chemicals: a meta-analysis of 30 epidemiology studies. No COIs declared**							
**Last search**<br>Jan-18<br><br>**Study types**<br>Case-control (n=21)<br>Cohort (n=8)<br>Cross-sectional (n=1)<br><br>**Included studies in the review** = 30<br><br>**Included studies in the meta-analysis** = 4	A modification of the Newcastle-Ottawa Scale (NOS) was used to assess quality of case-control studies and cohort studies for three aspects: the selection of study groups, comparability of groups, and ascertainment of either the exposure or outcome of interest. Cross-	Only participant details provided was if the study sample was sourced from the general population or from a hospitalised population.<br><br>N = 7,127	**Type**<br>BPA<br><br>**Route**<br>Unspecified<br><br>**Measure**<br>Concentration in urine (µg/L), serum (µg/g) or fat (ng/g fat) (combined for meta-analysis)<br><br>**Exposure time**<br>Unspecified	Endometriosis<br>OR and 95%CI	Overall analysis excluded due to combination of all included endocrine disruptors.<br><br>There was no association between BPA exposure (urinary) and odds of endometriosis (OR=1.4, 95%CI 0.94, 2.08; 4 studies, 1130 participants).	No subgroup analysis	7

	sectional study was assessed by using Agency for Healthcare Research and Quality (AHRQ) with 11-item checklist. Quality was assessed by two independent authors. Quality assessment was depicted in Supplemental Table S3, the quality of all included studies was acceptable with score range 5–8.						
**Cai et al., 2019 [60] - Association between Phthalate Metabolites and Risk of Endometriosis: A Meta-Analysis. No COIs declared**							
**Last Search**<br>Mar-19<br><br>**Study types**<br>study total n=8<br>cross-sectional n= 1<br>case-control	Newcastle–Ottawa Scale (NOS) was used the authors make no claims on how this could impact on interpretation of assessment. All studies scored between 6-7 (scores could range from 0-9)	Women aged between 18-54;<br><br>n = 2 studies did not state their age range.<br><br>Cases n = 620; control n = 1922; total = 2542	**Type**<br>mono-(2-ethyl-5-hydroxyhexyl) phthalate (MEHHP)<br><br>**Route**<br>Unspecified<br><br>**Measure**<br>Urine: Unspecified on measurement	Endometriosis<br>OR and (95% CI)	Exposure to MEHHP was significantly associated with the risk of endometriosis in women (OR = 1.246, 95% CI = 1.003–1.549, p=0.111)	No subgroup analysis	7

n= 5<br>cohort n= 7<br><br>**Included studies in the review =** 8<br><br>**Included studies in the meta-analysis** = 8			**Exposure time**<br>non-specific				
			**Type**<br>mono(2-ethylhexyl) phthalate (MEHP)<br><br>**Route**<br>Unspecified<br><br>**Measure**<br>Urine; Plasma<br><br>**Exposure time**<br>non-specific	Endometriosis<br>OR and (95% CI)	No association between MEHP and endometriosis risk in women (OR=1.089, 95% CI=.858,1.383, 7 studies, no participant data).	No subgroup analysis	
			**Type**<br>monoethyl phthalate (MEP)<br><br>**Route**<br>Unspecified<br><br>**Measure**<br>Urine: Unspecified on measurement<br><br>**Exposure time**<br>non-specific	Endometriosis<br>OR and (95% CI)	No association with MEP with endometriosis in women (OR=1.073;95% CI 0.899, 1.282, 6 studies, no participant data noted)	No subgroup analysis	
			**Type**<br>monobenzyl phthalate (MBzP)<br><br>**Route**<br>Unspecified<br><br>**Measure**<br>Urine: Unspecified on measurement	Endometriosis<br>OR and (95% CI)	MBzP was not associated with endometriosis (7 studies) (OR=0.976;95% CI 0.810, 1.176)	No subgroup analysis	

			**Exposure time**<br>non-specific				
			**Type**<br>mono(2-ethyl-5-oxohexyl) phthalate (MEOHP)<br><br>**Route**<br>Unspecified<br><br>**Measure**<br>Urine: Unspecified on measurement<br><br>**Exposure time**<br>non-specific	Endometriosis<br>OR and (95% CI)	MEOHP was not associated with endometriosis (6 studies) (OR=1.282;95% CI 0.874, 1.881)	No subgroup analysis	
**Cano-Sancho et al., 2019 [61] - Human epidemiological evidence about the associations between exposure to organochlorine chemicals and endometriosis: Systematic review and meta-analysis. No COI declared.**							
**Last search**<br>Aug-18<br><br>**Study types**<br>Case-control (n=16)<br>cohort (n=1)<br><br>**Included studies in the review =** 17<br><br>**Included studies in the meta-analysis** = 9	Adapted National Toxicology Program/ Office of Health Assessment and Translation (NTP/OHAT) Risk of Bias Rating Tool for Human and Animal Studies. Overall, most studies were classified as tier 1 or tier 2 indicating the presence plausible bias that may raise	Unclear study states all ages, body mass index, and/or life-stage at exposure or outcome (assume women)<br><br>N = 32,743	**Type**<br>Polychlorinated biphenyl (PCB)<br><br>**Route**<br>NR<br><br>**Measure**<br>Serum (units NR)<br><br>**Exposure time**<br>NR	Endometriosis risk<br>log OR and 95%Cis;<br>High versus Low percentiles	Exposure to PCB was associated with increased odds in developing endometriosis (log OR=0.53, 95%CI: 0.18, 0.57; 9 studies, 31041 participants) corresponding to an OR= 1.70, 95%CI: 1.20, 2.39). There was considerable heterogeneity (I2=78%)	Subgroup by sample revealed an associated risk in serum samples (OR 2.02, 95%CI; 1.20 to 3.40; 6 studies, 2,271 participants) but not in adipose tissues samples (OR 1.42, 95%CI: 0.91 to 2.21; 3 studies, 28,770 participants).<br><br>Subgroup by the type of outcome revealed an associated risk whether deep endometriosis (OR 1.76, 95%CI: 1.35 to 2.28, 2 studies, participants unspecified) or total endometriosis (OR 1.73, 95%CI: 1.08 to 2.76; 7 studies, participants unspecified).	8

	some doubt about the results.						
**Roy et al., 2015 [62] - Integrated Bioinformatics, Environmental Epidemiologic and Genomic Approaches to Identify Environmental and Molecular Links between Endometriosis and Breast Cancer. No COI declared**							
**Last search** Unspecified<br><br>**Study types** Case-control<br><br>**Included studies in the review** = 6		Women with endometriosis and matched healthy controls<br><br>N = 1380 (case=542; control=838)	**Type** Polychlorinated biphenyl (PCB)<br><br>**Route** NR<br><br>**Measure** Serum (ng/g)<br><br>**Exposure time** NR	Endometriosis<br>OR and 95%CI<br>No logistic regression	Exposure to PCB was associated with increased odds of developing endometriosis in women (OR= 1.91, 95%CI: 1.05, 5.54; 6 studies, 1380).	No subgroup analysis conducted	3
**Cai et al., 2015 [63] - Human urinary/seminal phthalates or their metabolite levels and semen quality: A meta-analysis. No COIs declared**							
**Last search** Oct-14<br><br>**Study types** mostly cross-sectional. Did not identify other study types.<br><br>**Included studies in the review** 20 studies met the inclusion criteria; 14 were further	Used Elwood (1998) Critical Appraisal of Epidemiological Studies and Clinical Trials. Studies with a score no lower than–2 were excluded (three studies were removed) in the qualitative and quantitative analysis of the associations between phthalates or their metabolite	Most (no N identified) studies measuring urinary phthalate metabolites used sub-fertile males who were part of infertility workups.<br>n= 3 studies were in healthy men of reproductive age from the general population	**Type** MBP<br><br>**Route** Environmental<br><br>**Measure** Urinary phthalates or metabolite levels; Seminal phthalates levels; Serum phthalate levels<br><br>**Exposure time** non-specific	Sperm Concentration<br>OR and (95% Cl); Beta co-efficient (95% CI)<br><br>NB:<br>Risk (OR) indicative of semen quality were compared to a reference value in men at or above a sperm concentration of ≥ 20×10^6 *m*L, motility of ≥ 50% motile, and morphology of ≥ 4% normal morphology.	Exposure to MBP levels of 7.4-25.3ug/L was +ve associated with reduced sperm concentrations in males of reproductive age 2.6 (95% OR: 1.32, 5.15, 3 Studies, no participant data).<br><br>Exposure to MBP levels of 26.0-14459.0ug/L was +ve associated with reduced sperm concentration in males of reproductive age 2.39 (95% OR:1.26, 4.53, 5 studies, no participant data).<br><br>No associations between MBP and low sperm concentrations using beta-coefficients in males of reproductive age (b=0.04,	No subgroup analysis	6

included in the meta-analysis. Total n of 20 studies = 4945<br><br>**Included studies in the meta-analysis =**<br>14	levels in humans and semen quality	Most (no N identified) studies measuring seminal phthalates chose men from the general population as their subjects. (no N identified) male subjects of reproductive age (i.e.,20–50 years),<br>n=2 studies males of younger age, i.e., 18–22 years<br><br>total participants in 20 studies = 4945			95%OR: -0.45,0.54, 3 studies, no participant data)		
				Sperm motility<br>OR and (95% CI)	No association between MBP levels of 7.4-25.3ug/L and low sperm motility in males of reproductive age 1.16 (95% OR:0.58, 2.34, 3 studies, no participant data).<br><br>No association between MBP levels of 26.0-14459.0ug/L and low sperm motility 1.35 in males of reproductive age (95% OR: 0.86, 2.11, 5 studies, no participant data).	No subgroup analysis	
				Sperm Morphology<br>OR and (95% CI)	No association between MPB levels of 7.4-25.3ug/L and low sperm morphology in males of reproductive age 1.00 (95% OR:0.59, 1.71, 2 studies, no participant data).<br>No association between MPB levels of 26.0-14459.0ug/L and sperm morphology in males of reproductive age 1.43 (95% OR: 0.83, 2.47, 4 studies, no participant data).	No subgroup analysis	
				sperm volume<br>OR and (95% CI)	No association between MPB levels of 26.0-14459.0ug/L and sperm volume in males of reproductive age .80 (95% OR: 0.26,2.40, 2 studies, no participant data)	No subgroup analysis	
				Sperm motion parameters:<br>Straight Line velocity (VSL)	No association between MBP levels of 10.3–24.6 ug/L and Straight-line velocity (VSL) in men	No subgroup analysis	

				Beta co-efficients and 95% Confidence intervals	of reproductive age (b= -1.48 95%CI: -3.87, 0.92,3 studies, no participant data noted)) Exposure to MBP levels 24.6–14,459.0 was associated with Straight line velocity (VSL) in men of reproductive age (b= -2.51, 95%CI: -4.44, -0.59, 3 studies, no participant data noted)		
				Sperm motion parameters: Curvilinear velocity (VCL) Beta co-efficients and 95% Confidence intervals	No association between MBP levels of 10.3–24.6 ug/L and Curvilinear velocity (VCL) in men of reproductive age (b= -2.60 95%CI: -5.40, 0.19, 3 studies, no participant data noted) Exposure to MBP levels 24.6–14,459.0 was associated with Curvilinear velocity (VCL) in men of reproductive age (b= -3.81 95%CI: -6.74, -0.87, 3 studies, no participant data noted)	No subgroup analysis	
				Sperm motion parameters: Linearity (LIN) Beta co-efficients and 95% Confidence intervals	No association between MBP levels of 10.3–24.6 ug/L and Linearity (LIN) in men of reproductive age (b= -0.14 95%CI: -2.64, 2.36, 3 studies, no participant data noted) No association between MBP levels of 24.6–14,459.0 and Linearity (LIN) in men of reproductive age (b= -0.70 95%CI: -2.46, 1.07, 3 studies, no participant data noted)	No subgroup analysis	
				Comet Assay Parameters: Comet extent	No association between MBP (IQR 20.75 UG/l) and Comet extent in men of reproductive age (b= -0.30	No subgroup analysis	

				Beta co-efficients and 95% Confidence intervals	95% CI: -0.79, 0.19, 2 studies, no participant data noted)		
				Comet Assay Parameters: Percent DNA in tails<br>Beta co-efficients and 95% Confidence intervals	No association between MBP (IQR 20.75 UG/l) and Percent DNA in tail in men of reproductive age (b=0.64 95% CI: -0.94, 2.23, 2 studies, no participant data noted)	No subgroup analysis	
				comet assay parameters: Tail distributed moment<br>Beta co-efficients and 95% Confidence intervals	No association between MBP (IQR 20.75 UG/l) and Tail distributed moment in men of reproductive age (b=-0.122 95% CI: -0.32, 0.08, 2 studies, no participant data noted)	No subgroup analysis	
			**Type**<br>MBzP<br><br>**Route**<br>Environmental<br><br>**Measure**<br>Urinary phthalates or metabolite levels; Seminal phthalates levels; Serum phthalate levels<br><br>**Exposure time**<br>non-specific	Sperm Concentration<br>OR and (95% CI)	No association between MBzP levels of 0-14.0ug/L and reduced sperm concentration in males of reproductive age1.24 (95% CI: 0.67, 2.29, 6 studies, no participant data)<br>Exposure to MBzP levels of 14-540.2 ugl/L was +ve associated with low sperm concentration 2.23 (95% OR: 1.16, 4.30, 3 studies, no participant data)	No subgroup analysis	
				Sperm motility<br>OR and (95% CI)	No association between MBzP levels of 0-14.0ug/L and low sperm motility in males of reproductive age 1.24 (95% CI: 0.78, 1.84, 6 studies or participant data provided).<br><br>No association between MBzP levels of 14-540.2 ugl/L and low sperm motility in males of reproductive age1.47 (95% OR:	No subgroup analysis	

					0.91, 2.36, 3 studies, no participant data)		
				Sperm Morphology OR and (95% CI)	No association between MBzP levels of 0-14.0ug/L and low sperm morphology in males of reproductive age0.70 (95% CI: 0.38–1.28, 6 studies or participant data recorded).<br><br>No association between MBzP levels of 14-540.2 ugl/L and low sperm morphology in males of reproductive age 1027 (95% OR: 0.77, 2.08, 3 studies, no participant data)	No subgroup analysis	
				Sperm motion parameters: Straight Line velocity (VSL) Beta co-efficients and 95% Confidence intervals	No association between MBzP levels of 4.2–64.2 and Straight-line velocity (VSL) in men of reproductive age (b=-0.42 95%CI: -1.39, 0.55, 3 studies, no participant data noted)<br>No association between MBzP levels of 64.2–540.2 and Straight-line velocity (VSL) in men of reproductive age (b=-1.93 95%CI: -3.98, 0.12, 3 studies, no participant data noted)	No subgroup analysis	
				Sperm motion parameters: Curvilinear velocity (VCL) Beta co-efficients and 95% Confidence intervals	No association between MBzP levels of 4.2–64.2 and Curvilinear velocity (VCL) in men of reproductive age (b= -0.44 95%CI: -1.94, 1.07, 3 studies, no participant data noted)<br>No association between MBzP levels of 64.2–540.2 and Curvilinear velocity (VCL) in men	No subgroup analysis	

					of reproductive age (b=-1.70 95%CI: -5.21, 1.82, 3 studies, no participant data noted)		
				Sperm motion parameters: Linearity (LIN)<br>Beta co-efficients and 95% Confidence intervals	No association between MBzP levels of 4.2–64.2 and Linearity (LIN) in men of reproductive age (b=-0.22 95%CI: -0.81, 0.38, 3 studies, no participant data noted)<br>No association between MBzP levels of 64.2–540.2 and Linearity (LIN) in men of reproductive age (b=-1.05 95%CI: -2.51, 0.40, 3 studies, no participant data noted)	No subgroup analysis	
				Comet Assay Parameters: Comet extent<br>Beta co-efficients and 95% Confidence intervals	Exposure to MBzP (IQR 11.35UG/l) was associated with Comet extent in men of reproductive age (b=3.57 95% CI:0.89, 6.25, 2 studies, no participant data noted)	No subgroup analysis	
				Comet Assay Parameters: Percent DNA in tails<br>Beta co-efficients and 95% Confidence intervals	No association between MBzP (IQR 11.35UG/l) and Percent DNA in tailin men of reproductive age (b=0.05 95% CI: -0.38, 0.48, 2 studies, no participant data noted)	No subgroup analysis	
				comet assay parameters: Tail distributed moment<br>Beta co-efficients and 95% Confidence intervals	Exposure to MBzP (IQR 11.35UG/l) was associated with Tail distributed moment in men of reproductive age (b=1.72 95% CI:0.33, 3.12, 2 studies, no participant data noted)	No subgroup analysis	
			**Type**	Sperm Concentration<br>OR and (95% CI)	No association between MMP levels of 1.5-9.0ug/L and low	No subgroup analysis	

			MMP<br><br>**Route**<br>Environmental<br><br>**Measure**<br>Urinary phthalates or metabolite levels; Seminal phthalates levels; Serum phthalate levels<br><br>**Exposure time**<br>non-specific		sperm concentration in males of reproductive age 0.89 (95% CI: 0.48–1.67, 6 studies or participant data noted)<br>No association between MMP levels of 9.0-745.0ug/L and low sperm concentration in males of reproductive age 0.96 (95% CI: 0.28–3.29, 6 studies or participant data noted)		
				Sperm motility<br>OR and (95% CI)	No association between MMP levels of 1.5-9.0ug/L and low sperm Motility in males of reproductive age1.13 (95% CI: 0.53–2.39, 6 studies or participant data noted)<br>No association between MMP levels of 9.0-745.0ug/L and low sperm Motilityin males of reproductive age 0.71 (95% CI: 0.39–1.32, 6 studies or participant data noted)	No subgroup analysis	
				Sperm Morphology<br>OR and (95% CI)	No association between MMP levels of 1.5-9.0ug/L and low sperm Morphology in males of reproductive age 0.78 (95% CI: 0.43–1.40, 6 studies or participant data noted)<br>No association between MMP levels of 9.0-745.0ug/L and low sperm Morphology in males of reproductive age 0.84 (95% CI: 0.44–1.60, 6 studies or participant data noted)	No subgroup analysis	
				Sperm motion parameters: Straight Line velocity (VSL)	No association between MMP levels of 1.5–8.3 and Straight-line	No subgroup analysis	

				Beta co-efficients and 95% Confidence intervals	velocity (VSL) in men of reproductive age (b=-0.14 95%CI: -1.76, 1.49, 3 studies, no participant data noted)<br>No association between MMP levels of 8.3–278.1 and Straight-line velocity (VSL) in men of reproductive age (b=0.79 95%CI: -1.29, 2.88, 3 studies, no participant data noted)		
				Sperm motion parameters: Curvilinear velocity (VCL)<br>Beta co-efficients and 95% Confidence intervals	No association between MMP levels of 1.5–8.3 and Curvilinear velocity (VCL) in men of reproductive age (b=-1.54 95%CI: -4.31, 1.24, 3 studies, no participant data noted)<br>No association between MMP levels of 8.3–278.1 and Curvilinear velocity (VCL) in men of reproductive age (b=.18 95%CI: -3.39, 3.74, 3 studies, no participant data noted)	No subgroup analysis	
				Sperm motion parameters: Linearity (LIN)<br>Beta co-efficients and 95% Confidence intervals	No association between MMP levels of 1.5–8.3 and Linearity (LIN) in men of reproductive age (b=0.99 95%CI: -0.17, 2.14, 3 studies, no participant data noted)<br>No association between MMP levels of 8.3–278.1 and Linearity (LIN)in men of reproductive age (b=.93 95%CI: -0.61, 2.47, 3 studies, no participant data noted)		
				Comet Assay Parameters: Comet extent	No association between MMP (IQR 8.85UG/l) and Comet extent	No subgroup analysis	

				Beta co-efficients and 95% Confidence intervals	in men of reproductive age (b=-02.08 95%CI: -10.89, 6.73, 2 studies, no participant data noted)		
				Comet Assay Parameters: Percent DNA in tails<br>Beta co-efficients and 95% Confidence intervals	No association between MMP (IQR 8.85UG/l) and Percent DNA in tail in men of reproductive age (b=-2.44 95%CI: -7.16, 2.29, 2 studies, no participant data noted)	No subgroup analysis	
				Comet assay parameters: Tail distributed moment<br>Beta co-efficients and 95% Confidence intervals	No association between MMP (IQR 8.85UG/l) and Tail distributed moment in men of reproductive age (b=0.31 95%CI: -1.23, 1.84, 2 studies, no participant data noted)	No subgroup analysis	
			**Type**<br>MEP<br><br>**Route**<br>Environmental<br><br>**Measure**<br>Urinary phthalates or metabolite levels; Seminal phthalates levels; Serum phthalate levels<br><br>**Exposure time**<br>non-specific	Sperm Concentration<br>OR and (95% CI)	No association between MEP levels of 3.3-49.8ug/L and reduced sperm concentration in males of reproductive age 0.84 (95% CI:0.43–1.63, 6 studies no participant data noted)<br>No association between MEP levels of 77.2-11371ug/L and low sperm concentration in males of reproductive age 1.42 (95% CI:0.84–2.42, 6 studies no participant data noted)	No subgroup analysis	
				Sperm motility<br>OR and (95% CI)	No association between MEP levels of 3.3-49.8ug/L and low sperm Motility in males of reproductive age 0.77 (95% CI:0.30–1.96, 6 studies no participant data noted)<br>No association between MEP levels of 77.2-11371ug/L and low	No subgroup analysis	

					sperm Motility in males of reproductive age 0.89 (95% CI:0.59–1.32, 6 studies no participant data noted)		
				Sperm Morphology<br>OR and (95% CI)	No association between MEP levels of 3.3-49.8ug/L and reduced sperm Morphology in males of reproductive age 0.88 (95% CI:0.44–1.75, 6 studies no participant data noted)<br>No association between MEP levels of 77.2-11371ug/L and low sperm Morphology in males of reproductive age1.21 (95% CI:0.42–3.42, 6 studies no participant data noted)	No subgroup analysis	
				Sperm motion parameters:<br>Straight Line velocity (VSL)<br>Beta co-efficients and 95% Confidence intervals	No association between MEP levels of 59.6–979.5 and Straight-line velocity (VSL) in men of reproductive age (b= 0.12 95%CI: -0.88, 1.12, 3 studies, no participant data noted)<br>Exposure to MEP levels of 979.5–11,371.0 was associated with Straight line velocity (VSL) in men of reproductive age (b=2.36 95%CI:0.28, 4.45, 3 studies, no participant data noted)	No subgroup analysis	
				Sperm motion parameters:<br>Curvilinear velocity (VCL)<br>Beta co-efficients and 95% Confidence intervals	No association between MEP levels of 59.6–979.5 and Curvilinear velocity (VCL)in men of reproductive age (b= -0.21 95%CI: -1.80, 1.38, 3 studies, no participant data noted)<br>Exposure to MEP levels of 979.5–11,371.0 was associated with	No subgroup analysis	

					Curvilinear velocity (VCL) in men of reproductive age (b= .23 95%CI:1.67, 8.80, 3 studies, no participant data noted)		
				Sperm motion parameters: Linearity (LIN)<br>Beta co-efficients and 95% Confidence intervals	No association between MEP levels of 59.6–979.5 and Linearity (LIN) in men of reproductive age (b=0.01 95%CI: -0.82, 0.85, 3 studies, no participant data noted)<br>No association between MEP levels of 979.5–11,371.0 and Linearity (LIN) in men of reproductive age (b= -0.44 95%CI: -1.94, 1.04, 3 studies, no participant data noted)	No subgroup analysis	
				Comet Assay Parameters: Comet extent<br>Beta co-efficients and 95% Confidence intervals	Exposure to MEP (IQR 449.4UG/l) was associated with Comet extent in men of reproductive age (b=4.22 95%CI:1.66, 6.77, 2 studies, no participant data noted)	No subgroup analysis	
				Comet Assay Parameters: Percent DNA in tails<br>Beta co-efficients and 95% Confidence intervals	No association between MEP (IQR 449.4UG/l) and Percent DNA in tail in men of reproductive age (b=-0.18 95%CI: -0.79, 0.44, 2 studies, no participant data noted)	No subgroup analysis	
				Comet assay parameters: Tail distributed moment<br>Beta co-efficients and 95% Confidence intervals	Exposure to MEP (IQR 449.4UG/l) was associated with Tail distributed moment in men of reproductive age (b=1.64 95%CI:0.24, 3.03, 2 studies, no participant data noted)	No subgroup analysis	
			**Type**<br>MEHP	Sperm Concentration<br>OR and (95% CI)	No association between MEHP levels 0.4–1.9ug/L and low sperm	No subgroup analysis	

			**Route**<br>Environmental<br><br>**Measure**<br>Urinary phthalates or metabolite levels; Seminal phthalates levels; Serum phthalate levels<br><br>**Exposure time**<br>non-specific		concentration in males of reproductive age 8.00 (95% CI:1.00–60.30, 6 studies no participant data noted)<br>No association between 3.8–875.8ug/L and reduced sperm concentration in males of reproductive age 0.99 (95% CI:0.64–1.54, 6 studies no participant data noted).<br><br>Using Beta coefficients, no associations between (MEHP-no levels listed) and sperm concentration in males of reproductive age (b= -001 95% CI: -017,0.17, 3 studies, no participant data noted)		
				Sperm Motility<br>OR and (95% CI)	No association between MEHP levels of 0.4–1.9ug/L and low sperm Motility in males of reproductive age 0.70 (95% CI:0.20–2.00, 6 studies no participant data noted)<br>No association between MEHP levels of 3.8–875.8ug/L and low sperm Motility in males of reproductive age 1.17 (95% CI:0.78–1.76, 6 studies no participant data noted)<br><br>Using beta-coefficients no association between MEHP (no levels listed) and sperm motility in males of reproductive age b=	No subgroup analysis	

					94.62 (95%CI: -176.54, 365.77, 3 studies, no participant data noted)		
				Sperm Morphology<br>OR and (95% CI)	No association between 3.8–875.8ug/L and low sperm Morphology in males of reproductive age 1.00 (95% CI:0.66–1.51, 6 studies no participant data noted)<br>No other levels reported for sperm morphology<br><br>Using beta-coefficients no association between MEHP (no levels listed) and low sperm morphology in males of reproductive age b= 0.19 (95%CI: -0.40, 0.79, 3 studies, no participant data noted)	No subgroup analysis	
				Sperm motion parameters:<br>Straight Line velocity (VSL)<br>Beta co-efficients and 95% Confidence intervals	Exposure to MEHP levels of 3.1–208.1 was associated with Straight line velocity (VSL) in men of reproductive age (b=-1.06 95%CI: -1.99, -0.12, 3 studies, no participant data noted)<br>No association between MEHP levels of 208.1–875.8 and Straight-line velocity (VSL) in men of reproductive age (b= -1.76 95%CI: -3.83, 0.31, 3 studies, no participant data noted)	No subgroup analysis	
				Sperm motion parameters:<br>Curvilinear velocity (VCL)<br>Beta co-efficients and 95% Confidence intervals	No association between MEP levels of 3.1–208.1 and Curvilinear velocity (VCL)in men of reproductive age (b=-1.48 95%CI: -2.99, 0.03, 3 studies, no	No subgroup analysis	

					participant data noted)<br>No association between MEHP levels of 208.1–875.8 and Curvilinear velocity (VCL)in men of reproductive age (b=-2.41 95%CI: -5.96, 1.15, 3 studies, no participant data noted)		
				Sperm motion parameters: Linearity (LIN)<br>Beta co-efficients and 95% Confidence intervals	Exposure to MEHP levels of 3.1–208.1 was associated with Linearity (LIN) in men of reproductive age (b=-0.43 95%CI: -0.80, -0.06, 3 studies, no participant data noted)<br>No association between MEHP levels of 208.1–875.8 and Linearity (LIN) in men of reproductive age (b=-0.43 95%CI: -1.90, 1.05, 3 studies, no participant data noted)	No subgroup analysis	
				Comet Assay Parameters: Comet extent<br>Beta co-efficients and 95% Confidence intervals	No association between MEHP (IQR 14.35UG/l) and Comet extent in men of reproductive age (b=-0.16 95%CI: -1.45, 1.13, 2 studies, no participant data noted)	No subgroup analysis	
				Comet Assay Parameters: Percent DNA in tails<br>Beta co-efficients and 95% Confidence intervals	No association between MEHP (IQR 14.35UG/l) and Percent DNA in tail in men of reproductive age (b=1.40 95%CI: -1.60, 4.40, 2 studies, no participant data noted)	No subgroup analysis	
				Comet assay parameters: Tail distributed moment<br>Beta co-efficients and 95% Confidence intervals	No association between MEHP (IQR 14.35UG/l) and Tail distributed moment in men of reproductive age (b=0.01 95%CI: -	No subgroup analysis	

					0.53, 0.54, 2 studies, no participant data noted)		
			**Type**<br>MEOHP<br><br>**Route**<br>Environmental<br><br>**Measure**<br>Urinary phthalates or metabolite levels; Seminal phthalates levels; Serum phthalate levels<br><br>**Exposure time**<br>non-specific	Sperm Concentration<br>OR and (95% CI)	No association between MEOHP levels of 1.9–30.6ug/L and low sperm concentration in males of reproductive age 1.66 (95% CI:0.50–5.50, 6 studies no participant data noted)<br>No association between MEOHP levels of 32.1–3063.0ug/L and low sperm concentration in males of reproductive age 1.30 (95% CI:0.45–3.75, 6 studies no participant data noted)	No subgroup analysis	
				Sperm motility<br>OR and (95% CI)	No association between MEOHP levels of 1.9–30.6ug/L and low sperm Motility in males of reproductive age 0.84 (95% CI:0.47–1.50, 6 studies no participant data noted)<br>No association between MEOHP levels of 32.1–3063.0ug/L and low sperm Motility in males of reproductive age 0.66 (95% CI:0.33–1.31, 6 studies no participant data noted)	No subgroup analysis	
				Sperm Morphology<br>OR and (95% CI)	No association between MEOHP levels of 1.9–30.6ug/L and low sperm Morphology in males of reproductive age1.40 (95% CI:0.50–3.70, 6 studies no participant data noted)<br>No association between MEOHP levels of 32.1–3063.0ug/L and low sperm Morphology in males of reproductive age0.59 (95%	No subgroup analysis	

					CI:0.26–1.33, 6 studies no participant data noted)		
			**Type**<br>MEHP AND MEOHP<br><br>**Route**<br>Environmental<br><br>**Measure**<br>Urinary phthalates or metabolite levels; Seminal phthalates levels; Serum phthalate levels<br><br>**Exposure time**<br>non-specific	Sperm Concentration<br>OR and (95% CI)	No association between MEHP, MEOHP levels of 2.2–84.2ug/L and low sperm concentration in males of reproductive age 1.16 (95% CI:0.67–2.03, 6 studies no participant data noted)<br>No association between MEHP, MEOHP levels of 93.9–3938.8ug/L and low sperm concentration in males of reproductive age 0.94 (95% CI:0.48–1.81, 6 studies no participant data noted)	No subgroup analysis	
				sperm motility<br>OR and (95% CI)	No association between MEHP, MEOHP levels of 2.2–84.2ug/L and low sperm Motility in males of reproductive age 0.94 (95% CI:0.62–1.43, 6 studies no participant data noted)<br>No association between MEHP, MEOHP levels of 93.9–3938.8ug/L and low sperm Motility in males of reproductive age 0.99 (95% CI:0.62–1.60, 6 studies no participant data noted)	No subgroup analysis	
				Sperm Morphology<br>OR and (95% CI)	No association between MEHP, MEOHP levels of 2.2–84.2ug/L and reduced sperm Morphology in males of reproductive age1.01 (95% CI:0.56–1.81, 6 studies no participant data noted)<br>No association between MEHP, MEOHP levels of 93.9–3938.8ug/L and low sperm Morphology in males of reproductive age 0.70	No subgroup analysis	

					(95% CI:0.41–1.20, 6 studies no participant data noted)		
			**Type**<br>DBP<br><br>**Route**<br>Environmental<br><br>**Measure**<br>Urinary phthalates or metabolite levels; Seminal phthalates levels; Serum phthalate levels<br><br>**Exposure time**<br>non-specific	Sperm Motility (from semen levels)<br>Beta co-efficients and 95% Confidence intervals	Exposure to DBP was negatively associated with sperm motility (b= -0.19, 95%CI: -0.28 to - 0.1; 2 studies, no participant data noted)	No subgroup analysis	
			**Type**<br>DEHP and DEHP metabolites<br><br>**Route**<br>Environmental<br><br>**Measure**<br>Urinary phthalates or metabolite levels; Seminal phthalates levels; Serum phthalate levels<br><br>**Exposure time**<br>non-specific	Sperm Concentration<br>OR and (95% CI)	No association between DEHP metabolite levels of 23.2–79.5ug/L and low sperm concentration in males of reproductive age 1.20 (95% CI:0.74–1.94, 6 studies no participant data noted)<br>No association between DEHP metabolite levels of 79.5–8744.8ug/L and low sperm concentration in males of reproductive age 1.32 (95% CI:0.62–2.80, 6 studies no participant data noted)	No subgroup analysis	
				Sperm Motility<br>OR and (95% CI)	No association between DEHP metabolite levels of 23.2–79.5ug/L and reduced sperm Motility in males of reproductive age 1.01 (95% CI:0.57–1.78, 6 studies no participant data noted)	No subgroup analysis	

					No association between DEHP metabolite levels of 79.5–8744.8ug/L and low sperm Motility in males of reproductive age 0.88 (95% CI:0.57–1.37, 6 studies no participant data noted)		
				Sperm Motility (from semen levels)<br>Beta co-efficients and 95% Confidence intervals	Exposure to DEHP was negatively associated with low sperm motility (b= -0.21, 95%CI: -0.3 to -0.12; 2 studies, no participant data noted)	No subgroup analysis	
				Sperm Morphology<br>OR and (95% CI)	No association between DEHP metabolite levels of 23.2–79.5ug/L and low sperm Morphology in males of reproductive age 1.28 (95% CI:0.85–1.93, 6 studies no participant data noted)<br>No association between DEHP metabolite levels of 79.5–8744.8ug/L and reduced sperm Morphology in males of reproductive age 1.10 (95% CI:0.54–2.25, 6 studies no participant data noted)	No subgroup analysis	

2.4 Endocrine outcomes

Study details	Appraisal details	Participants	Plastic exposure	Health outcomes	Findings	Subgroup Findings	AMSTAR score
**Hu et al., 2018 [73] - The association between the environmental endocrine disruptor bisphenol A and polycystic ovary syndrome: a systematic review and meta-analysis. No COI declared**							

Study details	Appraisal details	Participants	Plastic exposure	Health outcomes	Findings	Subgroup Findings	AMSTAR score
**Last search**<br>Aug-17<br><br>**Study types**<br>Case-control (n=9)<br><br>**Included studies in the review =** 9<br><br>**Included studies in the meta-analysis** = 8 (2 studies with subsets)	Newcastle-Ottawa quality assessment scale (NOS) - studies medium to high	Women (PCOS) age and BMI matched controls<br><br>N = 933	**Type**<br>BPA<br><br>**Route**<br>NR<br><br>**Measure**<br>serum (n=8); follicular fluid (n=1) - ng/ml<br><br>**Exposure time**<br>NR	Polycystic ovary syndrome (PCOS)<br>SMD and 95%CI	Women with PCOS in women were found to have significantly higher BPA levels than women without PCOS (SMD 2.437; 95%CI: 1.265, 3.609, 11 studies, 933 participants)	Serum samples<br>SMD 2.515, 95%CI: 1.241 to 3.789; 10 studies, participants unspecified<br><br>>19 years<br>SMD 2.311, 95%CI: 1.009 to 3.612; 9 studies, participants unspecified	9
**Shoshtari-Yeganeh et al., 2019 [69] - Systematic review and meta-analysis on the association between phthalates exposure and insulin resistance. No COI reported**							
**Last search**<br>Jan-18<br><br>**Study types**<br>Cross sectional<br><br>**Included studies in the review =** 8<br><br>**Included studies in the meta-analysis** = 8	The authors have stated that they used assessed the quality of all included studies using the "Cochrane checklist" the results of this assessment are not provided in the manuscript or as supplementary material.	Participants aged from 12-79 years in the included studies. 3 studies from North American, 4 studies from Asia, 1 study from Europe<br><br>N = 13,808	**Type**<br>Any type of phthalate<br><br>**Route**<br>Unspecified<br><br>**Measure**<br>Unspecified only regression coefficients reported<br><br>**Exposure time**<br>Unspecified	Homeostatic model assessment - Insulin Resistance (HOMA-IR)<br>Beta-coefficient effect size (transformed z-score)	Overall analyses excluded due to unit of analysis error	Subgrouping occurred based on phthalate compound.<br>Positive associations were found for insulin resistance and the following compounds:<br>Mono-butyl phthalate (MBP) (b=0.13, 95%CI 0.07,0.19; 6216 participants)<br>Mono-benzyl phthalate (MBzP) (b=0.05, 95CI: 0.01, 0.10; 5 studies, 11439 participants)<br>Mono-(3-carboxypropyl) Phthalate (MCCP) (b=0.15, 95%CI: 0.03,0.28; 3 studies, 1908 participants)<br>Mono(2-ethyl-5-carboxypentyl) phthalate (MECPP) (b=0.16,	4

Study details	Appraisal details	Participants	Plastic exposure	Health outcomes	Findings	Subgroup Findings	AMSTAR score
						95%CI: 0.05, 0.27; 2 studies, 1122 participants)<br>Mono-(2-ethylhexyl) phthalate (MEHP) (b=0.08, 95%CI: 0.03, 0.12; 7 studies,13248 participants)<br>Mono(2-ethyl-5-oxohexyl) phthalate<br>(MEOHP) (b=0.10, 95%CI: 0.01, 0.19; 5 studies, 7795 participants)<br>Mono-iso-butyl phthalate (MiBP) (b=0.10, 95%CI: 0.03, 0.17; 4 studies, 6569 participants)<br>MnBP (Abbreviation not explained) (b= 0.13, 95%CI: 0.06, 0.2; 3 studies,5783 participants)<br>T.DEHP (Abbreviation not explained) (b=0.26, 95%CI 0.15, 0.38; 2 studies 4997 participants).<br><br>No association were found for insulin resistance and the following compounds:<br>Mono(2-ethyl-5-hydroxyhexyl) phthalate (MEHHP) (b=0.09 95%CI -0.01, 0.18; 5 studies, 7795 participants)<br>Mono-ethyl phthalate (MEP) (b=0.02, 95%CI: -0.04, 0.08; 6 studies, 12455 participants)<br>Mono-methyl phthalate (MMP)	

Study details	Appraisal details	Participants	Plastic exposure	Health outcomes	Findings	Subgroup Findings	AMSTAR score
						(b=0.02, 95%CI: -0.06, 0.11; 3 studies, 2158 participants)	
**Hwang et al., 2018 [65] - Bisphenol A exposure and type 2 diabetes mellitus risk: a meta-analysis. No COI declared**							
**Last search**<br>Dec-18<br><br>**Study types**<br>Cross-sectional (n=12); Case-control (n=3); Observational (n=1)<br><br>**Included studies in the review =**<br>16<br><br>**Included studies in the meta-analysis**<br>= 16	Downs and Black score. The average quality score was 16 with scores ranging from 13 to 18	NR<br><br>N = 41320	**Type**<br>Bisphenol A (BPA)<br><br>**Route**<br>NR<br><br>**Measure**<br>Urinary and serum BPA levels (ng/mL)<br><br>**Exposure time**<br>NR	Risk of T2DM<br>OR and 95%CI	BPA exposure was positively associated with T2DM risk in humans (OR [fixed effects model] 1.28; 95% CI: 1.14-1.44, I2=89.2%, p=0.000; 16 studies).<br><br>Sensitivity analysis (after exclusion of serum BPA levels and high heterogeneity)<br>Random effects<br>OR 1.20, 95% CI: 1.09 to 1.31; 14 studies; 38,059 participants	Urine samples<br>OR 1.01, 95%CI: 1.00 to 1.02; 14 studies, 38,298 participants<br>Serum samples<br>OR 1.59, 95%CI: 1.06 to 2.38; 2 studies, 3,022 participants	6
**Rancière et al., 2015 [66] - Bisphenol A and the risk of cardiometabolic disorders: a systematic review with meta-analysis of the epidemiological evidence. No COI declared**							
**Last search**<br>Aug-14<br><br>**Study types**<br>Cross-sectional (n=28) and Prospective Longitudinal (n=5) (pooled together)	A scoring system based on the established OHAT guidelines [31] adapted to reflect the characteristics of the included studies: longitudinal design (2 points), population-based	Adults or children (however only diabetes in adults, in an attempt to limit the analysis to type 2).<br>Pregnant	**Type**<br>Bisphenol A<br><br>**Route**<br>Non-specific<br><br>**Measure**<br>Urinary BPA and amniotic fluid (µg/L). Compared with extreme categories	Diabetes<br>OR with 95% CI	Exposure to BPA was +ve associated with prevalent diabetes in the general population 1.47 (95%CI: 1.21,1.80,3 studies, 9291 participants; lowest to highest quartile)	No subgroup analysis	**7**

Study details	Appraisal details	Participants	Plastic exposure	Health outcomes	Findings	Subgroup Findings	AMSTAR score
**Included studies in the review =** 33<br><br>**Included studies in the meta-analysis** = 12	study (1 point), outcome assessment including measurements (1 point), collection of at least 2 urine samples per participant (1 point), control for urine dilution (1 point), adjustment for dietary intake (1 point), and adjustment for socioeconomic variables (1 point). Studies were then classified as 'low quality' (total score between 0 and 2), 'medium quality' (total score between 3 and 5), or 'high quality' (total score between 6 and 8). Studies ranged from Low, medium to High. No discussion on the implications of quality on findings.	women were excluded.<br><br>N = 69,486	of urinary BPA levels (the highest vs. the lowest). Highest levels found a mean (SE) of 5.0 (0.3) ng/mL in boys and 4.6 (0.3) ng/mL in girls. Lowest: a median (interquartile range, IQR) of 0.60 (0.20–1.37) ng/mL.<br><br>**Exposure time**<br>non-specific				

Study details	Appraisal details	Participants	Plastic exposure	Health outcomes	Findings	Subgroup Findings	AMSTAR score
**Song et al., 2016 [67] - Endocrine-disrupting chemicals, risk of type 2 diabetes, and diabetes-related metabolic traits: A systematic review and meta-analysis. No COI declared.**							
**Last search**<br>Mar-14<br><br>**Study types**<br>cross-sectional (n=41), cohort (n=8)<br><br>**Included studies in the review =** 49<br><br>**Included studies in the meta-analysis** = 32	Unspecified	Unspecified<br><br>N = 55,774	**Type**<br>BPA<br><br>**Route**<br>Unspecified<br><br>**Measure**<br>Urinary (ng/mL)<br><br>**Exposure time**<br>Unspecified	Type 2 diabetes<br>Risk ratio and Cis; Highest versus Lowest exposure categories (cut offs NR)	There was a positive association between BPA exposure and type 2 diabetes (RR= 1.45, 95%CI: 1.13, 1.97; 4 studies, 10541 participants). Dose response MA from highest (>1.43 to >4.20 ng/mL) versus lowest (<0.47 to <1.36 ng/mL) exposure ranges and reported a dose response RR of 1.09 per 1ng/mL increase (95%CI 1.03 to 1.15).	No subgroup analysis	6
				Homeostatic Model Assessment of Insulin Resistance (HOMA-IR) Weighted mean difference and 95%Cis	Higher HOMA-IR was associated with higher BPA concentrations (WMD=0.80, 95%CI: 0.36-1.25; 4 studies, 6,520 participants).	No subgroup analysis	
				Fasting glucose<br>Weighted mean difference and 95%CIs	No association between BPA and fasting glucose (MD 0.97mg/dL, 95%CI: -0.19, 2.14; 4 studies, 9,854 participants)	No subgroup analysis	
				Fasting insulin<br>Weighted mean difference and 95%CIs	No association between BPA and fasting insulin (MD 0.15, 95%CI; -0.12 - 0.41; 4 studies, 9,854 participants)	No subgroup analysis	
			**Type**<br>Phthalates<br><br>**Route**<br>NR<br><br>**Measure**<br>Urinary (ng/mL)<br><br>**Exposure time**	Type 2 diabetes<br>RR and 95%CI; Highest versus Lowest exposure categories; MEP > 17.5 ng/mL) with lowest (MEP ≤7.2 ng/mL)	No association between phthalates and type 2 diabetes (RR=1.48, 95%CI: 0.98, 2.25; 4 studies, 5307 participants).	Higher concentrations of monoethyl phthalate (MEP) was associated with increased risk of type 2 diabetes (RR=1.39, 95%CI: 0.55, 3.48; 4 studies and participants not reported). Higher concentrations of Monoisobutyl phthalate (MiBP) was associated with increased risk of type 2 diabetes (RR=1.90,	

Study details	Appraisal details	Participants	Plastic exposure	Health outcomes	Findings	Subgroup Findings	AMSTAR score
			NR			95%CI: 1.17, 3.09; no. studies and participants not reported)	
				Fasting glucose	Mean difference between highest and lowest concentration 0.98 mg/dL (95% CI 0.00–1.97 mg); 3 studies, 3926 participants		
				Homeostatic model assessment - Insulin Resistance (HOMA-IR)	Pooled WMD of all metabolites 0.71 (95% CI 0.30–1.12); 4 studies, 5396 participants		
			**Type**<br>Polychlorinated biphenyls (PCB)<br><br>**Route**<br>Unspecified<br><br>**Measure**<br>serum or urinary (pg/ml or pg/g or ng/g lipid)<br><br>**Exposure time**<br>Unspecified	Type 2 diabetes<br>RR and 95%CI; Highest versus Lowest exposure categories; highest (PCB153 concentrations of >104 to >1348 ng/g lipid) and lowest (corresponding to PCB153 concentrations of ≤60 to ≤455 ng/g lipid)	There was a positive association between PCB and type 2 diabetes (RR=2.39, 95%CI: 1.86, 3.08; 21 studies, 18100 participants)	The RR was greater in women (RR=2.65, 95%CI: 1.57, 4.48; No. studies and participants not reported) than men (RR=1.73, 95%CI:0.80, 3.75; No. studies and participants not reported).	
				Fasting glucose<br>MD and 95%CI; Highest versus Lowest exposure categories; highest (PCB153 concentrations of >104 to >1348 ng/g lipid) and lowest (corresponding to PCB153 concentrations of ≤60 to ≤455 ng/g lipid)	There was a positive association between PCB and fasting glucose (MD=3.27, 95%CI: 1.87 –4.67; 3 studies, 2882 participants)	No subgroup analysis	
				2-h glucose<br>MD and 95%CI; Highest versus Lowest exposure categories; highest (PCB153 concentrations of >104 to >1348 ng/g lipid) and lowest (corresponding to PCB153 concentrations of ≤60 to ≤455 ng/g lipid)	No association (MD= 0.72, 95%CI: −7.44 – 8.87; 2 studies, 836 participants)	No subgroup analysis	

Study details	Appraisal details	Participants	Plastic exposure	Health outcomes	Findings	Subgroup Findings	AMSTAR score
				Fasting insulin<br>MD and 95%CI; Highest versus Lowest exposure categories; highest (PCB153 concentrations of >104 to >1348 ng/g lipid) and lowest (corresponding to PCB153 concentrations of ≤60 to ≤455 ng/g lipid)	No association (MD −0.48, 95%CI: −2.06 –1.09; 3 studies, 2882 participants)	No subgroup analysis	
				2-h insulin<br>MD and 95%CI; Highest versus Lowest exposure categories; highest (PCB153 concentrations of >104 to >1348 ng/g lipid) and lowest (corresponding to PCB153 concentrations of ≤60 to ≤455 ng/g lipid)	No association (MD=−17.56, 95%CI; −59.06 –23.93; 2 studies; 836 participants)	No subgroup analysis	
				HOMA- IR<br>MD and 95%CI; Highest versus Lowest exposure categories; highest (PCB153 concentrations of >104 to >1348 ng/g lipid) and lowest (corresponding to PCB153 concentrations of ≤60 to ≤455 ng/g lipid)	No association (MD=−2.05, 95%CI: −4.65–0.56; 3 studies, 933 participants)	No subgroup analysis	
**Wu et al., 2013 [68] - Persistent Organic Pollutants and Type 2 Diabetes: A Prospective Analysis in the Nurses' Health Study and Meta-analysis. No COIs declared**							
**Last search**<br>Dec-11<br><br>**Study types**<br>Case-control (n=2)<br>Prospective cohort (n=4)	Cohort study with additional meta-analysis (2 nested case control included). No critical appraisal was performed	Adult men and women from the general population<br><br>N = 3,880	**Type**<br>Polychlorinated biphenyls (PCBs)<br><br>**Route**<br>Unspecified<br><br>**Measure**	Incident diabetes<br>OR and 95%CI<br>All fixed effect analyses presented, authors indicated similar results with random effects	Positive association between exposure to PCB and incidence of diabetes (OR=1.7, 95%CI:1.28, 2.27; 6 studies, 2413 participants). One study excluded (due to PCB and PCDF poisoning) OR 2.05,95% CI: 1.41 to 2.98; 5 studies, 2,035 participants.	No subgroup analysis	4

Study details	Appraisal details	Participants	Plastic exposure	Health outcomes	Findings	Subgroup Findings	AMSTAR score
NOTE* authors also include their own data in the meta-analysis (2 cohorts)<br><br>**Included studies in the review =** 7<br><br>**Included studies in the meta-analysis** = 7			Serum concentrations (lipid standardised (ng/g)<br><br>**Exposure time**<br>Unspecified		Estimate OR 1.06 (95%CI 1.02 to 1.09) ng/g serum increase in total PCBs		
			**Type**<br>PCB – 118<br><br>**Route**<br>Unspecified<br><br>**Measure**<br>Serum concentrations (lipid standardised (ng/g)<br><br>**Exposure time**<br>Unspecified	Incident diabetes<br>OR and 95%CI	No association between exposure to PCB-118 and incidence of diabetes (OR=1.20, 95%CI:0.73, 1.96; 4 studies, 2,471 participants).	No subgroup analysis	
			**Type**<br>PCB – 138<br><br>**Route**<br>Unspecified<br><br>**Measure**<br>Serum concentrations (lipid standardised (ng/g)<br><br>**Exposure time**<br>Unspecified	Incident diabetes<br>OR and 95%CI	No association between exposure to PCB-138 and incidence of diabetes (OR=1.36, 95%CI:0.69,2.68; 2 studies, 1,820 participants)	No subgroup analysis	
			**Type**<br>PCB – 153<br><br>**Route**<br>Unspecified	Incident diabetes<br>OR and 95%CI	No association between exposure to PCB-153 and incidence of diabetes (OR=1.06, 95%CI: 0.79, 1.42; 4 studies, 2,742 participants)	No subgroup analysis	

Study details	Appraisal details	Participants	Plastic exposure	Health outcomes	Findings	Subgroup Findings	AMSTAR score
			**Measure**<br>Serum concentrations (lipid standardised (ng/g)<br><br>**Exposure time**<br>Unspecified				
			**Type**<br>PCB -180<br><br>**Route**<br>Unspecified<br><br>**Measure**<br>Serum concentrations (lipid standardised (ng/g)<br><br>**Exposure time**<br>Unspecified	Incident diabetes<br>OR and 95%CI	No association between exposure to PCB-180 and incidence of diabetes (OR=1.46, 95%CI:0.77,2.77; 3 studies, 2,000 participants)	No subgroup analysis	
**Kim et al., 2019 [71] - Association Between Diethylhexyl Phthalate Exposure and Thyroid Function: A Meta-Analysis. No COI declared**							
**Last search**<br>Oct-17<br><br>**Study types**<br>Cross-sectional, case-control and cohort (numbers not reported)<br><br>**Included studies in the review =** 13	None reported	Children (neonates excluded) and adolescents (aged < 18 years); pregnant women, adults (aged ≥ years), and general population<br><br>N = 12,674	**Type**<br>Diethylhexyl phthalate (DEHP) - metabolite (monoethylhexyl phthalate [MEHP])<br><br>**Route**<br>Unspecified<br><br>**Measure**<br>Urinary<br><br>**Exposure time**<br>Unspecified	Thyroid function (free thyroxine [fT4])<br>Pearson's correlation coefficient (r)	No association found between MEHP and fT4 levels (r=-0.02, 95%CI: -0.05,0.00, 10 studies, 4673 participants)	Children: No association found between MEHP and fT4 levels (r=0.03, 95%CI: -0.01,0.08, 6 studies, 1832 participants)<br>Pregnant Women: No association found between MEHP and fT4 levels (r=-0.04, 95%CI: -0.07,0.00, 4 studies, 2841 participants)<br>Adults: No association found between MEHP and fT4 levels (r=-0.03, 95%CI: -0.14,0.07, 2 studies, 1829 participants)	5
				Thyroid function (total free thyroxine [TT4])	No association found between MEHP and TT4 levels (r=0.01,	Children: No association found between MEHP and TT4 levels	

Study details	Appraisal details	Participants	Plastic exposure	Health outcomes	Findings	Subgroup Findings	AMSTAR score
**Included studies in the meta-analysis** = 13				Pearson's correlation coefficient (r)	95%CI: -0.03, 0.06, 13 studies, 5097 participants)	(r=0.02, 95%CI -0.04,0.07, 7 studies, 2061 participants)<br>Pregnant Women: No association found between MEHP and TT4 levels (r=-0.01, 95%CI: -0.13,0.11, 4 studies, 2841 participants)<br>Adults: No association found between MEHP and TT4 levels (r=-0.04, 95%CI: -0.08,0.01, 4 studies, 2024 participants)	
				Thyroid function (thyrotropin [TSH])<br>Pearson's correlation coefficient (r)	No association found between MEHP and TSH levels (r=-0.03, 95%CI: -0.07,0.01, 13 studies, 5096 participants)	Children: No association found between MEHP and TSH levels (r=-0.01, 95%CI: -0.05,0.04, 7 studies, 2060 participants)<br>Pregnant Women: No association found between MEHHP and fT4 levels (r=-0.04, 95%CI: -0.08,0.00, 3 studies, 2766 participants)<br>Adults: No association found between MEHHP and fT4 levels (r=-0.08, 95%CI: -0.14,0.01, 3 studies, 7832 participants)	
			**Type**<br>Diethylhexyl phthalate (DEHP) - metabolite (2-ethyl-5-hydroxyhexyl) phthalate [MEHHP])	Thyroid function (free thyroxine [fT4])<br>Pearson's correlation coefficient (r)	MEHHP was associated with fT4 levels (r=-0.03, 95%CI: -0.05, -0.01, 10 studies, 10,601 participants)	Children: MEHHP was associated with fT4 levels (r=0.06, 95%CI: 0.01,0.10, 6 studies, 1832 participants)<br>Pregnant Women: No association found between MEHHP and fT4 levels (r=-0.04, 95%CI: -0.08,0.00, 3 studies, 2766 participants)<br>Adults: An association found between MEHHP and fT4 levels (r=-0.08, 95%CI: -0.14, -0.01, 3 studies, 7832 participants)	

Study details	Appraisal details	Participants	Plastic exposure	Health outcomes	Findings	Subgroup Findings	AMSTAR score
				Thyroid function (total free thyroxine [TT4])<br>Pearson's correlation coefficient (r)	No association found between MEHHP and TT4 levels (r=0.03, 95%CI: -0.01,0.08, 11 studies, 10,830 participants)	Children: No association with TT4 levels (r=0.04, 95%CI: 0.00,0.09, 7 studies, 2061 participants)<br>Pregnant Women: No association found between MEHHP and TT4 levels (r=-0.00, 95%CI: -0.19,0.19, 3 studies, 2766 participants)<br>Adults: No association found between MEHHP and TT4 levels (r=0.00, 95%CI: -0.02,0.03, 3 studies, 7832 participants)	
				Thyroid function (thyrotropin [TSH])<br>Pearson's correlation coefficient (r)	No association found between MEHHP and TSH levels (r=-0.02, 95%CI -0.07,0.03, 10 studies, 4826 participants)	Children: No association found between MEHHP and TSH levels (r=-0.00, 95%CI: -0.05,0.05, 7 studies, 2060 participants)<br>Pregnant Women: No association found between MEHHP and TSH levels (r=-0.00, 95%CI: -0.20,0.19, 3 studies, 2766 participants)<br>Adults: No association found between MEHHP and TSH levels (r=-0.04, 95%CI: -0.09,0.00, 2 studies, 1829 participants)	
			**Type**<br>Diethylhexyl phthalate (DEHP) - mono (2-ethyl-5-oxohexyl) phthalate [MEOHP])	Thyroid function (free thyroxine [fT4])<br>Pearson's correlation coefficient (r)	No association found between MEOHP and fT4 levels (r-0.01, 95%CI: -0.03,0.01, 10 studies, 10,601 participants)	Children: No association found between MEOHP and fT4 levels (r=0.05, 95%CI: 0.00,0.10, 6 studies, 1832 participants)<br>Pregnant Women: No association found between MEOHP and fT4 levels (r=0.02, 95%CI: -0.05,0.10, 3 studies, 2766 participants)<br>Adults: No association found between MEOHP and fT4 levels	

Study details	Appraisal details	Participants	Plastic exposure	Health outcomes	Findings	Subgroup Findings	AMSTAR score
						(r=-0.05, 95%CI: -0.10,0.01, 3 studies, 7832 participants)	
				Thyroid function (total free thyroxine [TT4]) Pearson's correlation coefficient (r)	MEOHP was associated with TT4 levels (r=0.02, 95%CI: 0.00,0.04, 11 studies, 10,830 participants)	Children: MEOHP was associated with TT4 levels (r=0.05, 95%CI: 0.01,0.10, 7 studies, 2061 participants) Pregnant Women: No association found between MEOHP and TT4 levels (r=-0.03, 95%CI: -0.13,0.08, 3 studies, 2766 participants) Adults: No association found between MEOHP and TT4 levels (r=0.01, 95%CI: -0.01,0.03, 3 studies, 7832 participants)	
				Thyroid function (thyrotropin [TSH]) Pearson's correlation coefficient (r)	No association found between MEOHP and TSH levels (r-0.02, 95%CI: -0.07,0.03, 10 studies, 4826 participants)	Children: No association found between MEOHP and TSH levels (r=0.00, 95%CI -0.04,0.05, 7 studies, 2060 participants) Pregnant Women: No association found between MEOHP and TSH levels (r=0.03, 95%CI: -0.16,0.20, 3 studies, 2766 participants) Adults: No association found between MEOHP and TSH levels (r=-0.10, 95%CI: -0.221,0.03, 2 studies, 1829 participants)	
**Zhao et al., 2015 [72] - The Correlation between Polybrominated Diphenyl Ethers (PBDEs) and Thyroid Hormones in the General Population: A Meta-Analysis. No COI reported.**							
**Last search** Sep-14<br><br>**Study types** Cross-sectional	Agency for Healthcare Research and Quality (Rostom A,Dube C,Cranney A,Saloojee N,Sy R, Garritty C, et al.	general population (children and adults)<br><br>N = 2,922	**Type** Polybrominated diphenyl ethers (PBDEs)<br><br>**Route** Unspecified	Thyroid function - thyroid stimulating hormone (TSH) Transformation of Pearson correlation coefficients (r) to Fisher's z	Overall analyses excluded due to unit of analysis error	No association between median PBDEs levels < 30 ng/g lipid and TSH levels; The pooled z value of subgroup one was random effects z -0.07 (95%CI -0.14, 0.00; 10 studies, 1064 participants); Fixed effects z -0.07, 95%CI -0.13,	9

Study details	Appraisal details	Participants	Plastic exposure	Health outcomes	Findings	Subgroup Findings	AMSTAR score
**Included studies in the review =** 19<br><br>**Included studies in the meta-analysis** = 17	Celiac Disease. Rockville (MD): Agency for Healthcare Research and Quality (US); 2004 Sep.(Evidence Reports/Technology Assessments, No. 104.)		**Measure**<br>lipid ng/g<br><br>**Exposure time**<br>unspecified			-0.01; 10 studies, 1,064 participants	
				Thyroid function - total thyroxine (TT4)<br>Transformation of Pearson correlation coefficients (r) to Fisher's z		Exposure to PBDEs levels between 35 ng/g and 100 ng/g lipid was positively associated with TT4 levels random and fixed effects z 0.15, 95%CI: 0.06,0.24; 3 studies, 466 participants)	
**Kim et al., 2018 [70] - Association between perfluoroalkyl substances exposure and thyroid function in adults :A meta-analysis. No COIs declared**							
**Last search**<br>Apr-17<br><br>**Study types**<br>cross-sectional<br><br>**Included studies in the review =** 12<br><br>**Included studies in the meta-analysis** = 12	A modified cross-sectional assessment provided by the Agency for Healthcare Research. All articles scored in 6-8 range. Authors concluded that the quality of the cross-sectional studies did not affect the quality of the meta-analysis	Adults >18years; mean age (39-63.6) Sportfish anglers(n=31) General population (n=556) General Population (Inuit) (n=506) General Population (NHANES) (n=2863) General Population (Riverside)(n=87) Pregnant women (24-41 weeks) (n=392) Pregnant women (17-18 weeks) (n=903)	**Type**<br>PFOS<br><br>**Route**<br>Unspecified<br><br>**Measure**<br>blood ng/mL<br>< 8 ng/mL, (Low), 8±16 ng/mL (intermediate), and > 16 ng/mL (high).<br><br>**Exposure time**<br>unspecified	Thyroid Function: free T4 Pearson correlation coefficient transformed by the Fisher z-transformation.	Exposure to PFOS and free T4 was associated with increased free t4 levels in the total adult population. Pooled Z values 0.05 (0.03; 0.08, 9 studies, 4741 participants). Sensitivity analysis between models (fixed vs random) showed no difference.	Sub-analysis of different levels of free T4 conducted:<br><br>No correlation between Mean PFOS (<8ng/mL) and free T4. The pooled Z value was 0.05 (95% CI): -0.03; 0.13), 2 studies, 548 participants). Sensitivity analysis between fixed effects models and random effects model 0.04 (-0.9; 0.16) showed no difference<br><br>Mean PFOS (8-16ng/mL) was +ve correlated with increased free T4. The pooled Z value was 0.07 (95% CI): 0.02; 0.11, 3 studies, 1852 participants). Sensitivity analysis between fixed effects models and random effects model 0.07 (95% CI): 0.02; 0.11) showed no difference<br><br>Mean PFOS (>16ng/mL) was +ve	7

Study details	Appraisal details	Participants	Plastic exposure	Health outcomes	Findings	Subgroup Findings	AMSTAR score
		Pregnant women (unspecified on gestation) (n=440)<br>IVF clinic (n=246)<br>Women attempting to conceive (n=99)<br><br>N = 6,123				correlated with increased free T4. The pooled Z value was 0.05 (95% CI):1.01;0.09), 4 studies, 2341 participants). Sensitivity analysis between fixed effects models and random effects model occurred. Random effects model indicated no correlation between Mean PFOS (>16ng/mL) and free T4 0.03 (-0.01;0.13).<br><br>Sub-analysis between pregnant and general population: No association between PFOS and free T4 in pregnant women. Pooled Z values 0.05 (95% CI: -0.02;0.11, 3 studies). Exposure to PFOS was associated with increased free t4 levels in the general population. Pooled z values 0.06 (95% CI) 0.02;0.09, 6 studies).	
				Thyroid Function: Total T4 Pearson correlation coefficient transformed by the Fisher z-transformation.	Using Random effects model no association between PFOS and total T4 in total population. Pooled Z values 0.01 (95% CI):-0.05;0.07, 8 studies, 4489 participants)<br><br>Sensitivity analysis between models (fixed vs random) showed no difference. Fixed effects model: -0.03 (95%CI):-	Sub-analysis of different levels of total T4conducted:<br><br>no correlation between Mean PFOS (<8ng/mL) and total T4 in total population. The pooled Z value was 0.02 (95%CI): -0.10; 0.05), 2 studies, 713 participants)<br>Sensitivity analysis between fixed effects models and random effects model 0.02 (95% ci):-0.10;	

Study details	Appraisal details	Participants	Plastic exposure	Health outcomes	Findings	Subgroup Findings	AMSTAR score
					0.06;0.00, 8 studies, 4489 participants)	0.06) showed no difference<br><br>no correlation between Mean PFOS (8-16ng/mL) and total T4 in total population. The pooled Z value was -0.04 (95% CI): -0.09: 0.00 3 studies, 1839 participants)<br>Sensitivity analysis between fixed effects models and random effects model -0.01 (95% CI): - .11; 0.09) showed no difference<br><br>no correlation between Mean PFOS (>16ng/mL) and total T4 in total population. The pooled Z value was 0.02 (95% CI): - 0.06;0.02), 3 studies, 1937 participants).<br>Sensitivity analysis between fixed effects models and random effects model 0.09 (95% CI): - 0.11; 0.28)<br><br>Sub-analysis between pregnant women/ general population and Total T4:<br>No correlation between PFOS and total T4 in pregnant women. The pooled Z value was 0.06 (- 0.03;0.15, 2 studies).<br>No association between PFOS and total t4 in general population. Pooled Z value: 0.00 (-0.07;0.07, 6 studies)	

Study details	Appraisal details	Participants	Plastic exposure	Health outcomes	Findings	Subgroup Findings	AMSTAR score
				Thyroid Function: T3<br>Pearson correlation coefficient transformed by the Fisher z-transformation.	Using Random effects model no association between PFOS and total T3 in total population-0.02 (95% CI):-0.07;0.04, 8 studies, 4555 participants).<br>Sensitivity analysis between models (fixed vs random) showed the fixed effects model was -ve associated Pooled Z values (-0.05 (95%CI):-0.08; 0.02, 8 studies, 4555 participants).	Sub-analysis of different levels of total T3conducted:<br><br>Mean PFOS (8-16ng/mL) was associated with total T3 in total population. The pooled Z value was -0.05 (95% CI): -0.10: -0.01 3 studies, 1843 participants)<br>Sensitivity analysis between (fixed vs random) models. Random effects model showed no association -0.03 (95% CI): -.11; 0.06).<br><br>Mean PFOS (>16ng/mL) was associated with total T3 in total population. The pooled Z value was -0.05 (95% CI): -0.09;0.02), 4 studies, 2557 participants).<br>Sensitivity analysis between (fixed vs random) models. Random effects model showed no association 0.01 (95% CI): -0.10:0.11)<br><br>Sub-analysis between pregnant women/ general population and Total T3:<br>No correlation between PFOS and total T3 in pregnant women. The pooled Z value was -0.01 (-0.10;0.09, 2 studies).<br>No association between PFOS and total t3 in general	

Study details	Appraisal details	Participants	Plastic exposure	Health outcomes	Findings	Subgroup Findings	AMSTAR score
						population. Pooled Z value: -0.01 (-0.08;0.06, 6 studies).	
				Thyroid Function: TSH<br>Pearson correlation coefficient transformed by the Fisher z-transformation.	Using Random effects model no correlation between PFOS and total TSH in total population -0.02 (95% CI):-0.07; 0.03, 12 studies, 6445 participants). Sensitivity analysis between models (fixed vs random) showed no difference. Fixed effects Pooled Z values -0.01 (95%CI):-0.04; 0.01, 12 studies, 6445 participants).	Sub-analysis of different levels of total TSH (fixed-effects model reported when < 5 studies) with sensitivity analysis on model (Fixed vs random effects model) type conducted:<br><br>Mean PFOS (<8ng/mL) was correlated with total TSH in total population. The pooled Z value was - .10(95% ci):-0.16; -0.65), 3 studies, 1105 participants)<br>Sensitivity analysis between fixed effects models and random effects model. Random effects model showed no association -0.14 (95% CI) -0.28; 0.01).<br><br>No correlation between Mean PFOS (8-16ng/mL) and total TSH in total population. The pooled Z value was 0.03 (95% CI): 0.00; 0.07, 4 studies, 2753 participants)<br>Sensitivity analysis between (fixed vs random) models showed no difference 0.03 (95% CI): 0.00: 0.07, 4 studies, 2753 participants).<br><br>No correlation between Mean PFOS (>16ng/mL) and total TSH	

Study details	Appraisal details	Participants	Plastic exposure	Health outcomes	Findings	Subgroup Findings	AMSTAR score
						in total population. The pooled Z value was -0.02 (95% CI): -0.06;0.02), 5 studies, 2587 participants).<br>Sensitivity analysis between (fixed vs random) models. Random effects model showed no association -0.01 (95% CI): -0.08;0.07)<br><br>Sub-analysis between pregnant women/ general population and Total TSH:<br>No correlation between PFOS and total TSH in pregnant women. The pooled Z value was -0.08 (-0.12; 0.08, 4 studies).<br>No association between PFOS and total TSH in general population. Pooled Z value: -0.01 (-0.04; 0.02, 8 studies).	
			**Type**<br>PFHxS<br><br>**Route**<br>Unspecified<br><br>**Measure**<br>ng/mL<br>< 0.8 ng/mL (Low),<br>and 0.8 ng/mL (high).<br><br>**Exposure time**<br>unspecified	Thyroid Function: free t4 Pearson correlation coefficient transformed by the Fisher z-transformation.	Using Random effects model no correlation between PFHxS and free T4 in total population 0.02 (95% CI):-0.01; 0.05, 6 studies, 3641 participants).<br>Sensitivity analysis between models (fixed vs random) showed no difference. Fixed effects Pooled Z values 0.02 (95% CI):-0.01; 0.05, 6 studies, 3641 participants).	Sub-analysis of different levels of total PFHxS conducted:<br><br>No correlation between Mean PFHxS (<8ng/mL) and free T4 in total population. The pooled Z value was - 0.00(95% ci):-0.10; 0.09), 3 studies, 415 participants)<br>Sensitivity analysis between fixed effects models and random effects model. Random effects model showed no association -0.00(95% ci):-0.10; 0.09), 3	

Study details	Appraisal details	Participants	Plastic exposure	Health outcomes	Findings	Subgroup Findings	AMSTAR score
						studies, 415 participants)<br><br>No correlation between Mean PFHxS (>8ng/mL) and free T4 in total population. The pooled Z value was 0.02 (95% CI): -0.01; 0.06, 3 studies, 3226 participants)<br>Sensitivity analysis between (fixed vs random) models showed no difference. Random effects model: 0.02 (95% CI): -0.01; 0.06, 3 studies, 3226 participants)<br><br>Sub-analysis between pregnant women/ general population and Total TSH occurred:<br>No correlation between PFHxS and free T4 in pregnant women. The pooled Z value was 0.01 (-0.01; 0.05, 2 studies).<br>No association between PFHxS and free T4 in general population. Pooled Z value: 0.02 (-0.01; 0.05, 4 studies).	
				Thyroid Function: Total T4 Pearson correlation coefficient transformed by the Fisher z-transformation.	Using Random effects model no correlation between PFHxS and total T4 in total population 0.04 (95% CI):-0.04; 0.01, 6 studies, 4154 participants).<br>Sensitivity analysis between models (fixed vs random). Fixed effects model found exposure to	Sub-analysis of different levels of total PFHxS occurred (fixed-effects model reported when < 5 studies) with sensitivity analysis on model (Fixed vs random effects model) type conducted:<br><br>No correlation between Mean	

Study details	Appraisal details	Participants	Plastic exposure	Health outcomes	Findings	Subgroup Findings	AMSTAR score
					PFHxS was correlated with total T4:<br>Pooled Z values -0.04 (95% CI):-0.07; -0.01, 6 studies, 4154 participants).	PFHxS (<8ng/mL) and total T4 in total population. The pooled Z value was - 0.04(95% ci):-0.11; 0.02), 3 studies, 929 participants)<br>Sensitivity analysis between fixed effects models and random effects model. Random effects model showed no association -0.04(95% CI):-0.11; 0.02), 3 studies, 415 participants)<br><br>No correlation between Mean PFHxS (>8ng/mL) and total T4 in total population. The pooled Z value was -0.04 (95% CI): -0.07; 0.00, 3 studies, 3225 participants)<br>Sensitivity analysis between (fixed vs random) models showed no difference. Random effects model: -0.02 (95% CI): -0.09; 0.04, 3 studies, 3225 participants)<br><br>Sub-analysis between pregnant women/ general population and Total T4 occurred:<br>No correlation between PFHxS and total T4 in pregnant women. The pooled Z value was 0.01 (-0.18; 0.20, 2 studies).<br>Exposure to PFHxS correlated with total T4 in general	

Study details	Appraisal details	Participants	Plastic exposure	Health outcomes	Findings	Subgroup Findings	AMSTAR score
						population. Pooled Z value: -0.04 (-0.07; -0.01, 4 studies).	
				Thyroid Function: Total T3 Pearson correlation coefficient transformed by the Fisher z-transformation.	Using fixed effects model no correlation between PFHxS and total T3 in total population 0.00 (95% CI):-0.03; 0.04, 5 studies, 3600 participants).<br><br>Sensitivity analysis between models (fixed vs random) found no difference. random effects model: 0.00 (95% CI): -0.03; 0.04, 5 studies, 3600 participants)	Sub-analysis of different levels of total PFHxS conducted:<br><br>No correlation between Mean PFHxS (<8ng/mL) and total T3 in total population. The pooled Z value was - 0.03(95% CI): -0.13; 0.07), 2 studies, 375 participants) Sensitivity analysis between fixed effects models and random effects model. Random effects model showed no association 0.00(95% CI):-0.19; 0.19), 2 studies, 375 participants)<br><br>No correlation between Mean PFHxS (>8ng/mL) and total T3 in total population. The pooled Z value was 0.01 (95% CI): -0.03; 0.04, 3 studies, 3225 participants) Sensitivity analysis between (fixed vs random) models showed no difference. Random effects model: 0.01 (95% CI): - 0.03; 0.04, 3 studies, 3225 participants)<br><br>Sub-analysis between pregnant women/ general population and Total T3 occurred: No correlation between PFHxS	

Study details	Appraisal details	Participants	Plastic exposure	Health outcomes	Findings	Subgroup Findings	AMSTAR score
						and total T3 in pregnant women. The pooled Z value was 0.01 (-0.16; 0.14, 2 studies).<br>No correlation between PFHxS and total T3 in general population. Pooled z value: -0.04 (-0.03; 0.04, 4 studies).	
				Thyroid Function: TSH<br>Pearson correlation coefficient transformed by the Fisher z-transformation.	Using random effects model no correlation between PFHxS and total TSH in total population 0.00 (95% CI):-0.03; 0.04, 8 studies, 5099 participants).<br>Sensitivity analysis between models (fixed vs random) found no difference. Fixed effects model: 0.00 (95% CI): -0.03; 0.03, 8 studies, 5099 participants)	Sub-analysis of different levels of total PFHxS conducted:<br><br>No correlation between Mean PFHxS (<8ng/mL) and total TSH in total population. The pooled Z value was 0.01(95% CI):-0.03; 0.06), 5 studies, 1872 participants)<br>Sensitivity analysis between fixed effects models and random effects model. Random effects model showed no association 0.02(95% CI):-0.04; 0.07), 5 studies, 1872 participants)<br><br>No correlation between Mean PFHxS (>8ng/mL) and total TSH in total population. The pooled Z value was -0.00 (95% CI): -0.04; 0.03, 3 studies, 3227 participants)<br>Sensitivity analysis between (fixed vs random) models showed no difference. Random effects model: -0.01 (95% CI): -0.07; 0.04, 3 studies, 3227	

Study details	Appraisal details	Participants	Plastic exposure	Health outcomes	Findings	Subgroup Findings	AMSTAR score
						participants)<br><br>Sub-analysis between pregnant women/ general population and Total TSH occurred:<br>No correlation between PFHxS and total TSH in pregnant women. The pooled Z value was 0.00 (-0.12; 0.13, 3 studies).<br>No correlation between PFHxS and total TSH in general population. Pooled Z value: 0.00 (-0.04; 0.03, 5 studies).	
			**Type**<br>PFOA<br><br>**Route**<br>Unspecified<br><br>**Measure**<br>ng/mL<br><<br>2 ng/mL (Low), 2±3 ng/mL (intermediate), and > 3 ng/mL (high)<br><br>**Exposure time**<br>unspecified	Thyroid Function: FREE T4<br>Pearson correlation coefficient transformed by the Fisher z-transformation. T4 was measured differently: chemiluminescent immunoassay (n = 5 studies), radioimmunoassay (n = 3 studies), or enzyme-linked immunosorbent<br>assay (n = 1 studies). However, no measurement ranges of T4 were notes.	Using random effects model no correlation between PFOA and free T4 in total population 0.01 (95% CI):-0.02; 0.04, 8 studies, 4120 participants).<br>Sensitivity analysis between models (fixed vs random) found no difference. Fixed effects model:0.01 (95% CI):-0.02; 0.04, 8 studies, 4120 participants).	Sub-analysis of different levels of total PFOA occurred (fixed-effects model reported when < 5 studies) with sensitivity analysis on model (Fixed vs random effects model) type conducted:<br><br>No correlation between mean PFOA (<2ng/mL) and free T4 in total population. The pooled Z value was 0.02(95% CI):-0.08; 0.12), 2 studies, 423 participants)<br>Sensitivity analysis between fixed effects models and random effects model. Random effects model showed no association: 0.02 (95%CI):-0.08; 0.12), 2 studies, 423 participants)<br>No correlation between Mean PFOA(2-3 ng/mL) and free T4 in total population. The pooled Z	

Study details	Appraisal details	Participants	Plastic exposure	Health outcomes	Findings	Subgroup Findings	AMSTAR score
						value was 0.02 (95% CI): -0.03; 0.06, 4 studies, 2008 participants)<br>Sensitivity analysis between (fixed vs random) models showed no difference. Random effects model: 0.02 (95% CI): -0.03; 0.06, 4 studies, 2008 participants)<br><br>No correlation between Mean PFOA (>3ng/mL) and free T4 in total population. The pooled Z value was -0.00 (95% CI): -0.05; 0.05, 2 studies, 1689 participants)<br>Sensitivity analysis between (fixed vs random) models showed no difference. Random effects model: 0.05 (95% CI): -0.12; 0.21, 2 studies, 1689 participants)<br><br>Sub-analysis between pregnant women/ general population and PFOA AND FREE T4 occurred:<br>No correlation between PFOA and free T4 in pregnant women. The pooled Z value was 0.00 (-0.07; 0.06, 3 studies).<br>No correlation between PFOA and free T4 in general population. Pooled Z value: 0.01 (-0.02; 0.05, 5 studies).	

Study details	Appraisal details	Participants	Plastic exposure	Health outcomes	Findings	Subgroup Findings	AMSTAR score
				Thyroid function: Total T4 Pearson correlation coefficient transformed by the Fisher z-transformation. T4 was measured differently: chemiluminescent immunoassay (n = 5 studies), radioimmunoassay (n = 3 studies), or enzyme-linked immunosorbent assay (n = 1 studies). However, no measurement ranges of T4 were notes.	Using random effects model no correlation between PFOA and TOTAL T4 in total population 0.01 (95% CI):-0.07; 0.05, 8 studies, 4487 participants).<br>Sensitivity analysis between models (fixed vs random). FIXED effects model found<br>-ve correlation: -0.05 (95% CI):-0.08; -0.02, 8 studies, 4487 participants).	Sub-analysis of different levels of total PFOA conducted:<br><br>No correlation between Mean PFOA (2-3 ng/mL) and TOTAL T4 in total population. The pooled Z value was -0.04 (95% CI): -0.08; 0.00, 5 studies, 2552 participants)<br>Sensitivity analysis between (fixed vs random) models showed no difference. Random effects model: -0.00 (95% CI): -0.08; 0.07, 5 studies, 2552 participants)<br>Exposure between Mean PFOA (>3ng/mL) was -ve correlated with TOTAL T4 in total population. The pooled Z value was -0.06 (95% CI): -0.10; -0.01, 3 studies, 1935 participants)<br>Sensitivity analysis between (fixed vs random) model. Random effects model found no association: -0.00 (95% CI): -0.16; 0.16, 3 studies, 1689 participants)<br><br>Sub-analysis between pregnant women/ general population and PFOA AND TOTAL T4 occurred:<br>No correlation between PFOA and TOTAL in pregnant women. The pooled Z value was 0.04 (-	

Study details	Appraisal details	Participants	Plastic exposure	Health outcomes	Findings	Subgroup Findings	AMSTAR score
						0.06; 0.13, 2 studies).<br>No correlation between PFOA and TOTAL in general population. Pooled Z value: -0.03 (-0.09; 0.04, 6 studies).	
				Thyroid Function: T3<br>Pearson correlation coefficient transformed by the Fisher z-transformation.	Using random effects model no correlation between PFOA and TOTAL T3 in total population 0.05 (95% CI):0.00; 0.10, 7 studies, 3933 participants).<br>Sensitivity analysis between models (fixed vs random). Fixed effects model found no correlation: 0.03 (95% CI):0.00; 0.10, studies, 3933 participants).	Sub-analysis of different levels of total T3 conducted:<br><br>No correlation between Mean PFOA (2-3 ng/mL) and total T3 in total population. The pooled Z value was 0.02 (95% CI): -0.02; 0.06, 4 studies, 1998 participants)<br>Sensitivity analysis between (fixed vs random) models showed no difference. Random effects model: 0.05 (95% CI): -0.03; 0.14, 4 studies, 1998 participants)<br><br>No correlation between Mean PFOA (>3ng/mL) and total T3 in total population. The pooled Z value was 0.04 (95% CI): 0.00; 0.08, 3 studies, 1935 participants)<br>Sensitivity analysis between (fixed vs random) model. Random effects model found no association: 0.06 (95% CI): -0.02; 0.14, 3 studies, 1935 participants)	

Study details	Appraisal details	Participants	Plastic exposure	Health outcomes	Findings	Subgroup Findings	AMSTAR score
						Sub-analysis between pregnant women/ general population and PFOA and total T3 occurred:<br>No correlation between PFOA and total T3 in pregnant women. The pooled Z value was 0.04 (-0.05; 0.14, 2 studies).<br>No correlation between PFOA and total T3 in general population. Pooled Z value: 0.05 (-0.01; 0.11, 5 studies).	
				Thyroid Function: TSH<br>Pearson correlation coefficient transformed by the Fisher z-transformation.	Using random effects model no correlation between PFOA and TSH in total population 0.00 (95% CI):-0.03; 0.04, 11 studies, 5823 participants).<br>Sensitivity analysis between models (fixed vs random). fixed effects model found no correlation: 0.00 (95% CI):0.02; 0.03, 11 studies, 5823 participants).	Sub-analysis of different levels of total TSH conducted:<br><br>No correlation between Mean PFOA (<2ng/mL) and TSH in total population. The pooled Z value was 0.04(95% CI):-0.06; 0.13), 2 studies, 423 participants)<br>Sensitivity analysis between fixed effects models and random effects model. Random effects model showed no association: 0.04(95% CI):-0.06; 0.13), 2 studies, 423 participants)<br><br>No correlation between Mean PFOA (2-3 ng/mL) and TSH in total population. The pooled Z value was -0.01 (95% CI): -0.06; 0.04, 6 studies, 3466 participants)<br>Sensitivity analysis between (fixed vs random) models	

Study details	Appraisal details	Participants	Plastic exposure	Health outcomes	Findings	Subgroup Findings	AMSTAR score
						showed no difference. Fixed effects model: -0.00 (95% CI): -0.04; 0.03, 6 studies, 3466 participants)<br><br>No correlation between Mean PFOA (>3ng/mL) and TSH in total population. The pooled Z value was 0.00 (95% CI): -0.04; 0.05, 3 studies, 1934 participants)<br>Sensitivity analysis between (fixed vs random) model.<br>Random effects model found no association: 0.03 (95% CI): -0.06; 0.12, 3 studies, 1934 participants)<br><br>Sub-analysis between pregnant women/ general population and PFOA and TSH occurred:<br>No correlation between PFOA and TSH in pregnant women. The pooled Z value was 0.04 (-0.05; 0.14, 2 studies).<br>No correlation between PFOA and TSH in general population. Pooled Z value: 0.05 (-0.01; 0.11, 5 studies).	

2.5 Children's neurodevelopmental outcomes

Study details	Appraisal details	Participants	Plastic exposure	Health outcomes	Findings	Subgroup Findings	AMSTAR score
**Lam et al., 2017 [74] - Developmental PBDE Exposure and IQ/ADHD in Childhood: A Systematic Review and Meta-analysis. No COIs declared**							

Study details	Appraisal details	Participants	Plastic exposure	Health outcomes	Findings	Subgroup Findings	AMSTAR score
**Last Search**<br>Sep-16<br><br>**Study types**<br>Cross-sectional (n=1)<br>Prospective Cohort (n=13)<br>Case Control (n=1)<br><br>**Included studies in the review =** 15<br><br>**Included studies in the meta-analysis =** 4	Evaluated risk of bias using a modified instrument based on the Cochrane "Risk of Bias" tool and the Agency for Healthcare Research and Quality's (AHRQ) domains (i.e., selection bias, confounding, performance bias, attrition bias, detection bias, and reporting bias)<br><br>Risk of bias for studies of IQ was generally "low" or "probably low" across studies and domains. Studies that received "probably high ratings" evaluated outcomes related to IQ, such as infant/toddler assessments of intelligence (i.e., Bayley Scales), and these studies were not included in the meta-analysis that informed our final decision. As such, we agreed that these limitations within certain studies were not strong enough to warrant downgrading for risk of bias across all studies.	The authors state "humans", however reading this can be simplified to mother-child pairs studied during late-pregnancy. However, the only synthesised effect estimate presented is a comparison of prenatal PBDE exposure and IQ on children 48-84 months.<br><br>N = 2,884	**Type**<br>Polybrominated diphenyl ethers (PBDE)<br><br>**Route**<br>Unspecified, but detection limited to umbilical cord blood or maternal serum during gestation or at birth<br><br>**Measure**<br>Authors present [PBDE] in ln(10)-units. Verbatim from author "…overall pooled estimates from RE model per 10-fold increase (in other words, times 10) in PBDE exposure<br><br>**Exposure time**<br>Unspecified<br>But exposure was prenatal	IQ<br>Beta coefficient effect size (ln (10)).<br>All primary studies provided lipid adjusted estimates.	Prenatal exposure to PBDE was associated with a decrease in IQ points per 10-fold increase in PBDE concentration (lipid adjusted) (b = -3.7 points, 95% CI: -6.56, -0.83 points, (p-value not specified); 4 studies; 595 participants).	No subgroup or sensitivity analysis	11
**Lee et al., 2018 [75] - Prenatal and postnatal exposure to di-(2-ethylhexyl) phthalate and neurodevelopmental outcomes: A systematic review and meta-analysis. No COI declared**							
**Last search**<br>Sep-17<br><br>**Study types**<br>Cross-sectional (n=2);<br>Observational (n=8)<br><br>**Included studies in the review =** 10	Newcastle-Ottawa quality assessment scale (NOS) - low-quality (0–3), moderate-quality (4–6), or high-quality (7–9). Scores of the cohort studies (n = 8) ranged from 7 to 8, and the cross-sectional studies (n = 2) both scored at 8.	Age: 6 months to 12 years<br><br>N = 2,496	**Type**<br>Di-(2-ethylhexyl) phthalate (DEHP)<br><br>**Route**<br>pre-natal<br><br>**Measure**<br>pre-natal maternal urine (n=8); child's urine (n=2); both (n=3) -	Neurodevelopment measured with Weschsler Intelligence Scale for Children (WISC) and Bayley scales of infant development (BSID)	Longitudinal data: No association found between DEHP and neurodevelopment (WISC/BSID/MDI/FSIQ) in children (b=-0.14, 95%CI: -0.705, 0.41, 8 studies, 1625 participants).	Longitudinal data: No association found between DEHP and neurodevelopment (components of BSID; mental development index [MDI] in children (b= -0.36 95%CI -1.05,0.32, 5 studies, 871 participants).	
					Cross-sectional data: Exposure to DEHP was	No subgroup analysis conducted	7

Study details	Appraisal details	Participants	Plastic exposure	Health outcomes	Findings	Subgroup Findings	AMSTAR score
**Included studies in the meta-analysis** = 10			**Exposure time**<br>Unspecified	beta coefficient (b)	negatively associated with neurodevelopment (WISC/ FSIQ/cognitive) in children (b= -1.03, 95%CI -1.88, -0.18, 5 studies, 1462 participants).		
**Radke et al., 2020 [76] - Phthalate exposure and neurodevelopment: A systematic review and meta-analysis of human epidemiological evidence. No COIs reported**							
**Last search**<br>Mar-19<br><br>**Study type**<br>Prospective Cohort (n=25)<br>Case-control (n=1)<br>However different values provided in the review that do not align. These values have been taken from Table 2.<br><br>**Included studies in the review** = 26<br><br>**Included studies in the meta-analysis** = 7	ROBINS-I tool.<br>Four studies were classified as high confidence; ten studies were classified as medium confidence and three were classified as low confidence.	With one exception, all the included studies were birth cohorts with follow-up infancy to childhood (newborns to age 11 years), ranging in sample size between 135-657 children. The remaining study was a cohort of children admitted to the paediatric intensive care unit and followed at 4 years post-admission. All children included in meta-analysis were 4 years of age or younger.<br><br>N = 5,573	**Type**<br>di(2-ethylhexyl) phthalate (DEHP)<br><br>**Route**<br>Unspecified, but exposure level measured in urine or plasma<br>Varies between included study<br><br>**Measure**<br>nmol/mL<br>ng/mL<br>µg/g<br><br>**Exposure time**<br>Unspecified	Mental Development Index (MDI)<br>Beta coefficient effect size (natural units of the Bayley Scales of Infant Development)	No association between DEHP exposure and MDI (b= -0.1, 95%CI: -0.8, 0.5); 7 studies, 2536 participants).	No association between DEHP exposure and MDI in:<br>Girls<br>β −0.5, 95%CI: −2.2 to 1.2; studies and participants unspecified<br>Boys<br>β 0.1, 95%CI: −1.2 to 1.3; studies and participants unspecified	8
			**Type**<br>butyl benzyl phthalate (BBP)<br><br>**Route**<br>Unspecified, but exposure level measured in urine or plasma<br>Varies between included study<br><br>**Measure**<br>ng/mL<br>µg/g<br><br>**Exposure time**	Mental Development Index (MDI)<br>Beta coefficient effect size (natural units of the Bayley Scales of Infant Development)	No association between BBP exposure and MDI (b= -0.1, 95%CI: -0.8, 0.5); 6 studies, 2119 participants).	No association between BBP exposure and MDI in:<br>Girls<br>β −0.7, 95%CI: −1.6 to 0.2; studies and participants unspecified<br>Boys<br>β 0.8, 95%CI: −0.3 to 1.9; studies and participants unspecified	

Study details	Appraisal details	Participants	Plastic exposure	Health outcomes	Findings	Subgroup Findings	AMSTAR score
			Unspecified				
			**Type**<br>butyl benzyl phthalate (BBP)<br><br>**Route**<br>Unspecified, but exposure level measured in urine or plasma<br>Varies between included study<br><br>**Measure**<br>ng/mL<br>µg/g<br><br>**Exposure time**<br>Unspecified	Mental Development Index (MDI)<br>Beta coefficient effect size (natural units of the Bayley Scales of Infant Development)	No association between BBP exposure and MDI (b= -0.1, 95%CI: -0.8, 0.5); 6 studies, 2119 participants).	No association between BBP exposure and MDI in:<br>Girls<br>β −0.7, 95%CI: −1.6 to 0.2; studies and participants unspecified<br>Boys<br>β 0.8, 95%CI: −0.3 to 1.9; studies and participants unspecified	
			**Type**<br>butyl benzyl phthalate (BBP)<br><br>**Route**<br>Unspecified, but exposure level measured in urine or plasma<br>Varies between included study<br><br>**Measure**<br>ng/mL<br>µg/g<br><br>**Exposure time**<br>Unspecified	Psychomotor Development Index (PDI)<br>Beta coefficient effect size (natural units (natural units of the Bayley Scales of Infant Development)	No association between BBP exposure and PDI (b= -0.7, 95%CI: -1.4, 0.0); 6 studies, 2119 participants).	There is an association between BBP exposure and PDI in girls<br>β −1.6, 95%CI: −2.6 to −0.6; studies and participants unspecified<br>No association between BBP exposure and MDI in boys<br>β 0.8, 95%CI: −0.2 to 1.9; studies and participants unspecified	
			**Type**<br>dibutyl phthalate (DBP)<br><br>**Route**<br>Unspecified, but exposure level measured in urine or plasma	Mental Development Index (MDI)<br>Beta coefficient effect size (natural units of	No association between DBP exposure and MDI (b= -0.2, 95%CI: -0.7, 0.4); 7 studies, 2536 participants).	No association between DBP exposure and MDI in:<br>Girls<br>β −0.8, 95%CI: −2.2 to 0.6; studies and participants unspecified	

Study details	Appraisal details	Participants	Plastic exposure	Health outcomes	Findings	Subgroup Findings	AMSTAR score
			Varies between included study<br><br>**Measure**<br>ng/mL<br>µg/g<br><br>**Exposure time**<br>Unspecified	the Bayley Scales of Infant Development)		Boys<br>β 0.4, 95%CI: −0.8 to 1.6; studies and participants unspecified	
			**Type**<br>dibutyl phthalate (DBP)<br><br>**Route**<br>Unspecified, but exposure level measured in urine or plasma<br>Varies between included study<br><br>**Measure**<br>ng/mL<br>µg/g<br><br>**Exposure time**<br>Unspecified	Psychomotor Development Index (PDI)<br>Beta coefficient effect size (natural units (natural units of the Bayley Scales of Infant Development)	No association between DBP exposure and PDI (b= -0.5, 95%CI: -1.5, 0.5); 6 studies, 2119 participants).	No association between DBP exposure and PDI in:<br>Girls<br>β −0.7, 95%CI: −1.8 to 0.3; studies and participants unspecified<br>Boys<br>β 0.0, 95%CI: −1.7 to 1.8; studies and participants unspecified	
			**Type**<br>Diethyl phthalate<br>(DEP)<br><br>**Route**<br>Unspecified, but exposure level measured in urine or plasma<br>Varies between included study<br><br>**Measure**<br>ng/mL<br>µg/g<br><br>**Exposure time**	Mental Development Index (MDI)<br>Beta coefficient effect size (natural units of the Bayley Scales of Infant Development)	No association between DEP exposure and MDI (b=0.3, 95%CI: -0.3, 0.9); 5 studies, 1791 participants).	No association between DEP exposure and MDI in:<br>Girls<br>β 0.3, 95%CI: −0.8 to 1.4; studies and participants unspecified<br>Boys<br>β 0.0, 95%CI: −1.1 to 1.2; studies and participants unspecified	

Study details	Appraisal details	Participants	Plastic exposure	Health outcomes	Findings	Subgroup Findings	AMSTAR score
			Unspecified				
			**Type**<br>Diethyl phthalate<br>(DEP)<br><br>**Route**<br>Unspecified, but exposure level measured in urine or plasma<br>Varies between included study<br><br>**Measure**<br>ng/mL<br>µg/g<br><br>**Exposure time**<br>Unspecified	Psychomotor Development Index (PDI)<br>Beta coefficient effect size (natural units (natural units of the Bayley Scales of Infant Development)	No association between DEP exposure and PDI (b= 0.0, 95%CI: -0.6, 0.6); 4 studies, 1361 participants).	No association between DEP exposure and PDI in:<br>Girls<br>β 0.4, 95%CI: −0.5 to 1.4; studies and participants unspecified<br>Boys<br>β 0.4, 95%CI: −0.5 to 1.4; studies and participants unspecified	
			**Type**<br>diisobutyl phthalate (DIBP)<br><br>**Route**<br>Unspecified, but exposure level measured in urine or plasma<br>Varies between included study<br><br>**Measure**<br>ng/mL<br>µg/g<br><br>**Exposure time**<br>Unspecified	Mental Development Index (MDI)<br>Beta coefficient effect size (natural units of the Bayley Scales of Infant Development)	No association between DIBP exposure and MDI (b= -0.1, 95%CI: -0.6, 0.4); 4 studies, 1361 participants).	No association between DIBP exposure and MDI in:<br>Girls<br>β −0.8, 95%CI: −2.1 to 0.6; studies and participants unspecified<br>Boys<br>β 0.8, 95%CI: −0.3 to 1.8; studies and participants unspecified	
			**Type**<br>diisobutyl phthalate (DIBP)<br><br>**Route**	Psychomotor Development Index (PDI)<br>Beta coefficient effect size	No association between DIBP exposure and PDI (b= -0.4, 95%CI: -1.1, 0.3); 5 studies, 1689 participants).	No association between DIBP exposure and PDI in:<br>Girls	

Study details	Appraisal details	Participants	Plastic exposure	Health outcomes	Findings	Subgroup Findings	AMSTAR score
			Unspecified, but exposure level measured in urine or plasma<br>Varies between included study<br><br>**Measure**<br>ng/mL<br>µg/g<br><br>**Exposure time**<br>Unspecified	(natural units (natural units of the Bayley Scales of Infant Development)		β −0.5, 95%CI: −1.9 to 0.9; studies and participants unspecified<br>Boys<br>β −0.1, 95%CI: −1.4 to 1.2; studies and participants unspecified	
**Forns et al., 2020 [77] - Early Life Exposure to Perfluoroalkyl Substances (PFAS) and ADHD: A Meta-Analysis of Nine European Population-Based Studies. No COI declared.**							
**Last search**<br>Unspecified<br><br>**Study types**<br>European cohort<br><br>**Included studies in the review** = 9<br><br>**Included studies in the meta-analysis** = 9	unspecified	Children (≤18 years old) and mothers (maternal-exposure)<br><br>N = 4826 mother-child pairs	**Type**<br>Perfluoroalkyl substances (PFAS)- perfluorooctane sulfonate (PFOS) and perfluorooctanoic acid (PFOA)<br><br>**Route**<br>Prenatal exposure, unless exposure measured in breast milk<br><br>**Measure**<br>Maternal serum/plasma or breast milk (ng/mL)<br><br>**Exposure time**<br>First 24 months of life	Attention deficit and hyperactivity disorder (ADHD) children 4-11 years old<br>OR and 95%CIs; logistic regression models		No associations between PFOA and ADHD in various time periods based on the model used: at birth (OR, 1.01, 95%CI: 0.93 to 1.11; 9 cohorts, 4,826 participants), 3 months (OR 1.02, 95%CI: 0.93 to 1.11; 9 cohorts, 4,826 participants) , 6 months (OR 1.01, 95%CI: 0.91 to 1.12; 9 cohorts, 4,826 participants), 12 months (OR 1.00, 95%CI: 0.89 to 1.12;9 cohorts, 4,826 participants) and 24 months (OR 0.99, 95%CI: 0.88 to 1.12;9 cohorts, 4,826 participants)<br><br>No associations between PFOS and ADHD in various time periods based on the model used: at birth (OR 0.99, 95%CI: 0.92 to 1.07; 9 cohorts; 4,826 participants), 3 months (OR 0.99, 95%CI:0.92 to 1.06; 9 cohorts; 4,826 participants), 6 months (OR 0.98, 95%CI:0.90 to 1.06; 9 cohorts; 4,826 participants), 12 months (OR 0.96, 95%CI: 0.87 to 1.06; 9 cohorts; 4,826 participants)	**3**

Study details	Appraisal details	Participants	Plastic exposure	Health outcomes	Findings	Subgroup Findings	AMSTAR score
						and 24 months (OR 0.97, 95%CI: 0.88 to 1.07; 9 cohorts; 4,826 participants)	
						An association between PFOA and ADHD in girls was found at birth (OR 1.28, 95%CI: 1.03 to 1.59; 9 cohorts, 82 participants) and at 3 months (OR, 1.28, 95%CI: 1.01 to 1.62; 9 cohorts, 82 participants). No association was found at 6 months (OR 1.29, 95%CI: 1.00 to 1.66; 9 cohorts, 82 participants), 12 months (OR 1.24, 95%CI: 0.96 to 1.61; 9 cohorts, 82 participants), 24 months (OR 1.30, 95%CI: 0.98 to 1.73; 9 cohorts, 82 participants). No association between PFOA and ADHD in boys in various time periods: at birth (OR 0.98, 95%CI:0.87 to 1.09; 9 cohorts, 306 participants), 3 months (OR, 1.00, 95%CI:0.89 to 1.11; 9 cohorts, 306 participants), 6 months (OR, 1.02, 95%CI: 0.86 to 1.22; 9 cohorts, 306 participants), 12 months (OR 1.03, 95%CI: 0.85 to 1.25; 9 cohorts, 306 participants) and 24 months (OR 0.97, 95%CI: 0.83 to 1.14; 9 cohorts, 306 participants)	
						No associations between PFOS and ADHD in various time periods based on the model used in subgroup of girls: at birth (OR 1.14, 95%CI: 0.91 to 1.34; 9 cohorts, 82 participants), 3 months (OR 1.12, 95%CI :0.94 to 1.34; 9 cohorts, 82 participants), 6	

Study details	Appraisal details	Participants	Plastic exposure	Health outcomes	Findings	Subgroup Findings	AMSTAR score
						months (OR 1.13 95%CI: 0.93 to 1.36; 9 cohorts, 82 participants), 12 months (OR 1.19, 95%CI: 0.92 to 1.53; 9 cohorts, 82 participants), 24 months (OR 1.26, 95%CI: 0.93 to 1.72; 9 cohorts, 82 participants)<br>No associations between PFOS and ADHD in various time periods based on the model used in subgroup of boys: at birth (OR 0.96, 95%CI: 0.88 to 1.05; 9 cohorts, 306 participants), 3 months (OR 0.96 , 95%CI: 0.89 to 1.05; 9 cohorts, 306 participants), 6 months (OR 0.95 , 95%CI: 0.86 to 1.05; 9 cohorts, 306 participants), 12 months (OR 0.93, 95%CI: 0.83 to 1.04; 9 cohorts, 306 participants), 24 months (OR 0.92, 95%CI: 0.81 to 1.03; 9 cohorts, 306 participants)	

2.6 Nutritional outcomes

Study details	Appraisal details	Participants	Plastic exposure	Health outcomes	Findings	Subgroup Findings	AMSTAR score
**Golestanzadeh et al., 2019 [54] - Association of exposure to phthalates with cardiometabolic risk factors in children and adolescents: a systematic review and meta-analysis. No COI declared.**							
**Last search**<br>Dec-18<br><br>**Study types**<br>cohort (n=17)<br>cross sectional (n=15)<br>case-control (n=3)<br><br>**Included studies in the review** = 35<br><br>**Included studies in the meta-analysis** = 23	STROBE checklist. ****Note this is a reporting guideline not a critical appraisal tool. ****The quality of the included observational studies was good and most of them gained a high score.	Children (≤ 18 years)<br><br>N = 24,943	**Type**<br>Phthalates<br><br>**Route**<br>Unspecified<br><br>**Measure**<br>Urine and serum (units unspecified)<br><br>**Exposure time**<br>Prenatal	BMI<br>Beta coefficient and 95% CI		For LMWP:<br>Exposure to MiBP was positively associated with BMI (z= 0.18, 95%CI: 0.002, 0.35; 3 studies, 950 participants) but not for MMP (z=0.09, 95%CI:−0.08, 0.26; 6 studies, 1695 participants), MEP (z= 0.19, 95%CI:− 0.09, 0.46; 6 studies, 2545 participants),<br>For HMWP: Exposure to MEHHP was positively associated with BMI (z= 0.18, 95%CI: 0.04, 0.31; 9 studies, 2490 participants) but not for MEHP (z= 0.15,95%CI: − 0.10 0.39; 9 studies, 3195 participants), MEOHP (z= − 0.001, 95%CI:− 0.09, 0.09; 9 studies, 2490 participants), MBzP (z= 0.17,95%CI: − 0.09, 0.43; 3 studies, 905 participants), MCOP (z= 0.05,95%CI: − 0.06, 0.17; 1 study, 276 participants), MCPP (z 0.15, 95%CI:− 0.10 0.41; 2 studies, 663 participants), and MECPP (z= − 0.12,95%CI: − 0.27, 0.03; 4 studies, 1059 participants).	5
				BMI z-score<br>Beta coefficient and 95% CI		Exposure to LMWP metabolites and BMI z-score was positively associated in all subgroups; Monobutylphthalate (MBP) (z=0.11, 95%CI: 0.06, 0.16; 5 studies, 3991 participants), monoethylphthalate (MEP) (z=0.11, 95%CI: 0.05, 0.17, 4 studies, 3774 participants),	

Study details	Appraisal details	Participants	Plastic exposure	Health outcomes	Findings	Subgroup Findings	AMSTAR score
						monoisobutylphthalate (MiBP) (z=0.11, 95%CI: 0.08, 0.17, 4 studies, 3774 participants). Exposure to HMWP metabolites was associated with a higher BMI z-score; monobenzylphthalate (MBzP) (z=0.09, 95%CI: 0.01, 0.16; 4 studies, 1154 participants), mono(3-carboxyprpoyl)phthalate (MCPP) (z=0.06, 95%CI: 0.01, 0.11; 3 studies, 912 participants), monocarboxyoctylphthalate (MCOP) (z=0.11, 95%CI: 0.06, 0.16; 1 study, 345 participants) and di-2-ethylhexylphthalate (DEHP subgroups) (z= 0.07, 95%CI: 0.01, 0.13; 4 studies, 3774 participants), but not for MEHP (z= 0.16, 95%CI:− 0.06, 0.15; 2 studies, 629 participants), MEHHP (z= 0.20, 95%CI:− 0.08, 0.48; 2 studies, 629 participants), MEOHP (z= 0.12, 95%CI: − 0.02, 0.26; 2 studies, 629 participants).	
				Waist circumference Beta coefficient and 95% CI		For LMWP: No associations were found for MMP (z= 0.06, 95%CI: − 0.06, 0.18; 3 studies, 777 participants), MEP (z= 0.17, 95%CI: − 0.18, 0.52; 3 studies, 922 participants), MBP (z= 0.19, 95%CI: − 0.19 0.58; 4 studies, 1043 participants) and MiBP (z= − 0.33, 95%CI: − 1.11, 0.45; 2 studies, 646 participants). Exposure to the HMWP metabolites was associated with waist	

Study details	Appraisal details	Participants	Plastic exposure	Health outcomes	Findings	Subgroup Findings	AMSTAR score
						circumference; mono(2-ethylhexyl)phthalate (MEHP) (z=0.13, 95%CI: 0.04, 0.21; 5 studies, 1301 participants), mono(2-ethyl-5-hydroxyhexyl)phthalate (MEHHP) (z=0.28, 95%CI: 0.09, 0.47, 5 studies, 1301 participants) and monobenzylphthalate (MBzP) (z=0.12, 95%CI: 0.02, 0.22, 3 studies, 905 participants) but not for MEOHP (z=0.05, 95%CI:− 0.02, 0.13; 5 studies, 1301 participants), MCOP (z=0.02, 95%CI:− 0.10, 0.14; 1 study, 276 participants), MCPP (z= − 0.46, 95%CI: − 1.42, 0.51; 2 studies, 663 participants), and MECPP (z= − 0.11, 95%CI: − 0.24, 0.03: 4 studies, 1059 participants)	
**Ribeiro et al., 2019 [81] - Association between the exposure to phthalates and adiposity: A meta-analysis in children and adults. No COIs reported (Funding body acknowledged)**							
**Last search**<br>Aug-19<br><br>**Study types**<br>Cross-sectional (n=25)<br>Prospective cohort study (n=1)<br>Case control (n=3)<br><br>**Included studies in the review** = 29<br><br>**Included studies in the meta-analysis** = 8	The STROBE checklist was used to evaluate the methodological quality of the included studies. In children the paper with the lowest quality level was Shoaff with 14.8/22points, but for adults we found 3 papers with low quality: Peck (11.7/22), Milosevic (12.7/22) and Stojanoska (12.9/22). However, since all are above 11, it was considered as acceptable methodological quality, and they were eligible for the systematic review and meta-analysis. The three papers with the lowest quality score	Children and adults, no apparent age limiters used.<br>Adults (n=16075)<br>Children (n=10893)<br><br>N = 26,968	**Type**<br>Mono-butyl phthalate (MBP)<br><br>**Route**<br>Unspecified<br><br>**Measure**<br>Unspecified only regression coefficients reported<br><br>**Exposure time**<br>Combined post and pre-natal exposure	BMI<br>Beta-coefficient effect size (transformed z-score)	No association between exposure to MBP and BMI **in children** (b= 0.00, 95%CI: -0.11, 0.12; 3 studies, 820 participants).	Children and adult data were split from the outset and no test for subgroup differences was performed. There was no meta-analysis performed on this outcome in the adult population<br>In general, studies presented positive associations, both in children and adults, however most of the studies did not reach statistical significance. Meta-analyses were performed for seven compounds, seven for BMI, five for WC and four for obesity (categorical BMI). Regarding adults, summary estimates indicate a	6

Study details	Appraisal details	Participants	Plastic exposure	Health outcomes	Findings	Subgroup Findings	AMSTAR score
	were included in meta-analysis but they had the lowest weight in the overall estimate.					negative association for MEHP and a positive association for MEP and MECPP but only for MECPP statistical significance was reached. In children, overall estimates were null for MBP; negative for MiBP, MbzP and MCPP and positive for MEP but none showed a statistically significant association. The inconsistency in the results and the fact that most of them reported associations that were not statistically significantly require some putative explanations, such as: 1) the study design and the short-half-life of phthalates, 2) the lipophilic capacity of phthalates, 3) gender and age differences.	
				Waist Circumference Beta-coefficient effect size (cm)	No association between exposure to MBP and waist circumference **in children** (b=0.13cm, 95%CI: -0.86, 1.13; 3 studies, 820 participants).	Children and adult data were split from the outset and no test for subgroup differences was performed. There was no meta-analysis performed on this outcome in the adult population	
			**Type**<br>Mono-(2-ethylhexyl) phthalate (MEHP)<br><br>**Route**<br>Unspecified<br><br>**Measure**<br>Unspecified only regression coefficients reported	BMI Beta-coefficient effect size (kg/m2)	No association between exposure to MEHP and BMI **in adults** (b= -0.05kg/m2, 95%CI: -0.15, 0.05; 3 studies, 1298 participants).	Children and adult data were split from the outset and no test for subgroup differences was performed. There was no meta-analysis performed on this outcome in the child population	
				Obesity (categorised using BMI)	No association between exposure to MEHP and obesity **in adults** (OR = 0.91, 95%CI: 0.66, 1.27;	Children and adult data were split from the outset and no test for subgroup differences was performed.	

Study details	Appraisal details	Participants	Plastic exposure	Health outcomes	Findings	Subgroup Findings	AMSTAR score
			**Exposure time**<br>Combined post and pre-natal exposure	OR and (95% CI)<br>No logistic regression	3 studies, 2432+ participants).		
				Waist Circumference<br>Beta-coefficient effect size (cm)	Positive association between exposure to MEHP and waist circumference (b= 0.58cm, 95%CI: 0.55, 0.62cm; 3 studies,2435 participants).	Children and adult data were split from the outset and no test for subgroup differences was performed. There was no meta-analysis performed on this outcome in the child population	
				Obesity (categorised using BMI)<br>OR and (95% CI)<br>No logistic regression	No association between exposure to MEHP and obesity **in children** (OR = 0.78, 95%CI: 0.47, 1.29; 3 studies, 773+ participants).	Children and adult data were split from the outset and no test for subgroup differences was performed.	
			**Type**<br>Mono-ethyl phthalate (MEP)<br><br>**Route**<br>Unspecified<br><br>**Measure**<br>Unspecified only regression coefficients reported<br><br>**Exposure time**<br>Combined post and pre-natal exposure	BMI<br>Beta-coefficient effect size (kg/m2)	No association between exposure to MEP and BMI **in adults** (b=0.05kg/m2, 95%CI: -0.06, 0.16; 4 studies, 512 participants).	Children and adult data were split from the outset and no test for subgroup differences was performed. There was no meta-analysis performed on this outcome in the child population	
				Obesity (categorised using BMI)<br>OR and (95% CI)<br>No logistic regression	No association between exposure to MEP and Obesity **in adults** (OR = 1.22, 95%CI: 0.94, 1.5; 4 studies, 3701+ participants).	Children and adult data were split from the outset and no test for subgroup differences was performed. There was no meta-analysis performed on this outcome in the child population	
				BMI<br>Beta-coefficient effect size (transformed z-score)	No association between exposure to MEP and BMI score **in children** (b= 0.02, 95%CI: -0.06, 0.10; 3 studies, 820 participants).	Children and adult data were split from the outset and no test for subgroup differences was performed. There was no meta-analysis performed on this outcome in the adult population	

Study details	Appraisal details	Participants	Plastic exposure	Health outcomes	Findings	Subgroup Findings	AMSTAR score
				Waist Circumference Beta-coefficient effect size (cm)	No association between exposure to MEP and waist circumference **in children** (b= 0.47cm, 95%CI: -0.23, 1.17; 3 studies, 820 participants).	Children and adult data were split from the outset and no test for subgroup differences was performed. There was no meta-analysis performed on this outcome in the adult population	
			**Type**<br>Mono-iso-butyl phthalate (MiBP)<br><br>**Route**<br>Unspecified<br><br>**Measure**<br>Unspecified only regression coefficients reported<br><br>**Exposure time**<br>Combined post and pre-natal exposure	BMI Beta-coefficient effect size (transformed z-score)	No association between exposure to MiBP and BMI **in children** (b= -0.01, 95%CI: -0.10, 0.07; 3 studies, 820 participants).	Children and adult data were split from the outset and no test for subgroup differences was performed. There was no meta-analysis performed on this outcome in the adult population	
				Waist Circumference Beta-coefficient effect size (cm)	No association between exposure to MiBP and Waist Circumference **in children** (b=-0.62cm, 95%CI: -1.6, 0.37; 3 studies, 820 participants).	Children and adult data were split from the outset and no test for subgroup differences was performed. There was no meta-analysis performed on this outcome in the adult population	
			**Type**<br>Mono-benzyl phthalate (MBzP)<br><br>**Route**<br>Unspecified<br><br>**Measure**<br>Unspecified only regression coefficients reported<br><br>**Exposure time**<br>Combined post and pre-natal exposure	BMI Beta-coefficient effect size (transformed z-score)	No association between MBzP and BMI **in children** (b=-0.06, 95%CI: -0.15, 0.04; 3 studies, 820 participants).	Children and adult data were split from the outset and no test for subgroup differences was performed. There was no meta-analysis performed on this outcome in the adult population	
				Waist Circumference Beta-coefficient effect size (cm)	No association between MBzP and Waist circumference **in children** (b=-0.35cm, 95%CI: -1.16, 0.48cm; 3 studies, 820 participants).	Children and adult data were split from the outset and no test for subgroup differences was performed. There was no meta-analysis performed on this outcome in the adult population	
				BMI	No association between MCPP and BMI **in**	Children and adult data were split from the outset and no test for	

Study details	Appraisal details	Participants	Plastic exposure	Health outcomes	Findings	Subgroup Findings	AMSTAR score
			**Type**<br>Mono-(3-carboxypropyl) Phthalate (MCPP)<br>**Route**<br>Unspecified<br><br>**Measure**<br>Unspecified only regression coefficients reported<br><br>**Exposure time**<br>Combined post and pre-natal exposure	Beta-coefficient effect size (transformed z-score)	**children** (b=-0.12, 95%CI: -0.24, 0; 3 studies, 820 participants).	subgroup differences was performed. There was no meta-analysis performed on this outcome in the adult population	
				Waist Circumference Beta-coefficient effect size (cm)	No association between MCPP and waist circumference **in children** (b=-0.73, 95%CI -1.74, 0.28; 3 studies, 820 participants).	Children and adult data were split from the outset and no test for subgroup differences was performed. There was no meta-analysis performed on this outcome in the adult population	
			**Type**<br>Mono(2-ethyl-5-carboxypentyl) phthalate (MECPP)<br><br>**Route**<br>Unspecified<br><br>**Measure**<br>Unspecified only regression coefficients reported<br><br>**Exposure time**<br>Combined post and pre-natal exposure	Obesity (categorised using BMI) OR and (95% CI) No logistic regression	Positive association between exposure to MECPP and odds of obesity in adults (OR = 1.67, 95%CI: 1.3,2.16; 3 studies, 3599+ participants)	Children and adult data were split from the outset and no test for subgroup differences was performed. There was no meta-analysis performed on this outcome in the children population	
**Liu et al., 2018 [82] - Perfluorooctanoic Acid (PFOA) Exposure in Early Life Increases Risk of Childhood Adiposity: A Meta-Analysis of Prospective Cohort Studies. No COIs declared**							
**Last search**<br>May-18<br><br>**Study types**<br>Prospective<br>Cohort<br><br>**Included studies in the review** = 10	Newcastle–Ottawa scale. All studies scored between 7-8 and were therefore deemed as high quality.	Unclear in the characteristics of the included participants. Combined males and females, but age not noted.<br><br>N = 6,077	PFOA<br>Unspecified<br>maternal serum/ plasma or cord blood<br>Measured through pregnancy and up to 3 weeks postpartum. However unspecified on when the PFOA entered blood stream of mother	Childhood Obesity<br>Effect size (RR and OR) 95% CI. There was no clear definition in the research about what was	PFOA in early life had a statistically significant association with childhood overweight risk (1.25, 95% CI: 1.04, 1.50, 8 studies, participants unspecified).***<br>Authors reported an	When the studies stratified analysis by the effect size (relative risk vs. odds ratio), a significant correlation between early-life exposure to PFOA and childhood overweight risk was observed in group of relative risk (RR) (RR = 1.26, 95% CI: 1.01, 1.56; 6 studies, 4224 participants) while the odds ratio	7

Study details	Appraisal details	Participants	Plastic exposure	Health outcomes	Findings	Subgroup Findings	AMSTAR score
**Included studies in the meta-analysis** = 10				considered obesity	effect size that was RR and OR combined.	(OR) group had a slightly higher assessment but no significant risk for childhood overweight (OR = 1.39, 95% CI: 0.85, 2.28; 2 studies, 1223 participants).	
				Childhood increased BMI Beta-coefficients; 95% CI. There was no clear definition in the research about what was considered high BMI	Exposure to PFOA in early life could slightly increase the z-score of childhood BMI (β = 0.10, 95% CI: 0.03,.17, 9 studies, 5,411 participants); Sensitivity analysis β 0.07, 95% CI: 0.01 to 0.14; 5 studies, 3,825 participants	Studies were stratified by measurement timing: Prenatal exposure β= 95%CI: 0.09 0.02, 0.17, studies unspecified, 5505participants and Postnatal exposure β=0.16 95%CI: 0.01, 0.30, studies unspecified, 571 participants to PFOA indicated a small increase in the z-score of childhood BMI.<br><br>Early-life exposure to PFOA and childhood BMI z-score a significant association was observed among the studies performed in Europe β=0.10 95%CI: 0.02, 0.17, 7 studies, 3545 participants<br><br>No association between Early-life exposure to PFOA and childhood BMI z-score among the studies performed in Northern America β=0.19 95%CI: −0.05, 0.42, 3 studies, 2102 participants<br><br>Early-life exposure to PFOA and childhood BMI z-score a significant association was observed in the group adjusted by adjusted by maternal parity β= 0.13 95%CI: 0.02, 0.24, 7studies, 3949	

Study details	Appraisal details	Participants	Plastic exposure	Health outcomes	Findings	Subgroup Findings	AMSTAR score
						participants.<br>However, no association between PFOA and when maternal parity was not adjusted β= 0.07 95%CI: −0.01, 0.15, 4 studies, 2127 participants.<br><br>The subgroup of birth weight was evaluated, and PFOA exposure could statistically significantly increase the z-score of childhood BMI in the group that was not adjusted by birth weight β= 0.10 95%CI: 0.03, 0.17, 10 studies, 5705 participants.<br><br>There was no association between PFOA and BMI z-score in girls β=0.0695%CI: −0.01, 0.13, studies unspecified, 1549 participants and in boys β=−0.01 95%CI:−0.10, 0.08, studies unspecified, 1628 participants.	
**Rancière et al., 2015 [66] - Bisphenol A and the risk of cardiometabolic disorders: a systematic review with metaanalysis of the epidemiological evidence. No DOI declared**							
**Last search**<br>Aug-14<br><br>**Study types**<br>Cross-sectional (n=28) and Prospective Longitudinal (n=5) (pooled together)	A scoring system based on the established OHAT guidelines [31] adapted to reflect the characteristics of the included studies: longitudinal design (2 points), population-based study (1 point), outcome assessment including measurements (1 point), collection of at	Adults or children (however only diabetes in adults, in an attempt to limit the analysis to type 2). Pregnant women were excluded.	**Type**<br>Bisphenol A<br><br>**Route**<br>Non-specific<br><br>**Measure**<br>Urinary BPA and amniotic fluid (μg/L). Compared with extreme categories of	Overweight<br>OR with 95% CI	No association between BPA and prevalent overweight in children and adults 1.21 95% CI:0.98,1.50, 7 studies, no participant data recorded)	No association between BPA and prevalent overweight in children 1.24 (95% CI:.88, 1.75,5 studies, no participant data reported).<br>Exposure to BPA was positively associated with prevalent overweight in adults 1.25 (95% CI:0.98,1.5,2 studies, no participant data reported)	7

Study details	Appraisal details	Participants	Plastic exposure	Health outcomes	Findings	Subgroup Findings	AMSTAR score
**Included studies in the review** = 33<br><br>**Included studies in the meta-analysis** = 12	least 2 urine samples per participant (1 point), control for urine dilution (1 point), adjustment for dietary intake (1 point), and adjustment for socioeconomic variables (1 point). Studies were then classified as 'low quality' (total score between 0 and 2), 'medium quality' (total score between 3 and 5), or 'high quality' (total score between 6 and 8). Studies ranged from Low, medium to High. No discussion on the implications of quality on findings.	N = 69,486	urinary BPA levels (the highest vs. the lowest). Highest levels found a mean (SE) of 5.0 (0.3) ng/mL in boys and 4.6 (0.3) ng/mL in girls. Lowest: a median (interquartile range, IQR) of 0.60 (0.20–1.37) ng/mL.<br><br>**Exposure time**<br>non-specific	Obesity<br>OR with 95% CI	Exposure to BPA was positively associated with prevalent obesity in children and adults 1.67 (95% CI:1.41,1.98, 3 studies, no participant data reported).	Exposure to BPA was positively associated with prevalent obesity in children 2.05(95% CI:.1.38,3.04, 1 study, no participant data reported).<br>Exposure to BPA was positively associated with prevalent obesity in adults 1.60 (95% CI:1.32, 1.93, 2 studies, no participant data reported)	
				Elevated Waist Circumference<br>OR with 95% CI	Exposure to BPA was positively associated with prevalent waist circumference in children and adults 1.48(95% CI:1.25,1.76, 4 studies, no participant data reported)	No association between BPA and prevalent elevated waist circumference in children 1.4 (95% CI:0.91,2.15, 1 study, no participant data reported).<br>Exposure to BPA was positively associated with prevalent waist circumference in adults 1.52 (1.21,1.90, 3 studies, no participant data reported).	
**Ribeiro et al., 2020 [80] - Exposure to endocrine-disrupting chemicals and anthropometric measures of obesity: a systematic review and meta-analysis. No COIs declared**							
**Last search**<br>Jun-18<br><br>**Study types**<br>Cross-sectional<br><br>**Included studies in the review** = 9<br><br>**Included studies in the meta-analysis** = 6	Newcastle-Ottawa Scale. Quality assessment using the Newcastle-Ottawa Scale indicated that 65% of cross-sectional studies and all prospective studies (note- these were not included in the MA) had a low or medium risk of bias.	Adults and children from the general population, from 6-74 years<br><br>N = 23,214	**Type**<br>BPA<br><br>**Route**<br>Unspecified<br><br>**Measure**<br>Urinary [BPA] (units not specified)<br><br>**Exposure time**<br>Unspecified	Prevalent Elevated Waist Circumference (Methods of categorisation unspecified)<br>OR and 95%CI no logistic regression	Positive association between BPA exposure and prevalence of an elevated waist circumference (OR=1.49, 95%CI: 1.29, 1.72; 6 studies; 10005 participants).	When subgrouped based on age, there was no association between BPA exposure and prevalence of an elevated waist circumference in children (OR=1.62, 95%CI: 0.97, 2.72; 3 studies; 3836 participants). There was an association between BPA exposure and an elevated waist circumference in adults (OR=1.25, 95%CI: 1.27, 1.78; 2 studies, 6137 participants).	7
				Prevalent Overweight (as	Positive association between BPA exposure and prevalence of being	When subgrouped based on age, there was no association between BPA exposure and prevalence of	

Study details	Appraisal details	Participants	Plastic exposure	Health outcomes	Findings	Subgroup Findings	AMSTAR score
				classified by BMI)<br>OR and 95%CI<br>no logistic regression	overweight (OR=1.32, 95%CI: 1.01, 1.72; 5 studies, 11339 participants).	being overweight in children (OR=1.67, 95%CI: 0.82, 3.38; 3 studies; 5202 participants).<br>There was an association between BPA exposure and prevalence of being overweight in adults (OR=1.25, 95%CI: 1.01, 1.56; 2 studies, 6137 participants).	
				Prevalent Obese (as classified by BMI)<br>OR and 95%CI<br>no logistic regression	Positive association between BPA exposure and prevalence of obesity (OR=1.57, 95%CI: 1.35, 1.83; 5 studies, 12749 participants)	Weh subgrouped based on age, there were still positive association between BPA exposure for both children (OR=2.05, 95%CI 1.38, 3.04; 1 study, 3370 participants) and adults (OR=1.50, 95%CI: 1.35, 1.83; 4 studies, 9379 participants).	
**Kim et al., 2019 [78] - The association between Bisphenol A exposure and obesity in children - A systematic review with meta-analysis. No COI declared**							
**Last search**<br>Oct-17<br><br>**Study types**<br>Cross-sectional (n=8); Case-control (n=2); Observational (n=3)<br><br>**Included studies in the review =**<br>13<br><br>**Included studies in the meta-analysis =** 13	Newcastle-Ottawa quality assessment scale (NOS) - score of 9 (n=7); score of 8 (n=2); score of 7 (n=3); score of 6 (n=1)	Age: 14 months to 19 years<br><br>N = 11,303	**Type**<br>BPA<br><br>**Route**<br>post-natal<br><br>**Measure**<br>Urinary - µg/L<br><br>**Exposure time**<br>NR	Obesity<br>OR and 95%CI (Unspecified how measured in primary studies)	Exposure to BPA was associated with an increased odd of obesity (BMI) when compared to low exposure (reference group) (1.57; 95%CI 1.10,2.23; 7 studies, 9,602 participants)	Relatively high exposed group<br>OR 1.58, 95%CI: 1.077 to 2.315; 6 studies; 9,522 participants	6
				Obesity<br>SMD and 95%CI	No association found between urinary BPA levels and being obese compared to normal weight (SMD=0.166, 95%CI: -0.121,0.453; 8 studies, 2,092 participants)	Obese vs normal weight children (excluding pilot studies)<br>SMD 0.044, 95%CI: -0.088 to 0.176; 6 studies, 1,962 participants	
**Wu et al., 2020 [79] - Bisphenol A and the Risk of Obesity a Systematic Review with Meta-Analysis of the Epidemiological Evidence. No COI declared**							

Study details	Appraisal details	Participants	Plastic exposure	Health outcomes	Findings	Subgroup Findings	AMSTAR score
**Last search**<br>01-Jan-20<br><br>**Study types**<br>Cross-sectional (n=6)<br>Survey (n=3)<br>Cohort (n=1)<br><br>**Included studies in the review =** 10<br><br>**Included studies in the meta-analysis =** 10	Newcastle-Ottawa Scale (0-9)<br>Quality score of all 10 publications ranged from 6 to 8, with a median score of 7.	Children, adults and elderly (range, 6-79 years)<br><br>N = 27,993	**Type**<br>Bisphenol A (BPA)<br><br>**Route**<br>NR<br><br>**Measure**<br>Urinary BPA (ng/mL or µg/L)<br><br>**Exposure time**<br>NR	Obesity<br>obesity risk reported using odds ratios (ORs), hazard ratios (HRs), or relative risk ratios (RRs) with the corresponding 95% confidence interval for the highest level versus lowest level of BPA exposure	Overall values for obesity not extracted due to duplicate counting of data		5
				Abdominal obesity	Exposure to BPA was associated with an increase odds in developing abdominal obesity across all populations (OR:1.43, 95% CI: 1.27-1.62, I2=0%, P= 0.651, 7 studies, 21,629 participants)	A dose–response analysis revealed that 1-ng/mL increase in BPA increased the risk of abdominal obesity by 12% (OR: 1.12, 95% CI: 1.09-1.14, p value for a linear trend test <.001). No evidence of non-linear association was found between BPA and abdominal obesity risk.	
				Generalised obesity	Exposure to BPA was associated with an increase odds in developing generalised obesity across all populations (OR: 1.83, 95% CI: 1.58-2.12, I2=16.7%, P= 0.299. 8 studies, 25,779 participants)	A dose–response analysis analysis revealed that a 1-ng/mL increase in BPA corresponded to a 16% increase in the risk of generalized obesity (OR: 1.16, 95%CI: 1.14-1.19, P value for a linear trend test <.001). Evidence of non-linear association was found between BPA and generalised obesity risk	

Study details	Appraisal details	Participants	Plastic exposure	Health outcomes	Findings	Subgroup Findings	AMSTAR score
						(P=024).	
				Generalised overweight	Exposure to BPA was associated with an increase odds in developing a generalised overweight condition across all populations (OR: 1.24, 95% CI: 1.02-1.51, I2=31.7%, P=0.198, 6 studies, 18,404 participants).	A dose–response analysis analysis revealed that a 1-ng/mL increase in BPA increased the risk of generalized overweight by 5.8% (OR: 1.058, 95% CI: 1.034-1.084, P value for linear trend test <.001). No evidence of non-linear association was found between BPA and of generalized overweight risk.	

2.7 Circulatory outcomes

Study details	Appraisal details	Participants	Plastic exposure	Health outcomes	Findings	Subgroup Findings	AMSTAR score
**Dunder et al., 2019 [84] - Urinary bisphenol A and serum lipids: a meta-analysis of six NHAMES examination cycles (2003-2014). No COIs declared.**							
**Last search** 2003-2014<br><br>**Study types** Continuous cross-sectional surveillance data<br><br>**Included studies in the review** = NA<br><br>**Included studies in the meta-analysis** = NA	no studies to appraise	Children (≤ 17 years; n=4604); Adults (≥ 18 years; n=10989)<br><br>N = 15,593	**Type** BPA<br><br>**Route** NR<br><br>**Measure** Urinary (mmol/L)<br><br>**Exposure time** NR	Concentration BPA in low-density cholesterol (LDL-C) Beta coefficient and 95%CI	No association found between urinary BPA levels and LDL-C (b=-0.02, 95%CI: -0.05,0.01)	**Children - standard adjusted model**: No association found between urinary BPA levels and LDL-C (b=-0.005, 95%CI: -0.05,0.05) **Children - fully adjusted model**: No association found between urinary BPA levels and LDL-C (b=0.003, 95%CI: -0.05,0.05)<br><br>No association between urinary BPA levels and LDL-C in boys (β −0.03, 95%CI: −0.09 to 0.04) and girls (β 0.04, 95%CI: −0.04 to 0.11) (participants unspecified).	4
						**Adults - standard adjusted model**: No association found between	

Study details	Appraisal details	Participants	Plastic exposure	Health outcomes	Findings	Subgroup Findings	AMSTAR score
						urinary BPA levels and LDL-C (b=--0.02, 95%CI: -0.05,0.01; p=0.22 (); **Adults - fully adjusted model**: No association found between urinary BPA levels and LDL-C (b=--0.02, 95%CI: -0.05,0.01)<br><br>No association between urinary BPA levels and LDL-C in males (β −0.02, 95%CI: −0.07 to 0.02; 6 studies, participants unspecified) and in females (β −0.01, 95%CI: −0.05 to 0.03; 6 studies, participants unspecified)	
				Concentration BPA in high-density cholesterol (HDL-C) Beta coefficient and 95%CI	No association found between urinary BPA levels and HDL-C (b=-0.01, 95%CI: -0.02,0.00)	**Children - standard adjusted model**: No association found between urinary BPA levels and HDL-C (b=--0.01, 95%CI: -0.02,0.002); **Children - fully adjusted model**: No association found between urinary BPA levels and HDL-C (b=--0.01, 95%CI: -0.02,0.002)<br>No association between urinary BPA levels and HDL-C in boys (β -0.01, 95%CI: −0.03 to 0.003; 6 studies, participants unspecified) and girls (β -0.01, 95%CI: −0.03 to 0.007; 6 studies, participants unspecified)	
						**Adults - standard adjusted model**: No association found between urinary BPA levels and HDL-C (b=-0.012, 95%CI: -0.02,0.001); **Adults - fully adjusted model**: No association found between urinary	

Study details	Appraisal details	Participants	Plastic exposure	Health outcomes	Findings	Subgroup Findings	AMSTAR score
						BPA levels and HDL-C (b=-0.006, 95%CI: -0.01,0.003<br>No association between urinary BPA levels and HDL-C in males (β -0.008, 95%CI: −0.02 to 0.004; 6 studies, participants unspecified) and in females (β -0.01, 95%CI: −0.03 to 0.0002; 6 studies, participants unspecified)	
				Concentration in total cholesterol (TC) Beta coefficient and 95%CI	No association found between urinary BPA levels and TC (b=-0.02, 95%CI: -0.04,0.00)	**Children - standard adjusted model**: No association found between urinary BPA levels and TC (b= 0.008, 95%CI: -0.03,0.05); **Children - fully adjusted model.** No association found between urinary BPA levels and TC (b= 0.01, 95%CI: -0.03,0.05)<br>No association between urinary BPA levels and TC in boys (β 0.02, 95%CI: −0.04 to 0.07; 6 studies, participants unspecified) and girls (β -0.02, 95%CI: −0.08 to 0.04; 6 studies, participants unspecified)	
						**Adults - standard adjusted model**: No association found between urinary BPA levels and TC (b=--0.02, 95%CI: -0.04,0.004); **Adults - fully adjusted model**: No association found between urinary BPA levels and TC (b=--0.02, 95%CI: -0.01,0.003)<br>No association between urinary BPA levels and TC in males (β -0.02, 95%CI: −0.05 to 0.01; 6 studies, participants unspecified) and females (β -0.02, 95%CI: −0.05 to	

Study details	Appraisal details	Participants	Plastic exposure	Health outcomes	Findings	Subgroup Findings	AMSTAR score
						0.02; 6 studies, participants unspecified)	
				Concentration in triglycerides (TG)<br>Beta coefficient and 95%CI	No association found between urinary BPA levels and TG (b=--0.01, 95%CI: -0.03,0.01)	**Children - standard adjusted model**: No association found between urinary BPA levels and TG (b= 0.01, 95%CI: -0.02,0.05); **Children - fully adjusted model**: No association found between urinary BPA levels and TG (b= 0.01, 95%CI: -0.02,0.05)<br>No association between urinary BPA levels and TG in boys (β 0.04, 95%CI −0.003 to 0.09; 6 studies, participants unspecified) and girls (β -0.02, 95%CI −0.07 to 0.03; 6 studies, participants unspecified)	
						**Adults - standard adjusted model**: No association found between urinary BPA levels and TG (b=-0.02, 95%CI: -0.04,0.004); **Adults - fully adjusted model**: No association found between urinary BPA levels and TG (b=-0.021, 95%CI: -0.01,0.003)<br>No association between urinary BPA levels and TG in males (β -0.01, 95%CI −0.04 to 0.02; 6 studies, participants) and females (β -0.01, 95%CI −0.04 to 0.01; 6 studies, participants unspecified)	
				Concentration in apolipoprotein B (ApoB)	No association found between urinary BPA levels and ApoB (b=--0.89, 95%CI: -1.843,0.06)	**Children - standard adjusted model**: No association found between urinary BPA levels and ApoB (b=--0.48, 95%CI: -2.1,1.2); **Children - fully adjusted model**: No	

Study details	Appraisal details	Participants	Plastic exposure	Health outcomes	Findings	Subgroup Findings	AMSTAR score
				Beta coefficient and 95%CI		association found between urinary BPA levels and ApoB (b=-0.54, 95%CI: -2.3,1.2)<br>No association between urinary BPA levels and ApoB in boys (β -0.72, 95%CI: −2.8 to 1.4; 6 studies, participants unspecified) and girls (β -0.18, 95%CI: −2.9 to 2.6; 6 studies, participants unspecified)	
						**Adults - standard adjusted model**: No association found between urinary BPA levels and ApoB (b=-0.89, 95%CI: -1.8,0.06); **Adults - fully adjusted model**: No association found between urinary BPA levels and ApoB (b=-0.91, 95%CI: -1.82,-0.02)<br>No association between urinary BPA levels and ApoB in males (β -0.66, 95%CI: −1.9 to 0.6; 6 studies, participants unspecified) and females (β -0.98, 95%CI: −2.3 to 0.4; 6 studies, participants unspecified)	
**Golestanzadeh et al., 2019 [54] - Association of exposure to phthalates with cardiometabolic risk factors in children and adolescents: a systematic review and meta-analysis. No COI declared.**							
**Last search**<br>Dec-18<br><br>**Study types**<br>cohort (n=17)<br>cross sectional (n=15)<br>case-control (n=3)	STROBE checklist. ****Note this is a reporting guideline not a critical appraisal tool. ****The quality of the included observational studies was good and most of them gained a high score.	Children (≤ 18 years)<br><br>N = 24,943	**Type**<br>Phthalates<br><br>**Route**<br>Unspecified<br><br>**Measure**<br>Urine and serum (units unspecified)<br><br>**Exposure time**	Blood pressure (systolic)<br>Beta coefficient and 95% CI		Exposure to the phthalate metabolites was associated with increased systolic blood pressure; MEHHP (z= 0.16, 95%CI: 0.09, 0.23; 3 studies, 761 participants), mono(2-ethyl-5-oxohexyl)phthalate (MEOHP) (z= 0.12, 95%CI: 0.01, 0.24; 3 studies, 761 participants) but not MMP (z= 0.09, 95%CI:−0.03, 0.20: 2 studies, 518	5

Study details	Appraisal details	Participants	Plastic exposure	Health outcomes	Findings	Subgroup Findings	AMSTAR score
**Included studies in the review** = 35<br><br>**Included studies in the meta-analysis** = 23			Prenatal			participants), MEHP (z= 0.13, 95%CI:− 0.02, 0.28; 3 studies, 731 participants) and MBzP (z= 0.09, 95%CI:− 0.11, 0.29; 3 studies, 518 participants).	
				Blood pressure (diastolic)<br>Beta coefficient and 95% CI		No association between phthalate metabolites and diastolic blood pressure; MMP (z= 0.02, 95%CI: − 0.06, 0.11; 2 studies, 518 participants), MEHP (z= − 0.01, 95%CI: − 0.09, 0.08; 2 studies, 518 participants), MEHHP (z= 0.07, 95%CI: − 0.02, 0.15; 2 studies, 518 participants), MEOHP (z= 0.03, 95%CI: − 0.06, 0.12;2 studies, 518 participants) and MBzP (z= 0.04, 95%CI: − 0.05, 0.12; 2 studies, 518 participants)	
				HDL (lipid)<br>Beta coefficient and 95% CI		Exposure to the phthalate MEOHP was associated with HDL (z=0.31, 95%CI: 0.25, 0.37; 2 studies, 485 participants) but not MEHP (z= − 0.20, 95%CI:− 0.42, 0.03; 3 studies, 622 participants), MBP (z=− 0.15, 95%CI: − 0.88, 0.58; 2 studies, 397 participants), MEHHP (z= 0.20, 95%CI:− 0.23, 0.63; 2 studies, 485 participants), MBzP (z= − 0.11, 95%CI:− 0.47, 0.26; 3 studies, 1400 participants), MCPP (z= 0.11, 95%CI: − 0.10, 0.33; 2 studies, 1158 participants) and DEHP subgroups (z= 0.09 , 95%CI:− 0.26, 0.44; 4 studies, 3231 participants	

Study details	Appraisal details	Participants	Plastic exposure	Health outcomes	Findings	Subgroup Findings	AMSTAR score
				Triglyceride (Lipids)<br>Beta coefficient and 95% CI		No association between phthalate metabolites and triglycerides; MEHP (z= 0.2, 95%CI:− 0.06, 0.47; 3 studies, 787 participants), MBP (z= 0.08, 95%CI: − 0.18, 0.34; 2 studies, 397 participants), MEHHP (z= 0.01, 95%CI:− 0.05, 0.07; 2 studies, 485 participants), MEOHP (z= − 0.06, (%%CI: − 0.19, 0.06; 2 studies, 485 participants), MBzP (z=0.14, 95%CI:− 0.10, 0.37; 3 studies, 1400 participants), MCPP (z=− 0.04, 95%CI:− 0.09, 0.02; 2 studies, 1158 participants) and DEHP subgroups (z=− 0.11, 95%CI:− 0.31, 0.08; 4 studies, 3907 participants)	
**Fu et al., 2020 [87]- The association between environmental endocrine disruptors and cardiovascular disease: A systematic review and meta-analysis. No COI reported**							
**Last search**<br>Jan-19<br><br>**Study types**<br>Cross-sectional (n=17)<br>Retrospective cohort (n=7)<br>Prospective cohort (n=4)<br>Case-control (n=1)<br><br>**Included studies in the review =**<br>29<br><br>**Included studies in the meta-analysis =** 11	Newcastle Ottawa Scale. The authors only provide the final score per study with the following statement "The literatures were considered as high quality, medium quality,<br>and low quality with the corresponding scores of ≥7, 5–7, and<5."	Anybody in whom EED (environmental endocrine disruptor) exposure was pre-determined. No age limitations.<br><br>N = 41854	**Type**<br>Bisphenol A (BPA)<br><br>**Route**<br>Unspecified<br><br>**Measure**<br>Unspecified<br><br>**Exposure time**<br>Unspecified	Incidence of cardiovascular disease (CVD)<br>OR and 95%CI no logistic regression	Positive association between exposure to BPA and incidence of CVD (OR=1.19, 95%CI:1.03, 1.37; 10 studies, 23953 participants).	No subgroup analysis conducted	6

Study details	Appraisal details	Participants	Plastic exposure	Health outcomes	Findings	Subgroup Findings	AMSTAR score
			**Type**<br>Any Phthalate<br><br>**Route**<br>Unspecified<br><br>**Measure**<br><br>urine<br><br>**Exposure time**<br>Unspecified	Incidence of cardiovascular disease (CVD)<br>OR and 95%CI<br>No logistic regression		Subgrouping conducted based on phthalate subtype.<br>No association between MBzP exposure and incidence of CVD (OR=1.18, 95%CI:0.93, 1.51; 4 studies, 9,261 participants).<br>No association between MBP exposure and incidence of CVD OR=1.02, 95%CI:0.78, 1.32; 4 studies 9,261 participants).<br>No association between MECCP and incidence of CVD (OR=1.15, 95%CI:0.94, 1.41; 4 studies 9,261 participants).<br>No association between MEHHP exposure and incidence of CVD (OR=1.08, 95%CI:0.95,1.23; 4 studies 9,261 participants).<br>No association between MEHP exposure and incidence of CVD (OR=1.05, 95%CI:0.97, 1.13; 4 studies 9,261 participants).<br>No association between MEOHP exposure and incidence of CVD (OR=1.09, 95%CI:0.93, 1.26; 4 studies 9,261 participants).<br>No association between MEP exposure and incidence of CVD (OR=1.15, 95%CI:0.99,1.34; 4 studies 9,261 participants).<br>No association between MiBP exposure and CVD (OR=1.18, 95%CI:0.99,1.38; 4 studies 9,261 participants).<br>EED exposure is a risk factor for CVD. PCBs, BPA, OCPs, and PAEs	

Study details	Appraisal details	Participants	Plastic exposure	Health outcomes	Findings	Subgroup Findings	AMSTAR score
						have a great impact on the development and progression of CVD. No statistical significance was found in the pooled OR value between the eight metabolites. However, the pooled OR value of total PAEs was 1.11	
			**Type**<br>Polychlorinated biphenyl (PCBs)<br><br>**Route**<br>Unspecified<br><br>**Measure**<br>Unspecified<br><br>**Exposure time**<br>Unspecified	Prevalence cardiovascular disease (CVD) (categorisation unspecified)<br>OR and 95%CI<br>No logistic regression	Positive association between PCB exposure and incidence of CVD (OR=1.28, 95%CI:1.17, 1.39; 11 studies, 86296 participants).	Subgroup conducted based on PCB subtype. Positive association between PCB-138 exposure and incidence of CVD (OR=1.35, 95%CI:1.10, 1.66; 7 studies, 13409 participants). Positive association between PCB-153 and incidence of CVD (OR=1.35, 95%CI:1.13,1.62; 10 studies, 49326 participants). No association between exposure to PCB-180 and incidence of CVD (OR=1.19, 95%CI:0.98, 1.45; 9 studies,14735 participants). No association between exposure to total PCBs and incidence of CVD (1.32, 95%CI:0.97, 1.78; 4 studies, 8826 participants).	
**Rancière et al., 2015 [66] - Bisphenol A and the risk of cardiometabolic disorders: a systematic review with metaanalysis of the epidemiological evidence. No DOI declared**							
**Last search**<br>Aug-14<br><br>**Study types**<br>Cross-sectional (n=28) and Prospective Longitudinal (n=5) (pooled together)	A scoring system based on the established OHAT guidelines [31] adapted to reflect the characteristics of the included studies: longitudinal design (2 points), population-based study (1 point), outcome assessment including measurements (1 point), collection of at least 2 urine samples per	Adults or children (however only diabetes in adults, in an attempt to limit the analysis to type 2). Pregnant women were excluded.<br><br>N = 69,486	**Type**<br>Bisphenol A<br><br>**Route**<br>Non-specific<br><br>**Measure**<br>Urinary BPA and amniotic fluid (μg/L). Compared with extreme categories of urinary BPA levels (the highest vs.	Hypertension<br>OR with 95% CI	Exposure to BPA was +ve associated with hypertension in adults 1.41(95% CI:1.12,1.79, 2 studies, 4488 participants)	No subgroup analysis	7

Study details	Appraisal details	Participants	Plastic exposure	Health outcomes	Findings	Subgroup Findings	AMSTAR score
**Included studies in the review** = 33<br><br>**Included studies in the meta-analysis** = 12	participant (1 point), control for urine dilution (1 point), adjustment for dietary intake (1 point), and adjustment for socioeconomic variables (1 point). Studies were then classified as 'low quality' (total score between 0 and 2), 'medium quality' (total score between 3 and 5), or 'high quality' (total score between 6 and 8). Studies ranged from Low, medium to High. No discussion on the implications of quality on findings.		the lowest). Highest levels found a mean (SE) of 5.0 (0.3) ng/mL in boys and 4.6 (0.3) ng/mL in girls. Lowest: a median (interquartile range, IQR) of 0.60 (0.20–1.37) ng/mL.<br><br>**Exposure time**<br>non-specific				
**Park et al., 2016 [85] - Body burden of persistent organic pollutants on hypertension: a meta-analysis. No COI declared.**							
**Last search**<br>Jun-15<br><br>**Study types**<br>Cross-sectional (n=10) cohort (n=1)<br><br>**Included studies in the review =** 11<br><br>**Included studies in the meta-analysis =** 11	Newcastle Ottawa scale. We decided to include all 11 studies, since the Newcastle-Ottawa scale scores of selected studies were equal to or greater than 6 (moderate-high quality).	General population<br><br>N = 14,742	**Type**<br>Polychlorinated biphenyls (PCB)<br><br>**Route**<br>Unspecified<br><br>**Measure**<br>Serum (n=10) adipose tissue (n=1) (ng/g lipid or pg/g lipid or µg/L or µg/kg lipid)<br><br>**Exposure time**<br>Unspecified	Hypertension OR and 95%CI Test unspecified, logistic regression unspecified	No association between PCB-118 and hypertension (OR=1.26, 95%CI: 1.00, 1.58; 5 studies, 9134 participants)<br><br>No association between PCB-153 and hypertension (OR=1.09, 95%CI: 0.97, 1.23; 6 studies, 9431 participants)<br><br>No association between Dioxin-like PCBs and hypertension (OR=1.45, 95%CI: 1.00, 2.12; 5 studies, 8793 participants)<br><br>No association between Non-Dioxin-like PCBs and hypertension	No subgroup analysis conducted	7

Study details	Appraisal details	Participants	Plastic exposure	Health outcomes	Findings	Subgroup Findings	AMSTAR score
					(OR=1.00, 95%CI: 0.89, 1.12; 3 studies, 2048 participants)		
**Li et al., 2015 [83] - Mortality after exposure to polychlorinated biphenyls and polychlorinated dibenzofurans: A meta-analysis of two highly exposed cohorts. No COIs reported**							
**Last search**<br>No search<br><br>**Study types**<br>Cohort<br><br>**Included studies in the review =** 2<br><br>**Included studies in the meta-analysis =** 2	None	Adult men and women occupationally exposed to PCBs - workers from "Yusho" incident, Japan (1968) and "Yu-Cheng" incident, Taiwan (1979)<br><br>N = 3,467	**Type**<br>polychlorinated biphenyls (PCB)<br><br>**Route**<br>Yusho (1968) and Yucheng (1979) food contamination events<br><br>**Measure**<br>ICD-9 codes (cause of death)<br><br>**Exposure time**<br>Unspecified	Hypertension mortality	No association between PCB and hypertension standardized mortality (SMR=1.6, 95%CI: 0.9, 2.9; 2 studies, 3467 participants)	No association between PCB and hypertension standardized mortality in **males** (SMR=1.5, 95%CI: 0.7, 3.4; 2 studies, 1690 participants)<br>No association between PCB and **hypertension** standardized mortality in **females** (SMR=1.4, 95%CI: 0.3, 5.6; 2 studies, 1777 participants)	4
				Heart disease mortality	Exposure to PCBs was associated with an increased **heart disease** standardized mortality rate (SMR=1.3, 95%CI: 1.0, 1.7; 2 studies, 3467 participants)		
				Cerebrovascular disease mortality	No association between PCB and cerebrovascular disease standardized mortality (SMR=1.0, 95%CI: 0.8, 1.29; 2 studies, 3467 participants)	No association between PCB and cerebrovascular disease standardized mortality in **males** (SMR=0.9, 95%CI: 0.6, 1.2; 2 studies, 1690 participants)<br>No association between PCB and **cerebrovascular disease** standardized mortality in **females** (SMR=1.1, 95%CI: 0.8, 1.5; 2 studies, 1777 participants)	

2.8 Respiratory outcomes

Study details	Appraisal details	Participants	Plastic exposure	Health outcomes	Findings	Subgroup Findings	AMSTAR score
**Li et al., 2017 [88] - Phthalate esters and childhood asthma: A systematic review and congener-specific meta-analysis. No COI declared.**							
**Last search**<br>Oct-16<br><br>**Study types**<br>Case-control (n=3)<br>cross-sectional (n=4)<br>cohort (n=2)<br><br>**Included studies in the review** = 9<br><br>**Included studies in the meta-analysis** = 9	Newcastle-Ottawa scale. The score of the included cohort or case-control studies ranged from 6 to 8, and 7 to 9 for cross-sectional studies (moderate to high quality).	Children (≤18 years)<br><br>N = 3,406	**Type**<br>Phthalates<br><br>**Route**<br>Pre and postnatal exposure<br><br>**Measure**<br>Urine and Dust (units unspecified)<br><br>**Exposure time**<br>Unspecified	Childhood asthma risk<br>OR and 95%CI; Test unspecified, logistic regression unspecified	Our systematic review and congener-specific meta-analysis suggested a positive association between phthalate exposure and childhood asthma. Future studies are warranted to identify the underlying mechanisms.		9
			**Type**<br>DnBP			No association between prenatal urinary DnBP levels and childhood asthma (OR 0.83, 95%CI: 0.12 to 5.77; 2 studies, participants unspecified) and postnatal urinary DnBP metabolites and childhood asthma (OR 0.72, 95%CI: 0.48 to 1.10; 5 studies, participants unspecified)	
			**Type**<br>DiBP			No association between postnatal urinary DiBP metabolites and childhood asthma (OR 1.06, 95%CI: 0.67 to 1.66; 3 studies, participants unspecified)	
			**Type**<br>BBzP			There is an association between prenatal urinary BBzP levels and increased risk in childhood asthma (OR 1.38, 95%CI: 1.09 to 1.75); 3 studies, participants unspecified) but not with postnatal urinary BBzP levels (OR 1.19, 95%CI: 0.79 to	

Study details	Appraisal details	Participants	Plastic exposure	Health outcomes	Findings	Subgroup Findings	AMSTAR score
						1.80; 5 studies, participants unspecified)	
			**Type**<br>DEHP			No association between prenatal urinary DEHP levels and childhood asthma (OR 1.17, 95%CI: 0.90 to 1.52; 3 studies, participants unspecified)<br>No association between prenatal (OR 1.11, 95%CI: 0.97 to 1.26; 3 studies, participants unspecified) and postnatal (OR 0.76, 95%CI: 0.32 to 1.79; 5 studies, participants unspecified) urinary DEHP metabolites and childhood asthma	
			**Type**<br>DiNP			No association between postnatal DiNP and childhood asthma (OR 1.21, 95%CI: 0.48 to 3.05; 2 studies, participants unspecified)	
**Wu et al., 2020 [90] - Association between phthalate exposure and asthma risk: A meta-analysis of observational studie. No COI declared**							
**Last search**<br>Jan-20<br><br>**Study types**<br>Case-control (n=2)<br>Cohort (n=7)<br>Cross-sectional (n=5)<br><br>**Included studies in the review** = 14<br><br>**Included studies in the meta-analysis** = 14	Study quality was assessed using the Newcastle-Ottawa Scale (NOS) for cohort and case-control studies, and an adapted form of the NOS cohort scale for cross-sectional studies. The estimated quality ranged from 7 to 9 in the cohort studies, 7–8 in the case control studies, and 6–8 in the cross-sectional studies, which indicated moderate to high quality in the included studies.	Men and women of any age, the only unique characteristic was that phthalate metabolites had to measured using urine biomarkers *Note* Authors only provide the number of CASES and not total participants in the studies.<br><br>N (cases) = 1731	**Type**<br>MBzP<br>(Mono-benzyl phthalate)<br><br>**Route**<br>Unspecified<br><br>**Measure**<br>Concentration in urine (µg/L)<br><br>**Exposure time**<br>Subgrouped based on prenatal or postnatal exposure	Prevalence<br>Asthma<br>OR and 95%CI<br>No logistic regression	There was a positive association between exposure to MBzP and asthma risk (OR=1.17, 95%CI: 1.06, 1.28; 15 studies)	The first subgroup was based on age. In children there was a positive association between MBzP exposure and asthma risk (OR=1.17, 95%CI: 1.05, 1.29; 12 studies). No association was found for adults (OR=1.17, 95%CI:0.94, 1.46; 3 studies).<br>The second subgroup analysis was conducted based on location, positive associations were found for studies from Europe (OR=1.16, 95%CI:1.02, 1.32; 5 studies) and North American (OR=1.23, 95%CI:1.05, 1.44; 7 studies), but no association was found for studies	5

Study details	Appraisal details	Participants	Plastic exposure	Health outcomes	Findings	Subgroup Findings	AMSTAR score
		participants numbers are all unspecified for the accompanying meta-analyses.				from Asia (OR=1.08, 95%CI:0.37,3.19; 4 studies).<br>The third subgroup analysis was conducted based exposure time. A positive association was identified for postnatal exposure (OR=1.17, 95%CI:1.03, 1.33; 10 studies) but no association was identified for prenatal exposure (OR=1.15, 95%CI 1.01, 1.32, 6 studies).<br>The fourth subgroup analysis was conducted based on gender. No associations were found for either male (OR=1.19, 95%CI 0.99,1.41; 5 studies) or female (OR=1.04, 95%CI: 0.77,1.42; 4 studies).	
			**Type**<br>MnBP (Mono-n-butyl phthalate)<br><br>**Route**<br>Unspecified<br><br>**Measure**<br>Concentration in urine (µg/L)<br><br>**Exposure time**<br>Subgrouped based on prenatal or postnatal exposure	Prevalence Asthma<br>OR and 95%CI	There was no association between exposure to MnBP and asthma risk (OR=1.03, 95%CI:0.85, 1.24; 11 studies)	The first subgroup analysis was conducted based on age. There was no association for children (OR=0.97, 95%CI:0.85, 1.09; 8 studies) or adults (OR=1.35, 95%CI:0.93, 1.96; 3 studies).<br>The second subgroup analysis was conducted based on study location. There was no association for studies from Europe (OR=0.98,95%CI:0.74,1.29; 4 studies) and North America (OR=1.09, 95%CI: 0.89, 1.33; 7 studies).<br>The third subgroup analysis was conducted based on exposure time. There was no association for postnatal (OR=0.95, 95%CI:0.78, 1.16; 7 studies) or prenatal exposure (OR=1.07, 95%CI:0.8,	

Study details	Appraisal details	Participants	Plastic exposure	Health outcomes	Findings	Subgroup Findings	AMSTAR score
						1.42; 4 studies).<br>The fourth subgroup analysis was conducted based on gender. No associations were found for males (OR=0.98, 95%CI:0.82, 1.16; 4 studies) or females (OR=0.84, 95%CI:0.56, 1.25; 3 studies).	
			**Type**<br>MiBP (Mono-isobutyl phthalate)<br><br>**Route**<br>Unspecified<br><br>**Measure**<br>Concentration in urine (μg/L)<br><br>**Exposure time**<br>Subgrouped based on prenatal or postnatal exposure	Prevalence<br>Asthma<br>OR and 95%CI<br>No logistic regression	There was no association between exposure to MiBP and asthma risk (OR=1.05, 95%CI0.93, 1.19; 10 studies)	The first subgroup analysis was conducted based on age. There was no association for children (OR=1.04, 95%CI:0.91, 1.19; 7 studies) or adults (OR=1.11, 95%CI: 0.84, 1.47; 3 studies).<br>The second subgroup was conducted based on study location. There was no association for studies from Europe (OR=1.05, 95%CI:0.90, 1.23; 4 studies) or North America (OR=1.06, 95%CI:0.87, 1.29; 6 studies).<br>The third subgroup was conducted based on exposure time. There was no association between postnatal (OR=1.06, 95%CI:0.89,1.27; 7 studies) or prenatal exposure (OR=1.05, 95%CI: 0.88, 1.24; 3 studies).<br>The fourth subgroup was conducted based on gender. There was no association between males (OR=1.08, 95%CI:0.88, 1.33; 4 studies) or females (OR=0.81, 95%CI:0.51, 1.29; 3 studies).	
			**Type**<br>MEP (Mono-ethyl phthalate)	Prevalence<br>Asthma	There was no association between exposure to MEP and	The first subgroup analysis was conducted based on age. There was no association for children	

Study details	Appraisal details	Participants	Plastic exposure	Health outcomes	Findings	Subgroup Findings	AMSTAR score
			**Route**<br>Unspecified<br><br>**Measure**<br>Concentration in urine (µg/L)<br><br>**Exposure time**<br>Subgrouped based on prenatal or postnatal exposure	OR and 95%CI<br>No logistic regression	asthma risk (OR=1.03, 95%CI: 0.96, 1.12; 13 studies)	(OR=1.02, 95%CI:0.94, 1.11; 10 studies) or adults (OR=1.11, 95%CI:0.89, 1.39; 3 studies).<br>The second subgroup analysis was conducted based location. There was no association for studies coming from Europe (OR=1.06, 95%CI:0.9, 1.24; 4 studies), North America (OR=1.03, 95%CI:0.93, 1.14; 7 studies) or Asia (OR=1.03, 95%CI: 0.86, 1.25; 3 studies).<br>The third subgroup analysis was conducted based on exposure time. No association was found for postnatal (OR=1.08, 95%CI:0.95,1.23; 9 studies) or prenatal exposure (OR=1.02, 95%ci:0.93, 1.12; 5 studies).<br>The fourth subgroup analysis was conducted based on gender. No association was found for males (OR=1.12, 95%CI 0.97. 1.31; 5 studies) or females (OR=0.94, 95%CI:0.58, 1.53; 3 studies).	
			**Type**<br>MEHP (Mono-2-ethylhexyl phthalate)<br><br>**Route**<br>Unspecified<br><br>**Measure**<br>Concentration in urine (µg/L)<br><br>**Exposure time**<br>Subgrouped based on prenatal or postnatal exposure	Asthma Risk (diagnosis of asthma)<br>OR and 95%CI<br>No logistic regression	There was no association between exposure to MEHP and asthma risk (OR=1.04, 95%CI:0.89, 1.20;5 studies) in children	The first subgroup analysis was conducted based on location. No association was found for studies coming from Europe (OR=1.04, 95%CI:0.89, 1.21; 3 studies) or from Asia (OR=1.14, 95%CI:0.48, 2.71; 3 studies).<br>The second subgroup analysis was conducted based on exposure time. These was no association for postnatal (OR=0.78, 95%CI:0.41, 1.48; 3 studies) or prenatal	

Study details	Appraisal details	Participants	Plastic exposure	Health outcomes	Findings	Subgroup Findings	AMSTAR score
						exposure (OR=1.06, 95%CI0.91, 1.23; 3 studies).<br>The third subgroup analysis was conducted based on gender. No association was found for males (OR=0.99, 95%CI: 0.81, 1.19; 2 studies) or females (OR=1.04, 95%CI:0.10, 10.62; 1 study).	
			**Type**<br>MEHHP (Mono-(2-ethyl-5-hydroxyhexul) phthala)<br><br>**Route**<br>Unspecified<br><br>**Measure**<br>Concentration in urine (μg/L)<br><br>**Exposure time**<br>Subgrouped based on prenatal or postnatal exposure	Prevalence<br>Asthma<br>OR and 95%CI<br>No logistic regression	There was a positive association between MEHHP exposure and asthmas risk (OR=1.13, 95%CI: 1.03, 1.24; 5 studies) in children.	The first subgroup analysis was conducted based on location. No association was found for studies from Europe (OR=1.11, 95%CI:0.94, 1.31; 3 studies) or for North America (OR=1.03, 95%CI:0.89, 1.20; 1 study). A positive association was found for studies from Asia (OR=1.33, 95%CI:1.11, 1.60; 1 study).<br>The second subgroup analysis was conducted based on exposure time. A positive association was found for postnatal exposure (OR=1.30, 95%CI:1.09, 1.56; 2 studies), no association was found for prenatal exposure (OR=1.07, 95%CI:0.96, 1.20; 3 studies).	
			**Type**<br>MEOHP (Mono-(2-ethyl-5-oxohexyl) phthalate)<br><br>**Route**<br>Unspecified<br><br>**Measure**	Prevalence<br>Asthma<br>OR and 95%CI<br>No logistic regression	There was no association between MEOHP exposure and asthma risk (OR=1.09, 95%CI:0.77, 1.53; 3 studies) in children.	Subgroup was conducted based on exposure time. No association was found for postnatal (OR=0.5, 95%CI:0.23, 1.31; 1 study) or prenatal exposure (OR=1.19, 95%CI: 0.88, 1.61; 2 studies).	

Study details	Appraisal details	Participants	Plastic exposure	Health outcomes	Findings	Subgroup Findings	AMSTAR score
			Concentration in urine (µg/L)<br><br>**Exposure time**<br>Subgrouped based on prenatal or postnatal exposure				
			**Type**<br>MECPP (Mono-(2-ethyl-5-carboxypentyl) phthalate)<br><br>**Route**<br>Unspecified<br><br>**Measure**<br>Concentration in urine (µg/L)<br><br>**Exposure time**<br>Subgrouped based on prenatal or postnatal exposure	Prevalence<br>Asthma<br>OR and 95%CI<br>No logistic regression	There was no association between MECPP exposure and asthma risk (OR=1.2, 95%CI: 1.0, 1.42; 3 studies) in children.	Subgroup analysis was conducted based on exposure time. There was no association for postnatal exposure (OR=0.59, 95%CI: 0.25, 1.41; 1 study). There was a positive association for prenatal exposure (OR=1.23, 95%CI: 1.03, 1.47; 2 studies).	
			**Type**<br>ΣDEHP ((Sum of all) Di-2-ethylhexyl phthalate)<br><br>**Route**<br>Unspecified<br><br>**Measure**<br>Concentration in urine (µg/L)<br><br>**Exposure time**<br>Subgrouped based on prenatal or postnatal exposure	Prevalence<br>Asthma<br>OR and 95%CI<br>No logistic regression	There was no association between ΣDEHP exposure and asthma risk (OR=0.99, 95%CI:0.8, 1.22; 11 studies).	The first subgroup analysis was conducted based on age. No association was found for children (OR=0.87, 95%CI:0.67, 1.14; 8 studies) or adults (OR=1.27, 95%CI:0.99, 1.61; 3 studies).<br>The second subgroup was conducted based on location. There was no association for studies from Europe (OR=1.16, 95%CI:1.0,1.34; 4 studies), North America, (OR=0.81, 95%CI:0.57, 1.17; 6 studies) and Asia (OR=1.89l, 95%CI:0.79, 4.53; 2 studies).<br>No association for both postnatal (OR= 1.04, 95%CI: 0.71, 1.54; 7 studies) and prenatal exposure	

Study details	Appraisal details	Participants	Plastic exposure	Health outcomes	Findings	Subgroup Findings	AMSTAR score
						(OR= 1.08, 95%CI:0.92, 1.26; 5 studies)	
			**Type**<br>MCNP (Mono-(carboxynonyl) phthalate)<br><br>**Route**<br>Unspecified<br><br>**Measure**<br>Concentration in urine (µg/L)<br><br>**Exposure time**<br>Subgrouped based on prenatal or postnatal exposure	Prevalence<br>Asthma<br>OR and 95%CI<br>No logistic regression	There was no association between MCNP and asthma risk (OR=1.10, 95%CI:0.98, 1.24; 7 studies).	The first subgroup was conducted based on age. There was no association for children (OR=1.15, 95%CI:1,1.31; 5 studies) or adults (OR=1.0, 95%CI:0.8, 1.24; 2 studies).<br>The second subgroup analysis was conducted based on location. A positive association was found for studies from Europe (OR=1.18, 95%CI:1.02,1.37; 2 studies). No association was found for studies from North America (OR=0.99, 95%CI:0.82, 1.19; 5 studies).<br>The third subgroup analysis was conducted based on exposure time. There was no association for postnatal (OR=1.07, 95%CI:0.92, 1.26; 5 studies) or prenatal exposure (OR=1.14, 95%CI:0.96, 1.34; 2 studies).<br>The fourth subgroup analysis was conducted based on gender. There was no association for males (OR=1.12, 95%CIL0.95, 1.33; 3 studies) or females (OR=1.02, 95%CI:0.73, 1.44; 2 studies).	
			**Type**<br>MCPP (Mono-(3-carboxypropyl) phthalate)<br><br>**Route**	Prevalence<br>Asthma<br>OR and 95%CI<br>No logistic regression	There was no association between MCPP exposure and asthma risk (OR=1.04, 95%CI:0.91, 1.19; 8 studies).	The first subgroup analysis was conducted based on age. No association was found for children (OR=0.97, 95%CI:0.83, 1.13; 6 studies) or adults (OR=1.32, 95%CI:1.0, 1.75; 2 studies).<br>The second subgroup analysis was	

Study details	Appraisal details	Participants	Plastic exposure	Health outcomes	Findings	Subgroup Findings	AMSTAR score
			Unspecified<br><br>**Measure**<br>Concentration in urine (µg/L)<br><br>**Exposure time**<br>Subgrouped based on prenatal or postnatal exposure			conducted based on location. There was no association found for studies from Europe (OR=0.96, 95%CI:0.8, 1.15; 2 studies) or North America (OR=1.14, 95%CI: 0.94, 1.4; 6 studies).<br>The third subgroup analysis was conducted based on exposure time. There was no association for postnatal (OR=1.09, 95%CI:0.91, 1.32; 5 studies) or prenatal exposure (OR=0.99, 95%CI: 0.81, 1.2; 3 studies).<br>The fourth subgroup analysis was conducted based on gender. There was no association for males (OR=0.93, 95%CI:0.76, 1.14; 4 studies) or females (OR=1.36, 95%CI:0.98, 1.88; 3 studies).	
			**Type**<br>MCOP (Monocarboxy-isooctyl phthalate)<br><br>**Route**<br>Unspecified<br><br>**Measure**<br>Concentration in urine (µg/L)<br><br>**Exposure time**<br>Subgrouped based on prenatal or postnatal exposure	Prevalence<br>Asthma<br>OR and 95%CI<br>No logistic regression	There was no association between MCOP exposure and asthma risk (OR=1.13, 95%CI:0.99, 1.28; 5 studies).	The first subgroup analysis was conducted based on age. There was a positive association for children (OR=1.19, 95%CI: 1.02, 1.37; 4 studies), but no association for adults (OR=0.96, 95%CI:0.73, 1.25; 1 study).<br>The second subgroup analysis was conducted based on study location. There was no association for studies from Europe (OR=1.13, 95%CI:0.95, 1.34; 2 studies) or North America (OR=1.10, 95%CI: 0.74, 1.64; 3 studies).<br>The third subgroup analysis was conducted based on exposure time. There was no association for	

Study details	Appraisal details	Participants	Plastic exposure	Health outcomes	Findings	Subgroup Findings	AMSTAR score
						postnatal (OR=1.08, 95%CI:0.9, 1.31; 3 studies) or prenatal exposure (OR=1.17, 95%CI: 0.98, 1.41; 2 studies.	
**Luo et al., 2020 [89] - Exposure to perfluoroalkyl substances and allergic outcomes in children: A systematic review and meta-analysis. No COIs declared**							
**Last search**<br>Oct-19<br><br>**Study types**<br>cohort (n=10), cross sectional (n=2), case control (n=1)<br><br>**Included studies in the review** = 13<br><br>**Included studies in the meta-analysis** = 13	The 9-star Newcastle-Ottawa Scale (NOS). Two studies were classified as medium quality and 11 as high quality.	Children (birth to 18 years of age)<br><br>N = 11,255	**Type**<br>Perfluoroalkyl substances (PFAS)<br><br>**Route**<br>Prenatal exposure, except three studies. Details of exposure not reported in postnatal exposure studies.<br><br>**Measure**<br>Cord blood or plasma, serum, maternal serum or plasma (units NR)<br><br>**Exposure time**<br>NR	Childhood asthma<br>Odds ratio 95%CI. Analysis type NR.	No association between childhood asthma and PFOS (OR=1.11, 95%CI: 0.88, 1.40; 8 studies, 7050 participants), PFOA (OR=1.11, 95% CI: 0.85, 1.24; 8 studies, 7050 participants) or PFNA (OR=0.99, 95%CI: 0.81-1.21; 8 studies, 7050 participants) or PFHxS (OR=1.02, 95%CI: 0.85-1.24; 8 studies, 7050 participants)	When studies were grouped by exposure, there was no association found for PFOA and prenatal (OR 0.92, 95%CI: 0.79 to 1.07; 6 studies, participants unspecified) and postnatal (OR 2.05, 95%CI: 0.58 to 7.27; 2 studies, participants unspecified) exposure;<br>PFOS and prenatal exposure (OR 0.99, 95%CI: 0.80 to 1.22; 6 studies, participants unspecified) and postnatal exposure (OR 1.57, 95%CI: 0.62 to 4.00; 2 studies, participants unspecified); PFHxS and prenatal exposure (OR 0.94, 95%CI: 0.84 to 1.05; 6 studies, participants unspecified) and postnatal exposure (OR 1.83, 95%CI: 0.45 to 7.38; 2 studies, participants unspecified), and PFNA and prenatal (OR 0.90, 95%CI: 0.74 to 1.06; 6 studies, participants unspecified) and postnatal (OR 1.52, 95%CI: 0.60 to 3.85; 2 studies, participants unspecified) exposure.	7
				Childhood wheeze<br>Odds ratio 95%CI. Analysis type NR.	No association between childhood wheeze and PFOS (OR=0.90, 95%CI: 0.78, 1.04; 6 studies, 6672 participants),	When studies were grouped by exposure, there was no association found for PFOA and prenatal exposure (OR 1.03, 95%CI: 0.90 to 1.17; 5 studies, participants	

Study details	Appraisal details	Participants	Plastic exposure	Health outcomes	Findings	Subgroup Findings	AMSTAR score
					PFOA (OR=1.03, 95%CI: 0.93, 1.15; 6 studies, 6672 participants), PFHxS (OR=0.97, 95%CI: 0.87, 1.08; 6 studies, 6672 participants), or PFNA (OR=0.98, 95%CI: 0.88, 1.08; 6 studies, 6672 participants).	unspecified); PFOS and prenatal exposure (OR 0.91, 95%CI: 0.76 to 1.09; 5 studies, participants unspecified); PFHXs and prenatal exposure (OR 1.00, 95%CI: 0.89 to 1.13; 5 studies, participants unspecified); and PFNA and prenatal exposure (OR 0.99, 95%CI: 0.86 to 1.13; 5 studies, participants unspecified)	
				Allergic rhinitis Odds ratio 95%CI. Analysis type NR.	Exposure to PFOA was significantly associated with risk of allergic rhinitis in children (OR=1.32, 95%CI: 1.13, 1.55; 4 studies, 3396 participants). There was no association between risk of allergic rhinitis and PFOS (OR=1.07, 95%CI: 0.89, 1.29; 4 studies, 3396 participants), PFHxS (OR=0.94, 95%CI: 0.79, 1.13; 4 studies, 3396 participants), or PFNA (OR=0.99, 95%CI: 0.71, 1.37; 4 studies, 3396 participants).	When studies were grouped by exposure, there was no association found in PFOA and prenatal exposure (OR 1.29, 95%CI: 1.00 to 1.66; 3 studies, participants unspecified); PFOS and prenatal exposure (OR 0.97, 95%CI: 0.74 to 1.29; 3 studies, participants unspecified); PFHxS and prenatal exposure (OR 0.99, 95%CI: 0.84 to 1.16; 3 studies, participants unspecified); and PFNA and prenatal exposure (OR 0.83, 95%CI: 0.47 to 1.46; 3 studies, participants unspecified)	
**Gascon et al., 2014 [91] - Prenatal Exposure to DDE and PCB 153 and Respiratory Health in Early Childhood: A Meta-Analysis. No COI declared.**							
**Last search**<br>Unspecified<br><br>**Study types**<br>Cohort	unspecified	Mother-children pairs<br><br>N = 4,608	**Type**<br>Polychlorinated biphenyl (PCB)-153<br><br>**Route**<br>Maternal/prenatal	Bronchitis and/or wheeze (<18 months age)	No association between PCBs and presence of bronchitis and/or wheeze in infants < 18 months old (RR=1.02,	No subgroup analysis	3

Study details	Appraisal details	Participants	Plastic exposure	Health outcomes	Findings	Subgroup Findings	AMSTAR score
**Included studies in the review =** NA<br><br>**Included studies in the meta-analysis =** 10			**Measure**<br>Maternal whole blood or serum/ cord plasma or serum/breast milk (ng/L or ng/g lipid)<br><br>**Exposure time**<br>Prenatal	RR and 95%CI (continuous per log, ng/L);logistic regression	95%CI: 0.96, 1.08; 9 studies, 4394 participants)		
				Bronchitis (<18 months age) RR and 95%CI (continuous per log, ng/L);logistic regression	No association between PCBs and presence of bronchitis in infants < 18 months old (RR=1.06, 95%CI: 1.01, 1.12; 7 studies, 2990 participants) (P=0.89)	No subgroup analysis	

2.9 Skin related outcomes

Study details	Appraisal details	Participants	Plastic exposure	Health outcomes	Findings	Subgroup Findings	AMSTAR score
**Luo et al., 2020 [89] - Exposure to perfluoroalkyl substances and allergic outcomes in children: A systematic review and meta-analysis. No COIs declared**							
**Last search**<br>Oct-19<br><br>**Study types**<br>cohort (n=10), cross sectional (n=2), case control (n=1)<br><br>**Included studies in the review =** 13<br><br>**Included studies in the meta-analysis =** 13	The 9-star Newcastle-Ottawa Scale (NOS). Two studies were classified as medium quality and 11 as high quality.	Children (birth to 18 years of age)<br><br>N = 11,255	**Type**<br>Perfluoroalkyl substances (PFAS)<br><br>**Route**<br>Prenatal exposure, except three studies. Details of exposure not reported in postnatal exposure studies.<br><br>**Measure**<br>Cord blood or plasma, serum, maternal serum or plasma (units NR)<br><br>**Exposure time**<br>NR	Childhood eczema Odds ratio 95%CI. Analysis type NR.	PFNA exposure was found to be inversely associated with childhood eczema (OR=0.89, 95%CI: 0.80, 0.99; 5 studies, 5276 participants). No association between childhood eczema and exposure to PFOS (OR=0.91, 95%CI: 0.81, 1.02; 5 studies, 5276 participants), PFOA (OR=0.99, 95%CI: 0.88, 1.10; 5 studies, 5276 participants), PFHxS (OR=1.07, 95%CI: 0.96,		7

Study details	Appraisal details	Participants	Plastic exposure	Health outcomes	Findings	Subgroup Findings	AMSTAR score
					1.20; 5 studies, 5276 participants) was found.		
				Atopic dermatitis	PFOS exposure was found to be significantly associated with atopic dermatitis (OR=1.26, 95%CI: 1.01, 1.58; 4 studies, 2650 participants). There was no association between atopic dermatitis and PFOA (OR=1.39, 95%CI: 0.89, 2.18; 4 studies, 2650 participants), PFHxS (OR=1.08, 95%CI: 0.92, 1.27; 4 studies, 2650 participants), or PFNA (OR=0.96, 95%CI: 0.65, 1.43; 4 studies, 2650 participants).		

2.10 Cancer outcomes

Study details	Appraisal details	Participants	Plastic exposure	Health outcomes	Findings	Subgroup Findings	AMSTAR score
**Roy et al., 2015 [62] - Integrated Bioinformatics, Environmental Epidemiologic and Genomic Approaches to Identify Environmental and Molecular Links between Endometriosis and Breast Cancer. No COI declared.**							
**Last search**<br>Unspecified<br><br>**Study types**<br>Case-control (n=20), cohort (n=2), cross sectional (n=1)	No critical appraisal	Breast cancer patients and matched healthy controls<br><br>N = 9,781	**Type**<br>Polychlorinated biphenyl (PCB)<br><br>**Route**<br>NR<br><br>**Measure**	Breast cancer OR and 95% Cis; unadjusted and/or adjusted logistic regression models.	No association between PCB exposure and risk of developing breast cancer in women (OR= 1.33, 95%CI: 0.72, 2.65; 6 studies, 2458 participants).	No subgroup analysis conducted	3

Study details	Appraisal details	Participants	Plastic exposure	Health outcomes	Findings	Subgroup Findings	AMSTAR score
**Included studies in the review =** 23<br><br>**Included studies in the meta-analysis =** 12<br><br>**Study types**<br>Case-control<br><br>**Included studies in the review =** 6			Serum or plasma (ng/g)<br><br>**Exposure time**<br>NR				
		Women with endometriosis and matched healthy controls<br><br>N = 1380 (case=542; control=838)	**Type**<br>Polychlorinated biphenyl (PCB)<br><br>**Route**<br>NR<br><br>**Measure**<br>Serum (ng/g)<br><br>**Exposure time**<br>NR	Endometriosis<br>OR and 95%CI<br>No logistic regression	Exposure to PCB was associated with increased odds of developing endometriosis in women (OR= 1.91, 95%CI: 1.05, 5.54; 6 studies, 1380).	No subgroup analysis conducted	
**Zani et al., 2013 [92] - Polychlorinated Biphenyls and Cancer: An Epidemiological Assessment. no COI reported.**							
**Last Search**<br>Dec-12<br><br>**Study types**<br>Case-control and cohort<br><br>**Included studies in the review =** 29<br><br>**Included studies in the meta-analysis =** 29	No critical appraisal was performed	General population and men/women occupationally exposed to PCBs (including workers from "Yusho" incident, Japan [1968] and "Yu-Cheng" incident, Taiwan [1979])<br><br>N = 16,067	**Type**<br>polychlorinated biphenyl (PCB)<br><br>**Route**<br>rice oil accidentally contaminated by PCBs and polychlorinated<br><br>**Measure**<br>dibenzofurans (PCDFs)<br>(ng/g Lipid)<br><br>**Exposure time**<br>single incident	Non-Hodgkin lymphoma<br>OR and 95%CIs; quantiles	Exposure to PCBs was associated with an increased OR of non-Hodgkin lymphoma for the highest compared to the lowest PCB serum levels (OR=1.40, 95%CI: 1.14–1.71; 11 studies, 4422 participants)	No association found for individual measures of exposure and non-Hodgkin's lymphoma in subgroup of cohort studies (OR= 1.34, 95%CI: 0.97, 1.86; 7 studies, participants unspecified)<br>Exposure to PCBs in subgroup of case control studies were associated with a risk of Non-Hodgkin's lymphoma (OR= 1.51, 95%CI: 1.17, 1.96; 4 studies, participants unspecified)	2
				Breast Cancer<br>OR and 95%CIs; quantiles	No association between PCB serum levels and risk of breast cancer (OR=1.15, 95%CI: 0.92–1.43; 18 studies, 11,645 participants)	No association found for PCBs and breast cancer for subgroups of cohort studies (OR=1.01, 95%CI: 0.78, 1.31; 6 studies, participants unspecified) and case-control studies (OR=1.19, 95%CI: 0.92,	

Study details	Appraisal details	Participants	Plastic exposure	Health outcomes	Findings	Subgroup Findings	AMSTAR score
						1.43; 12 studies, participants unspecified)	
**Zhang et al., 2015 [93] - Environmental Polychlorinated Biphenyl Exposure and Breast Cancer Risk: A MetaAnalysis of Observational Studies.No COI reported.**							
**Last search**<br>Nov-2014<br><br>**Study types**<br>prospective cohort (n=9)<br>retrospective cohort (n=16)<br><br>**Included studies in the review** = 25<br><br>**Included studies in the meta-analysis** = 25	Newcastle-Ottawa Scale: moderate to high (5 to 9 stars) - prospective studies higher (7 to 9 stars) than retrospective studies (5 to 8 stars).	Female (age: 18 years and over)<br><br>N = 12,866	**Type**<br>polychlorinated biphenyl (PCB)<br><br>**Route**<br>unspecified<br><br>**Measure**<br>adipose tissue, serum or plasma ng/g lipid<br><br>**Exposure time**<br>unspecified	Risk of Breast Cancer<br>OR and 95%CI median and/or interquartile range; geometric mean and/or standard deviation	No association between PCB and risk of breast cancer in adult women (OR=1.09, 95%CI: 0.97, 1.22; 25 studies, 12,866 participants); Sensitivity analysis excluding 3 retrospective studies with divergent ORs<br>OR 1.06, 95% CI 0.98 to 1.15; 22 studies, 11,729 participants	No association in subgroups of retrospective studies by specimen type: Serum/plasma<br>(OR 1.12, 95%CI 0.95 to 1.32; 14 studies, 7,556 participants),<br>Adipose tissue (OR 1.06. 95%CI 0.70 to 1.60; 2 studies, 985 participants).	8
**Leng et al., 2016 [94] - Polychlorinated biphenyls and breast cancer: A congener-specific meta-analysis - No COIs declared**							
**Last search**<br>Jan-15<br><br>**Study types**<br>Case-control (n=11);<br>Nested case-control (n=5)<br><br>**Included studies in the review** = 16<br><br>**Included studies in the meta-analysis** = 13	Newcastle Ottawa Scale (NOS) - 5/8 (n=2); 6/8 (n=5); 7/8 (n=1)	Female; evidence of exposure to any one of 209 PCB congeners<br><br>N = 7,041	**Type**<br>polychlorinated biphenyls (PCB) - PCB-187<br><br>**Route**<br>Unspecified<br><br>**Measure**<br>serum (n=9); adipose tissue (n=7) - ng/g<br><br>**Exposure time**<br>Unspecified	Breast Cancer<br>Adjusted OR and 95%CI - (25th, and 75th percentiles)	Exposure to PCB-187 was associated with an increased odds in developing breast cancer in women (OR=1.18, 95%CI: 1.01,1.39; 7 studies, 1456 participants)	No subgroup analysis conducted	8
			**Type**<br>polychlorinated biphenyls (PCB) - PCB-118		No association between PCB-118 and risk of breast cancer in women (OR=1.32, 95%CI: 0.98,	Subgroup based on sample size	

Study details	Appraisal details	Participants	Plastic exposure	Health outcomes	Findings	Subgroup Findings	AMSTAR score
					1.78; 9 studies, 2,446 participants)		
			**Type** polychlorinated biphenyls (PCB) - PCB-138		No association between PCB-138 and risk of breast cancer in women (OR=1.08, 95%CI: 0.99, 1.17; 11 studies, 2,911 participants)	No subgroup analysis conducted	
			**Type** polychlorinated biphenyls (PCB) - PCB-156		No association between PCB-156 and risk of breast cancer in women (OR=1.19, 95%CI: 0.85, 1.67; 6 studies, 1,506 participants	Subgroup based on statistical method (variable type) PCB as categorical variable There is an association OR 1.35, 95%CI: 1.02 to 1.78; 5 studies, 1,202 participants	
			**Type** polychlorinated biphenyls (PCB) - PCB-170		No association between PCB-170 and risk of breast cancer in women (OR=1.28, 95%CI: 0.89, 1.86; 6 studies, 1,334 participants)	Subgroup based on PCB levels PCB 170 ≥ 12.5 ng/g lipid No association OR 1.05, 95%CI: 0.84 to 1.32; 5 studies, 1,274 participants	
			**Type** polychlorinated biphenyls (PCB) - PCB-99		Exposure to PCB-99 was associated with an increased odds in developing breast cancer in women (OR=1.36, 95%CI: 1.02, 1.80; 4 studies, 970 participants)	No subgroup analysis conducted	
			**Type** polychlorinated biphenyls (PCB) - PCB-153		No association between PCB-153 and risk of breast cancer in women (OR=1.04, 95%CI: 0.81, 1.34; 11 studies, 2,881 participants)		

Study details	Appraisal details	Participants	Plastic exposure	Health outcomes	Findings	Subgroup Findings	AMSTAR score
			**Type**<br>polychlorinated biphenyls (PCB) - PCB-180		No association between PCB-180 and risk of breast cancer in women (OR=1.02, 95%CI: 0.81, 1.29; 11 studies, 2,881 participants)		
			**Type**<br>polychlorinated biphenyls (PCB) - PCB-183		Exposure to PCB-183 was associated with an increased odds in developing breast cancer in women (OR=1.56, 95%CI: 1.25, 1.95; 6 studies, 1506 participants)	No subgroup analysis conducted	
						Subgroup of congeners reported by only two studies<br>PCB 28<br>OR 2.39, 95%CI: 0.16 to -35.60; 2 studies, 135 participants<br>PCB 52<br>OR 0.98, 95%CI: 0.78 to 1.23; 2 studies, 130 participants<br>PCB 74<br>OR 0.94, 95%CI: 0.84 to 1.04; 2 studies, 334 participants unspecified<br>PCB 77<br>OR 1.20, 95%CI: 0.39 to 3.73; 2 studies, 113 participants<br>PCB 101<br>OR 1.02, 95%CI: 0.80 to 1.31; 2 studies, 130 participants unspecified<br>PCB 105	

Study details	Appraisal details	Participants	Plastic exposure	Health outcomes	Findings	Subgroup Findings	AMSTAR score
						OR 2.22, 95%CI: 1.18 to 4.17; 2 studies, 287 participants unspecified<br>PCB 126<br>OR 1.40, 95%CI: 0.78 to 2.50; 2 studies, 113 participants unspecified<br>PCB 167<br>OR 0.87, 95%CI: 0.07 to 10.71; 2 studies, 142 participants unspecified	
**Catalani et al., 2019 [96] - Occupational and environmental exposure to polychlorinated biphenyls and risk of non-Hodgkin lymphoma: a systematic review and meta-analysis of epidemiology studies. No COI declared.**							
**Last search**<br>Mar-18<br><br>**Study types**<br>Cohort (n=12)<br>case-control (n=18)<br><br>**Included studies in the review** = 30<br><br>**Included studies in the meta-analysis** = 30	Newcaste-Ottawa Scale. No interpretation provided by authors	Workers occupationally exposed to PCBs (telecommunication workers, electrical workers and transformer and capacitor workers) or populations living in areas with reported PCB exposure.<br><br>N = 309,975	**Type**<br>Polychlorinated biphenyl (PCB)<br><br>**Route**<br>Occupational, food contamination, or residents in polluted areas<br><br>**Measure**<br>Unspecified<br><br>**Exposure time**<br>1920-2008	Non-Hodgkin's lymphoma risk RR and 95%CI; Highest versus lowest quartile	No association between PCB 118 and non-Hodgkin's lymphoma RR 0.82, 95%CI: 0.53 to 1.10; 8 studies, 1,571 participants		6
					No association between PCB 138 and non-Hodgkin's lymphoma RR 0.93, 95%CI: 0.59 to 1.27; 8 studies, 1,571 participants		
					No association between PCB 153 and non-Hodgkin's lymphoma RR 1.10 95%CI: 0.68 to 1.53; 8 studies, 1,571 participants		
					No association between PCB 180 and non-Hodgkin's lymphoma		

Study details	Appraisal details	Participants	Plastic exposure	Health outcomes	Findings	Subgroup Findings	AMSTAR score
					RR 1.07, 95%CI: 0.67 to 1.47; 7 studies, 954 participants		
					No association between PCB 170 and non-Hodgkin's lymphoma RR 0.89, 95%CI: 0.58 to 1.21: 5 studies, 984 participants		
**Zani et al., 2013 [92]- Polychlorinated Biphenyls and Cancer: An Epidemiological Assessment. No COI reported.**							
**Last Search**<br>Dec-12<br><br>**Study types**<br>Case-control and cohort<br><br>**Included studies in the review** = 29<br><br>**Included studies in the meta-analysis** = 29	No critical appraisal was performed	General population and men/women occupationally exposed to PCBs (including workers from "Yusho" incident, Japan [1968] and "Yu-Cheng" incident, Taiwan [1979])<br><br>N = 16,067	**Type**<br>polychlorinated biphenyl (PCB)<br><br>**Route**<br>rice oil accidentally contaminated by PCBs and polychlorinated<br><br>**Measure**<br>dibenzofurans (PCDFs)<br>(ng/g Lipid)<br><br>**Exposure time**<br>single incident	Non-Hodgkin lymphoma<br>OR and 95%CIs; quantiles	Exposure to PCBs was associated with an increased OR of non-Hodgkin lymphoma for the highest compared to the lowest PCB serum levels (OR=1.40, 95%CI: 1.14–1.71; 11 studies, 4422 participants)	No association found for individual measures of exposure and non-Hodgkin's lymphoma in subgroup of cohort studies (OR= 1.34, 95%CI: 0.97, 1.86; 7 studies, participants unspecified)	2
				Breast Cancer<br>OR and 95%CIs; quantiles	No association between PCB serum levels and risk of breast cancer (OR=1.15, 95%CI: 0.92–1.43; 18 studies, 11,645 participants)		
**Zani et al., 2017 [95] - Do polychlorinated biphenyls cause cancer? A systematic review and meta-analysis of epidemiological studies on risk of cutaneous melanoma and non-Hodgkin lymphoma. No COI Reported**							
**Last search**<br>Dec-16<br><br>**Study types**<br>case-control (n=1)<br>cohort (n=10)	No critical appraisal was performed	Workers (both men and women) in capacitor and transformer producing factories, in electrical power,	**Type**<br>polychlorinated biphenyl (PCB)<br><br>**Route**<br>Unspecified (but occupational exposure)	Mortality due to melanoma standardized mortality ratios (SMR) and 95%CI	Positive association between occupational exposure to PCBs and mortality due to melanoma (SMR=1.32, 9%CI: 1.05, 1.64; 8	No subgroup analysis conducted	5

Study details	Appraisal details	Participants	Plastic exposure	Health outcomes	Findings	Subgroup Findings	AMSTAR score
**Included studies in the review** = 11<br><br>**Included studies in the meta-analysis** = 10		and in transformer manufacture and telecommunications industry<br><br>N = 217,048	**Measure**<br>ng/g lipid<br><br>**Exposure time**<br>occupational exposure		studies, 214241 participants).		
					There is a risk of exposure to PCBs and non-Hodgkin lymphoma (OR 1.5, 95%CI: 1.1 to 1.7; 6 studies, 2,540 participants).<br>Dose response<br>OR 1.42, 95%CI: 1.10 to 1.83; 5 studies, 2,668 participants, for PCB serum levels around 1000 ng/g lipid, compared to values lower than, or next to, 500 ng/g lipid.<br><br>No association between occupational exposure to PCBs and mortality due to non-Hodgkin lymphoma (SMR=0.94, 95%CI: 0.73, 1.23; 7 studies, 174207 participants).	No subgroup analysis conducted	
**Li et al., 2015 [83] - Mortality after exposure to polychlorinated biphenyls and polychlorinated dibenzofurans: A meta-analysis of two highly exposed cohorts. No COI reported.**							
**Last search**<br>No search<br><br>**Study types**<br>Cohort<br><br>**Included studies in the review** = 2	None	Adult men and women occupationally exposed to PCBs - workers from “Yusho” incident, Japan (1968) and “Yu-Cheng”	**Type**<br>polychlorinated biphenyls (PCB)<br><br>**Route**<br>Yusho (1968) and Yucheng (1979) food contamination events<br><br>**Measure**	All cause mortality<br>SMR (only reported where I^2 <50%)	Exposure to PCBs was associated with an increased all cause standardized mortality rate (SMR=1.1, 95%CI: 1.1, 1.2; 2 studies, 3467 participants)	Exposure to PCBs was associated with an increased all cause standardized mortality rate in males (SMR=1.2, 95%CI: 1.1, 1.3; 2 studies, 1690 participants)<br><br>No association between PCB and all cause standardized mortality in	4

Study details	Appraisal details	Participants	Plastic exposure	Health outcomes	Findings	Subgroup Findings	AMSTAR score
**Included studies in the meta-analysis** = 2		incident, Taiwan (1979)<br><br>N = 3,467	ICD-9 codes (cause of death)<br><br>**Exposure time**<br>Unspecified			females (SMR=1.1, 95%CI: 0.9, 1.2; 2 studies, 1777 participants)	
				All cancer mortality		Exposure to PCBs was associated with an increased all cancer standardized mortality rate in males (SMR=1.3, 95%CI: 1.1, 1.6; 2 studies, 1690 participants)	
						No association between PCB and all cancer standardized mortality in females (SMR=0.8, 95%CI: 0.5, 1.3; 2 studies, 1777 participants)	
				Stomach cancer mortality	No association between PCB and stomach cancer standardized mortality in females (SMR=0.3, 95%CI: 0.1, 1.1; 2 studies, 1777 participants)	No subgroup analysis conducted	
				Rectum cancer mortality	No association between PCB and stomach cancer standardized mortality in females (SMR=1.0, 95%CI: 0.2, 5.8; 2 studies, 1777 participants)	No subgroup analysis conducted	
				Liver cancer mortality	Exposure to PCBs was associated with an increased liver cancer standardized mortality rate in females (SMR=2.0, 95%CI: 1.1, 3.6; 2 studies, 1777 participants)	No subgroup analysis conducted	
				Pancreas cancer mortality	No association between PCB and pancreas	No subgroup analysis conducted	

Study details	Appraisal details	Participants	Plastic exposure	Health outcomes	Findings	Subgroup Findings	AMSTAR score
					cancer standardized mortality in females (SMR=1.1, 95%CI: 0.4, 3.75.8; 2 studies, 1777 participants)		
				Lung cancer mortality	Exposure to PCBs was associated with an increased lung cancer standardized mortality rate (SMR=1.5, 95%CI: 1.1, 2.1; 2 studies, 3467 participants)	Exposure to PCBs was associated with an increased lung cancer standardized mortality rate in males (SMR=1.2, 95%CI: 1.2, 2.3; 2 studies, 1690 participants)<br><br>No association between PCB and lung cancer standardized mortality in females (SMR=0.7, 95%CI: 0.3, 1.9; 2 studies, 1777 participants)	
				Breast (female) cancer mortality	No association between PCB and breast cancer standardized mortality in females (SMR=1.1, 95%CI: 0.4, 2.9; 2 studies, 1777 participants)	No subgroup analysis conducted	
				Uterus cancer mortality	No association between PCB and uterus cancer standardized mortality in females (SMR=1.1, 95%CI: 0.4, 3.4; 2 studies, 1777 participants)	No subgroup analysis conducted	
				Leukaemia mortality	No association between PCB and leukaemia standardized mortality in males (SMR=2.0, 95%CI: 0.6, 6.0; 2	No subgroup analysis conducted	

Study details	Appraisal details	Participants	Plastic exposure	Health outcomes	Findings	Subgroup Findings	AMSTAR score
					studies, 1690 participants)		
				Hypertension mortality	No association between PCB and hypertension standardized mortality (SMR=1.6, 95%CI: 0.9, 2.9; 2 studies, 3467 participants)	No association between PCB and hypertension standardized mortality in males (SMR=1.5, 95%CI: 0.7, 3.4; 2 studies, 1690 participants)<br><br>No association between PCB and hypertension standardized mortality in females (SMR=1.4, 95%CI: 0.3, 5.6; 2 studies, 1777 participants)	
				Heart disease mortality	Exposure to PCBs was associated with an increased heart disease standardized mortality rate (SMR=1.3, 95%CI: 1.0, 1.7; 2 studies, 3467 participants)	No subgroup analysis conducted	
				Cerebrovascular disease mortality	No association between PCB and cerebrovascular disease standardized mortality (SMR=1.0, 95%CI: 0.8, 1.29; 2 studies, 3467 participants)	No association between PCB and cerebrovascular disease standardized mortality in males (SMR=0.9, 95%CI: 0.6, 1.2; 2 studies, 1690 participants)<br><br>No association between PCB and cerebrovascular disease standardized mortality in females (SMR=1.1, 95%CI: 0.8, 1.5; 2 studies, 1777 participants)	
				Hepatic disease mortality	Exposure to PCBs was associated with an increased hepatic disease standardized	Exposure to PCBs was associated with an increased hepatic disease standardized mortality rate in	

Study details	Appraisal details	Participants	Plastic exposure	Health outcomes	Findings	Subgroup Findings	AMSTAR score
					mortality rate (SMR=1.5, 95%CI: 1.0, 2.4; 2 studies, 3467 participants)	males (SMR=1.9, 95%CI: 1.3, 2.8; 2 studies, 1690 participants)<br><br>No association between PCB and hepatic disease standardized mortality in females (SMR=1.0, 95%CI: 0.5, 1.9; 2 studies, 1777 participants)	

2.11 Other outcomes

Study details	Appraisal details	Participants	Plastic exposure	Health outcomes	Findings	Subgroup Findings	AMSTAR score
**Li et al., 2015 [83] - Mortality after exposure to polychlorinated biphenyls and polychlorinated dibenzofurans: A meta-analysis of two highly exposed cohorts. No COI reported.**							
**Last search**<br>No search<br><br>**Study types**<br>Cohort<br><br>**Included studies in the review** = 2<br><br>**Included studies in the meta-analysis** = 2	None	Adult men and women occupationally exposed to PCBs - workers from “Yusho” incident, Japan (1968) and “Yu-Cheng” incident, Taiwan (1979)<br><br>N = 3,467	**Type**<br>polychlorinated biphenyls (PCB)<br><br>**Route**<br>food contamination events<br><br>**Measure**<br>ICD-9 codes (cause of death)<br><br>**Exposure time**<br>Unspecified	Hepatic disease mortality	Exposure to PCBs was associated with an increased hepatic disease standardized mortality rate (SMR=1.5, 95%CI: 1.0, 2.4; 2 studies, 3467 participants)	Exposure to PCBs was associated with an increased hepatic disease standardized mortality rate in males (SMR=1.9, 95%CI: 1.3, 2.8; 2 studies, 1690 participants)<br>No association between PCB and hepatic disease standardized mortality in females (SMR=1.0, 95%CI: 0.5, 1.9; 2 studies, 1777 participants)	4
				All cause mortality SMR (only reported where I^2 <50%)	Exposure to PCBs was associated with an increased all cause standardized mortality rate (SMR=1.1, 95%CI: 1.1, 1.2; 2 studies, 3467 participants)	Exposure to PCBs was associated with an increased all cause standardized mortality rate in males (SMR=1.2, 95%CI: 1.1, 1.3; 2 studies, 1690 participants).<br>No association between PCB and all cause standardized mortality in females (SMR=1.1, 95%CI: 0.9, 1.2; 2 studies, 1777 participants)	

Note, the superscript number after year of publication indicates the number of the reference in the harvest plot figures in the main manuscript.
